# Stretching the Bisalkyne Raman Spectral Palette Reveals a New Electrophilic Covalent Motif

**DOI:** 10.1002/chem.202300953

**Published:** 2023-05-15

**Authors:** Manasa Punaha Ravindra, Martin Lee, Silviya Dimova, Craig F. Steven, Marie T. J. Bluntzer, Valerie G. Brunton, Alison N. Hulme

**Affiliations:** ^1^ School of Chemistry University of Edinburgh Joseph Black Building, David Brewster Road Edinburgh EH9 3FJ UK; ^2^ Edinburgh Cancer Research Institute of Genetics & Cancer University of Edinburgh Crewe Road South Edinburgh EH4 2XR UK

**Keywords:** covalent capture probes, DFT calculation, diynes, Pd−Cu cross-coupling, Raman spectroscopy

## Abstract

Small heteroaryl‐diyne (**Het‐DY**) tags with distinct vibrational frequencies, and physiologically relevant cLog *P* were designed for multiplexed bioorthogonal Raman imaging. Pd−Cu catalyzed coupling, combined with the use of Lei ligand, was shown to improve overall yields of the desired heterocoupled **Het‐DY** tags, minimizing the production of homocoupled side‐products. Spectral data were in agreement with the trends predicted by DFT calculations and systematic introduction of electron‐ rich/poor rings stretched the frequency limit of aryl‐capped diynes (2209–2243 cm^−1^). The improved Log *P* of these **Het‐DY** tags was evident from their diffuse distribution in cellular uptake studies and functionalizing tags with organelle markers allowed the acquisition of location‐specific biological images. LC–MS‐ and NMR‐based assays showed that some heteroaryl‐capped internal alkynes are potential nucleophile traps with structure‐dependent reactivity. These biocompatible **Het‐DY** tags, equipped with covalent reactivity, open up new avenues for Raman bioorthogonal imaging.

## Graphical abstract

**Raman bioorthogonal imaging**: Varying the electronics of the aryl end‐caps of bisaryl diynes (BADY) widened the palette for multiplexed Raman imaging. Electron‐poor end‐caps revealed the covalent reactivity of the aryl‐capped diynes, opening new avenues for covalent Raman imaging.

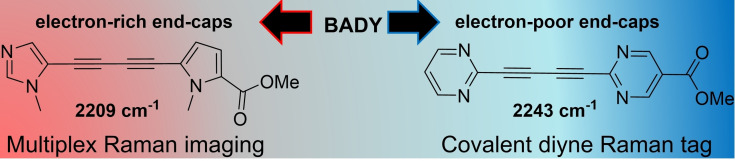

## Introduction

Raman microscopy is a powerful optical analytical method that measures the vibrational composition of a biological sample, allowing both non‐destructive and non‐invasive imaging of biomolecules.[^1^, ^2^] Alkynes have a large Raman scattering cross‐section in the cell‐silent region and are seldom found in Nature, rendering them an important spectroscopically bioorthogonal handle.[^3^, ^4^] In contrast to most fluorophores, their unidirectional vibrational mode gives very narrow Raman vibrational bands (FWHM ∼20 cm^−1^)^[5]^ allowing them to be readily adapted to multi‐channel imaging. Thus alkyne tagged Raman imaging (ATRI) has emerged as one of the most popular approaches to the Raman imaging of biomolecules (including DNA, RNA, proteins,^[6]^ lipids[^7^, ^8^, ^9^, ^10^, ^11^] and small biomolecules[^11^, ^12^]), organelles[^13^, ^14^, ^15^, ^16^] and drugs in recent years.[^17^, ^18^, ^19^, ^20^, ^21^, ^22^, ^23^]

Stimulated Raman scattering (SRS) based coherent Raman microscopy, is a non‐linear, resonantly enhanced technology that allows real‐time vibrational imaging of living cells and organisms.[^24^, ^25^, ^26^, ^27^, ^28^] Compared to spontaneous Raman, SRS offers improved sensitivity, spectral resolution and imaging speeds. SRS, used in tandem with either polyyne‐based Raman probes, or triple‐bond‐conjugated near‐infrared dyes coupled with isotopic substitutions, has allowed up to 24 resolvable ‘colours’ to be imaged.[^16^, ^29^] However, notwithstanding the many advantages, the large size, rigidity, planarity, low solubility and high lipophilicity of many polyyne probes challenge their wider application. When conjugated to important biomolecules or small molecular therapeutic agents, potential problems include, but are not limited to, perturbation of important biological functions including uptake, localization and target binding.[^30^, ^31^] Furthermore, the vibrational intensity variation with increasing alkyne units varies by multiple orders of magnitude, requiring multiple instrumental adjustments during the image acquisition process.

**Figure 1:**
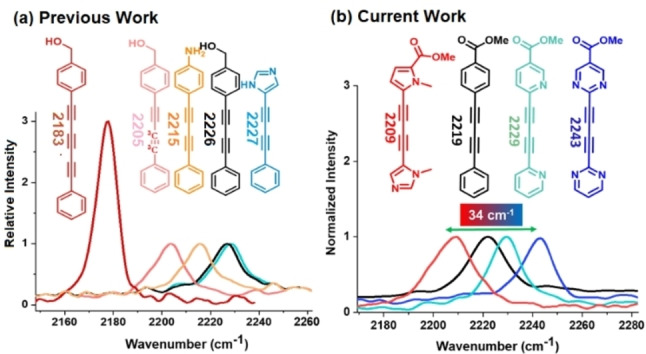
a) Raman probes from the Min and Graham groups.[^16^, ^32^] Ring modifications cause red and blue shifts relative to **BADY** (black); ^13^C isotopes or alkyne extension cause red shifts. b) Systematic replacement of phenyl rings in **BADY** (black) with electron rich (red) or electron poor (cyan and blue) heteroaromatic rings widens the diyne spectral palette (2209 cm^−1^–2243 cm^−1^).

While alkyne extension and ^13^C doping (Figure 1a) have been extensively exploited for frequency tuning of alkyne tags,^[16]^ an approach that has been largely overlooked is the tuning of π‐electron delocalization via modulation of end‐cap electronics. Electron‐withdrawing and electron‐donating ring substitutions have been shown to cause minor frequency shifts in aryl end‐capped alkynes, however in themselves these are not sufficient to facilitate multiplex imaging.[^16^, ^32^] In the current study (Figure 1b), we sought to demonstrate that electronic modulation of the end‐cap phenyl rings of **BADY** through heteroaryl substitution offers an alternative to the current strategies for multiplexed imaging.

## Results and Discussion

### Het‐DY tag design

**Figure 2:**
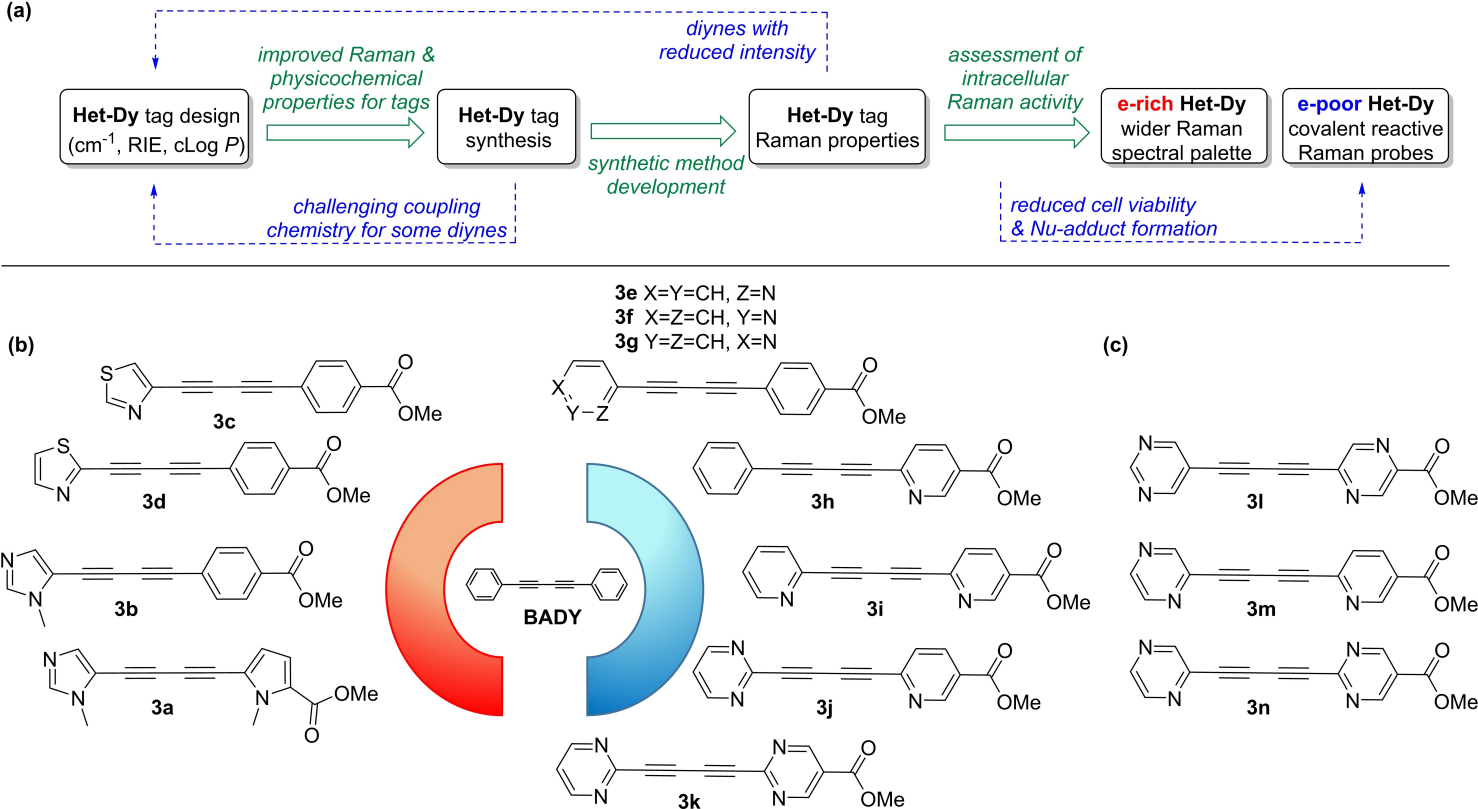
a) **Het**‐**DY** tag design workflow with green forward arrows representing developments highlighted in this paper and blue dotted arrows feedback points in **Het‐DY** tag development; b) Structures of **Het‐DY** tags **3 a**–**k**, electron rich and electron poor end‐cap rings cause a red‐shift and blue‐shift in the Raman vibrational frequency of alkynes; c) Structures **3 l**–**n** were designed to improve the physicochemical properties of the tags (cLog *P*<2) **3 h**–**j**, while retaining the incremental changes in Raman shift.

A significant challenge to the use of **BADY** or polyyne structures for bio‐imaging is their strong tendency to form π‐π stacked aggregates. Using ESI‐MS analysis and a series of all‐carbon **BADY** analogues, we recently showed that reduced compound aggregation correlates favorably with an improved cLog *P*.^[33]^ This earlier study underlines the pivotal need for improved physicochemical properties of conjugated alkyne Raman tags if they are to enter more widespread use. We predicted that electronic modulation via systematic replacement of the phenyl rings in **BADY** with heteroaromatic rings would not only induce π‐electron delocalization to generate new **Het‐DY** tags with frequencies tuned for multiplexed Raman imaging, but would also improve their log *P* while simultaneously maintaining their small molecular size (Figure 2a).

A library of **Het‐DY** tags was designed, with the phenyl end‐caps in **BADY** replaced either by electron‐rich rings (5‐membered heteroaromatic rings in place of the phenyl ring), or electron‐poor rings (nitrogen atom incorporation at the *ortho‐*, *meta*‐ and/or *para*‐ positions in the phenyl ring relative to alkyne substitution) to tune their Raman vibrational frequencies. Library members A1‐A94 (Supporting Information Table S1) were modeled using density functional theory (DFT) as their amide derivatives to best represent linkages between the tags and a target of interest in future applications and to enhance signal intensity.^[3]^ **Het‐DY** tags **3 a–n** (Figure 2b and c, Table 1) were selected from this library for experimental evaluation. Key parameters for selection of this subset included: i) a stepwise increase in predicted Raman frequencies (∼5 cm^−1^ shift per modification); ii) cLog *P* within the range 0–3.5; and iii) ease of **Het‐DY** tag synthesis, including hydrolysis of the ester functionality to allow attachment to a target of interest as the corresponding amide.

**Table 1:** Calculated Raman shifts; experimental Raman shifts of 10 mM DMSO solutions; relative intensities of 10 mM DMSO sample solutions compared to internal standard EdU (100 mM); and cLog *P* values of **Het‐DY** tags **3 a**–**n**.

Compd.	DFT Raman Shift [cm^−1^]^[a,b]^	Experimental Raman Shift [cm^−1^]	Relative Imaging Intensity Compared to EdU [RIE]^[c]^	cLog *P* ^[d]^
**3 a**	2198	2209	+	0.5
**3 b**	2210	2212	+	1.7
3 c*	2215	2225	+	3.2
**3 d**	2220	2216	++	3.4
**BADY**	2226	2219	++	4
**3 e**	2227	2226	++	3.1
**3 f**	2227	2219	++	2.7
**3 g**	2231	2225	++	2.7
**3 h**	2230	2223	+	3.1
**3 i**	2235	2229	‐^[e]^	2.2
**3 j**	2240	2236	+	2.1
**3 k**	2247	2243	+	2
**3 l**	2230	2229	‐^[e]^	0.3
**3 m**	2235	2231	+	0.9
**3 n**	2240	2233	‐^[e]^	1.1

[a] DFT calculations at B3YLP 6–31G(d,p) level with the 6–31G(d,p) double‐zeta plus polarization basis set were performed on the amide linked tags to accommodate frequencies and intensities of biologically imaged functionalized tags. [b] For the full list of DFT calculated Raman frequencies and relative signal intensities see Supporting Information Table S1. [c] ++ and+represent RIE ranges of 20–30 and 10–20, respectively. [d] The cLog *P* for each tag was calculated using ChemDraw 17.1. [e] Difficult to purify from homodimers and concentration of pure fractions not sufficient to acquire spectra with good intensity. [*] Tag **3 c** designed as an electron rich end‐capped **Het‐DY** showed a blue–shifted Raman frequency compared to **BADY**.

### Het‐DY tag synthesis

**Scheme 1:**
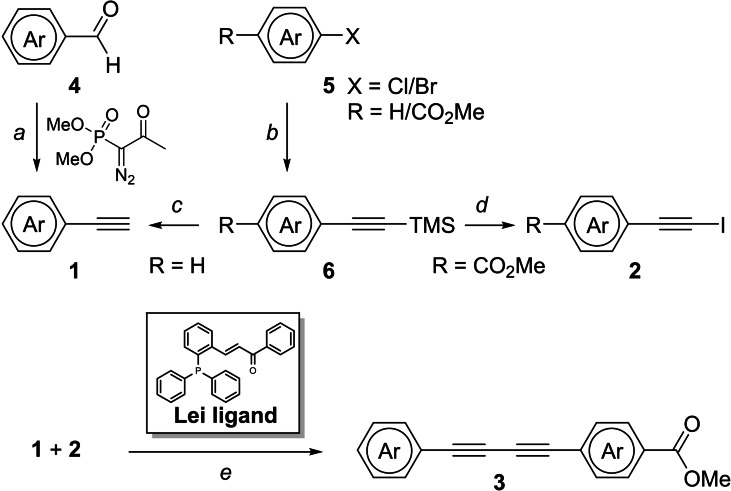
Synthesis of alkyne coupling partners, and Pd−Cu co‐catalyzed synthesis of unsymmetrical 1–3 diynes in the presence of the π‐acceptor Lei‐ligand. Reagents and conditions: a) K_2_CO_3_, MeOH, rt, 4 h; b) Pd(PPh_3_)_4_ (4 mol %), CuI (4 mol %), HC≡CSi(Me)_3_, Et_3_N, MeCN, μW, 70–100 °C, 1–5 h; c) K_2_CO_3_, THF‐MeOH, rt, 1 h; d) AgNO_3_ (5–50 mol %), NIS/NBS, DMF, rt, 1–3 h; e) Pd_2_(dba)_3_ (4 mol %), Lei ligand (4 mol %), CuI (2 mol %), Et_3_N, DMF, rt, 4 h.

Terminal alkynes **1** and activated haloalkynes **2** are the key intermediates required for the Pd−Cu co‐catalyzed synthesis of the selected 1,3‐diynes **3 a**–**n**; non‐commercial coupling partners were synthesized as shown in Scheme 1. Where possible, terminal alkynes **1** were accessed directly by Seyferth Gilbert homologation of commercial aldehydes **4**. Alternatively, Sonogashira coupling of commercial aromatic halides **5** and TMS acetylene under microwave conditions afforded the silyl protected alkynes **6**. TMS‐deprotection of these mono‐substituted arylalkyne intermediates under basic conditions gave terminal alkynes **1**. Activated haloalkynes **2** were obtained either by direct halogenation of commercial terminal alkynes (Supporting Information general procedure C), or by AgNO_3_‐catalysed halodesilylation of ester‐substituted TMS‐alkyne intermediates **6** with NIS/NBS (Scheme 1).

The key step in the synthesis of heterodimeric 1,3‐diynes is a C(sp)‐C(sp) cross‐coupling, which is often achieved via Cu‐catalysis in the presence of an excess (3–5 equiv.) of one of the alkyne coupling partners.^[34]^ Pd−Cu co‐catalysed coupling of terminal alkynes with activated bromo/iodoalkynes affords greater selectivity for synthesis of the unsymmetrical 1,3‐diyne without requiring the large stoichiometric excess.[^34^, ^35^] In the current study, we employed the Pd−Cu coupling conditions reported by Lei et al. for the synthesis of **3 b**–**n**.^[35]^ Pd_2_(dba)_3_, CuI and a π‐acceptor phosphine‐electron‐deficient olefin ligand, **Lei ligand**, which promotes the reductive elimination step, were used to give moderate to good cross‐coupling yields in the synthesis of compounds **3 b**–**n** (15–70 %) in a cleaner, greener and more economical alternative method (Scheme 1, Supporting Information Table S2).[^34^, ^35^, ^36^, ^37^] Excess alkyne coupling partners (**1**, **2**) were avoided, homocoupled side‐products and the difficulties associated with chromatographic separation of these side‐products from heterocoupled products **3** were also minimized. However, the yield of cross‐coupling reactions was lower in heteroaromatic substrates and steadily reduced upon each additional end‐cap ring nitrogen incorporated *ortho* to the alkyne (**3 e**, **3 i** and **3 j**) (Supporting Information Table S3).^[38]^ The synthesis of compound **3 k** in which all four *ortho‐*positions are occupied by nitrogen, failed. Replacing **Lei ligand** with the bulky (*t*‐Bu)_3_P ligand, which also facilitates faster reductive elimination, enabled its isolation albeit in a low yield. The susceptibility of the electron rich rings of the pyrrole or imidazole alkynes to halogenation under the NXS‐AgNO_3_ halodesilylation conditions required an alternative synthesis of **3 a**, which was achieved using a Cu/DMAP‐catalyzed Glaser coupling (Supporting Information general procedure D1)^[36]^ and an excess of the *N*‐methyl imidazole capped alkyne (5 equiv.) to afford the tag **3 a** (50 %).

### Het‐DY tag Raman properties

**Figure 3:**
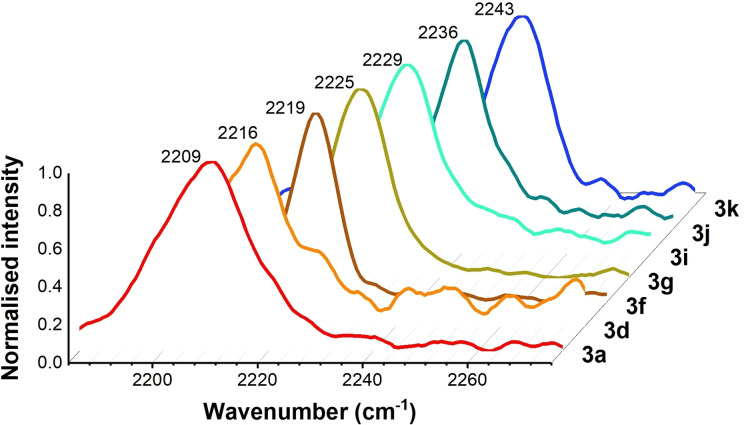
Selected Raman spectra showing incremental coarse‐tuned frequencies going from **3 a** to **3 k**. Normalized spontaneous Raman spectra (2180 cm^−1^ to 2280 cm^−1^) of 10 mM DMSO solutions.

**Het‐DY** tag **3 a**, end‐capped with electron rich *N*‐methyl imidazole and *N*‐methyl pyrrole rings (Figure 2b), was predicted by DFT calculations to show a significant red shift in its vibrational frequency compared to **BADY** (Table 1). This red shift is systematically reduced (Table 1, Supporting Information Table S1) as the electron rich rings are replaced with relatively electron poor rings in **3 b**–**d** (Figure 2b). Whilst the trend for the experimental spectral maxima of **Het‐DY** tags **3 a**, **3 b** and **3 d** was in agreement with DFT predictions (Table 1, Figure 3), the frequency range was somewhat reduced (**3 a** predicted 2198 cm^−1^, experimental 2209 cm^−1^) and 4‐thiazole capped **Het‐DY** tag **3 c** appeared out of sequence suggesting limitations to current DFT calculations. In contrast, end‐capping with electron poor rings is predicted to cause a blue shift (Table 1, Supporting Information Table S1) compared to **BADY**. DFT calculations for the **Het‐DY** tags **3 e**–**g** (Figure 2b) indicated that a single nitrogen incorporation, depending on its regioisomeric position (*ortho‐* and *para‐* to the alkyne in **3 e** and **3 g** respectively) causes a modest blue shift in frequency of 5 cm^−1^ compared to **BADY** (Table 1). Further 5 cm^−1^ blue‐shifts were predicted with additional *ortho*‐*N*‐incorporations in **3 i**–**k** (Figure 2b). Experimental maxima in the Raman spectra for tags **3 e**‐**k** were in close agreement with the DFT predictions (Table 1, Figure 3) and the trend of step‐wise increments in frequency going from one to four *ortho*‐*N*‐incorporations. Since DFT predictions showed that *meta*‐*N*‐incorporation did not substantially alter the Raman vibrational frequency, tags **3 l**–**n** (Figure 2c) were chosen as frequency matched analogues of tags **3 h**–**j** with improved physicochemical properties (cLog *P* <2) (Table 1).

### Intracellular Raman activity of Het‐DY tags

**Figure 4:**
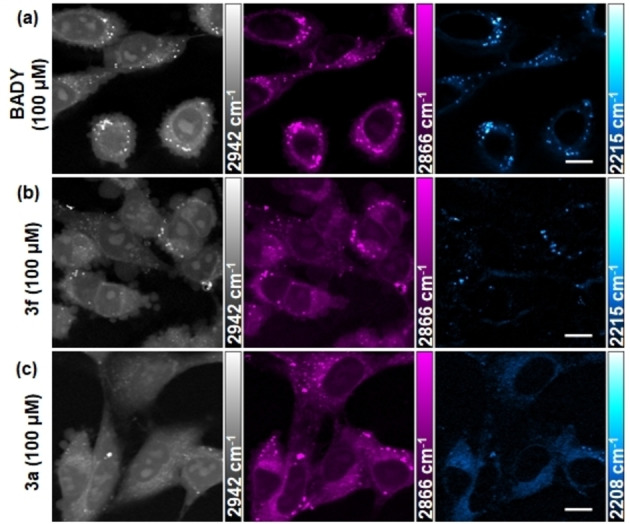
SRS images of ES2 cells treated with a) **BADY**, b) **3 f** and c) **3 a** were taken at three different wavenumbers: CH_3_, proteins 2942 cm^−1^ – grey scale; CH_2_, lipids 2866 cm^−1^ – magenta; and C≡C, alkyne, images taken at 2215 cm^−1^/2208 cm^−1^ with an off‐resonance image taken ∼30 cm^−1^ away subtracted – cyan hot. Cells were treated with 100 μM compound for 1 h. Scale bars: 10 μm.

The relative intensities of **BADY** and **Het‐DY** tags in DMSO solution (10 mM) compared to the internal standard EdU (100 mM) indicate that the high signal intensities of aryl‐aryl end‐capped diynes are largely retained (Table 1, Supporting Information Figure S1). The cLog *P* values of the **Het‐DY** tags vary by >3 Log units compared to **BADY** and fall within the optimum range of 0–3 for biological uptake and distribution of small molecules (Table 1, Supporting Information Table S1).^[39]^ In cells, **BADY** (cLog *P* 4) accumulates in lipid droplets, (Figure 4a). While the localization of **3 f** (cLog *P* 2.7) appears unchanged compared to **BADY** (Figure 4b), the more diffuse distribution of tag **3 a** (cLog *P* 0.5) was predominantly in the cytoplasm which may be explained by the greater reduction in cLog *P* (Figure 4c).

**Figure 5:**
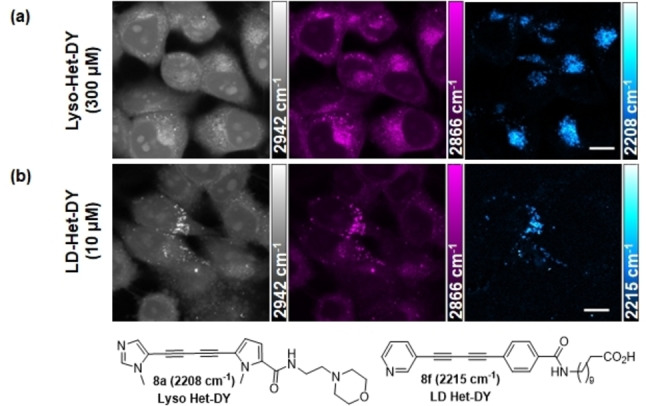
SRS images of ES2 cells treated with a) **Lyso‐Het‐DY** (300 μM, 1 h) and b) **LD‐Het‐DY** (10 μM, 24 h). Images were taken at: CH_3_, proteins 2942 cm^−1^ — grey scale; CH_2_, lipids 2866 cm^−1^ — magenta and C≡C, alkyne, images taken at 2208/2215 cm^−1^ with an off‐resonance image taken ∼30 cm^−1^ away subtracted – cyan hot. Scale bars: 10 μm.

The localization of **BADY** can be altered by tagging organelle targeting motifs to disparate parts of the cell, for example plasma membrane, mitochondria, lipid droplets and lysosomes (Supporting Information Figure S2). Several of the **Het‐DY** tags including **3 a** (2209 cm^−1^), **3 e** (2225 cm^−1^), **3 f** (2219 cm^−1^), **3 i** (2230 cm^−1^) and **3 k** (2243 cm^−1^) were selected for functionalization as organelle markers for multiplexed biorthogonal imaging. These markers were synthesized via ester hydrolysis of the **Het‐DY** tags **3** to the corresponding acids **7**, followed by amide coupling to afford the organelle specific markers **8** (Supporting Information Experimental). Despite the expected improvement in Log *P* due to heteroatom substitutions and concomitant improvement in intracellular distribution of the tags, imaging with some of the **Het‐DY** tags proved to be surprisingly challenging. Notwithstanding, tags **3 a** and **3 f** functionalized as imaging probes of lysosomes (**Lyso‐Het‐DY**, **8 a**) and lipid droplets (**LD‐Het‐DY**, **8 f**) were enriched in lysosomal (Figure 5a) and lipid rich regions (Figure 5b) of the cells respectively as expected.

Viability studies of **Het‐DY** tags **3 a‐n** were performed in ES2 cells (EC_50_ 0.54 ‐ >100 μM, Supporting Information Figure S3). The EC_50_ values following incubation with the **Het‐DY** tags for 72 h show that only the alkyne tags with electron rich (**3 a** and **3 b**) or neutral (**BADY**) and *meta*‐*N*‐incorporated end‐caps (**3 f**) are tolerated well (EC_50_ values ≥100 μM). Viability reduced with an *ortho‐* or *para‐*nitrogen in the end cap relative to the alkyne: **3 e** (EC_50_ 17.57 μM) and **3 g** (EC_50_ 24.10 μM); or with increased number of *ortho*‐nitrogens: **3 i** (EC_50_ 1.63 μM), **3 j** (EC_50_ 0.54 μM) and **3 k** (EC_50_ 2.35 μM).

### Covalent reactivity of Het‐DY tags

**Figure 6:**
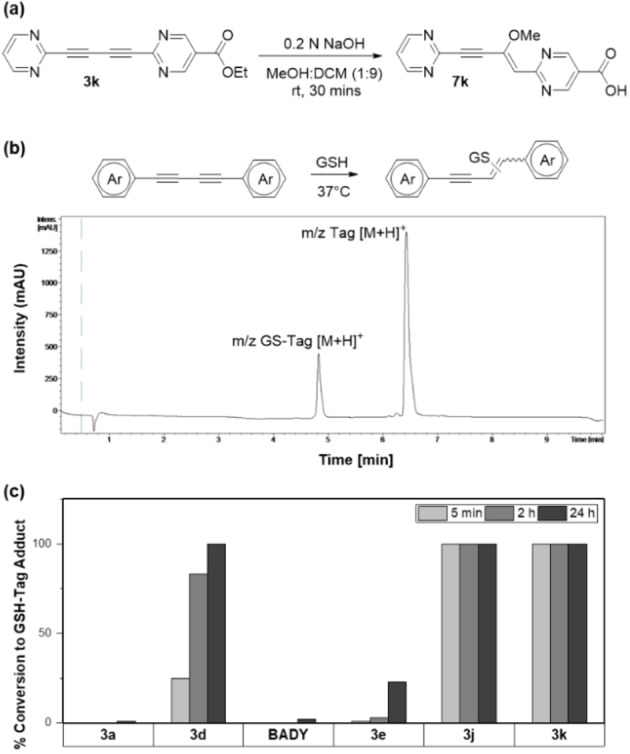
a) Methanolic hydrolysis of **3 k** at room temperature resulted in Michael addition; only one of the possible Michael addition products is shown. b) Representative LC–MS spectrum with detection at 254 nm; formation of GS‐Tag adduct in ACN:PBS buffer (1 : 1) at 37 °C. c) Relative reactivities of tags **3 a**, **3 d**, **BADY**, **3 e**, **3 j** and **3 k** calculated at 5 min, 2 h and 24 h post addition of the diyne tags to GSH in ACN‐PBS buffer at 37 °C. Tags **3 a** and **BADY** formed 1–2 % adducts over 24 h whilst tag **3 e** showed ∼25 % conversion. Tags **3 d**, **3 j** and **3 k** showed complete conversion to GS‐adducts; **3 j** and **3 k** showed complete conversion within 5 min post addition.

Terminal alkynes have been shown to have latent reactivity towards thiol nucleophiles.^[40]^ Alkynyl heterocycles with terminal and methyl‐substituted acetylenes designed to mimic Michael acceptor‐like systems show cysteine‐selective reactivity.[^41^, ^42^] While reduced cell viability of some of the **Het‐DY** tags provided an early indication, their electrophilic nature was first revealed in the process of base‐catalyzed hydrolysis of **3 k**. Aqueous hydrolysis of **3 k** resulted in multiple degradation products. However, methanolic hydrolysis under mildly basic conditions provided clear NMR evidence of the formation of Michael addition products (Figure 6a and Supporting Information Experimental). LC–MS based glutathione (GSH) reactivity assays (Figure 6b and c) of **BADY** and **Het‐DY** tags, with reaction progression recorded at 5 min, 2 h and 24 h to identify GS‐Tag adduct formation, shed light on their (a) cysteine trapping reactivity and (b) relative reactivity. Perhaps surprisingly, given the widespread use of the aryl‐capped diynes in ATRI bioimaging to date, all the tags, including **BADY**, formed GS‐adducts (Supporting Information Figure S4), albeit at different rates. Tags **3 a** and **BADY** were most stable, showing relatively low (1–2 %) conversion to the adduct over 24 h. Tag **3 d** formed (∼25 %) GS‐adduct within 5 min of GSH addition and reacted completely over 24 h. Correlating with the trend in increasing electron deficiency from **3e** to **3 k**, **3 e** formed ∼25 % adduct over 24 h, and **3 j** and **3 k** reacted completely within 5 min of GSH addition. Finally, NMR based GSH‐reactivity analysis of **3 a**, **BADY** and **3 k** reaffirmed their relative reactivity. Despite forming GS‐adducts as visible on LC–MS, **3 a** and **BADY** show no changes in their ^1^H NMR 24 h post GSH addition, indicating their low reactivity. However, in accordance with results from the LC–MS assays, within 5 min of GSH addition, ^1^H NMR of **3 k** showed multiple new peaks in the region corresponding to the alkene CH(sp^2^) protons of the Michael addition products (Supporting Information Figure S5). Together, the above results suggest that diynes can be tailored via careful end‐cap modifications as biocompatible imaging agents for multiplexing, or as covalently reacting Michael acceptors via conjugation with electron‐deficient heterocyclic end caps.

## Conclusions

We have rationally designed and synthesized **Het‐DY** tags **3 a**–**n** for fine‐tuned Raman frequencies. A frequency difference of 34 cm^−1^ was achieved between the two diyne tags **3 a** and **3 k**, which is considerably larger than single ^13^C incorporation (∼10 cm^−1^) and marginally higher than dual ^13^C incorporation (∼20 cm^−1^). To fully exploit diyne ATRI for multiplexing, we recommend incorporations of electron rich end‐caps in the design of new diyne Raman probes. The many advantages of the Pd−Cu co‐catalyzed synthesis of 1,3‐diynes (i. e., clean, convenient, efficient, and versatile), validate this method for further screening in the synthesis of a broad range of unsymmetrical 1,3‐diynes, specifically extending its scope to heteroaromatic substrates. We also demonstrate for the first time that electron deficient, aryl‐capped diyne Raman tags are nucleophile traps. Covalent capture alkyne probes offer an excellent opportunity to develop new and unexplored avenues in ATRI including high resolution imaging due to cellular trapping, real time tracking aided by the shift in the Raman activity of the alkyne and the alkyne‐nucleophile adducts, and the determination of covalent reaction kinetics *in cellulo*.

## Experimental Section

### General procedure for Pd‐catalyzed Cadiot‐Chodkievicz (CC) cross‐coupling

To an oven‐dried 2‐necked RBF with an oven‐dried PTFE‐coated magnetic stir‐bar, Pd_2_(dba)_3_ (4 mol %), Lei ligand (4 mol %), and CuI (2 mol %) were added. Anhydrous DMF (2 M) was added via a syringe and the mixture vacuum purged with nitrogen for three cycles. After stirring the mixture under nitrogen for 10 min, a vacuum purged and N_2_‐filled solution of terminal acetylene **1** (1.2 equiv.), in anhydrous DMF (1 M) was added via a syringe, followed by TEA (2 equiv.). The reaction mixture was stirred for another 5 min, then a vacuum purged and N_2_‐filled solution of haloacetylene **2** (1.0 equiv.) in anhydrous DMF (1 M) was added last via a syringe. The system was stirred at room temperature under N_2_ for 4 h. Reaction progress was monitored by TLC, which showed loss of starting material and appearance of three new spots. Upon completion, a minimum amount of MeOH and Celite (3× weight of the crude) were added and the solution was evaporated to afford a plug. The resulting plug was loaded on to an automatic flash column for purification. Fractions with desired R_*f*_ (TLC) were pooled and evaporated to afford the heterocoupled products **BADY**, **3 b**–**n**.

Additional references cited within the Supporting Information.[^43^, ^44^, ^45^, ^46^, ^47^, ^48^, ^49^, ^50^, ^51^, ^52^, ^53^, ^54^, ^55^, ^56^, ^57^, ^58^, ^59^, ^60^]

## Conflict of interest

The authors declare no conflict of interest.

1

## Supporting information

As a service to our authors and readers, this journal provides supporting information supplied by the authors. Such materials are peer reviewed and may be re‐organized for online delivery, but are not copy‐edited or typeset. Technical support issues arising from supporting information (other than missing files) should be addressed to the authors.

Supporting Information

## Data Availability

The data that support the findings of this study are available in the supplementary material of this article.
