# Supplementary material for: Stretching the Bisalkyne Raman Spectral Palette Reveals a New Electrophilic Covalent Motif
Source: Chemistry. 2023 May 15;29(38):e202300953. doi: 10.1002/chem.202300953 (PMC10946950; doi:10.1002/chem.202300953)
Supplement: Supplementary file 1 — Supporting Information [file CHEM-29-0-s001.pdf]

Chemistry–A European Journal

Supporting Information

Stretching the Bisalkyne Raman Spectral Palette Reveals a New Electrophilic Covalent Motif

Manasa Punaha Ravindra, Martin Lee, Silviya Dimova, Craig F. Steven, Marie T. J. Bluntzer, Valerie G. Brunton, and Alison N. Hulme*

Table of Contents

		Page
1.	General information	S1
2.	Computational Methods	S1
3.	Experimental for the Synthesis of Het-DY tags, BADY and Organelle Markers	S4
4.	Spontaneous Raman Spectroscopy	S14
5.	Sample Preparation for SRS microscopy, Image Acquisition and Analysis	S14
6.	Cell Viability Assay	S16
7.	GSH-reactivity Assay	S17
8.	^{1}H and ^{13}C NMR spectra for compounds **3a**-**n**, **8a** and **8f**	S19

1. General Information

Water-sensitive reactions were performed in anhydrous solvents under positive pressure of nitrogen (N_2). Anhydrous solvents used were either procured from an onsite solvent purification system and were transferred under N_2 or were purchased (Aldrich, Alfar Aeser, Fisher Scientific and Fluorochem), and were used without further purification. All commercially available compounds (Aldrich, Alfar Aeser, Fisher Scientific and Fluorochem) were used as received. NMR: Spectra were acquired on Bruker AvanceIII 400, 500 MHz and Avance Neo 600 MHz NMR spectrometers in the solvents indicated; chemical shifts (δ) are given in ppm relative to TMS, coupling constants (J) in Hz. The solvent signals were used as references and the chemical shifts converted to the TMS scale for ^{1}H and ^{13}C and H_3PO_4 scale for ^{31}P ($CDCl_3$: δ_C = 77.0 ppm; residual $CHCl_3$ in CDCl3: δ_H = 7.26 ppm; $(CD_3)_2SO$: δ_C = 39.52 ppm, residual $(CH_3)_2SO_3$ in $(CD_3)_2SO$: δ_H = 2.50 ppm). NMR spectra were processed using MestrNova software. MS ESI-MS were acquired on ESQ3000 (Bruker). Melting points were recorded on a Buchi B-540 melting point apparatus. HPLC analyses were conducted on a Shimadzu instrument (pump LC-20AD, autosampler SIL-20AC, column oven CTO-20AC, diode array detector SPD-M20A, controller CBM-20A, ESI detector and software Labsolutions) with an ZORBAX Eclipse Plus C18(1.8 µm, 3.0 or 4.6 mm ID × 50 mm) (Agilent). A binary gradient of MeCN in water or aq. ammonium bicarbonate buffer (20 mmol. pH 9) was used at a flow rate of 0.5 (3.0 mm ID) or 0.8 (4.6 mm ID) mL/min. The oven temperature was kept at 35 °C and the detection wavelength at 254 nm. LC-MS/MS experiments were performed using Agilent1200 instrument (pump Agilent G1312B SL binary, AutoSampler Agilent G1367A WP, Agilent G1316B Column oven, Agilent G1315C Starlight DAD) with a Phemoenex Kinetex C18 50×2.1 column and gradient methods of 10.02 minutes using 0.1% formic acid/water and 0.1% formic acid/MeCN as eluents. cLog P values were calculated on ChemDraw Prime. IUPAC names of the chemical structures were generated on Marvinsketch. The spontaneous Raman spectra were acquired using a confocal Raman spectrometer (inVia Raman microscope, Renishaw) at room temperature. A 297 mW (206 mW after objective) 785 nm diode laser was used to excite the sample through a 10×/50×/100×, N. A. 0.75 objective (Leica Biosystems) at 100% laser power. The recorded spectral range for grating 1200 g mm^{-1} was 400-3000 cm^{-1}. Data acquisition was performed during 40 s using the WiRE 3.4 (Renishaw) software. For in vitro studies, phenol red was purchased from Thermofisher, DMEM was purchased from Gibco, Pen/Strep was purchased from Thermofisher, and L-Glutamine was purchased from Thermofisher.

2. Computational Methods

The geometries of compounds evaluated in Table S1 were optimised at the B3LYP/6-31G(d,p) level of theory in the gas phase, using the Gaussian 09 Programme. Vibrational frequencies and Raman scattering activities, obtained from the polarizability derivatives, were calculated analytically.

DFT Predicted Raman Activity and cLog *P* Values of the Designed Het-DY tags

Table S1. DFT studies of the designed Het-DY analogues. Compounds **A1**-**14** were prioritized in the list to match with the compounds that were synthesized. Compounds **A15**-**74** are in the order of a systematic *N* atom incorporation, up to a maximum of 2 Ns in each ring. The 5-membered electron rich heteroaromatic rings were incorporated in **A75**-**94** from a published database of rings in drugs.[43]

No.	Structure	Freq (cm^{-1})	I_{ram}% compared to **BADY**	cLog *P*
**A1**	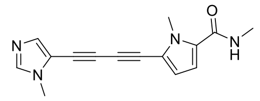	2198	63	-0.19
**A2**	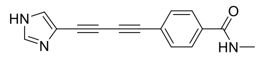	2223	97	1.1
**A3**	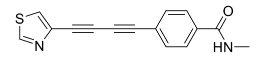	2215	156	2.5
**A4**	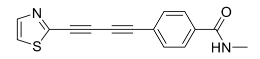	2220	116	2.78
**A5**	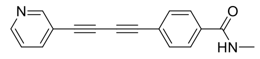	2227	95	2.05
**A6**	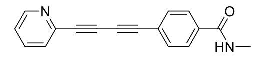	2227	93	2.47
**A7**	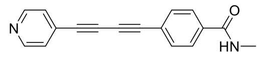	2231	103	2.05
**A8**	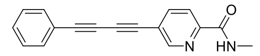	2229	108	2.47
**A9**	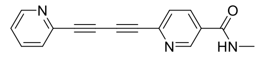	2236	90	1.56
**A10**	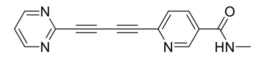	2241	94	1.423
**A11**	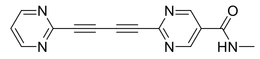	2247	87	1.3
**A12**	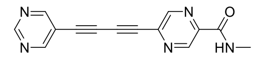	2230	99	-0.38
**A13**	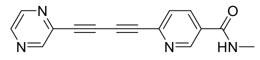	2231	101	0.22
**A14**	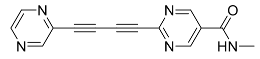	2240	94	0.09
**A15**	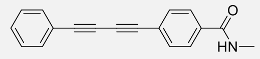	2226	100	3.38
**A16**	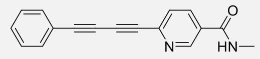	2226	108	2.47
**A17**	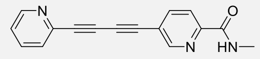	2231	103	1.56
**A18**	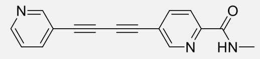	2227	103	1.13
**A19**	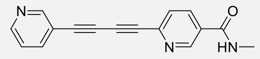	2230	103	1.13

No.	Structure	Freq (cm^{-1})	I_{ram}% compared to **BADY**	cLog *P*
**A20**	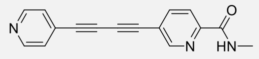	2229	96	1.13
**A21**	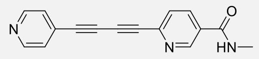	2233	96	1.13
**A22**	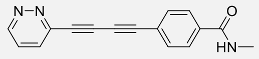	2233	88	2.08
**A23**	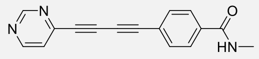	2232	88	1.87
**A24**	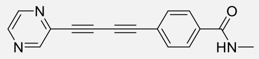	2229	104	1.13
**A25**	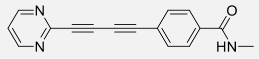	2235	96	2.34
**A26**	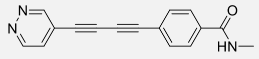	2226	100	1.65
**A27**	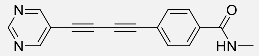	2227	101	1.45
**A28**	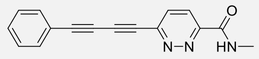	2229	100	2.50
**A29**	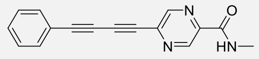	2226	108	1.56
**A30**	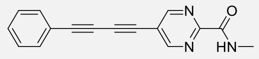	2225	105	2.34
**A31**	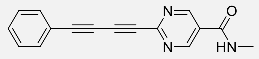	2231	101	2.34
**A32**	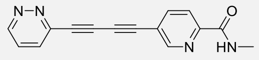	2233	94	1.16
**A33**	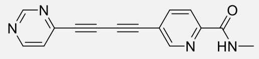	2232	88	0.96
**A34**	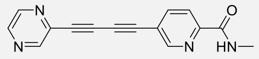	2231	93	0.22
**A35**	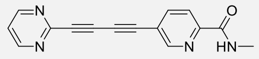	2236	93	1.43
**A36**	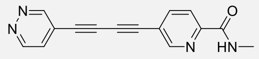	2227	97	0.74
**A37**	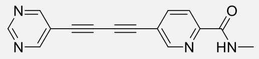	2227	98	0.54
**A38**	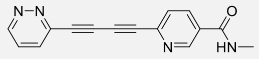	2238	90	1.16

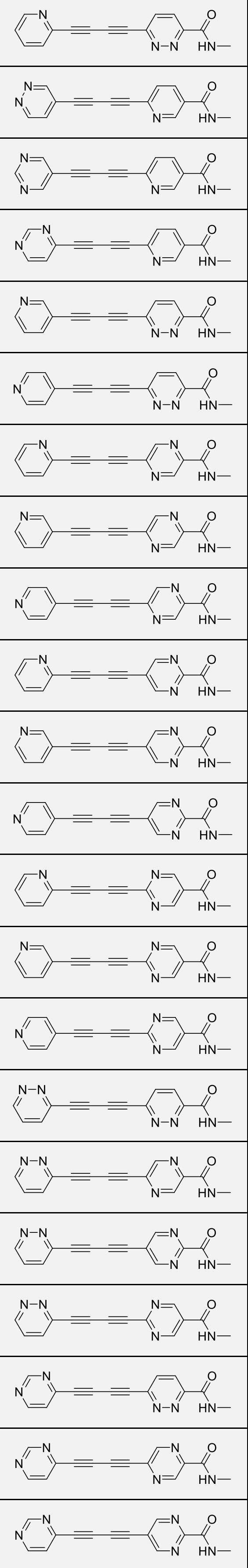

No.	Structure	Freq (cm^{-1})	I_{ram}% compared to **BADY**	cLog *P*
**A39**		2237	92	0.96
**A40**		2232	97	0.74
**A41**		2231	98	0.54
**A42**		2237	95	1.59
**A43**		2231	95	1.16
**A44**		2235	87	1.16
**A45**		2234	104	0.64
**A46**		2229	99	0.22
**A47**		2232	97	0.22
**A48**		2231	99	1.43
**A49**		2226	100	1
**A50**		2230	88	1
**A51**		2239	96	1.43
**A52**		2234	96	1
**A53**		2238	90	1
**A54**		2239	84	1
**A55**		2237	95	0.25
**A56**		2234	91	1.03
**A57**		2243	88	1.03
**A58**		2241	79	0.99
**A59**		2237	92	0.05
**A60**		2233	88	0.83

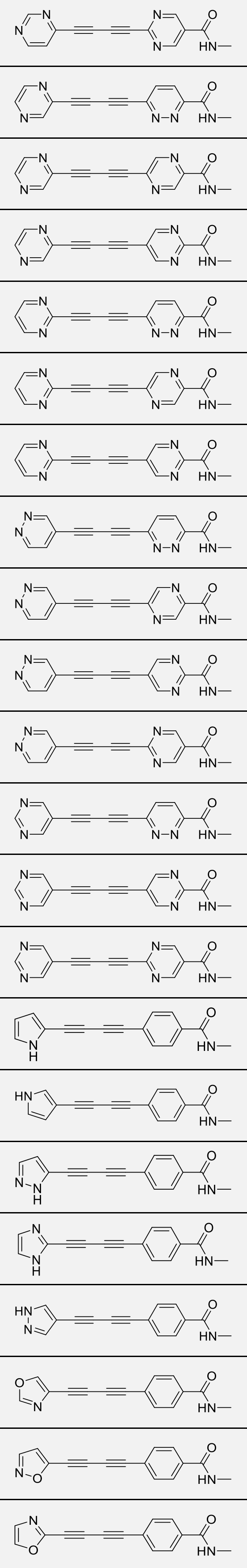

No.	Structure	Freq (cm^{-1})	I_{ram}% compared to **BADY**	cLog *P*
**A61**		2244	85	0.83
**A62**		2237	93	0.25
**A63**		2234	99	-0.69
**A64**		2230	98	0.09
**A65**		2244	86	1.46
**A66**		2241	94	0.51
**A67**		2238	90	1.3
**A68**		2235	88	0.77
**A69**		2231	98	-0.17
**A70**		2227	93	0.61
**A71**		2239	89	0.61
**A72**		2233	87	0.57
**A73**		2227	95	0.41
**A74**		2236	90	0.41
**A75**		2210	113	1.93
**A76**		2222	88	1.87
**A77**		2222	93	1.54
**A78**		2230	87	1.34
**A79**		2228	80	1.49
**A80**		2233	74	1.18
**A81**		2225	91	1.61
**A82**		2228	97	1.41

No.	Structure	Freq (cm^{-1})	I_{ram}% compared to **BADY**	cLog *P*
**A83**	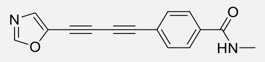	2220	96	0.81
**A84**	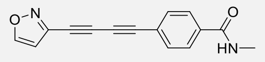	2238	67	1.98
**A85**	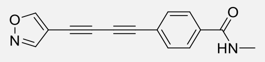	2233	72	1.56
**A86**	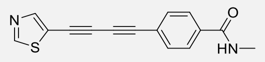	2215	110	2.18
**A87**	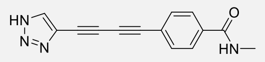	2234	75	1.52
**A88**	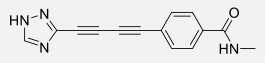	2241	71	1.93

No.	Structure	Freq (cm^{-1})	I_{ram}% compared to **BADY**	cLog *P*
**A89**	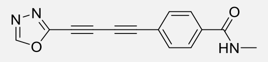	2233	77	1.16
**A90**	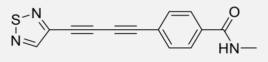	2231	92	2.96
**A91**	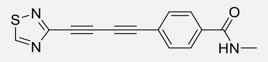	2237	86	3.37
**A92**	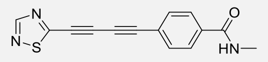	2220	99	3.13
**A93**	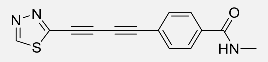	2223	97	2.53
**A94**	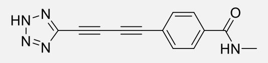	2242	65	0

3. Experimental for the Synthesis of Het-DY tags, BADY and Organelle Markers

A. General Procedure for Sonogashira Coupling:

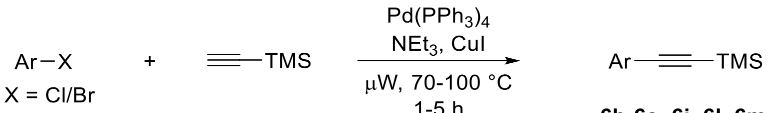

5 **6b-6e, 6j, 6l, 6m** (56%-92%)

To an oven-dried microwave vial with an oven-dried PTFE-coated magnetic stir-bar and aromatic halide **5** (1 eq), anhydrous ACN (2 M) was added. To the solution, $Pd(PPh_3)_4$ (4 mol%), CuI (4 mol%), TMS acetylene (1.2 eq) and TEA (3 eq) were added and stirred at 70-100 ˚C for 1-5 h under microwave conditions. Reaction progress was monitored by TLC which showed loss of starting material and appearance of a new product spot. After completion, the reaction mixture was diluted with ice-cold EtOAc, and filtered. To the filtrate, Celite (3× weight of the crude) was added and the solvent was evaporated to afford a plug. The resulting plug was loaded on to an automatic flash column for purification (SiO_2, 12 g, 40-60 mm ø, 0-100% PE/EtOAc or DCM/MeOH, ca. 10 mL fractions). Fractions with desired R_f (TLC) were pooled and evaporated to afford the trimethylsilyl acetylenes **6b-6e**, **6j**, **6l**, and **6m** in 56%-92% yields.[44]

Methyl 1-methyl-5-[2-(trimethylsilyl)ethynyl]-1*H*-pyrrole-2-carboxylate (6b)

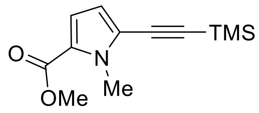

2 mmol scale reaction, synthesized from methyl 5-bromo-1-methyl-1*H*-pyrrole-2-carboxylate **5b**,[45] 100 ˚C, reaction went to completion in 1 h, purified by flash column chromatography (SiO_2, 4 g, 40-60 mm ø, 5% EtOAc in PE, ca. 10 Ml, R_f = 0.66 P:E 100:1) orange oil, 310 mg, 66% yield. **^{1}H NMR** (600 MHz, $CDCl_3$) δ 6.86 (d, *J* = 4.1 Hz, 1H, Ar-*H*), 6.40 (d, *J* = 4.1 Hz, 1H, Ar-*H*), 3.98 (s, 3H, O*CH₃*), 3.84 (s, 3H, N*CH₃*), 0.28 (s, 9H, Si*(CH₃)₃* ppm. **^{13}C NMR** (126 MHz, DMSO-d_6) δ 160.63, 123.77, 122.00, 117.05, 114.84, 101.85, 95.86, 51.80, 34.39, 0.19 ppm. **HRMS (ESI)**: calcd for $C_{12}H_{17}N_1O_2Si_1Na_1$ [M+Na]$^+$: 258.09208; found: 258.0924.

Methyl 6-[2-(trimethylsilyl)ethynyl]pyridine-3-carboxylate (6c)

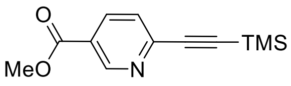

5 mmol scale, synthesized from methyl 6-chloropyridine-3-carboxylate, 100 ˚C, reaction went to completion in 2 h, purified by flash column chromatography (SiO_2, 12 g, 40-60 mm ø, 5% EtOAc in PE, ca. 10 mL, R_f = 0.62 P:E 20:1), yellow solid, 1070 mg, 92% yield. **^{1}H NMR** (500 MHz, $CDCl_3$) δ 9.19 (br s, 1H, Ar-*H*), 8.29 (dd, *J* = 8.4, 2.1 Hz, 1H, Ar-*H*), 7.56 (d, *J* = 8.4 Hz, 1H, Ar-*H*), 3.98 (s, 3H, O*CH₃*), 0.30 (s, 9H, Si*(CH₃)₃*) ppm. **^{13}C NMR** (151 MHz, $CDCl_3$) δ 165.28, 151.00, 146.61, 137.15, 126.76, 124.83, 103.04, 98.49, 52.62, -0.58 ppm. The spectroscopic data are in agreement with those reported in the literature.[46]

Methyl 2-[2-(trimethylsilyl)ethynyl]pyrimidine-5-carboxylate (6d)

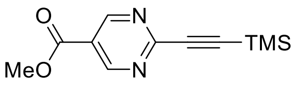

10 mmol scale reaction, synthesized from methyl 2-chloropyrimidine-5-carboxylate, 90 ˚C, reaction went to completion in 2 h, purified by flash column chromatography SiO_2, 24 g, 40-60 mm ø, 25% EtOAc in PE, ca. 10 mL, R_f = 0.25 P:E 2:1), brown oil, 1.54 g, 66% yield. **^{1}H NMR** (600 MHz, $CDCl_3$) δ 9.25 (s, 2H, Ar-*H*), 4.01 (s, 3H, O*CH₃*), 0.34 (s, 9H, Si*(CH₃)₃*) ppm. **^{13}C NMR** (151 MHz, $CDCl_3$) δ 163.71, 158.27, 154.78, 122.28, 101.96, 98.43, 52.83, -0.60 ppm. **HRMS (ESI)**: calcd for $C_{11}H_{14}N_2O_2Si_1Na$ [M+Na]$^+$: 257.07168; found 257.0714.

Ethyl 2-[2-(trimethylsilyl)ethynyl]pyrimidine-5-carboxylate (6d-Et)

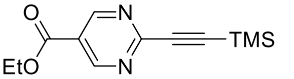

30 mmol scale reaction, synthesized from ethyl 2-chloropyrimidine-5-carboxylate, 90 ˚C, reaction went to completion in 2 h, purified by flash column chromatography (SiO_2, 12 g, 40-60 mm ø, 25% EtOAc in PE, ca. 10 mL, R_f = 0.3 P:E 2:1), yellow oil, 3520 mg, 47% yield. **^{1}H NMR** (600 MHz, $CDCl_3$) *δ* 9.24 (s, 2H, Ar-*H*), 4.47 (q, *J* = 7.1 Hz, 2H, O*CH₂*), 1.45 (t, *J* = 7.1 Hz, 3H, CH_2*CH_3*), 0.33 (s, 9H, Si*$(CH_3)_3$*) ppm. **^{13}C NMR** (151 MHz, $CDCl_3$) 163.26, 158.25, 154.70, 122.56, 101.99, 98.25, 62.11, 14.23, -0.58 ppm. **HRMS (ESI)**: calcd for $C_{12}H_{17}N_2O_2Si_1$ $[M+H]^+$: 249.10538; found 257.1054.

Methyl 5-[2-(trimethylsilyl)ethynyl]pyrazine-2-carboxylate (6e)

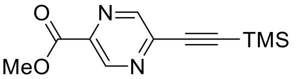

2.5 mmol scale reaction, synthesized from methyl 5-bromopyrazine-2-carboxylate, 80 ˚C, reaction went to completion in 1 h, purified by flash column chromatography (SiO_2, 4 g, 40-60 mm ø, 5% EtOAc in PE, ca. 10 mL, R_f = 0.59 P:E 3:1), yellow waxy solid, 390 mg, 56% yield. **Mp** 48-50 ˚C. **^{1}H NMR** (500 MHz, $CDCl_3$) *δ* 9.26 (d, *J* = 1.4 Hz, 1H, Ar-*H*), 8.77 (d, *J* = 1.4 Hz, 1H, Ar-*H*), 4.07 (s, 3H, O*CH_3*), 0.33 (s, 9H, Si*$(CH_3)_3$*) ppm. **^{13}C NMR** (126 MHz, $CDCl_3$) δ 164.01, 147.09, 145.60, 142.51, 140.69, 103.86, 100.25, 53.34, -0.33 ppm. **HRMS (ESI)**: calcd for $C_{11}H_{14}N_2O_2Si_1Na_1$ $[M+Na]^+$: 257.07168; found: 257.0726.

2-[2-(Trimethylsilyl)ethynyl]pyrimidine (6j)

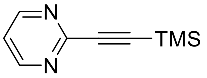

5 mmol scale reaction, synthesized from 2-bromopyrimidine, 70 ˚C, reaction went to completion in 3 h, purified by flash column chromatography (SiO_2, 12 g, 12 g, 40-60 mm ø, 5% EtOAc in PE, ca. 10 mL, R_f = 0.45 P:E 3:2), brown oil, 668 mg, 76% yield. **^{1}H NMR** (600 MHz, $CDCl_3$) *δ* 8.74 (d, *J* = 4.9 Hz, 2H, Ar-*H*), 7.26 (t, *J* = 4.9 Hz, 1H, Ar-*H*), 0.32 (s, 9H, Si*$(CH_3)_3$*) ppm. **^{13}C NMR** (151 MHz, $CDCl_3$) *δ* 157.22, 152.61, 120.03, 102.29, 94.51, -0.49 ppm. The spectroscopic data are in agreement with those reported in the literature.[47]

5-[2-(Trimethylsilyl)ethynyl]pyrimidine (6l)

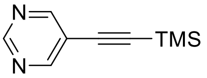

5 mmol scale reaction, synthesized from 5-bromopyrimidine, 100 ˚C, reaction went to completion in 1 h, purified by flash column chromatography (SiO_2, 12 g, 40-60 mm ø, 5% EtOAc in PE, ca. 10 mL, R_f = 0.89 P:E 3:1), brown waxy solid, 794 mg, 90% yield. **^{1}H NMR** (500 MHz, $CDCl_3$) *δ* 9.12 (s, 1H, Ar-*H*), 8.80 (s, 2H, Ar-*H*), 0.25 (s, 9H, Si*$(CH_3)_3$*) ppm. **^{13}C NMR** (126 MHz, $CDCl_3$) *δ* 159.06, 156.78, 119.77, 102.84, 97.62, -0.35 ppm. The spectroscopic data are in agreement with those reported in the literature.[48]

2-[2-(Trimethylsilyl)ethynyl]pyrazine (6m)

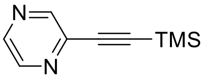

5 mmol scale reaction, synthesized from 2-chloropyrazine, 80 ˚C, reaction went to completion in 5 h, purified by flash column chromatography (SiO_2, 12 g, 40-60 mm ø, 40% EtOAc in PE, ca. 10 mL, R_f = 0.8 P:E 3:2), brown oil, 615 mg, 70% yield. **^{1}H NMR** (600 MHz, $CDCl_3$) *δ* 8.72 – 8.68 (d, *J* = 1.6 Hz, 1H, Ar-*H*), 8.57 – 8.53 (dd, *J* = 2.6, 1.6 Hz, 1H, Ar-*H*), 8.51 – 8.48 (d, *J* = 2.5 Hz, 1H, Ar-*H*), 0.31 (s, 9H, Si*$(CH_3)_3$*) ppm. **^{13}C NMR** (126 MHz, $CDCl_3$) *δ* 147.87, 144.36, 143.00, 139.89, 100.65, 99.89, -0.43 ppm. The spectroscopic data are in agreement with those reported in the literature.[47]

B. General Procedure for the Synthesis of Terminal Alkynes

B1. General procedure for Seyferth-Gilbert Homologation

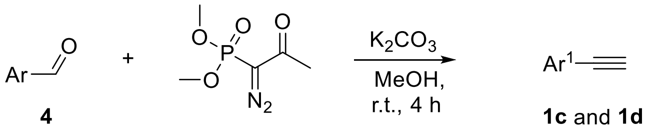

4 **1c** and **1d**
(49 and 57% respectively)

To a solution of aldehyde **4** and anhydrous potassium carbonate (2 eq) in methanol (0.1 M) was added dimethyl (1-diazo-2-oxopropyl)phosphonate (1.2 eq) dropwise. The reaction mixture was stirred at room temperature for 4 h. Reaction progress was monitored by TLC which showed loss of starting material and appearance of a new product spot. The reaction mixture was concentrated *in vacuo* and the resultant residue was redissolved in EtOAc and washed with water (3 × 10 mL). The aqueous portions were combined and extracted with EtOAc (3 × 10 mL). The combined organic layers were washed with NaCl (sat. aq., 1 × 10 mL), dried over anhydrous $MgSO_4$ and concentrated *in vacuo*. The crude product was purified by passing through a short pad of silica and eluting with 0-5% EtOAc in petroleum ether. Fractions with the product were pooled and evaporated to afford the terminal acetylenes **1c** and **1d** in 57% and 49% yields respectively.

4-ethynyl-1,3-thiazole (1c)

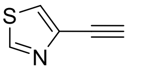

2 mmol scale, synthesized from 1,3-thiazole-4-carbaldehyde, yellow oil, 124 mg, 57%. R_f = 0.65 (P:E 6:1). ^{1}H NMR (500 MHz, $CDCl_3$) *δ* 8.77 (d, *J* = 2.1 Hz, 1H, Ar*H*), 7.58 (d, *J* = 2.1 Hz, 1H, Ar*H*), 3.14 (s, 1H,C*H*) ppm. ^{13}C NMR (126 MHz, $CDCl_3$) *δ* 152.67, 137.65, 123.66, 77.99, 77.45 ppm. The spectroscopic data are in agreement with those reported in the literature.[49]

2-ethynyl-1,3-thiazole (1d)

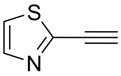

2 mmol scale, synthesized from 1,3-thiazole-2-carbaldehyde, yellow oil, 107 mg, 49%. R_f = 0.68 (P:E 6:1). ^{1}H NMR (500 MHz, $CDCl_3$) δ 7.84 (d, J = 3.3 Hz, 1H, Ar*H*), 7.37 (d, J = 3.3 Hz, 1H, Ar*H*), 3.47 (s, 1H, C*H*) ppm. ^{13}C NMR (126 MHz, $CDCl_3$) δ 147.77, 143.71, 121.20, 82.29, 76.50 ppm. The spectroscopic data are in agreement with those reported in the literature.[49]

B2. General procedure for Protodesilylation of TMS acetylenes*

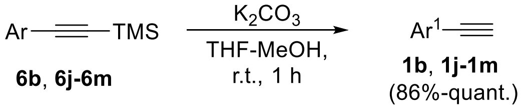

To an oven-dried RBF containing a PTFE-coated magnetic stir-bar, silyl-protected acetylene **6b, 6j-6m** (1 eq), and anhydrous potassium carbonate (0.2 eq), 1:1 MeOH/THF (0.5 M) were added. The reaction mixture was stirred at room temperature for 1 h. Reaction progress was monitored by TLC, which showed loss of starting material and appearance of new major polar spot. Upon completion of the reaction, the mixture was extracted with DCM and saturated aqueous NH_4Cl solution, and dried over anhydrous $MgSO_4$. The filtrate was evacuated *in vacuo* and the residue was flushed through a short silica column to afford the terminal acetylenes **1b**, **1j**, **1l**, **1m** in 86%-quantitative yields.

*TBAF and KF were not tolerated for Protodesilylation of heteroaryl alkynes. Though the reaction went to completion within minutes, degradation was observed upon aqueous work up and evaporation.

Methyl 5-ethynyl-1-methyl-1*H*-pyrrole-2-carboxylate (1b)

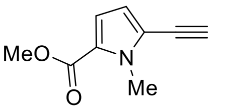

1.3 mmol scale, synthesized from silyl-protected acetylene **6b**, orange waxy solid, 212 mg, quant. yield. R_f = 0.13 (P:E 100:1). **Mp** 87-89 °C. **^{1}H NMR** (600 MHz, $CDCl_3$) δ 6.88 (d, J = 4.2 Hz, 1H, Ar-*H*), 6.45 (d, J = 4.2 Hz, 1H, Ar-*H*), 4.01 (s, 3H, O*CH₃*), 3.85 (s, 3H, N*CH₃*), 3.45 (s, 1H, *CH*) ppm. **^{13}C NMR** (151 MHz, $CDCl_3$) δ 161.09, 123.66, 121.43, 116.81, 114.75, 83.48, 74.72, 34.18 ppm. **HRMS (ESI)**: calcd for $C_9H_{10}N_1O_2$ $[M+H]^+$: 164.0706; found: 164.0714.

2-Ethynylpyrimidine (1j)

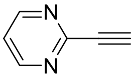

1.6 mmol scale, synthesized from silyl-protected acetylene **6j**, brown waxy solid, 151 mg, 91% yield. R_f = 0.17 (P:E 3:2). **^{1}H NMR** (600 MHz, $CDCl_3$) δ 8.76 (d, J = 5.0 Hz, 2H, Ar-*H*), 7.31 (t, J = 5.0 Hz, 1H, Ar-*H*), 3.16 (s, 1H, *CH*) ppm.**^{13}C NMR** (126 MHz, $CDCl_3$) δ 157.31, 152.26, 120.50, 81.79, 75.88. The spectroscopic data are in agreement with those reported in the literature.[50]

5-Ethynylpyrimidine (1l)

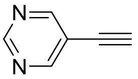

2 mmol scale, synthesized from silyl-protected acetylene **6l**, brown waxy solid, 207 mg, quant. yield. R_f = 0.61 (P:E 3:1). **^{1}H NMR** (500 MHz, $CDCl_3$) δ 9.19 (s, 1H, Ar-*H*), 8.85 (s, 2H, Ar-*H*), 3.42 (s, 1H, *CH*) ppm. **^{13}C NMR** (126 MHz, $CDCl_3$) δ 159.35, 157.27, 118.82, 84.45, 76.90 ppm. The spectroscopic data are in agreement with those reported in the literature.[51]

2-Ethynylpyrazine (1m)

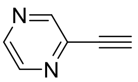

2 mmol scale, synthesized from silyl-protected acetylene **6m**, brown waxy solid, 179 mg, 86% yield. R_f = 0.4 (P:E 3:2). **^{1}H NMR** (500 MHz, $CDCl_3$) δ 8.74 (s, 1H, Ar-*H*), 8.60 – 8.55 (m, 2H, Ar-*H*), 3.37 (s, 1H, *CH*) ppm.**^{13}C NMR** (126 MHz, $CDCl_3$) δ 148.09, 144.49, 143.63, 139.30, 81.18, 80.01 ppm. The spectroscopic data are in agreement with those reported in the literature.[52]

C. General Procedure for iodination/iododesilylation*:

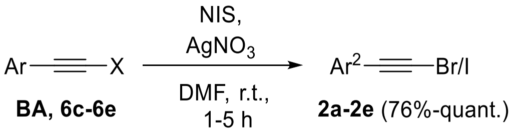

To a solution of terminal acetylene **BA** or silyl-protected acetylene **6c-6e** (1 eq) in dry DMF (1 M) in an oven-dried round bottom flask (RBF) with a PTFE-coated magnetic stir-bar, $AgNO_3$ (5-50 mol%) was added and cooled to 0 °C. NXS (1.2-2.5 eq) was then added, and the reaction was stirred at room temperature for 1-3 h. Reaction progress was monitored by TLC which showed loss of starting material and appearance of new major spot. After completion, the reaction mixture was diluted with water (10× volume of DMF) and filtered. The residue was re-dissolved in DCM, washed with saturated sodium thiosulfate, dried over anhydrous $MgSO_4$, filtered and evacuated *in vacuo* to provide haloacetylenes **2a-2e** in 76%-quantitative yields. The intermediates were taken forward without further purification.

*Bromodesilylation of the heteroaryl alkynes resulted in oily residues that were unstable upon aqueous work-up and subsequent evaporation of the organic fraction *in vacuo*. The residues had multiple UV-active products as observed on TLC. Alternatively, iodination afforded stable solids that precipitated upon aqueous work-up, and were stable after evaporation of solvent under reduced pressure.

Methyl 4-(2-bromoethynyl)benzoate (2a)

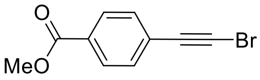

2 mmol scale, synthesized from commercially available methyl benzoate acetylene **BA**, 10 mol% $AgNO_3$, 1.2 eq NBS, reaction went to completion in 1 h, white solid, 414 mg, 87% yield. R_f = 0.66 (P:E 10:1). **^{1}H NMR** (600 MHz, $CDCl_3$) δ 8.01 (d, J = 8.1 Hz, 2H, Ar-*H*), 7.53 (d, J = 8.1 Hz, 2H, Ar-*H*), 3.94 (s, 3H, O*CH*$_3$) ppm. **^{13}C NMR (150 MHz, $CDCl_3$)** δ 166.44, 131.98, 130.01, 129.51, 127.34, 79.43, 52.28 ppm. The spectroscopic data are in agreement with those reported in the literature.[53]

Methyl 4-(2-iodoethynyl)benzoate (2b)

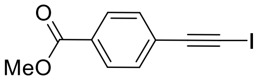

5 mmol scale, synthesized from commercially available methyl benzoate acetylene **BA**, 5 mol% $AgNO_3$, 1.2 eq NIS, reaction went to completion in 1 h, white solid, 1280 mg, 89% yield. R_f = 0.7 (P:E 30:1). **^{1}H NMR** (500 MHz, $CDCl_3$) δ 8.01-8.00 (d, J = 8.7 Hz, 2H, Ar-*H*), 7.52-7.5 (d, J = 8.7 Hz, 2H, Ar-*H*), 3.94 (s, 3H, O*CH*$_3$).ppm. **^{13}C NMR** (126 MHz, $CDCl_3$) δ 166.39, 132.29, 130.05, 129.41, 127.90, 93.47, 52.28. The spectroscopic data are in agreement with those reported in the literature.[54]

Methyl 6-(2-iodoethynyl)pyridine-3-carboxylate (2c)

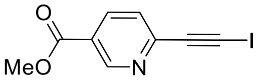

4.6 mmol scale, synthesized from silyl-protected acetylene **6c**, 15 mol% $AgNO_3$, 2.5 eq NIS, reaction went to completion in 1 h, brown crystals, 978 mg, 76% yield. R_f = 0.53 (P:E 20:1). **Mp** 171-173 °C. **^{1}H NMR** (600 MHz, $CDCl_3$) δ 9.18 (dd, J = 2.2, 0.9 Hz, 1H, Ar-*H*), 8.29 (dd, J = 8.1, 2.2 Hz, 1H, Ar-*H*), 7.51 (dd, J = 8.1, 0.9 Hz, 1H, Ar-*H*), 3.98 (s, 3H, O*CH*$_3$) ppm. **^{13}C NMR** (151 MHz, $CDCl_3$) δ 165.16, 150.99, 146.48, 137.22, 127.13, 125.18, 93.62, 52.57 ppm. **HRMS (ESI)**: calcd for $C_9H_7INO_2$ $[M+H]^+$: 287.9477; found 287.9516.

Methyl 2-(2-iodoethynyl)pyrimidine-5-carboxylate (2d)

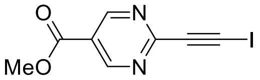

1 mmol scale, synthesized from silyl-protected acetylene **6d**, 50 mol% $AgNO_3$, 2.5 eq NIS, reaction went to completion in 5 h, brown crystals, 194 mg, 68%. R_f = 0.2 (P:E 4:1). **Mp** 191-193 °C. **^{1}H NMR** (600 MHz, $CDCl_3$) δ 9.25 (s, 2H, Ar-*H*), 4.01 (s, 3H, O*CH*$_3$) ppm. **^{13}C NMR** (151 MHz, $CDCl_3$) δ 163.61, 158.28, 154.53, 122.61, 93.41, 52.87 ppm. **HRMS (ESI)**: calcd for $C_8H_6IN_2O_2$ $[M+H]^+$: 288.9468; found 288.9475.

Ethyl 2-(2-iodoethynyl)pyrimidine-5-carboxylate (2d-Et)

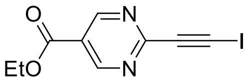

14 mmol scale, synthesized from silyl-protected acetylene **6d-Et**, 50 mol% $AgNO_3$, 2.5 eq NIS, reaction went to completion in 5 h, yellow crystals, 2900 mg, 72%. R_f = 0.71 (P:E 4:1). **Mp** 180-183 °C. **^{1}H NMR** (600 MHz, $CDCl_3$) δ 9.25 (s, 2H, Ar-*H*), 4.48 (q, J = 7.1 Hz, 2H, O*CH*$_2$), 1.45 (t, J = 7.1 Hz, 3H, CH_2*CH*$_3$) ppm. **^{13}C NMR** (151 MHz, $CDCl_3$) δ 163.15, 158.26, 154.45, 122.88, 93.43, 62.15, 14.22 ppm. **HRMS (ESI)**: calcd for $C_9H_8INO_2$ $[M+H]^+$: 302.96250; found 302.9639.

Methyl 5-(2-iodoethynyl)pyrazine-2-carboxylate (2e)

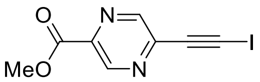

2.6 mmol scale, synthesized from silyl-protected acetylene **6e**, 15 mol% $AgNO_3$, 2.5 eq NIS, reaction went to completion in 12 h, yellow crystals, 693 mg, 92%. R_f = 0.3 (P:E 2:1). **Mp** 202 °C. **^{1}H NMR** (400 MHz, $CDCl_3$) δ 9.28 (d, J = 1.5 Hz, 1H, Ar-*H*), 8.77 (d, J = 1.5 Hz, 1H, Ar-*H*), 4.07 (s, 3H, O*CH*$_3$) ppm. **^{13}C NMR** (151 MHz, $CDCl_3$) δ 163.87, 147.56, 145.58, 142.36, 141.01, 91.20, 53.28 ppm. **HRMS (ESI)**: calcd for $C_8H_6IN_2O_2$$[M+H]^+$: 288.9429; found 288.9468.

D. BADY and Het-DY tag synthesis

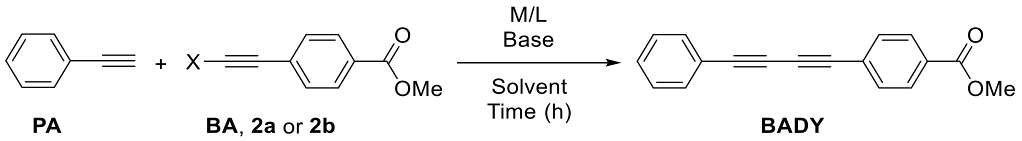

PA **BA**, **2a** or **2b** **BADY**

Table S2. Screening of catalytic conditions for efficient heterocoupling of alkyne coupling partners. [a] Synthesized via general procedure D1. [b] Synthesized via general procedure D2.

Compd. Name	X	Ratio of coupling partners	Scale	M	L	Solvent	Base	Time (h)	% Yield
**BA**	H	1:5	1 mmol	Cu	DMAP	ACN	N/A	20	60%[a]
**2a**	Br	1:1.2		Pd(0)	TPP	DMF	TEA	4	35%[b]
**2a**	Br	1:1.2		Pd(0)	ToTP	DMF	TEA	4	38%[b]
**2a**	Br	1:1.2		Pd(0)	Lei Ligand	DMF	TEA	4	60%[b]
**2b**	I	1.2:1		Pd(0)	Lei Ligand	DMF	TEA	4	70%[b]
**2b**	I	1.2:1		Pd(0)	(t-Bu)$_3$P	DMF	TEA	4	19%[b]

D1. General procedure for Glaser cross-coupling

Commercially available terminal acetylene **PA** or **1a** (5 eq), commercially available terminal acetylene ester **BA**, or terminal acetylene **1b** synthesized from general procedure B2 (1 eq), CuI (10 mol%) and DMAP (20 mol%) were added to acetonitrile (1 M) in an oven-dried RBF with PTFE-coated magnetic stir-bar, at room temperature under open atmosphere. The reaction mixture was stirred at room temperature for 20 h. Reaction progress was monitored by TLC, which showed loss of starting material and appearance of three new spots. Upon completion, minimum amount of MeOH and Celite (3× weight of the crude) were added and the solution was evaporated to afford a plug. The resulting plug was loaded on to an automatic flash column for purification. Fractions with desired R_f (TLC) were pooled and evaporated to afford the heterocoupled products **BADY** and **3a**.

D2. General procedure for Pd-catalyzed Cadiot-Chodkievicz (CC) cross-coupling

To an oven-dried 2-necked RBF with an oven-dried PTFE-coated magnetic stir-bar, $Pd_2(dba)_3$ (4 mol%), ligand (4 mol%), and CuI (2 mol%) were added. Anhydrous DMF (2 M) was added via a syringe and the mixture vacuum purged with nitrogen for three cycles. After stirring the mixture under nitrogen for 10 min, a vacuum purged and N_2-filled solution of commercially available terminal acetylenes **PA** or **1a** or terminal acetylenes **1c**, **1d**, **1j**, **1l**, **1m** (1.2 eq) synthesized from general procedure B2, in anhydrous DMF (1 M) was added via a syringe, followed by TEA (2 eq). The reaction mixture was stirred for another 5 min, then a vacuum purged and N_2-filled solution of haloacetylene **2a**-**2e** in anhydrous DMF (1 M) was added last via a syringe. The system was stirred at room temperature under N_2 for 4 h. Reaction progress was monitored by TLC, which showed loss of starting material and appearance of three new spots. Upon completion, a minimum amount of MeOH and Celite (3× weight of the crude) were added and the solution was evaporated to afford a plug. The resulting plug was loaded on to an automatic flash column for purification. Fractions with desired R_f (TLC) were pooled and evaporated to afford the heterocoupled products **BADY, 3b-n**.

Methyl 4-(4-phenylbuta-1,3-diyn-1-yl)benzoate (BADY)

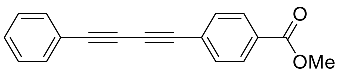

1 mmol scale (of the limiting reagent alkyne), synthesized via $Pd(dba)_3$-(t-Bu)$_3$P catalyzed general procedure D2 from commercially available terminal acetylene **PA** and iodoacetylene **2b**, reaction went to completion in 4 h, purified by flash column chromatography (SiO_2, 4 g, 40-60 mm ø, 10 % EtOAc in PE, R_f = 0.6 (P:E 4:1)). Fractions with the desired R_f (TLC) were pooled and evaporated to dryness to afford **BADY** as a buff coloured solid. 182 mg, 70%. **^{1}H NMR** (500 MHz, $CDCl_3$,) δ 8.05 – 8.01 (m, 2H, Ar-*H*), 7.63 – 7.59 (m, 2H, Ar-*H*), 7.58 – 7.55 (m, 2H, Ar-*H*), 7.44 – 7.35 (m, 3H, Ar-*H*), 3.99 – 3.93 (s, 3H, OCH_3) ppm. **^{13}C NMR** (151 MHz, $CDCl_3$) δ 166.35, 132.60, 132.41, 130.33, 129.57, 129.52, 128.52, 126.53, 121.50, 83.05, 80.49, 73.58, 52.33 ppm. The spectroscopic data are in agreement with those reported in the literature.[55]

Methyl 1-methyl-5-[4-(1-methyl-1H-imidazol-5-yl)buta-1,3-diyn-1-yl]-1H-pyrrole-2-carboxylate (3a)

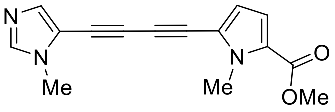

1 mmol scale (of the limiting reagent alkyne), synthesized via general procedure D1 from commercially available 1-methyl-1*H*-imidazole acetylene **1a** and terminal acetylene **1b**, purified by flash column chromatography (SiO_2, 12 g, 40-60 mm ø, 5% MeOH in DCM, ca. 10 mL fractions), buff coloured solid, 105 mg, 50%. R_f = 0.53 (D:M 40:1). **Mp** 149-151 °C. **^{1}H NMR** (600 MHz, $CDCl_3$) δ 8.36 (br s, 1H, Ar-*H*), 7.54 (br s, 1H, Ar-*H*), 6.91 – 6.88 (d, J = 4.2 Hz, 1H, Ar-*H*), 6.61 – 6.58 (d, J = 4.2 Hz, 1H, Ar-*H*), 4.03 (s, 3H, NCH_3), 3.89 (s, 3H, NCH_3), 3.87 (s, 3H, OCH_3) ppm. **^{13}C NMR** (151 MHz, $CDCl_3$) δ 160.80, 125.16, 120.31, 117.55, 117.10, 82.45, 78.98, 75.92, 51.54, 34.56 ppm. **HRMS (ESI) (*m/z*)**: calc'd for $C_{15}H_{13}N_3O_2$ [M+H]$^+$: 268.1080; found 268.1080.

Methyl 4-[4-(1-methyl-1*H*-imidazol-5-yl)buta-1,3-diyn-1-yl]benzoate (3b)

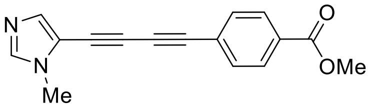

2 mmol scale (of the limiting reagent alkyne), synthesized via $Pd(dba)_3$-(t-Bu)$_3$P catalyzed general procedure D2 from commercially available 1-methyl-1*H*-imidazole acetylene **1a** and iodoacetylene **2b**, reaction went to completion in 5 h, purified by flash column chromatography (SiO_2, 4 g, 40-60 mm ø, 30 % MeOH in DCM, ca. 10 mL, R_f = 0.33 (P:E 30:1), buff coloured solid, 100 mg, 19% yield. **Mp** 148-150°C. **^{1}H NMR** (600 MHz, DMSO-d_6) δ 8.01 – 7.98 (d, J = 8.7 Hz, 2H, Ar-*H*), 7.86 (s, 1H, Ar-*H*), 7.78 – 7.74 (d, J = 8.7 Hz, 2H, Ar-*H*), 7.55 (s, 1H, Ar-*H*), 3.88 (s, 3H, OCH_3), 3.70 (s, 3H, NCH_3) ppm. **^{13}C NMR** (151 MHz, DMSO-d_6) δ 165.92, 133.11, 130.83, 129.94, 125.48, 88.46, 83.83, 80.79, 76.29, 52.93, 32.47 ppm. **HRMS (ESI) (*m/z*)**: calc'd for $C_{16}H_{12}N_2O_2$[M+H]$^+$: 265.0971; found 265.0983.

Methyl 4-[4-(thiazo-4-yl)buta-1,3-diyn-1-yl]benzoate (3c)

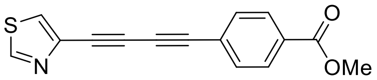

0.5 mmol scale (of the limiting reagent bromoalkyne), synthesized via $Pd(dba)_3$-Lei ligand catalyzed general procedure D2 from terminal acetylene **1b** and iodoacetylene **2b**, reaction went to completion in 4 h, purified by flash column chromatography (SiO_2, 24 g, 20-40 mm ø, 10 %, EtOAc in PE, ca. 10 mL, R_f = 0.24 (P:E 9:1), brown solid, 64.2 mg, 48% yield. **Mp** 162–164 °C. **^{1}H NMR** (500 MHz, $CDCl_3$) δ 8.80 (d, J = 2.0 Hz, 1H, Ar*H*), 8.02 – 8.01 (m, 2H, Ar*H*), 7.69 (d, J = 2.0 Hz, 1H, Ar*H*), 7.60 – 7.59 (m, 2H, Ar*H*), 3.93 (s, 3H, C*H*$_3$) ppm. **^{13}C NMR** (151 MHz, $CDCl_3$) δ 166.42, 152.99, 137.41, 132.69, 130.73, 129.71, 126.19, 125.43, 81.21, 76.37, 76.35, 74.13, 52.50 ppm. **HRMS (ESI) (*m/z*)**: calc'd for $C_{15}H_9NO_2SNa$ [M+Na]$^+$: 290.0246; found 290.0242.

Methyl 4-[4-(thiazo-2-yl)buta-1,3-diyn-1-yl]benzoate (3d)

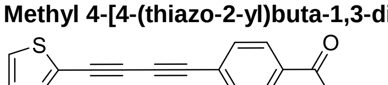

0.5 mmol scale (of the limiting reagent bromoalkyne), synthesized via $Pd(dba)_3$-Lei ligand catalyzed general procedure D2 from terminal acetylene **1c** and iodoacetylene **2b**, reaction went to completion in 4 h, purified by flash column chromatography (SiO_2, 12 g, 20-40 mm ø, 10 %, EtOAc in PE, ca. 10 mL, R_f = 0.32 (P:E 9:1), brown solid, 56.2 mg, 42% yield. **Mp** 122–125 °C. **^{1}H NMR** (500 MHz, $CDCl_3$) δ

8.04 – 8.02 (m, 2H, Ar*H*), 7.89 (d, *J* = 3.3 Hz, 1H, Ar*H*), 7.62 – 7.60 (m, 2H, Ar*H*), 7.43 (d, *J* = 3.3 Hz, 1H, Ar*H*), 3.94 (s, 3H, C*H*$_3$) ppm. **^{13}C NMR** (126 MHz, $CDCl_3$) δ 166.33, 147.46, 144.32, 132.78, 131.11, 129.76, 125.64, 122.11, 84.00, 78.37, 75.73, 74.82, 52.55 ppm. **HRMS (ESI) (*m/z*)**: calc'd for $C_{15}H_9NO_2SNa$ [M+Na]$^+$: 290.0246; found 290.0248.

Methyl 4-[4-(pyridin-2-yl)buta-1,3-diyn-1-yl]benzoate (3e)

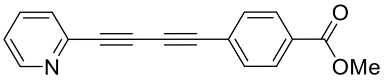

1 mmol-scale (of the limiting reagent iodoalkyne), synthesized via Pd(dba)$_3$-Lei ligand catalyzed general procedure D2 from commercially available 2-pyridyl acetylene **1e** and iodoacetylene **2b**, reaction went to completion in 5 h, purified by flash column chromatography (SiO_2, 4 g, 40-60 mm ø, 15 % EtOAc in PE, ca. 10 mL, R_f = 0.33 P:E 5:1), buff coloured solid, 125 mg, 48% yield. **Mp** 123-125 °C. **^{1}H NMR** (500 MHz, $CDCl_3$) *δ* 8.66 (d, *J* = 4.7 Hz, 1H, Ar-*H*), 8.04 (d, *J* = 8.7 Hz, 2H, Ar-*H*), 7.73 (td, *J* = 7.7, 1.8 Hz, 1H, Ar-*H*), 7.62 (d, *J* = 8.7 Hz, 2H, Ar-*H*), 7.57 (dt, *J* = 7.7, 1.1 Hz, 1H, Ar-*H*), 7.33 (ddd, *J* = 7.7, 4.7, 1.1 Hz, 1H, Ar-*H*), 3.96 (s, 3H, Ar-*H*) ppm. **^{13}C NMR** (151 MHz, $CDCl_3$) *δ* 166.27, 150.45, 142.08, 136.19, 132.60, 130.65, 129.57, 128.21, 126.00, 123.70, 81.53, 81.30, 76.20, 73.28, 52.36 ppm. **HRMS (ESI) (*m/z*)**: calc'd for $C_{17}H_{11}NO_2$ [M+Na]$^+$: 284.0682; found 284.0678.

Methyl 4-[4-(pyridin-3-yl)buta-1,3-diyn-1-yl]benzoate (3f)

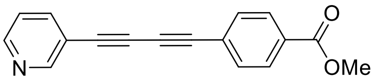

1 mmol scale (of the limiting reagent iodoalkyne), synthesized via Pd(dba)$_3$-Lei ligand catalyzed general procedure D2 from commercially available 3-pyridyl acetylene **1f** and iodoacetylene **2b**, reaction went to completion in 5 h, purified by flash column chromatography (SiO_2, 4 g, 40-60 mm ø, 15 %, EtOAc in PE, ca. 10 mL, R_f = 0.33 P:E 5:1), buff coloured solid, 175 mg, 67% yield. **Mp** 178-180 °C. **^{1}H NMR** (500 MHz, $CDCl_3$) *δ* 8.82 – 8.77 (m, 1H, Ar-*H*), 8.62 (dd, *J* = 4.9, 1.7 Hz, 1H, Ar-*H*), 8.04 (d, *J* = 8.6 Hz, 2H, Ar-*H*), 7.84 (dt, *J* = 7.9, 1.7 Hz, 1H, Ar-*H*), 7.62 (d, *J* = 8.6 Hz, 2H, Ar-*H*), 7.32 (dd, *J* = 7.9, 4.9 Hz, 1H, Ar-*H*), 3.96 (s, 3H, O*CH$_3$*) ppm. **^{13}C NMR** (126 MHz, $CDCl_3$) *δ* 166.27, 153.18, 149.48, 139.41, 132.51, 130.65, 129.61, 126.02, 123.11, 119.00, 81.65, 79.38, 76.06, 52.38 ppm. **HRMS (ESI) (*m/z*)**: calc'd for $C_{17}H_{11}NO_2$ [M+Na]$^+$: 284.0682; found 284.0689.

Methyl 4-[4-(pyridin-4-yl)buta-1,3-diyn-1-yl]benzoate (3g)

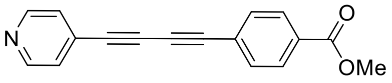

1 mmol scale (of the limiting reagent iodoalkyne), synthesized via Pd(dba)$_3$-Lei ligand catalyzed general procedure D2 from commercially available 4-pyridyl acetylene **1g** and iodoacetylene **2b**, reaction went to completion in 5 h, purified by flash column chromatography (SiO_2, 4 g, 40-60 mm ø, 20%, EtOAc in PE, ca. 10 mL, R_f = 0.33 P:E 5:1), buff coloured solid, 183 mg, 70% yield. **Mp** 184-186 °C. **^{1}H NMR** (500 MHz, $CDCl_3$) *δ* 8.66 (d, *J* = 6.1 Hz, 2H, Ar-*H*), 8.05 (d, *J* = 8.0 Hz, 2H, Ar-*H*), 7.63 (d, *J* = 8.0 Hz, 2H, Ar-*H*), 7.40 (d, *J* = 6.1 Hz, 2H, Ar-*H*), 3.96 (s, 3H, O*CH$_3$*) ppm. **^{13}C NMR** (126 MHz, $CDCl_3$) *δ* 166.20, 149.94, 132.57, 130.85, 129.78, 129.62, 126.05, 125.73, 82.54, 79.57, 77.77, 75.71, 52.39 ppm. **HRMS (ESI) (*m/z*)**: calc'd for $C_{17}H_{11}NO_2$ [M+Na]$^+$: 284.0682; found 284.0667.

Methyl 6-(4-phenylbuta-1,3-diyn-1-yl)pyridine-3-carboxylate (3h)

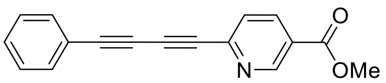

1 mmol scale (of the limiting reagent iodoalkyne), synthesized via Pd(dba)$_3$-Lei ligand catalyzed general procedure D2 from commercially available phenyl acetylene **PA** and iodoacetylene **2c**, reaction went to completion in 5 h, purified by flash column chromatography (SiO_2, 4 g, 40-60 mm ø, 5%, EtOAc in PE, ca. 10 mL, R_f = 0.28 P:E 20:1), buff coloured solid, 108 mg, 42% yield. **Mp** 131-133 °C. **^{1}H NMR** (500 MHz, $CDCl_3$) *δ* 9.22 (s, 1H, Ar-*H*), 8.30 (dd, *J* = 8.1, 2.1 Hz, 1H, Ar-*H*), 7.64 – 7.55 (m, 3H, Ar-*H*), 7.48 – 7.35 (m, 3H, Ar-*H*), 3.99 (s, 3H, O*CH$_3$*) ppm. **^{13}C NMR** (126 MHz, $CDCl_3$) *δ* 165.08, 151.15, 145.75, 137.32, 132.80, 129.89, 128.55, 127.55, 125.18, 121.00, 84.29, 79.41, 73.26, 52.62 ppm. **HRMS (ESI) (*m/z*)**: calc'd for $C_{17}H_{11}NO_2$ [M+H]$^+$: 262.0862; found 262.0866.

Methyl 6-[4-(pyridin-2-yl)buta-1,3-diyn-1-yl]pyridine-3-carboxylate (3i)

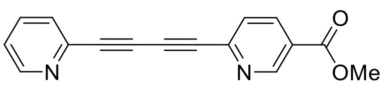

2 mmol scale (of the limiting reagent iodoalkyne), synthesized via Pd(dba)$_3$-Lei ligand catalyzed general procedure D2 from commercially available 2-pyridyl acetylene **xx** and iodoacetylene **2c**, reaction went to completion in 5 h. Due to similar polarities, the heterocoupled product was difficult to separate from the homocoupled product of the terminal alkyne **3i_AA**, 270 mg (1:0.25 ratio of **3i** and **3i_AA** calculated from NMR), 52% yield. The impure product was taken forward without further purification. **3i**: **^{1}H NMR** (600 MHz, $CDCl_3$) *δ* 9.23 (d, *J* = 2.2 Hz, 1H, Ar-*H*), 8.68 – 8.63 (m, 1H, Ar-*H*), 8.31 (dd, *J* = 8.1, 2.2 Hz, 1H, Ar-*H*), 7.72 (td, *J* = 7.8, 1.8 Hz, 1H, Ar-*H*), 7.64 (dd, *J* = 8.1, 0.9 Hz, 1H, Ar-*H*), 7.58 (dt, *J* = 7.8, 1.1 Hz, 1H, Ar-*H*), 7.34 (ddd, *J* = 7.7, 4.8, 1.2 Hz, 1H, Ar-*H*), 3.99 (s, 3H, O*CH$_3$*) ppm. **^{13}C NMR** (151 MHz, $CDCl_3$) *δ* 165.07, 151.34, 150.51, 145.48, 141.69, 137.23, 136.24, 128.54, 128.42, 125.43, 124.01, 82.35, 80.18, 75.89, 72.77, 52.63 ppm. **HRMS (ESI)**: calc'd for $C_{16}H_{10}N_2O_2$ [M+Na]$^+$: 285.0634; found 285.0635.

Side-product: 2-[4-(pyridin-2-yl)buta-1,3-diyn-1-yl]pyridine (3i_AA)

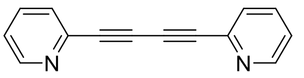

^{1}H NMR (600 MHz, $CDCl_3$) *δ* 8.68 – 8.63 (m, 2H), 7.76 – 7.70 (m, 2H), 7.61 – 7.56 (m, 2H), 7.37 – 7.31 (m, 2H) ppm. **^{13}C NMR** (151 MHz, $CDCl_3$) *δ* 150.22, 141.56, 136.37, 128.51, 123.85, 80.82, 73.50 ppm. **HRMS (ESI)**: calcd for $C_{14}H_8N_2$ [M+Na]$^+$: 227.0579; found 227.0583.

Methyl 6-[4-(pyrimidin-2-yl)buta-1,3-diyn-1-yl]pyridine-3-carboxylate (3j)

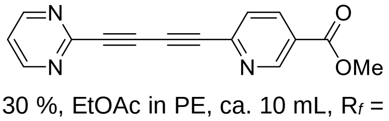

1 mmol scale (of the limiting reagent iodoalkyne), synthesized via Pd(dba)$_3$-Lei ligand catalyzed general procedure D2 from terminal acetylene **1d** and iodoacetylene **2c**, reaction went to completion in 5 h, purified by flash column chromatography (SiO_2, 4 g, 40-60 mm ø, 30 %, EtOAc in PE, ca. 10 mL, R_f = 0.1 P:E 4:1), light brown solid, 46 mg, 17% yield. **Mp** 170-172°C. **^{1}H NMR** (500 MHz, $CDCl_3$) *δ*

9.24 (s, 1H, Ar-*H*), 8.79 (d, *J* = 5.0 Hz, 2H, Ar-*H*), 8.33 (dd, *J* = 8.2, 2.2 Hz, 1H, Ar-*H*), 7.67 (d, *J* = 8.2 Hz, 1H, Ar-*H*), 7.34 (t, *J* = 5.0 Hz, 1H, Ar-*H*), 4.00 (s, 3H, O*CH₃*) ppm. **^{13}C NMR** (126 MHz, $CDCl_3$) *δ* 164.97, 157.42, 152.01, 151.32, 145.02, 137.31, 128.12, 125.72, 120.75, 80.98, 80.28, 75.41, 71.03, 52.68 ppm. **HRMS (ESI) (*m/z*)**: calc'd for $C_{15}H_9N_3O_2$ [M+H]$^+$: 264.0767; found 265.0772.

Methyl 2-[4-(pyrimidin-2-yl)buta-1,3-diyn-1-yl]pyrimidine-5-carboxylate (3k)*

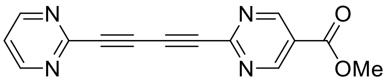

2.8 mmol scale (of the limiting reagent iodoalkyne), synthesized via $Pd(dba)_3$-(*t*-Bu)$_3$P catalyzed general procedure D2 from terminal acetylene **1d** and iodoacetylene **2d**, reaction went to completion in 5 h. Due to similar polarities, the heterocoupled product **3k** was difficult to separate from **3k_EAA**, the homocoupled product of **2d**. Light brown solid, 90 mg (1:0.2 ratio of **3k** and **3k_EAA** calculated from NMR), 12% yield. **^{1}H NMR** (500 MHz, $CDCl_3$) *δ* 9.28 (s, 2H, Ar-*H*), 8.80 (d, *J* = 4.9 Hz, 2H, Ar-*H*), 7.36 (t, *J* = 4.9 Hz, 1H, Ar-*H*), 4.03 (s, 3H, O*CH₃*) ppm. **^{13}C NMR** (126 MHz, $CDCl_3$) *δ* 163.49, 158.35, 157.45, 154.17, 151.76, 122.90, 120.98, 81.16, 79.24, 73.73, 70.51, 52.96 ppm. **HRMS (ESI) (*m/z*)**: calc'd for $C_{14}H_9N_4O_2$ [M+H]$^+$: 265.0720; found 265.0728.

* To overcome the similar polarity of homo- and heterodimers for purification, ethyl ester **3k-Et** was also synthesized and taken forward for synthesis of **7k**.

Side-product: 2-[4-(pyrimidin-2-yl)buta-1,3-diyn-1-yl]pyrimidine (3k_AA)

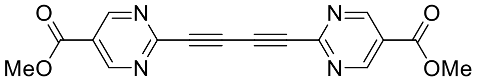

^{1}H NMR (600 MHz, $CDCl_3$) *δ* 9.23 (s, 4H, Ar-*H*), 4.03 (s, 3H, O*CH₃*) ppm. **^{13}C NMR** (151 MHz, $CDCl_3$) *δ* 163.49, 159.16, 158.37, 158.04, 53.37 ppm. **HRMS (ESI)**: calcd for $C_{16}H_{11}N_4O_4$ [M+Na]$^+$: 323.07748; found 323.0770.

Ethyl 2-[4-(pyrimidin-2-yl)buta-1,3-diyn-1-yl]pyrimidine-5-carboxylate (3k-Et)

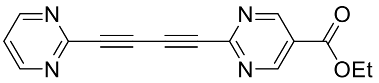

5 mmol scale (of the limiting reagent iodoalkyne), synthesized via $Pd(dba)_3$-(*t*-Bu)$_3$P catalyzed general procedure D2 from terminal acetylene **1d** and iodoacetylene **2e**, reaction went to completion in 5 h, purified by flash column chromatography (SiO_2, 4 g, 40-60 mm ø, 30 %, EtOAc in PE, ca. 10 mL, R_f = 0.25 P:E 1:1), light brown crystals (recrystallized from DCM), 140 mg, 10% yield. **Mp** 124-126 °C. **^{1}H NMR** (500 MHz, $CDCl_3$) *δ* 9.28 (s, 2H, Ar-*H*), 8.80 (d, *J* = 4.9 Hz, 2H, Ar-*H*), 7.35 (t, *J* = 4.9 Hz, 1H, Ar-*H*), 4.49 (q, *J* = 7.2 Hz, 2H, O*CH₂*), 1.46 (t, *J* = 7.2 Hz, 3H, CH_2*CH₃*) ppm. **^{13}C NMR** (126 MHz, $CDCl_3$) *δ* 163.03, 158.33, 157.45, 154.08, 151.77, 123.17, 120.97, 81.10, 79.29, 73.62, 70.53, 62.26, 14.22 ppm. **HRMS (ESI) (*m/z*)**: calc'd for $C_{15}H_{11}N_4O_2$ [M+H]$^+$: 279.0876; found 279.0882.

Methyl 5-[4-(pyrimidin-5-yl)buta-1,3-diyn-1-yl]pyrazine-2-carboxylate (3l)

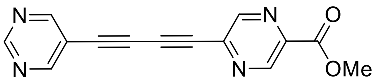

1 mmol scale (of the limiting reagent iodoalkyne), synthesized via $Pd(dba)_3$-Lei ligand catalyzed general procedure D2 from terminal acetylene **1e** and iodoacetylene **2f**, reaction went to completion in 5 h. Due to similar polarities, the heterocoupled product was difficult to separate from the homocoupled product of the terminal alkyne **3l_AA**, 83 mg (1:1.11 ratio of **3l** and **3l_AA** calculated from NMR), 31% yield. **3l**: **^{1}H NMR** (600 MHz, $CDCl_3$) *δ* 9.33 (d, *J* = 1.5 Hz, 1H, Ar-*H*), 9.26 (s, 1H, Ar-*H*), 8.93 (s, 2H, Ar-*H*), 8.88 (d, *J* = 1.5 Hz, 1H, Ar-*H*), 4.09 (s, 3H, O*CH₃*) ppm. **^{13}C NMR** (151 MHz, $CDCl_3$) *δ* 159.89, 159.71, 157.72, 157.49, 147.77, 145.97, 118.25, 79.42, 78.89, 78.09, 53.39 ppm. **HRMS (ESI)**: calcd for $C_{14}H_9N_4O_2$ [M+H]$^+$: 265.0681; found 265.0720.

Side-product: 5-[4-(pyrimidin-5-yl)buta-1,3-diyn-1-yl]pyrimidine (3l_AA)

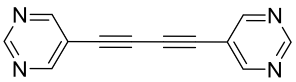

^{1}H NMR (600 MHz, $CDCl_3$) *δ* 9.24 (s, 2H), 8.91 (s, 4H) ppm. **^{13}C NMR** (151 MHz, $CDCl_3$) *δ* 159.7, 157.48, 79.58 ppm. **HRMS (ESI)**: calcd for $C_{12}H_7N_4$ [M+H]$^+$: 207.06652; found 207.0671.

Methyl 6-[4-(pyrazin-2-yl)buta-1,3-diyn-1-yl]pyridine-3-carboxylate (3m)

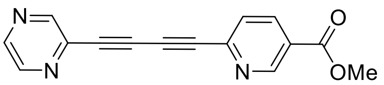

1 mmol scale (of the limiting reagent iodoalkyne), synthesized via $Pd(dba)_3$-Lei ligand catalyzed general procedure D2 from terminal acetylene **1f** and iodoacetylene **2c**, reaction went to completion in 5 h, purified by flash column chromatography (SiO_2, 4 g, 40-60 mm ø, 30 % EtOAc in PE, ca. 10 mL, R_f = 0.25 P:E 2:1), buff coloured solid, 40 mg pure product isolated, 15% yield. **Mp** 171-173°C. **^{1}H NMR** (500 MHz, $CDCl_3$) *δ* 1H NMR (500 MHz, $CDCl_3$) δ 9.24 (dd, *J* = 2.2, 0.9 Hz, 1H, Ar-*H*), 8.80 (d, *J* = 1.6 Hz, 1H, Ar-*H*), 8.63 (dd, *J* = 2.5, 1.6 Hz, 1H, Ar-*H*), 8.58 (d, *J* = 2.5 Hz, 1H, Ar-*H*), 8.33 (dd, *J* = 8.2, 2.2 Hz, 1H, Ar-*H*), 7.68 – 7.64 (dd, *J* = 8.2, 0.9 Hz, 1H, Ar-*H*), 4.00 (s, 3H, O*CH₃*) ppm. **^{13}C NMR** (126 MHz, $CDCl_3$) *δ* 164.97, 151.41, 148.82, 145.03, 144.82, 143.87, 138.82, 137.31, 127.94, 125.73, 81.52, 79.28, 76.55, 75.08, 52.69 ppm. **HRMS (ESI) (*m/z*)**: calc'd for $C_{15}H_9N_3O_2$ [M+H]$^+$: 264.0767; found 264.0772.

Methyl 2-[4-(pyrazin-2-yl)buta-1,3-diyn-1-yl]pyrimidine-5-carboxylate (3n)

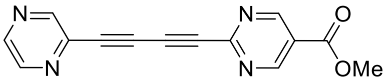

0.3 mmol scale (of the limiting reagent iodoalkyne), synthesized via $Pd(dba)_3$-Lei ligand catalyzed general procedure D2 from terminal acetylene **1f** and iodoacetylene **2d**, reaction went to completion in 5 h. Due to similar polarities, the heterocoupled product was difficult to separate from the homocoupled product of the terminal alkyne **3m_AA**, 20 mg (1:0.4 ratio of **3m** and **3m_AA** calculated from NMR) 14%. ^{1}H NMR (600 MHz, $CDCl_3$) *δ* 9.25 (s, 2H, Ar-H), 8.78 (br s, 1H, Ar-*H*), 8.62 – 8.60 (m, 1H, Ar-*H*), 8.57 (d, *J* = 2.6 Hz, 1H, Ar-*H*), 3.98 (s, 3H, O*CH₃*). ppm. ^{13}C NMR (151 MHz, $CDCl_3$) *δ* 158.34, 148.95, 144.88, 144.15, 80.46, 79.58, 76.20, 73.38, 52.95 ppm. **HRMS (ESI)**: calcd for $C_{14}H_8N_4O_2Na$ [M+Na]$^+$: 287.05395; found 287.0539.

Side-product: 2-[4-(pyrazine-2-yl)buta-1,3-diyn-1-yl]pyrazine (3n_AA)

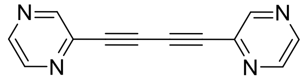

^{1}H NMR (600 MHz, $CDCl_3$) *δ* 8.75 (br s, 1H, Ar-*H*), 8.60 – 8.58 (m, 1H, Ar-*H*), 8.54 (d, *J* = 2.6 Hz, 1H, Ar-*H*) ppm. **^{13}C NMR** (151 MHz, $CDCl_3$) *δ* 148.76, 144.84, 143.97, 79.29, 76.03 ppm. **HRMS (ESI)**: calcd for $C_{12}H_7N_4$ [M+H]$^+$: 207.06652; found 207.0666

Table S3. Increasing yield of Lei ligand catalyzed CC coupling going from *(o)*- to *(m)*- to *(p)*-*N*-incorporation in **3e**-**3g**, and reduced yield with increasing no. of *(o)*-*N*-incorporation in **3e**, **3i**, **3j** and **3k**. [a]Yield theoretically calculated from mixture of hetero- and homocoupled products in 1:0.25 ratio.

Compd. No.	Yield (%) Lei ligand catalyzed CC coupling
**3e**	48
**3f**	67
**3g**	70
**3i**	40[a]
**3j**	17
**3k**	No heterocoupled product

E. General procedure for hydrolysis:

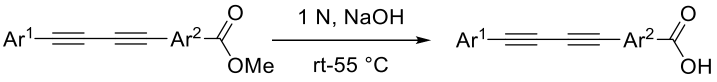

3a, 3f, 3k and **BADY**

7a (95%), **7f** (quant.), **7k** and **BADY-COOH** (97%)

E1. General procedure for aqueous hydrolysis

To a solution of carboxylic ester **3** dissolved in ACN (1 mL for 1 mmol ester) in an oven-dried RBF with PTFE-coated magnetic stir-bar, 1N NaOH (1 mL for 1 mmol ester) was added and the reaction was stirred at rt-55 °C. Reaction progress was monitored by TLC which showed loss of starting material and appearance of a new spot at the baseline, ACN was evaporated and pH was adjusted to 4-5 with 1N HCl at 0 °C. The resultant suspension was chilled overnight at 4 °C followed by filtration and drying of the precipitate *in vacuo* to afford the hydrolyzed products. The acids were taken forward without further purification.

E2. General procedure for methanolic hydrolysis

To a solution of carboxylic ester **3** dissolved in 1:5 MeOH:DCM (1 mL for 1 mmol ester) in an oven-dried RBF with PTFE-coated magnetic stir-bar, 100 mmol aq. NaOH (4 eq) was added and the reaction was stirred at rt. Reaction progress was monitored by TLC which showed loss of starting material and appearance of a new spot at the baseline, solvent was evaporated and pH was adjusted to 4-5 with 0.1N HCl at 0 °C. The resultant suspension was filtered and the residue was dried *in vacuo* to afford the hydrolyzed products.

1-Methyl-5-[4-(1-methyl-1*H*-imidazol-5-yl)buta-1,3-diyn-1-yl]-1H-pyrrole-2-carboxylic acid (7a)

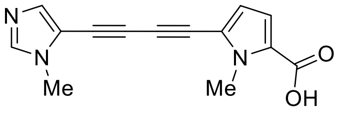

0.25 mmol scale, synthesized via general procedure E1 from **3a**, buff coloured solid, 60 mg, 95% yield. **Mp** 248-250 °C. **^{1}H NMR** (600 MHz, DMSO-d$_6$) *δ* 12.81 (s, 1H, COO*H*), 7.84 (s, 1H, Ar-*H*), 7.52 (s, 1H, Ar-*H*), 6.82 (d, *J* = 4.2 Hz, 1H, Ar-*H*), 6.69 (d, *J* = 4.2 Hz, 1H, Ar-*H*) 3.92 (s, 3H, N*CH$_3$*), 3.69 (s, 3H, N*CH$_3$*) ppm. **^{13}C NMR** (151 MHz, DMSO- d$_6$) *δ* 161.62, 138.34, 126.29, 119.87, 117.50, 117.14, 80.79, 79.54, 75.77, 73.17, 34.71, 32.44 ppm. **HRMS (ESI) (*m/z*)**: calc'd for $C_{14}H_{11}N_3O_2$[M+Na]$^+$: 276.0743; found 276.0750.

4-[4-(pyridin-3-yl)buta-1,3-diyn-1-yl]benzoic acid (7f)

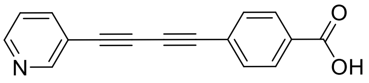

0.6 mmol-scale, synthesized via general procedure E1 from **3f**, buff coloured powder, 130 mg, 88% yield. **Mp** 324 – 326 °C **^{1}H NMR** (600 MHz, DMSO-d$_6$) *δ* 8.83 (dd, *J* = 2.4, 1.0 Hz, 1H, Ar-*H*), 8.65 (dd, *J* = 4.9, 1.7 Hz, 1H, Ar-*H*), 8.07 (dt, *J* = 8.0, 1.8 Hz, 1H, Ar-*H*), 7.95 (d, *J* = 8.4 Hz, 2H, Ar-*H*), 7.67 (d, *J* = 8.4 Hz, 2H, Ar-*H*), 7.50 (ddd, *J* = 8.0, 4.9, 1.0 Hz, 1H, Ar-*H*) ppm. **^{13}C NMR** (126 MHz, DMSO-d$_6$) *δ* 167.24, 153.68, 153.17, 150.44, 140.25, 132.78, 129.96, 124.16, 118.22, 82.87, 80.00, 76.68, 75.09 ppm. **HRMS (ESI)**: calcd for $C_{16}H_{10}NO_2$ [M+H]$^+$: 248.07061; found 248.0706.

6-[4-(pyridin-2-yl)buta-1,3-diyn-1-yl]pyridine-3-carboxylate (7k)*

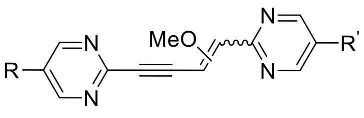

R = H/COOH, R' = H/COOH

0.03 mmol scale, synthesized via general procedure E2 from **3k** resulted in hydrolysis of the ester and Michael addition on one the diyne alkynes by the methoxide anion resulting in a E/Z mixture of **7k**. **^{1}H NMR** (500 MHz, DMSO-d$_6$) *δ* 9.26 (s, 3H, Ar-*H*), 9.20 (s, 2H, Ar-*H*), 8.91 – 8.89 (d, J = 4.9 Hz, 2H, Ar-*H*), 8.85 – 8.82 (d, J = 4.9 Hz, 3H, Ar-*H*), 7.56 – 7.53 (t, J = 4.8 Hz, 1H, Ar-*H*), 7.51 – 7.48 (t, J = 5.0 Hz, 2H, Ar-*H*), 6.75 (s, 1H, C*H*), 6.61 (s, 1H, C*H*), 4.24 (s, 3H, O*CH$_3$*), 4.23 (s, 4H, O*CH$_3$*) ppm. **^{13}C NMR** (126 MHz, CD_3CN+DMSO-d$_6$) *δ* 165.08, 165.03, 164.32, 162.77, 162.46, 160.06, 158.71, 158.60, 158.20, 157.98, 155.01, 152.77, 124.23, 123.16, 121.85, 120.94, 96.66, 95.30, 93.54, 90.33, 87.27, 83.60, 60.41, 60.35 ppm.

*Hydrolysis of **3k** via general procedure F1 resulted in multiple unidentified products

F. Synthesis of Het-DY imaging probes:

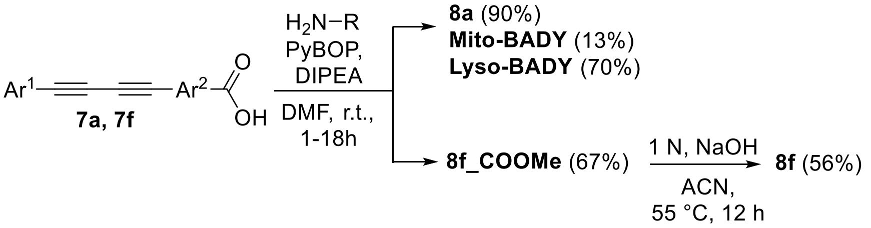

F. General procedure for Peptide coupling: To a solution of carboxylic acid (1 eq) in DMF (10 mL for 1.5 mmol of acid) in an oven-dried RBF with PTFE-coated magnetic stir-bar, amine (1 eq), PyBOP (1.2 eq), and DIPEA (3 eq) were added. The mixture was stirred at room temperature for 4 h to 18 h. Reaction progress was monitored by TLC which showed loss of starting material and appearance of new major non-polar spot. *Work-up a*: After completion, the mixture was poured into water (3× volume of DMF) and stirred for 10 min, the precipitate was filtered and washed with water and then dried *in vacuo* to give crude product. *Work-up b*: Alternatively, when the coupled product did not precipitate upon aqueous workup, extraction with EtOAc, drying over $MgSO_4$ and evaporation of the organic fraction gave the crude product. *Work-up c*: When a product neither precipitated nor separated efficiently upon extraction, the reaction mixture was evaporated to dryness *in vacuo* to afford a crude mixture. The crude (from the above three work-ups) was dissolved in a minimum amount of MeOH, Celite (3× weight of the crude) was added and the solution was evaporated to afford a plug. The resulting plug was loaded on to an automatic flash column for purification (SiO_2, 4 g, 90 mm ⌀, 0-100% EtOAc/petroleum ether or DCM/MeOH, ca. 10 mL fractions). Fractions with the desired R_f (TLC) were pooled and evaporated to dryness to afford the heterocoupled product as solids.

Hydrolysis: See general procedure F.

1-Methyl-5-[4-(1-methyl-1H-imidazol-5-yl)buta-1,3-diyn-1-yl]-*N*-[2-(morpholin-4-yl)ethyl]-1*H*-pyrrole-2-carboxamide (8a or Lyso Het-DY)

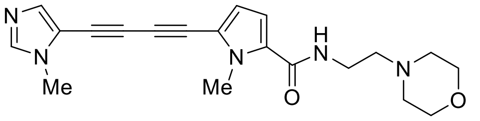

0.2 mmol scale, synthesized via general procedure F from **7a** and commercial 4-(2-aminoethyl)morpholine, reaction went to completion in 1 h, purified by flash column chromatography (SiO_2, 4 g, 40-60 mm ø, 70% MeOH in DCM, ca. 10 mL, R_f = 0.26 D:M 20:1), white powder, 65 mg, 90%. **Mp** 156.9 °C. **^{1}H NMR** (500 MHz, $CDCl_3$) δ 7.50 (s, 1H, Ar-*H*), 7.45 (s, 1H, Ar-*H*), 6.57 (d, *J* = 4.1 Hz, 1H, Ar-*H*), 6.54 (br s, 1H, CO*NH*), 6.49 (d, *J* = 4.1 Hz, 1H, Ar-*H*), 4.03 (s, 3H, N*CH₃*), 3.78 – 3.72 (m, 7H, N*CH₃*, CH₂*CH₂*), 3.54 – 3.47 (m, 2H, NH*CH₂*), 2.59 (t, *J* = 6.1 Hz, 2H, CH₂*CH₂*), 2.55 – 2.49 (m, 4H, CH₂*CH₂*) ppm. **^{13}C NMR** (126 MHz, $CDCl_3$) δ 160.97, 139.02, 137.61, 128.46, 119.44, 116.63, 110.98, 81.16, 79.29, 75.18, 71.45, 67.04, 56.85, 53.42, 53.32, 35.49, 34.37, 32.24 ppm. **HRMS (ESI) (*m/z*)**: calc'd for $C_{20}H_{23}N_5O_2$ [M+H]$^+$: 366.1924; found 366.1924

Methyl 11-({4-[4-(pyridin-3-yl)buta-1,3-diyn-1-yl]phenyl}[formamido)undecanoate (8f_COOMe)

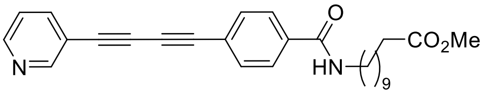

0.1 mmol scale, synthesized via general procedure F from **7f** and methyl 11-amino-undecanoate,[56] reaction went to completion in 12 h, purified by flash column chromatography (SiO_2, 4 g, 40-60 mm ø, 10 % EtOAc in PE, ca. 10 mL, R_f = 0.3 P:E 2:1), white powder, 30 mg, 67%. **Mp** 124-126 °C. **^{1}H NMR** (500 MHz, $CDCl_3$) *δ* 8.79 (s, 1H, Ar-*H*), 8.64 – 8.60 (d, *J* = 3.9 Hz, 1H, Ar-*H*), 7.87 – 7.81 (dt, *J* = 7.9, 1.7 Hz, 1H, Ar-*H*), 7.80 – 7.74 (d, *J* = 8.7 Hz, 2H, Ar-*H*), 7.65 – 7.58 (d, *J* = 8.7 Hz, 2H, Ar-*H*), 7.34 – 7.30 (dd, J = 8.6, 4.2 Hz, 1H, Ar-*H*), 6.13 – 6.07 (m, 1H, CO*NH*), 3.69 (s, 3H, COO*CH₃*), 3.53 – 3.44 (m, 2H, CONH*CH₂*), 2.36 – 2.29 (t, *J* = 7.5 Hz, 2H, *CH₂*COOCH₃), 1.69 – 1.59 (q, *J* = 7.6 Hz, 4H, *CH₂*), 1.44 – 1.30 (m, 12H, *CH₂*) ppm. **^{13}C NMR** (126 MHz, $CDCl_3$) *δ* 174.34, 166.43, 153.16, 149.43, 139.37, 135.38, 132.72, 126.98, 124.44, 123.10, 119.05, 81.67, 79.07, 75.43, 51.46, 40.24, 34.11, 29.72, 29.63, 29.41, 29.31, 29.25, 29.19, 29.10, 26.96, 24.93 ppm. **HRMS (ESI)**: calcd for $C_{28}H_{33}N_2O_3$ [M+H]$^+$: 445.2446; found 445.2485.

11-({4-[4-(pyridin-3-yl)buta-1,3-diyn-1-yl]phenyl}formamido)undecanoate (8f or LD Het-DY)

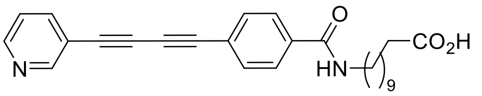

0.1 mmol scale, synthesized via general procedure F from **8f_COOMe**, white powder, 24 mg, 56%. **Mp** 175-177 °C. ^{1}H NMR (500 MHz, DMSO-d*₆*) *δ* 8.83 (d, *J* = 3.2 Hz, 1H, Ar-*H*), 8.66 (dd, *J* = 4.9, 1.8 Hz, 1H, Ar-*H*), 8.57 (t, *J* = 5.7 Hz, 1H, Ar-*H*), 8.07 (dt, *J* = 8.0, 1.8 Hz, 1H, Ar-*H*), 7.89 (d, *J* = 8.6 Hz, 2H, Ar-*H*), 7.72 (d, *J* = 8.6 Hz, 2H, Ar-*H*), 7.53 – 7.47 (ddd, *J* = 8.0, 4.9, 0.9 Hz, 1H, Ar-*H*), 3.28 – 3.23 (m, 2H, CONH*CH₂*), 2.18 (t, *J* = 7.3 Hz, 2H, *CH₂*COOCH₃), 1.55 – 1.45 (m, 4H, *CH₂*), 1.34 – 1.21 (m, 12H, *CH₂*) ppm. ^{13}C NMR (126 MHz, $CDCl_3$) *δ* 165.51, 153.19, 150.48, 140.25, 136.23, 132.92, 128.08, 124.17, 122.99, 118.18, 86.56, 82.59, 80.02, 76.61, 37.18, 34.24, 29.46, 29.39, 29.33, 29.20, 29.04, 26.93, 25.02 ppm. **HRMS (ESI)**: calcd for $C_{27}H_{31}N_2O_3$ [M+H]$^+$: 431.2290; found 431.2329.

G. Synthesis of Lei Ligand

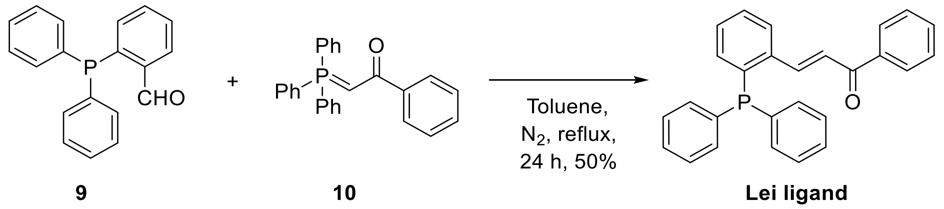

To an oven-dried 2-necked RBF with an oven-dried PTFE-coated magnetic stir-bar and charged with degassed toluene (10 mL) under N_2 atmosphere, 2-(Diphenylphosphino)benzaldehyde **8** (490 mg, 1.55 mmol, 1 eq) and 2-phenyl(triphenylphosphoranylidene)ethan-2-one **9** (1.17 g, 3.1 mmol, 2 eq) were added. The resulting mixture was heated to reflux for 24 h under inert conditions. TLC showed the completion of reaction with loss of the benzaldehyde spot and formation of a new polar spot (R_f = 0.3 P:E 2:1) . After completion, minimum amount of MeOH and Celite (3× weight of the crude) were added and the solution was evaporated to afford a plug. The resulting plug was loaded on to an automatic flash column for purification (SiO_2, 12 g, 40-60 mm ø, 3% EtOAc in PE, ca. 10 mL fractions). Fractions with desired R_f were pooled and evaporated to afford a sticky yellow solid which was resuspended in methanol to form a precipitate. The suspension was filtered and the residue was dried to afford **Lei ligand** as a cream solid (300 mg, 50%). Mp 123-127 °C. **^{1}H NMR** (600 MHz, $CDCl_3$) δ 8.39 – 8.34 (dd, J = 15.8, 4.4 Hz, 1H), 7.79 – 7.73 (m, 3H), 7.57 – 7.52 (m, 1H), 7.49 – 7.26 (m, 14H), 7.21 – 7.17 (d, J = 15.7 Hz, 1H), 6.98 – 6.94 (ddd, J = 7.8, 4.3, 1.4 Hz, 1H) ppm. **^{13}C NMR** (126 MHz, $CDCl_3$) δ 191.91, 143.65, 139.83, 138.51, 137.98, 135.88, 134.17, 133.72, 132.42, 129.94, 129.12, 128.99, 128.64, 126.99, 125.67 ppm. **^{31}P NMR** (162MHz, CDCl3) δ -13.78 ppm. The spectroscopic data are in agreement with those reported in the literature.[35]

4. Spontaneous Raman Spectroscopy

Spontaneous Raman spectra of individual compounds in DMSO (10 mM) or in combination with internal standard EdU (100 mM) were acquired from sample droplets. The raw spectra were baseline corrected using the Zhangfit method, and further smoothened and normalized using Origin 9.6.0.172 (OriginLab Corporation).[57]

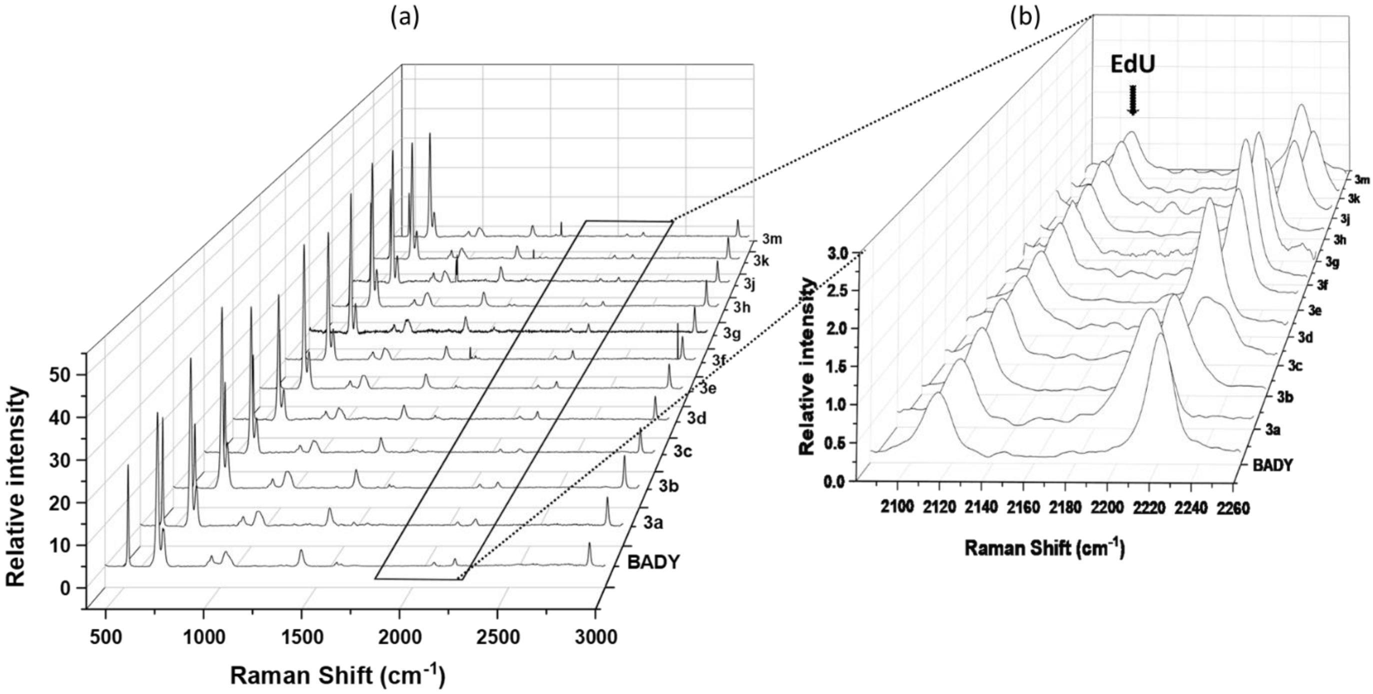

Figure S1. (a) Full spontaneous Raman spectra (500 cm^{-1} to 3000 cm^{-1}) of DMSO solutions of **Het-DY** (10 mM) with internal standard EdU (100 mM). Tags **3i**, **3l** and **3n** were excluded because of insufficient pure fractions available to acquire good signal intensities. Raman signal of EdU (2112 cm^{-1}) was normalized to 1; (b) Zoom in of (a) between 2080 cm^{-1} and 2260 cm^{-1} highlighting the relative intensities of tags **3a-3h**, **3j**, **3k** and **3m** compared to EdU and to each other.

5. Sample Preparation for SRS microscopy, Image Acquisition and Analysis

Cell Culture: Human ovarian cancer cell line ES2 or HeLa cells was purchased from the American Type Culture Collection, and cells maintained in a humidified incubator at 37°C and 5% CO_2 in Dulbecco's Modified Eagle Medium (high glucose, Pyruvate, L-Glutamine) (SIGMA, cat#D6429) and 10% FCS. Cells were plated into Fluorodish Cell Culture Dishes (World Precision Instruments, cat#FD-35-100) for 24 hours before treatment with compound. Details of the dosing concentrations and dosing times are provided in the figure legends. For multi-modal imaging experiments cells were treated with either Wheat Germ Agglutinin Alexa Fluor 594 Conjugate (ThermoFisher cat#W11262), LysoTracker Red DND-99 (ThermoFisher cat#L7528), MitoTracker Red CMXRos (ThermoFisher cat#M7512) or Nile Red (Merck cat#72485) made up as per instructions with matching SRS probe. BADY and Het-DY probes were made up in DMSO. All dilutions were made in culture media and cells imaged live.

Two-photon fluorescence and stimulated Raman scattering (SRS) microscopy. Images were acquired using a custom-built multi-modal microscope setup. A picoEmerald S (APE, Berlin, Germany) laser provided both a tunable pump laser (700–990 nm, 1.6 ps, 80 MHz repetition rate) and a spatially and temporally overlapped Stokes laser (1031 nm, 1.8 ps, 80 MHz repetition rate). The output beams were inserted into the scanning unit of an Olympus FV1000MPE microscope using a series of dielectric mirrors and a 4× lens based beam-expanding module. The resulting 3.4 mm beams were expanded by a further 3.6 lens within the microscope and directed into an Olympus XLPL25XWMP N.A. 1.05 objective lens using a short-pass 690 nm dichroic mirror (Olympus). Backscattered emission signals from two-photon fluorescence was separated from any reflected excitation light using a short-pass 690 nm dichroic mirror and IR cut filter (Olympus). A series of filters and dichroic mirrors (FF552-Di02, and FF440/520-Di01 (Semrock) and HQ610/75m (Chroma) were then used to deconvolve the different emission signals onto an available photo-multiplier tubes.

For SRS measurements, the Stokes beam was modulated with a 20 MHz EoM built into the picoEmerald S. Forward scattered light was collected by a 25× Olympus XLPL25XWMP N.A. 1.05 objective lens and Stokes light was removed by filtering with an ET890/220m filter (Chroma). A telescope focused the light onto an APE silicon photodiode connected to an APE lock in amplifier with the time constant set to 20 µs The lock in amplifier signal was fed into an Olympus FV10-Analog unit. Images at different wavelengths were acquired sequentially by changing the pump laser wavelength. Fluorescence images where acquired at the alkyne wavelength pump tuning with a 4 µs pixel dwell time whilst SRS images were recorded with a 20 µs pixel dwell. Laser powers after the objective

were measured at 20-40 mW for the pump laser and up to 70 mW for the Stokes laser. All images were recorded at 512 × 512 or 1024 × 1024 pixels using FluoView FV10-ASW scanning software (Olympus) and cropped to size for publication.

To remove background processes from the SRS images of alkynes, off resonance images were acquired by tuning the pump wavelength 2 nm (~30 cm^{-1}) from the on-resonance image and then subtracted from the on resonance image using the 'Image Calculator' function of ImageJ. ImageJ was also used to adjust the displayed brightness/contrast of the image as well as assign false colour assignments and scale bars.

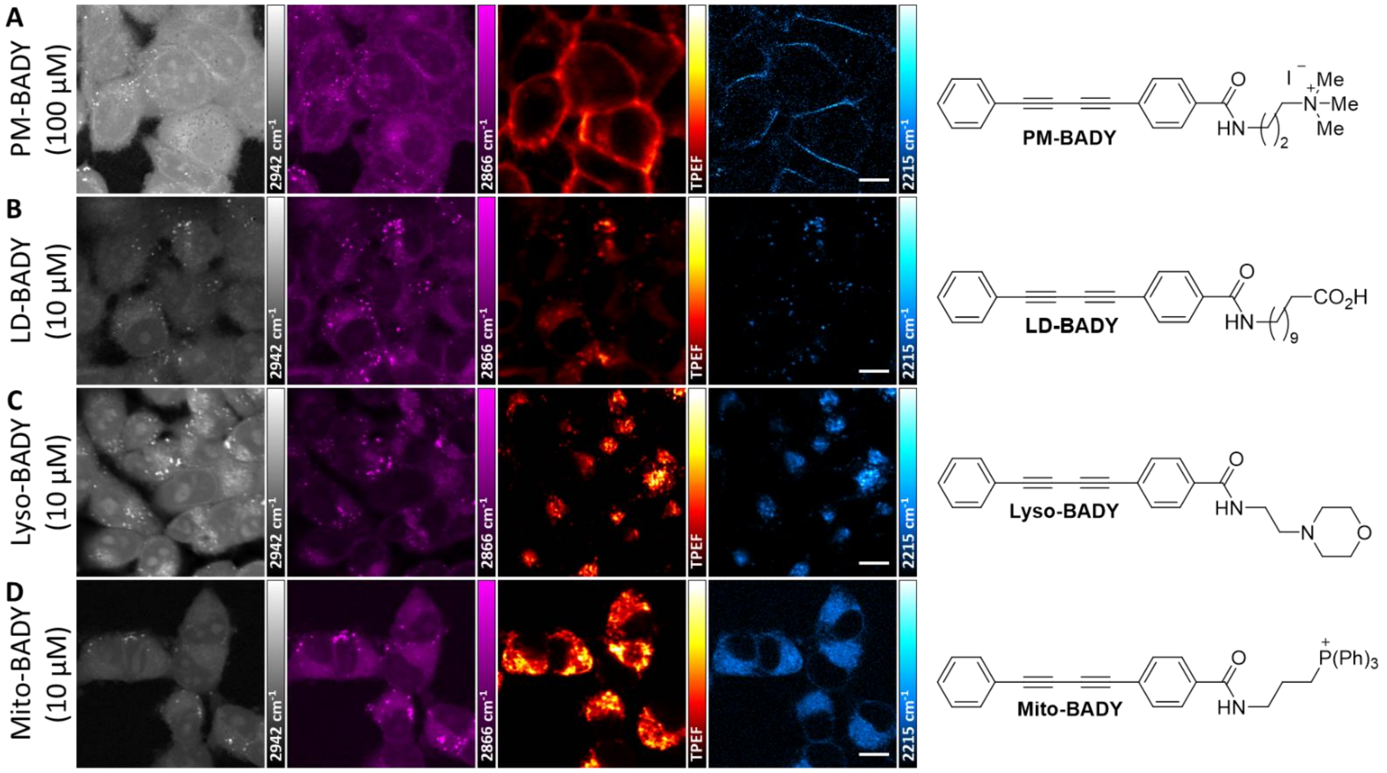

Figure S2. Multimodal images of cells. (A) HeLa cells treated with PM-BADY (100 µM, 30 mins) and Wheat Germ Agglutinin Alexa Fluor 594 Conjugate (100 nM, 30 mins) (B) ES2 cells treated with LD-BADY (10 µM, 24 hours) and Nile Red (10 µM, 30 mins) washed in PBS for imaging, (C) HeLa cells treated with Lyso-BADY (10 µM, 1 hour) and LysoTracker Red DND-99 (100 nM, 1 hour) washed in PBS for imaging and (D) ES2 cells treated with Mito-BADY (10 uM, 1 hour) and MitoTracker Red CMXRos (100nM, 15 mins) washed in PBS for imaging. Images shown are: CH_3, proteins 2942 cm^{-1} – grey scale; CH_2, lipids 2866 cm^{-1} – magenta; TPEF, - red hot and C≡C, alkyne, images taken at 2215 with an off resonance image taken ~30 cm^{-1} away subtracted – cyan hot. Scale bars: 10 µM. The synthesis of **PM-BADY**[58], **LD-BADY**, **Lyso-BADY**[59] and **Mito-BADY**[60] were synthesized via General procedures G1, H and published procedures.

6. Cell Viability Assay

Cell Culture. Human ovarian cancer cell line ES2 was purchased from the American Type Culture Collection, and cells maintained in a humidified incubator at 37°C and 5% CO_2 in Dulbecco's Modified Eagle Medium (DMEM, high glucose, Pyruvate, No Glutamine) (Gibco, cat#21969035), 10% FCS, 1% L-Glutamine (200 mM, ThermoFisher, cat#25-030-081), 1% Pen/Strep (ThermoFisher, cat# 15-140-122).

AlamarBlue cell viability assay. ES2 cells were plated in sextuplicate wells in a 96-well plate at 3000 cells per well. Compounds were made up as 10 mM stocks in DMSO and added to the cells after 24 h at a range of concentrations (100 µM, 30 µM, 10 µM, 3 µM, 1 µM, 0.3 µM, 0.1 µM, 0.03 µM, 0.01 µM, DMSO only) in 100µl. As a background control 6 wells were plated with media with added DMSO and without cells. After 72 h, 11µl (10%) alamarBlue cell viability reagent (ThermoFisher, cat# DAL1025) was added to each well, including the background control. The plate was incubated at 37°C for 4 h after which the absorbance of the wells was measured with a standard spectrophotometer (Tecan Spark 20M) read at 540nm excitation and 580nm emission wavelength. For each sample the background signal intensity was subtracted from the sample signal intensity and then normalized to the DMSO control and displayed as a percentage of the control. Subsequent analysis was performed on GraphPad Prism 9 software where the concentrations were converted to a logarithmic scale to which a non-linear regression curve was fitted using the log(inhibitor) vs normalized response with a variable slope model to calculate EC50 values.

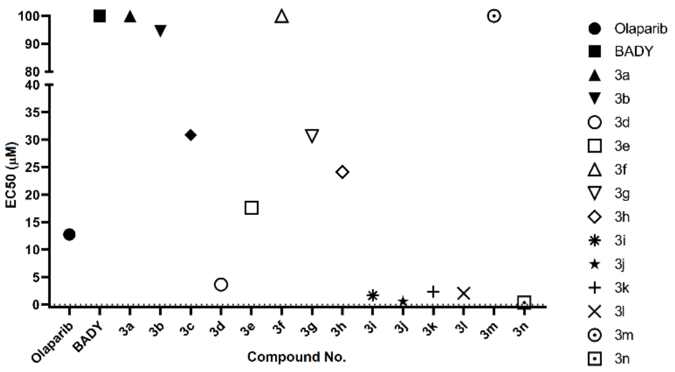

Figure S3. EC_{50} values for compounds **3a** – **3n** in ES2 cells. Values calculated from 6 technical replicates following incubation for 72 h.

7. GSH-reactivity Assay

LC-MS Assay: To 0.5 ml of GSH (50 mmol) in assay buffer (100 mM PBS in H_2O) at 37 °C, 0.5 ml of the diyne tag (5 mmol) in CH_3CN was added to give a final concentration of 25 mmol and 2.5 mmol of GSH and diyne tags respectively. A magnetic stirbar was added to the assay mixture and stirred at 37 °C. Aliquots from the assay mixture were pipetted out and diluted for LC-MS (ESI+) submissions at 5 min, 2 h and 24 h time points. The chromatogram peaks at 254 nm were extracted to pick the UV-active $[M+H]^+$ values corresponding to the unreacted tags and GS-Tag adducts. Relative intensity (or % conversion) of GS-Tag adduct compared to the unreacted tag for individual LC-MS run was calculated by taking the percentage of ^{mAU}GS-Tag divided by ^{mAU}GS-Tag + ^{mAU}unreacted tag (if A -> B, then % conversion = B/A+B). When $[M+H]^+$ peak of the unreacted tag was below detectable limits, adduct conversion was calculated to a 100% and noted as complete conversion.

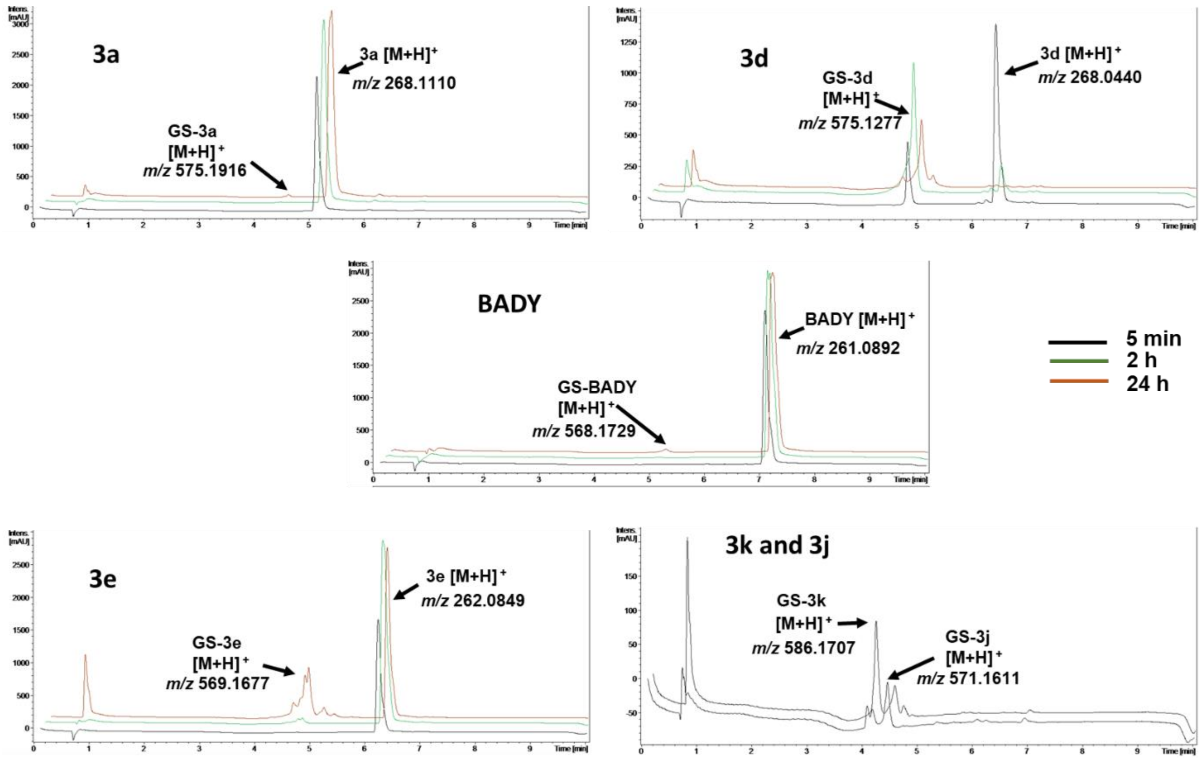

Figure S4. Overlay of the UV-active chromatograms extracted at 254 nm from the LC-MS runs of **3a**, **3d**, **BADY**, **3e**, **3j** and **3k**. The chromatograms for each tag at 5 min (black), 2 h (green), 24 h (red) are overlaid. Minimal (**3a**, **BADY**), partial (**3e**) and full (**3d**, **3j** and **3k**) conversion were observed which correlated with the ring electronics. Tags **3j** and **3k** showed full conversion to adducts within 5 minutes; the 5 minute (black) runs of **3j** and **3k** are overlaid in a single graph. Conversion is deemed complete when the tag (M) was below detectable limits. * Tags isolated as a mixture of hetero- and homodimers or insoluble in the assay buffer (**3i** and **3c**) were not included in the study. Tags **3b, 3f** and **3g** have not been shown as their reactivities were similar to the electronically similar **3a**, **BADY** and **3e** respectively.

NMR Assay: To 0.5 ml of assay buffer (1:1 CD_3CN : 100 mM PBS in H_2O), the individual diyne tags (10 mmol) were added, transferred to an NMR tube and ^1H NMR spectra was acquired. To another 0.5 ml of the assay buffer, GSH (100 mmol) was added, which was then transferred to the NMR tube containing the buffer solution of the tags. The solution was mixed by inverting the tube several times. A final solution of 5 mmol tag and 50 mM GSH was achieved, a magnetic stirbar was added to the tubes and the NMR tubes were left on a stirplate at 37 ˚C. ^1H NMR was recorded at 310 K (37 ˚C) after ~5 min and 24 h of reaction set up.

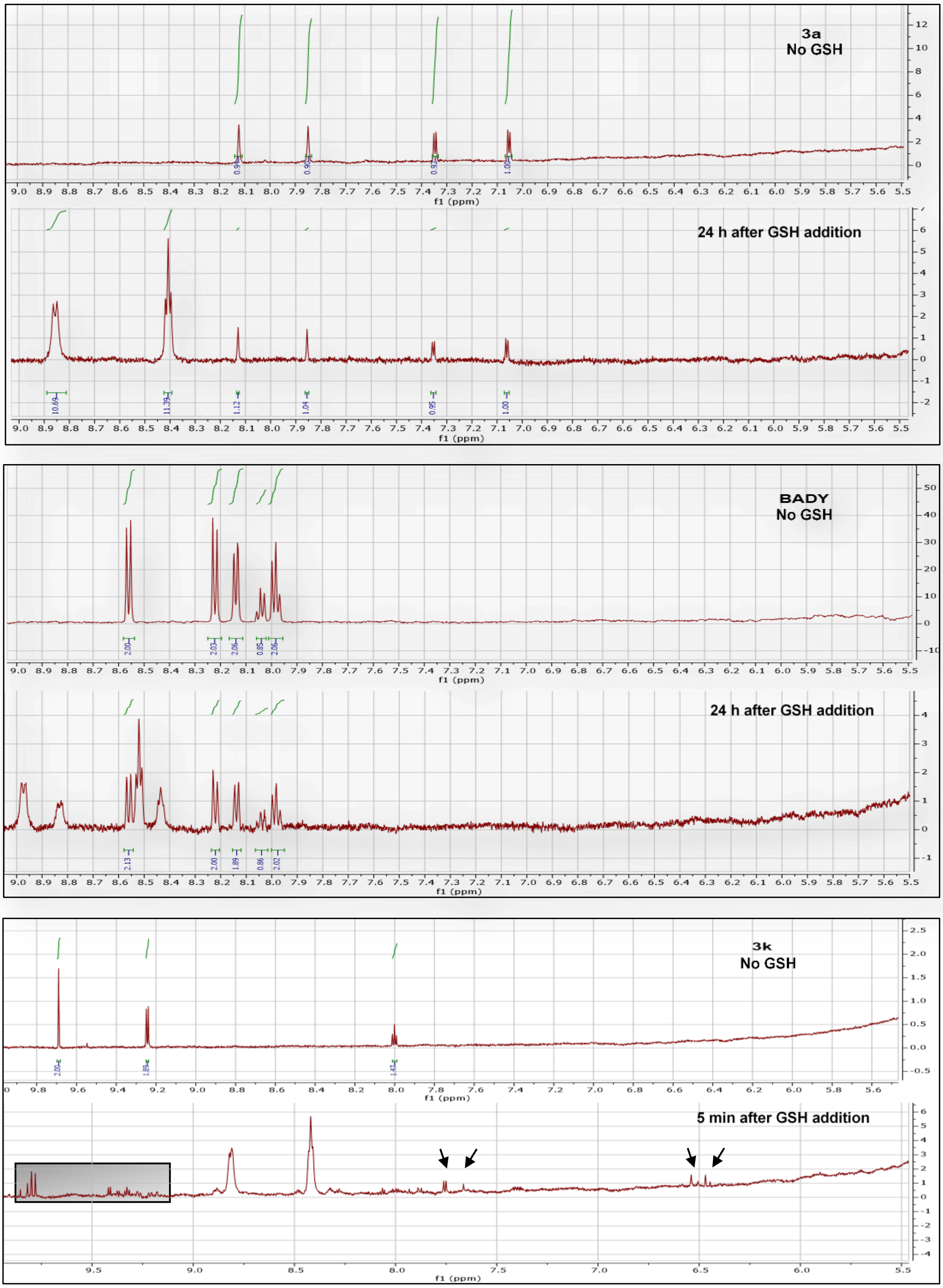

Figure S5. ^{1}H NMR of **3a**, **BADY** and **3k** in the absence and presence of GSH (at 37 ˚C) to identify loss/change in integration of starting material peaks in the aromatic region and appearance of new peaks in both aromatic and alkene region (5-9 ppm). **3a** and **BADY** show no changes in the ^{1}H NMR 24 h after addition of GSH. **3k** shows several new peaks in the alkene and aromatic regions (box and arrows) along with loss of starting material peaks ~5 min after GSH addition.

^{1}H and ^{13}C NMRs

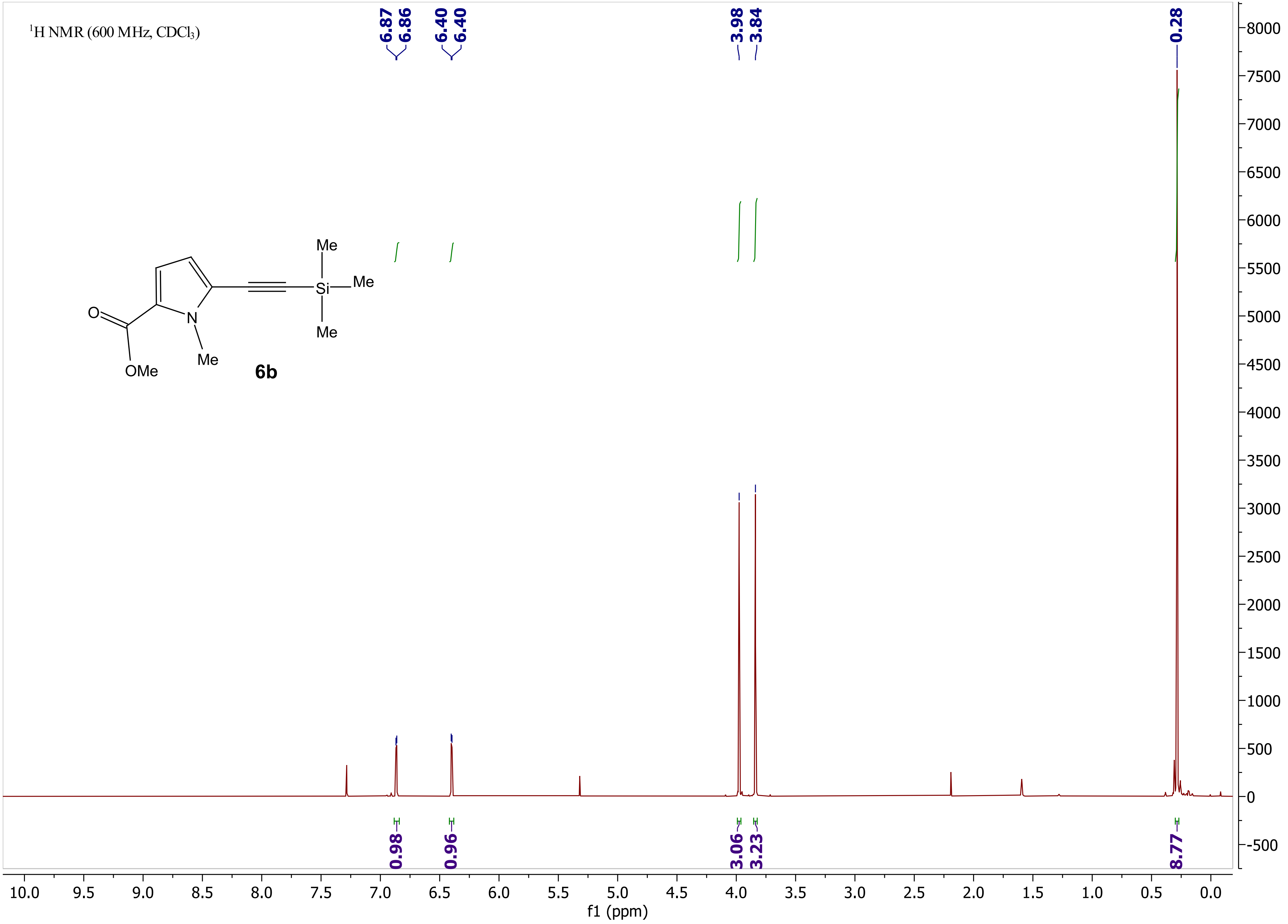
1H NMR (600 MHz, CDCl3)
6.87
6.86
6.40
6.40
3.98
3.84
0.28
0.98
0.96
3.06
3.23
8.77
Me
Si
Me
Me
O
OMe
N
Me
6b
10.0
9.5
9.0
8.5
8.0
7.5
7.0
6.5
6.0
5.5
5.0
4.5
4.0
3.5
3.0
2.5
2.0
1.5
1.0
0.5
0.0
f1 (ppm)
8000
7500
7000
6500
6000
5500
5000
4500
4000
3500
3000
2500
2000
1500
1000
500
0
-500

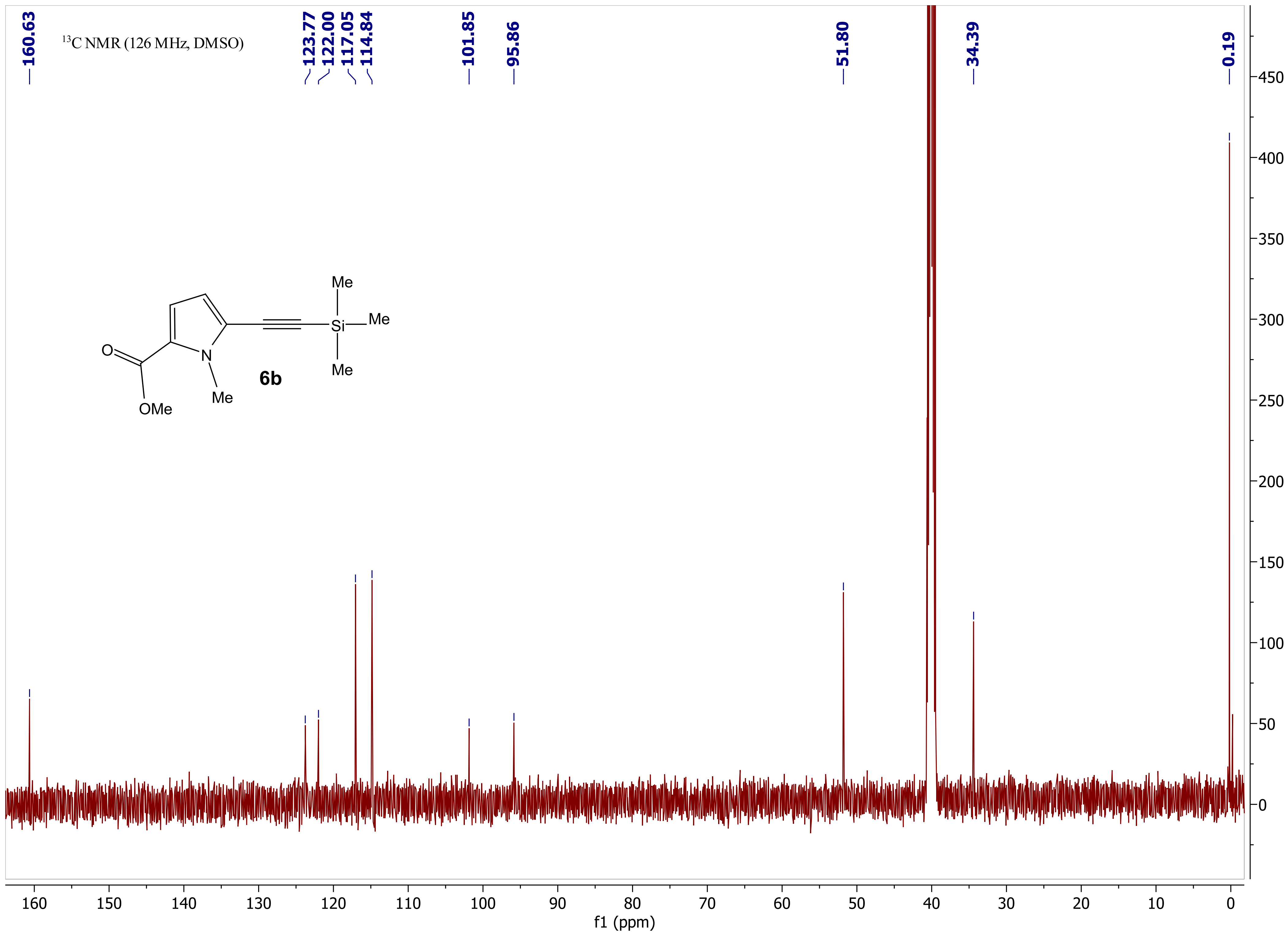
160.63
¹³C NMR (126 MHz, DMSO)
123.77
122.00
117.05
114.84
101.85
95.86
51.80
34.39
0.19
O
N
Me
OMe
Si
Me
Me
Me
6b
f1 (ppm)

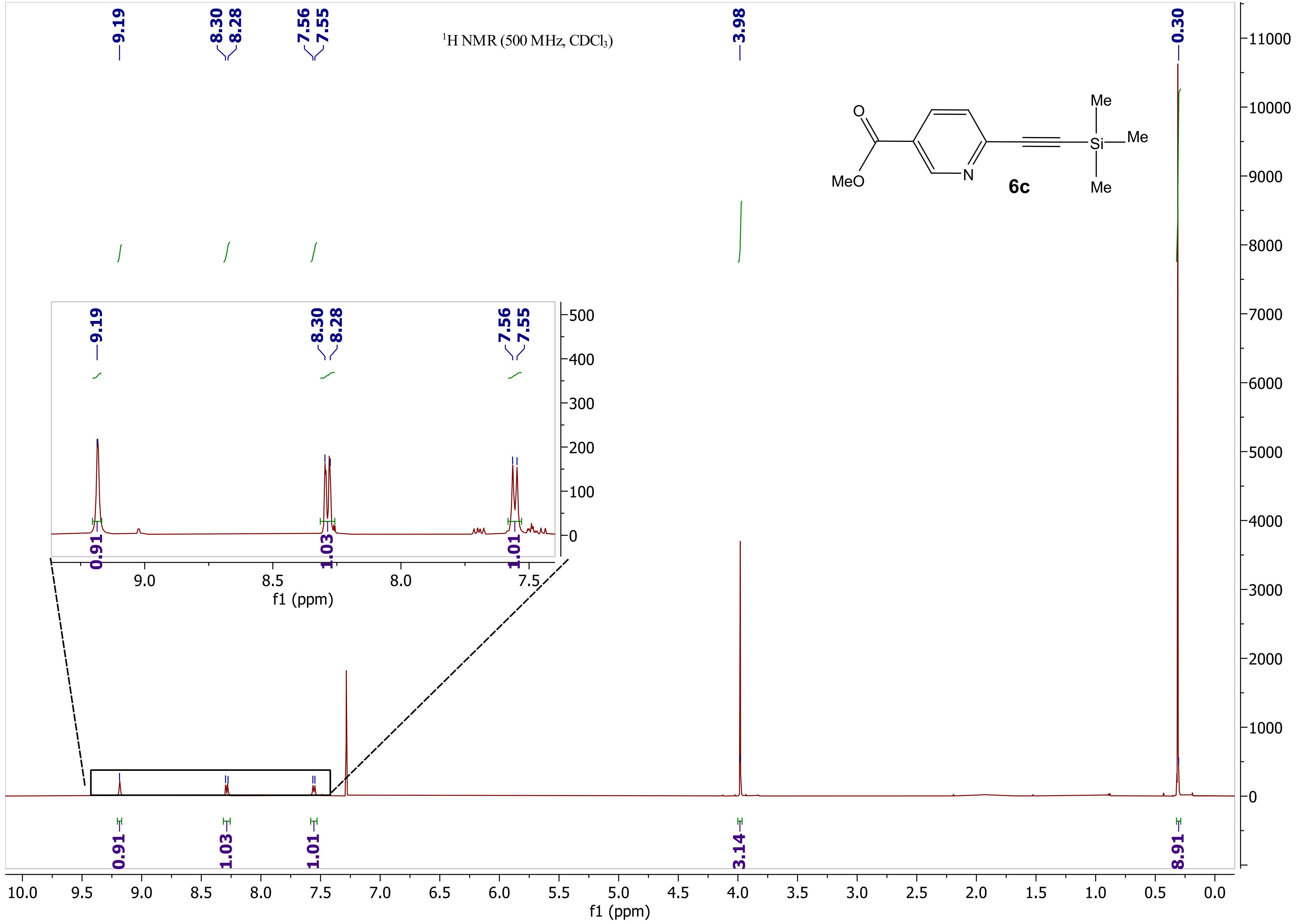

¹H NMR (500 MHz, CDCl₃)
9.19
8.30
8.28
7.56
7.55
3.98
0.30
MeO
O
N
Si
Me
Me
Me
6c
0.91
1.03
1.01
3.14
8.91
f1 (ppm)

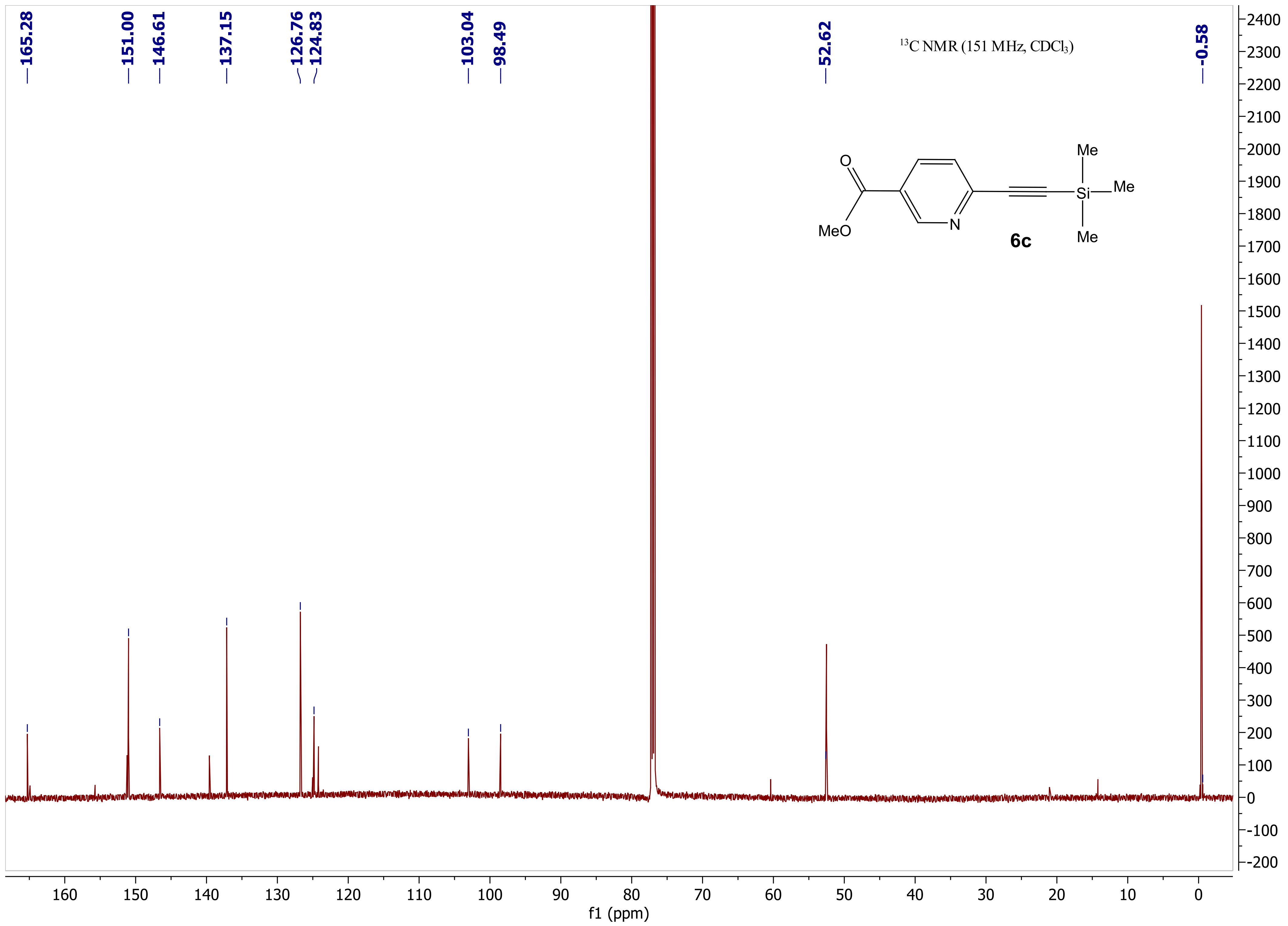
165.28
151.00
146.61
137.15
126.76
124.83
103.04
98.49
52.62
-0.58
^{13}C NMR (151 MHz, CDCl$_3$)
O
MeO
N
Si
Me
Me
Me
6c
f1 (ppm)

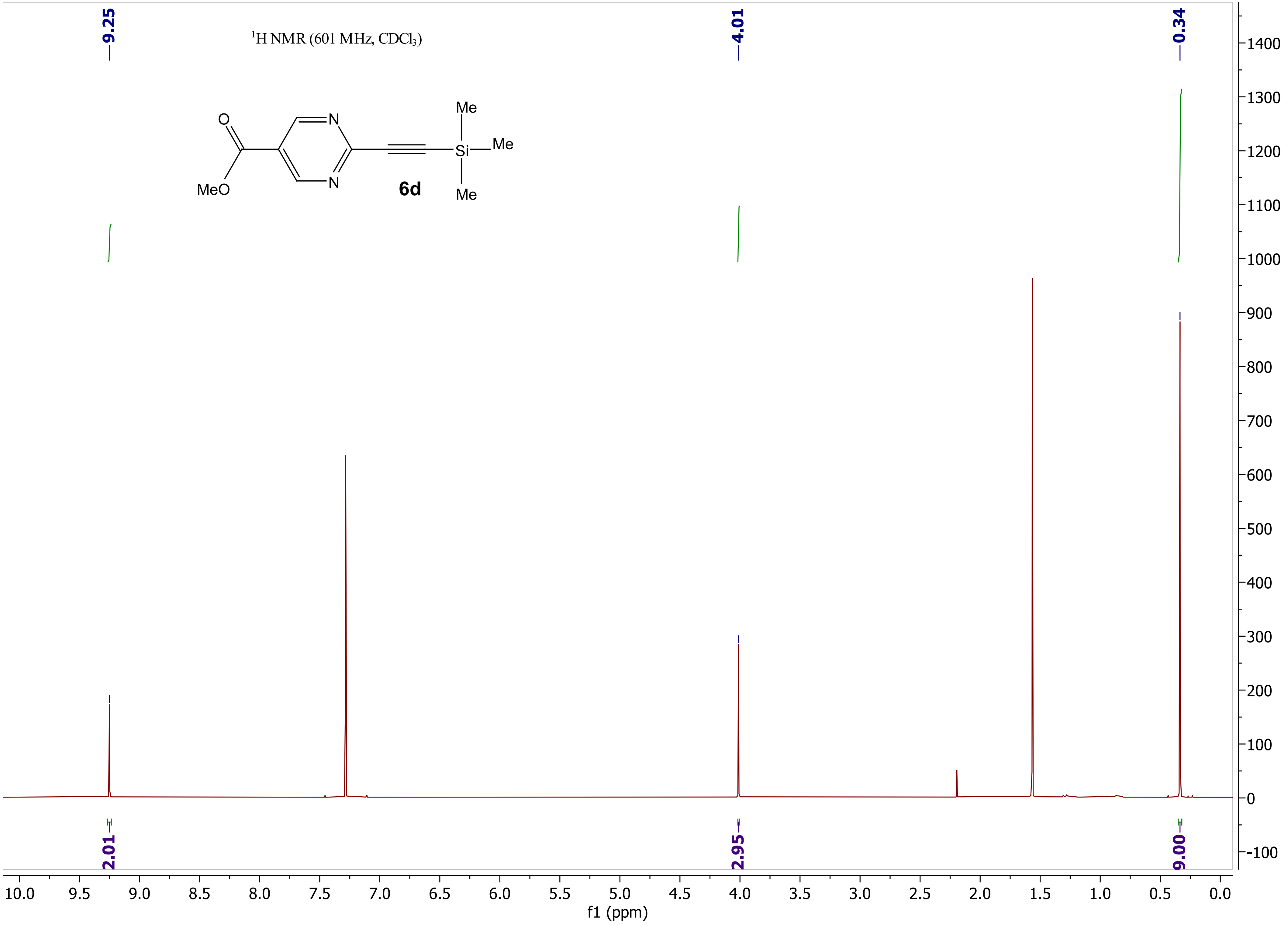

9.25
4.01
0.34
^{1}H NMR (601 MHz, CDCl$_3$)
O
N
N
MeO
Me
Si
Me
Me
6d
2.01
2.95
9.00
f1 (ppm)

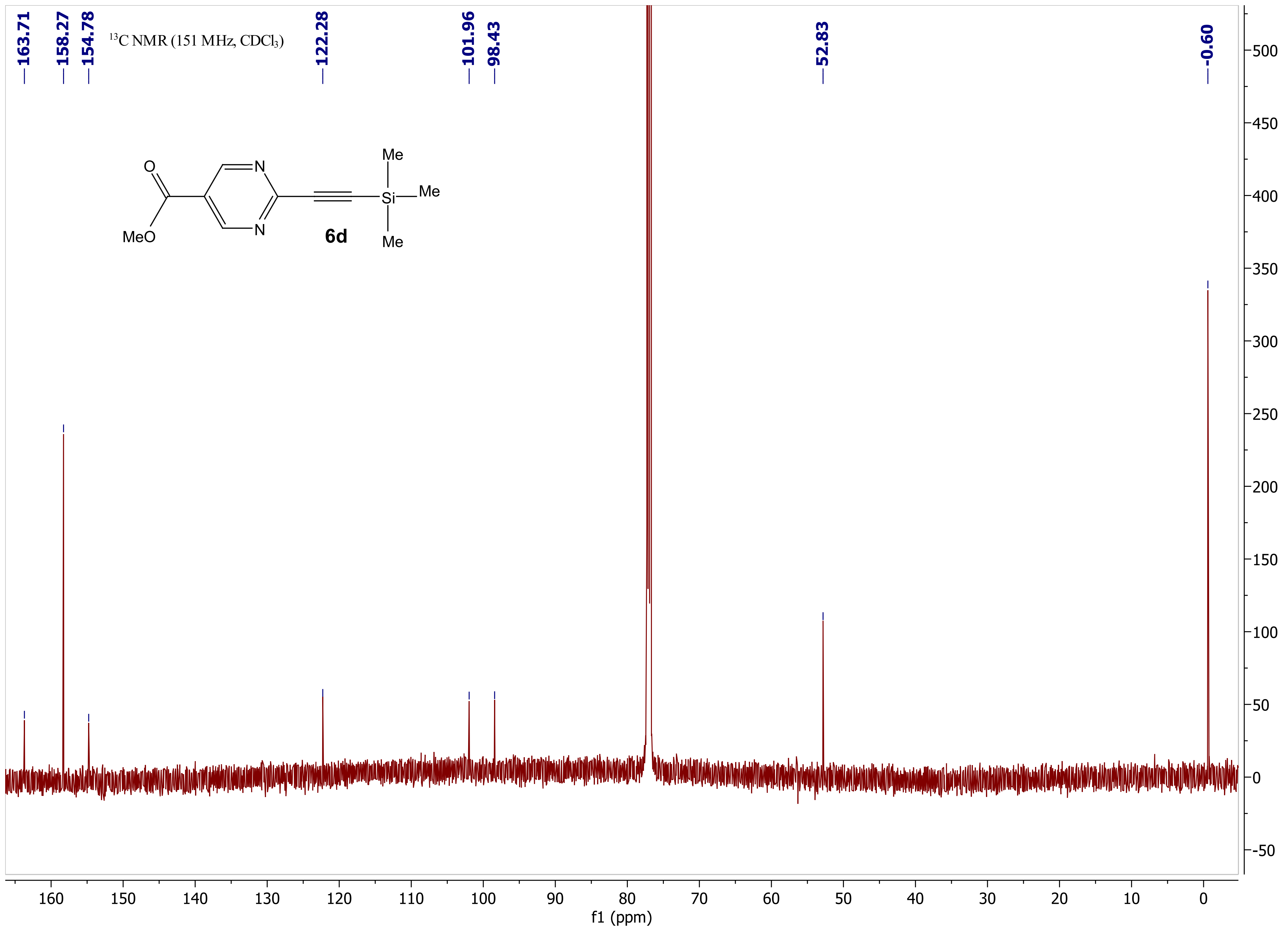

163.71
158.27
154.78
^{13}C NMR (151 MHz, CDCl$_3$)
122.28
101.96
98.43
52.83
-0.60
O
N
N
MeO
Me
Me
Me
Si
6d
f1 (ppm)

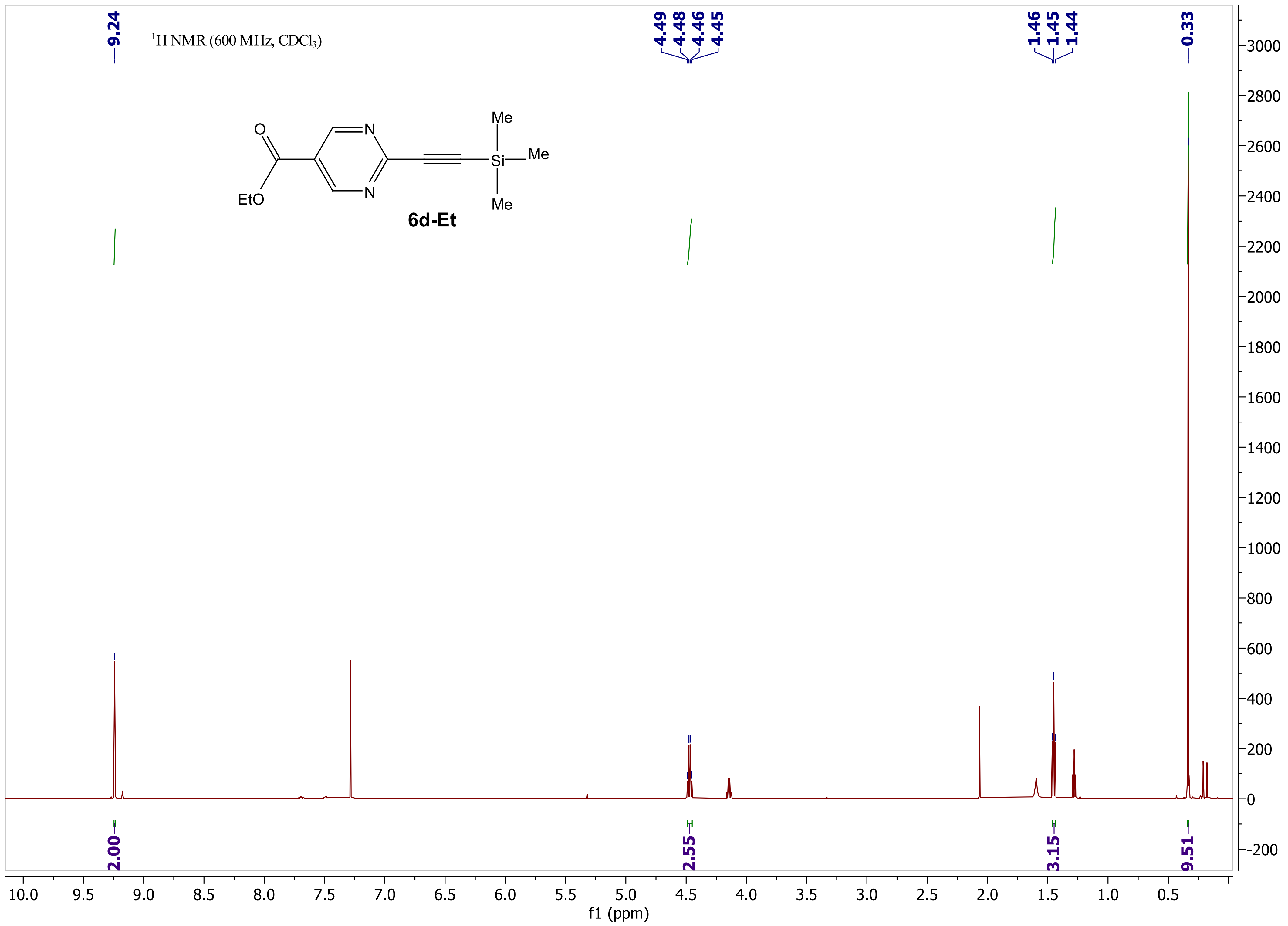

^{1}H NMR (600 MHz, CDCl$_3$)
6d-Et
EtO
O
N
N
Si
Me
Me
Me
9.24
4.49 4.48 4.46 4.45
1.46 1.45 1.44
0.33
2.00
2.55
3.15
9.51
f1 (ppm)

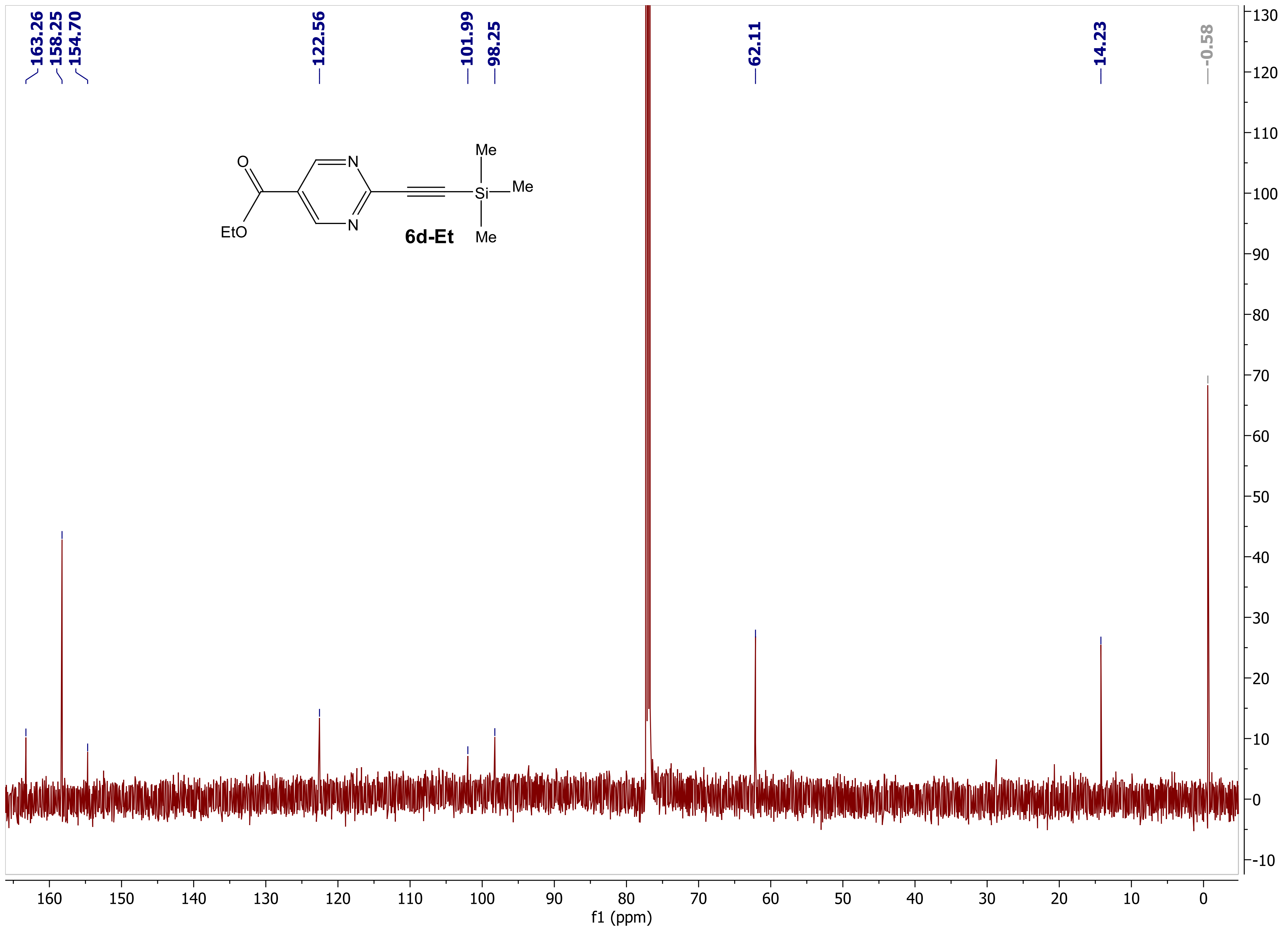
163.26
158.25
154.70
122.56
101.99
98.25
62.11
14.23
-0.58
O
N
EtO
N
Si
Me
Me
Me
6d-Et
f1 (ppm)

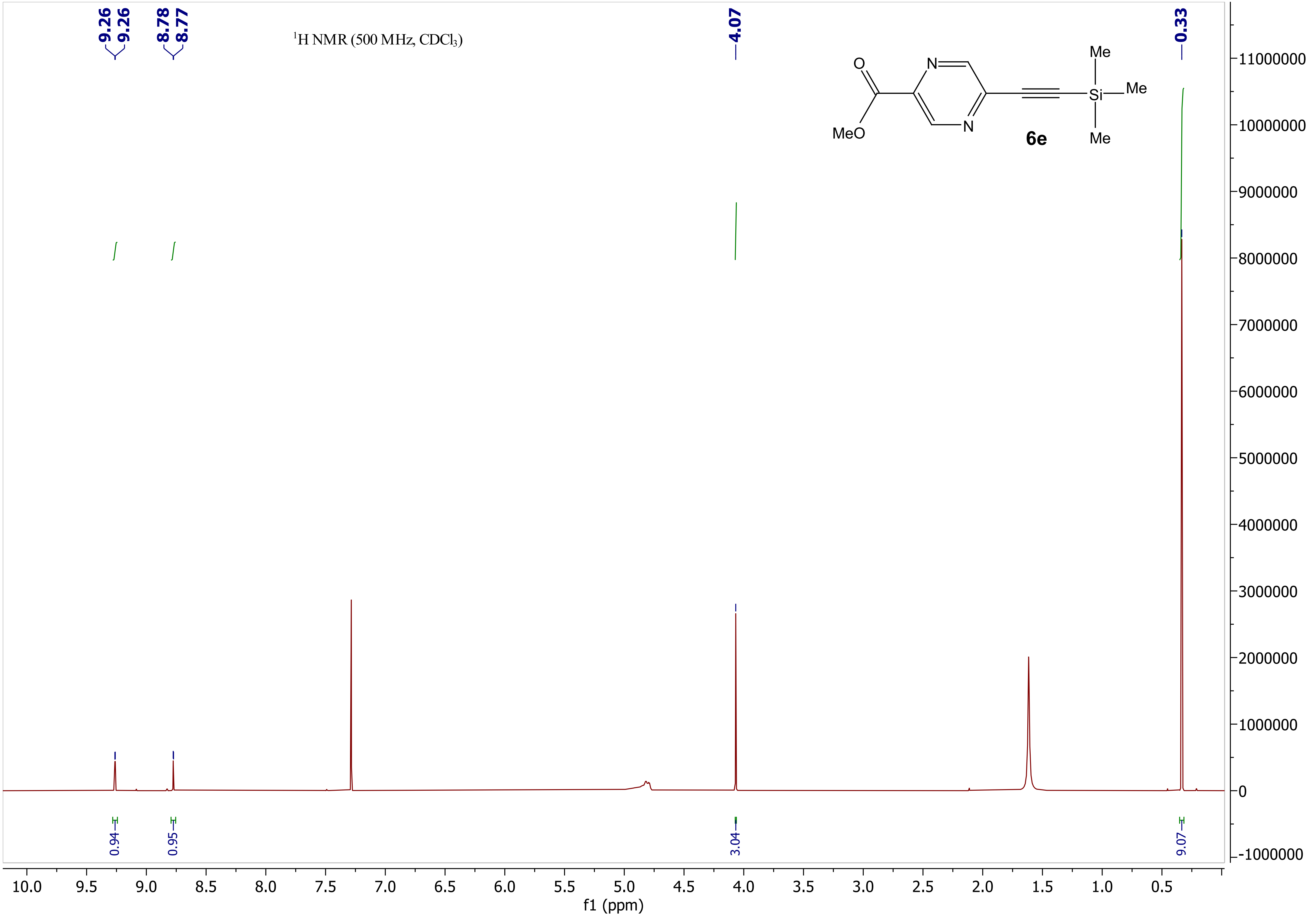

9.26
9.26
8.78
8.77
4.07
0.33
^{1}H NMR (500 MHz, $CDCl_3$)
O
MeO
N
N
Si
Me
Me
Me
6e
0.94
0.95
3.04
9.07
f1 (ppm)

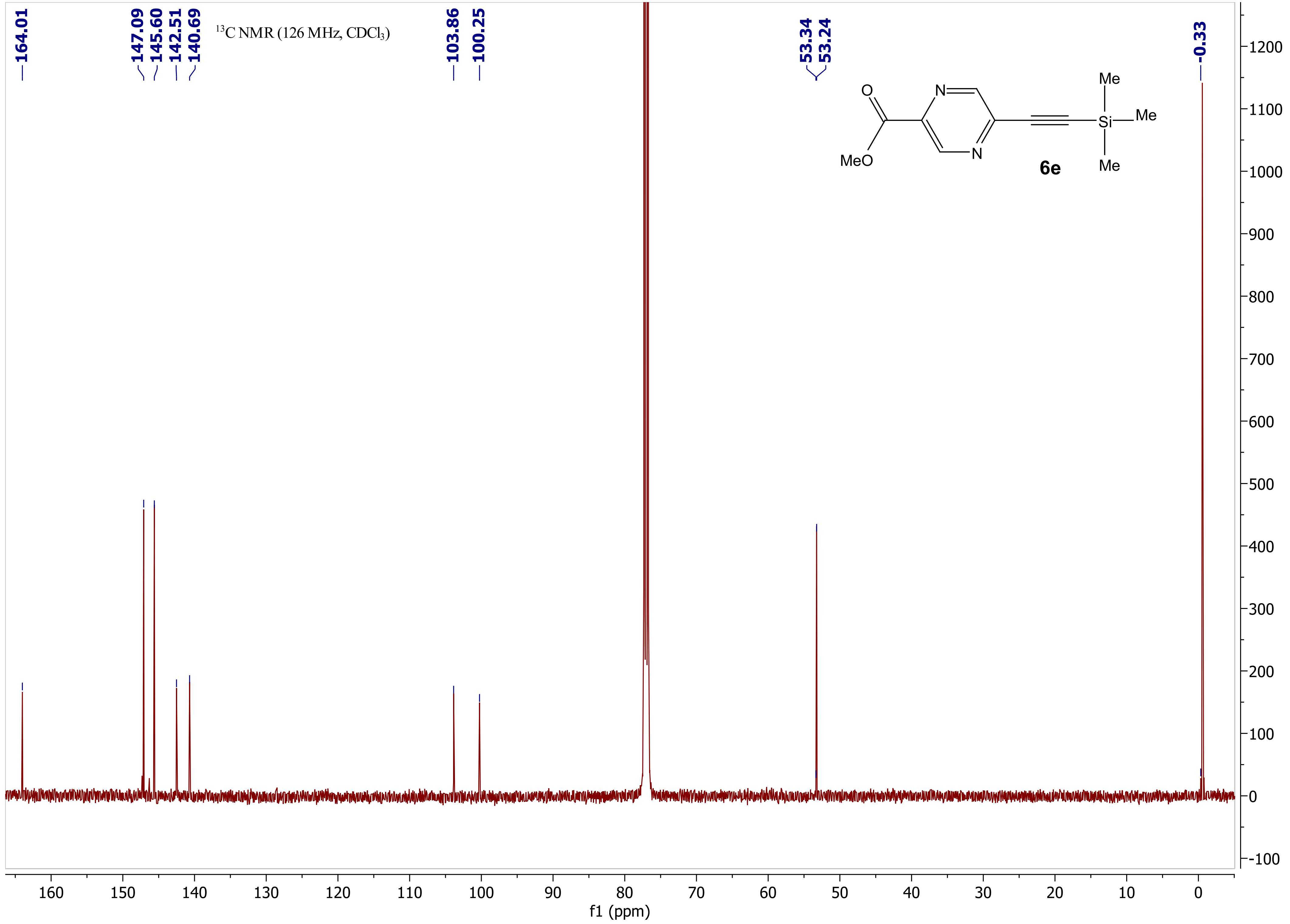

164.01
147.09
145.60
142.51
140.69
^{13}C NMR (126 MHz, $CDCl_3$)
103.86
100.25
53.34
53.24
-0.33
MeO
O
N
N
Si
Me
Me
Me
6e
f1 (ppm)

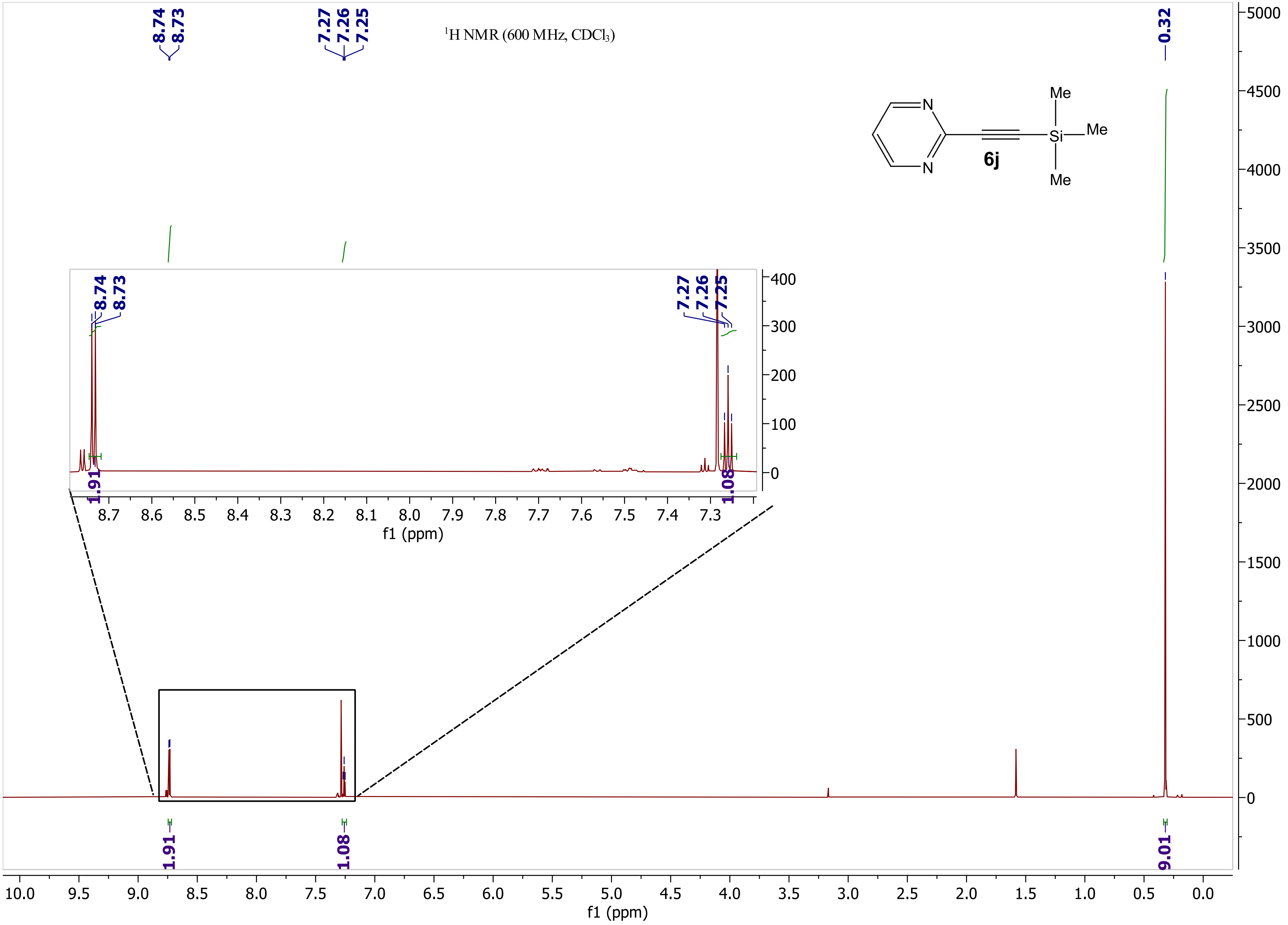

1H NMR (600 MHz, CDCl3)
8.74
8.73
7.27
7.26
7.25
0.32
N
N
Me
Me
Me
Si
6j
1.91
1.08
9.01
f1 (ppm)

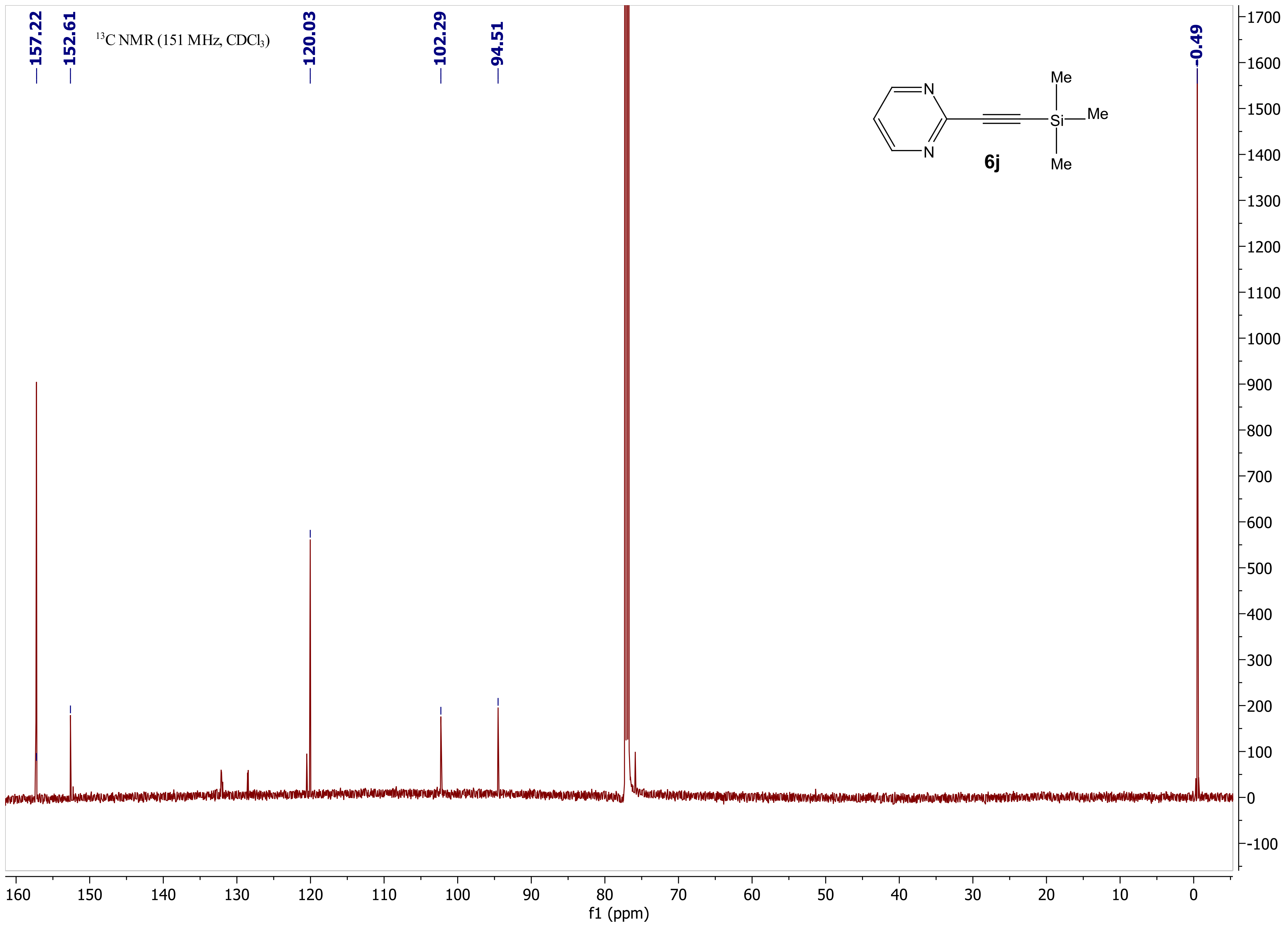
157.22
152.61
13C NMR (151 MHz, CDCl3)
120.03
102.29
94.51
-0.49
N
N
Me
Si
Me
Me
6j
f1 (ppm)

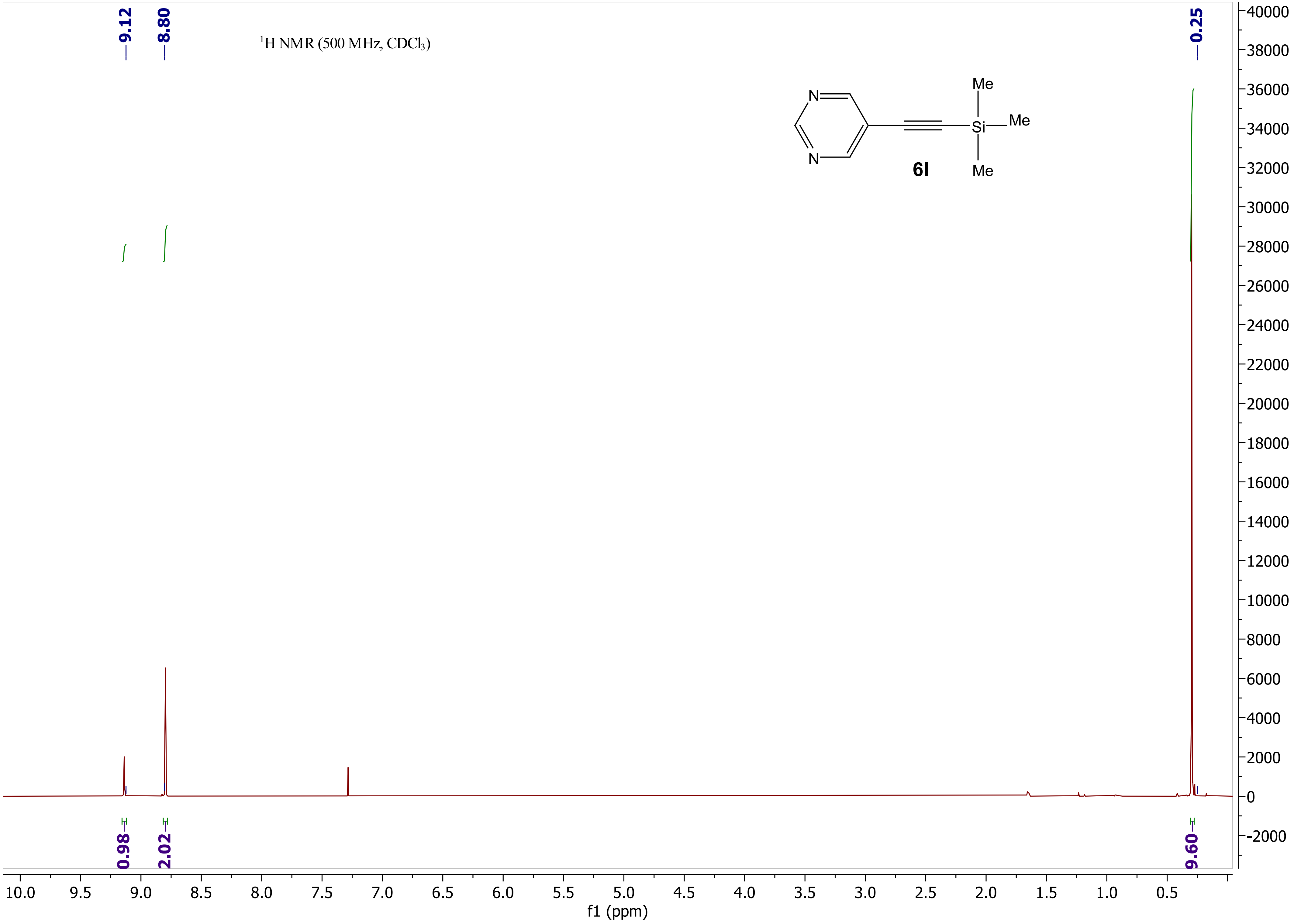

9.12
8.80
0.25
¹H NMR (500 MHz, CDCl₃)
N
N
Me
Me
Me
Si
6l
0.98
2.02
9.60
f1 (ppm)

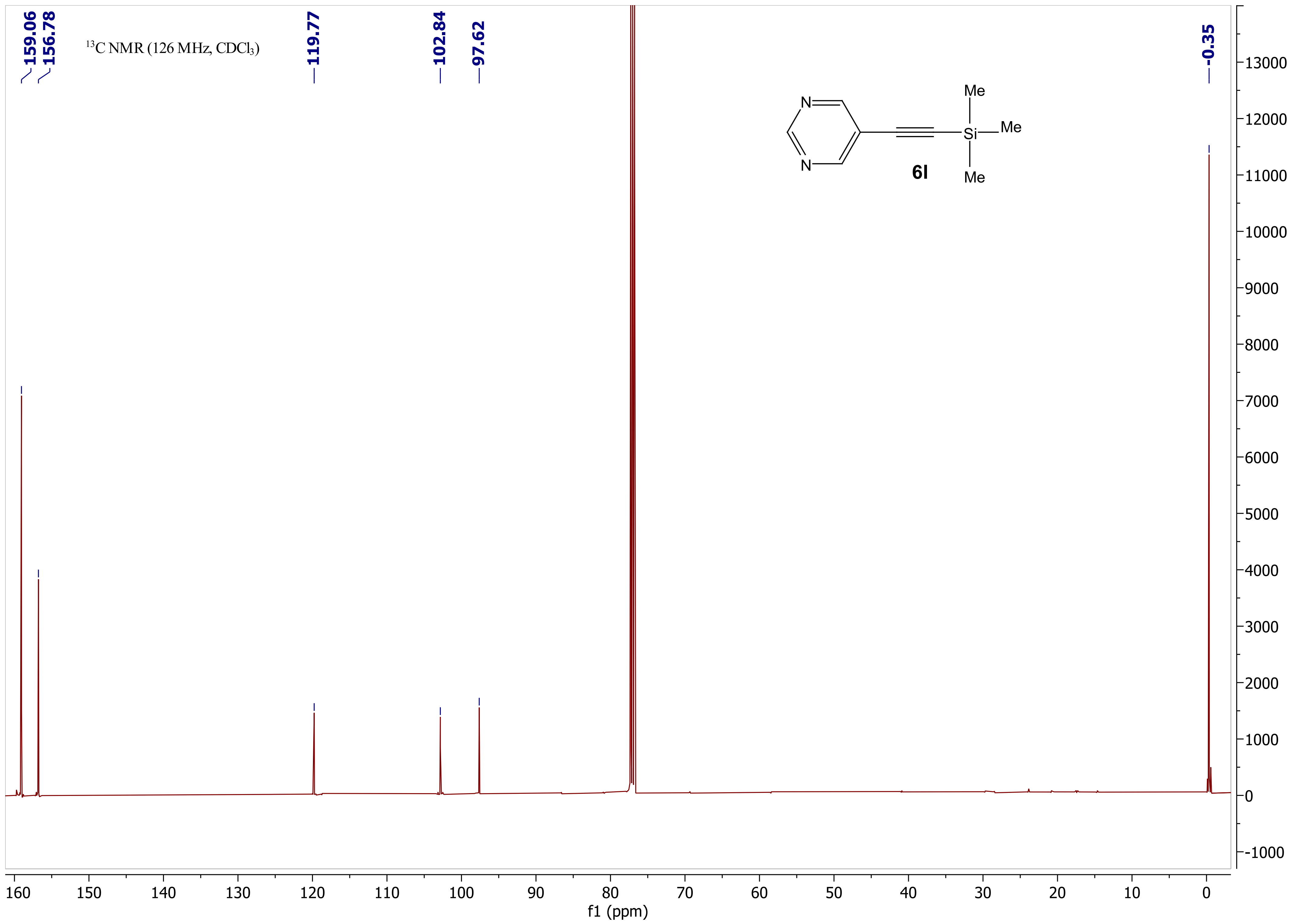

159.06
156.78
^{13}C NMR (126 MHz, $CDCl_3$)
119.77
102.84
97.62
-0.35
N
N
Me
Me
Si
Me
6l
f1 (ppm)

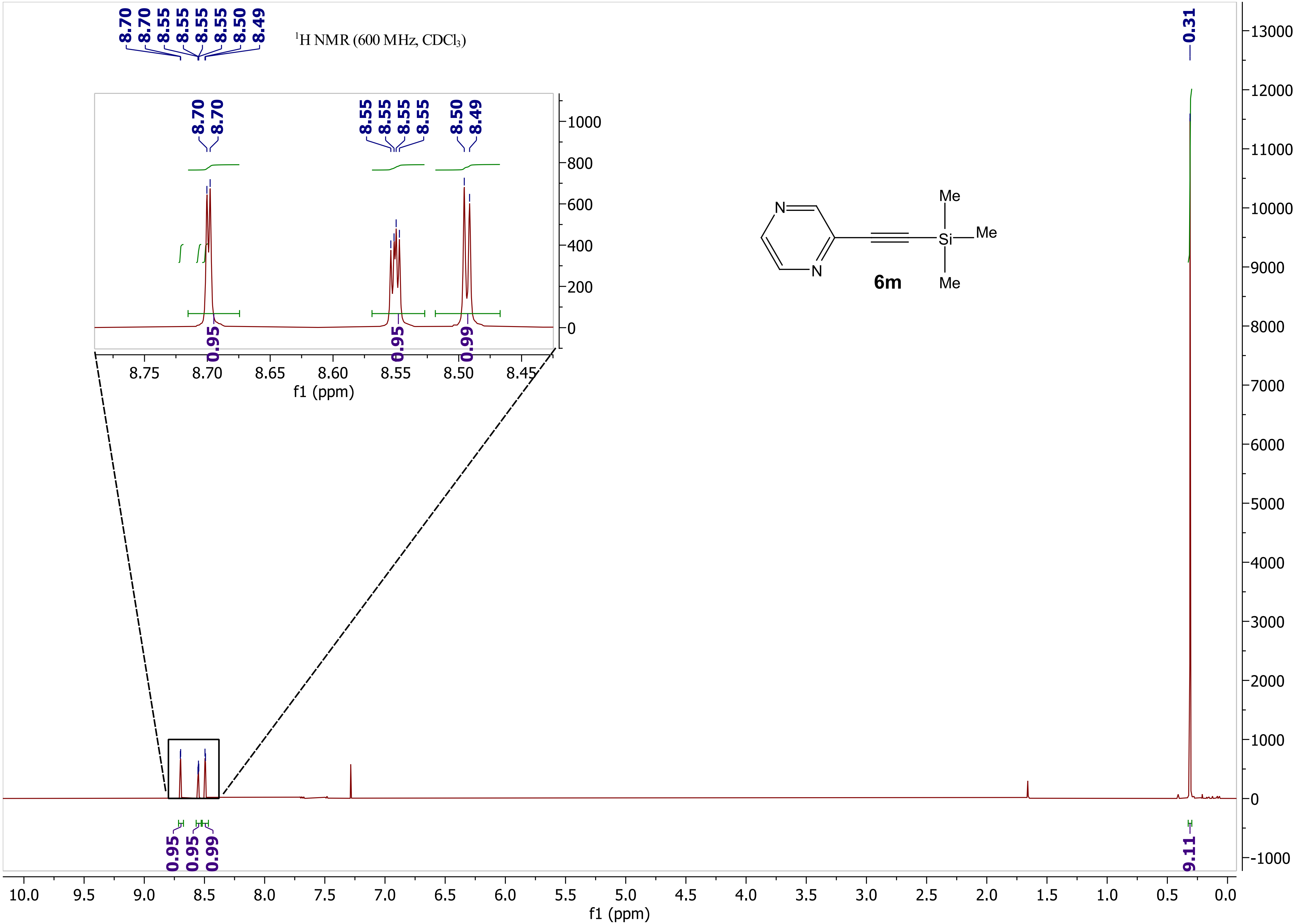

¹H NMR (600 MHz, CDCl3)
8.70
8.70
8.55
8.55
8.55
8.55
8.50
8.49
0.31
6m
Me
Me
Me
Si
N
N
0.95
0.95
0.99
9.11
f1 (ppm)

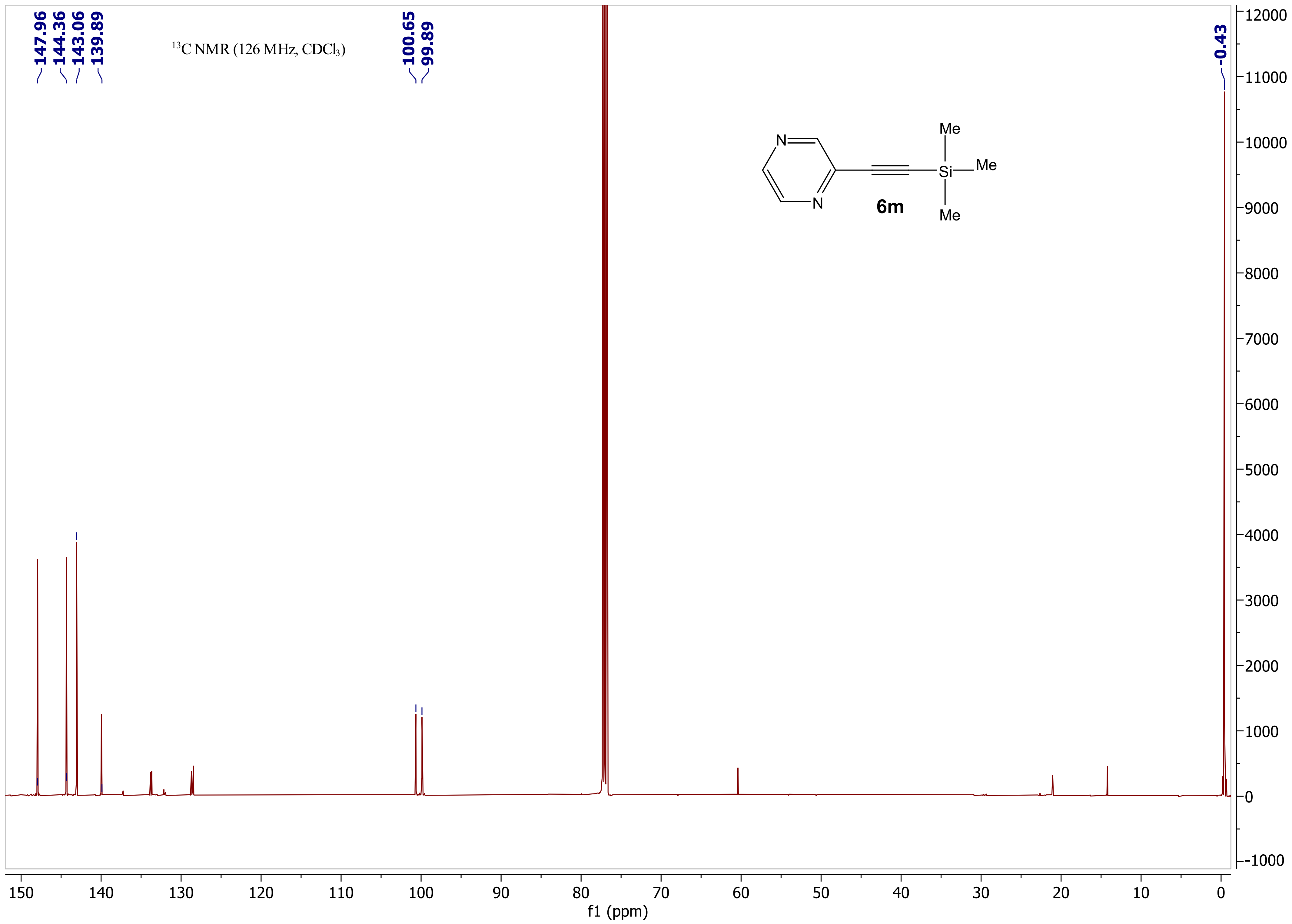
147.96
144.36
143.06
139.89
^{13}C NMR (126 MHz, CDCl$_3$)
100.65
99.89
-0.43
Me
N
Me
Si
N
6m
Me
150
140
130
120
110
100
90
80
70
60
50
40
30
20
10
0
f1 (ppm)
12000
11000
10000
9000
8000
7000
6000
5000
4000
3000
2000
1000
0
-1000

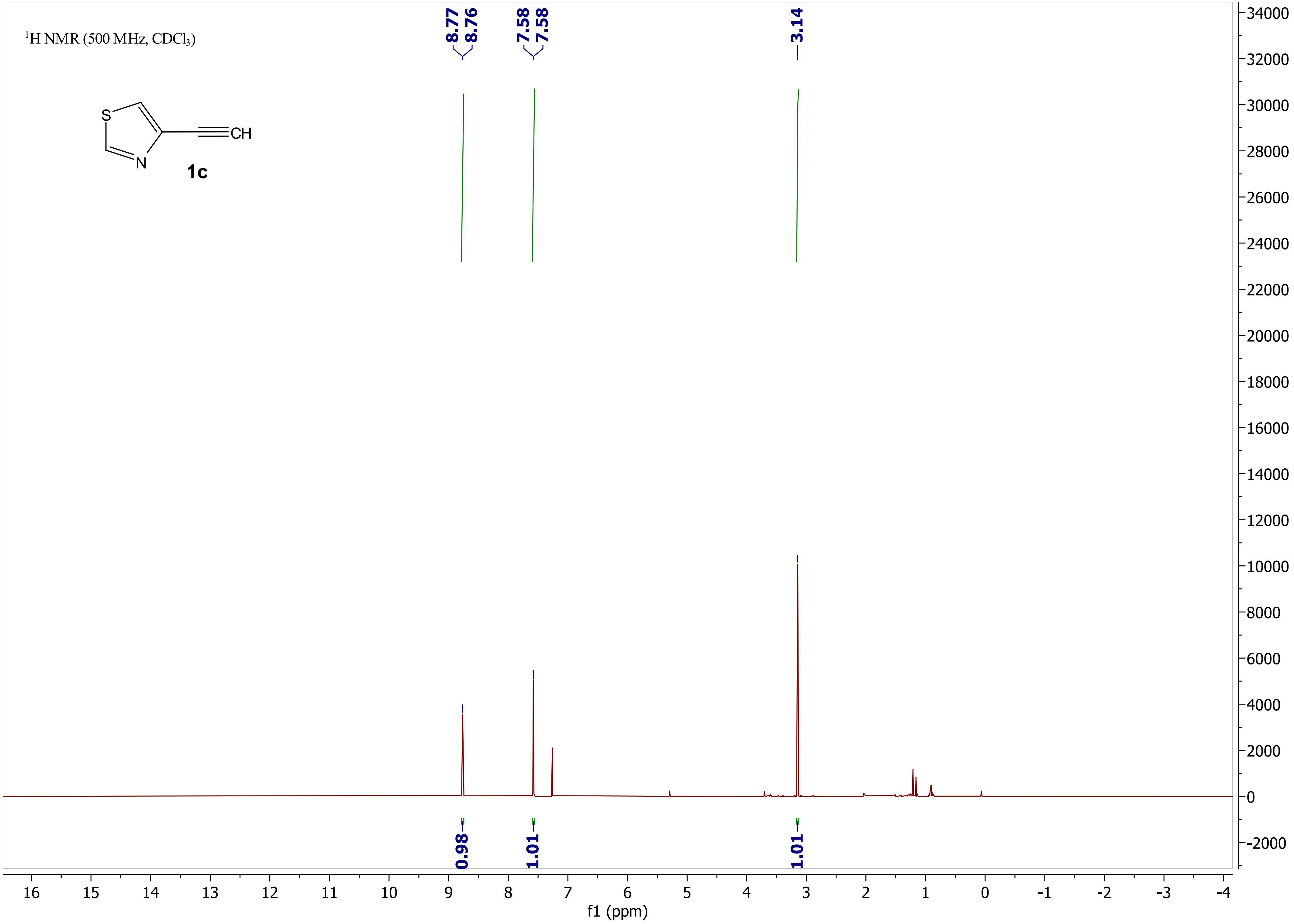

1H NMR (500 MHz, CDCl3)
S
N
CH
1c
8.77
8.76
7.58
7.58
3.14
0.98
1.01
1.01
f1 (ppm)

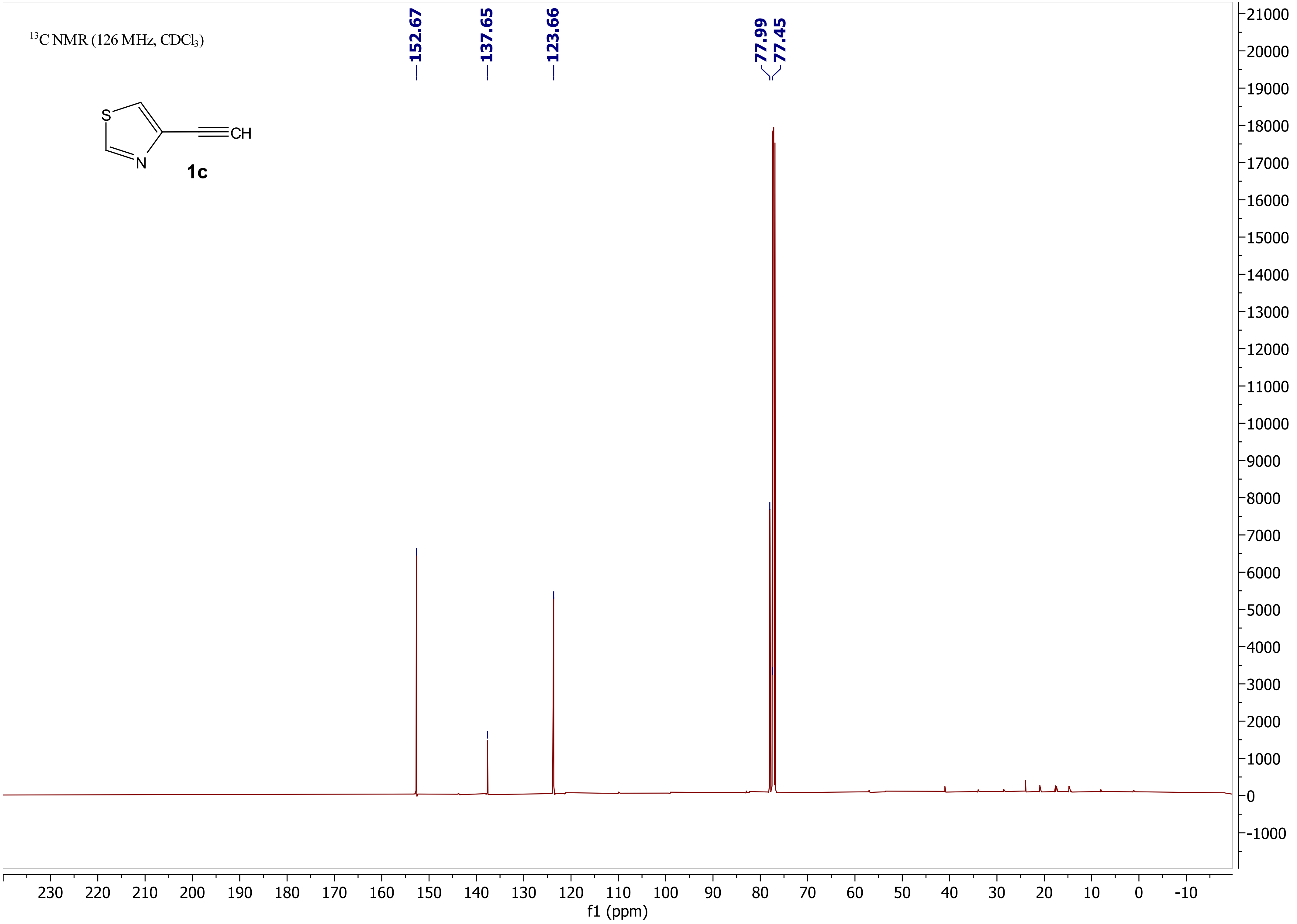
13C NMR (126 MHz, CDCl3)
S
N
CH
1c
152.67
137.65
123.66
77.99
77.45
f1 (ppm)

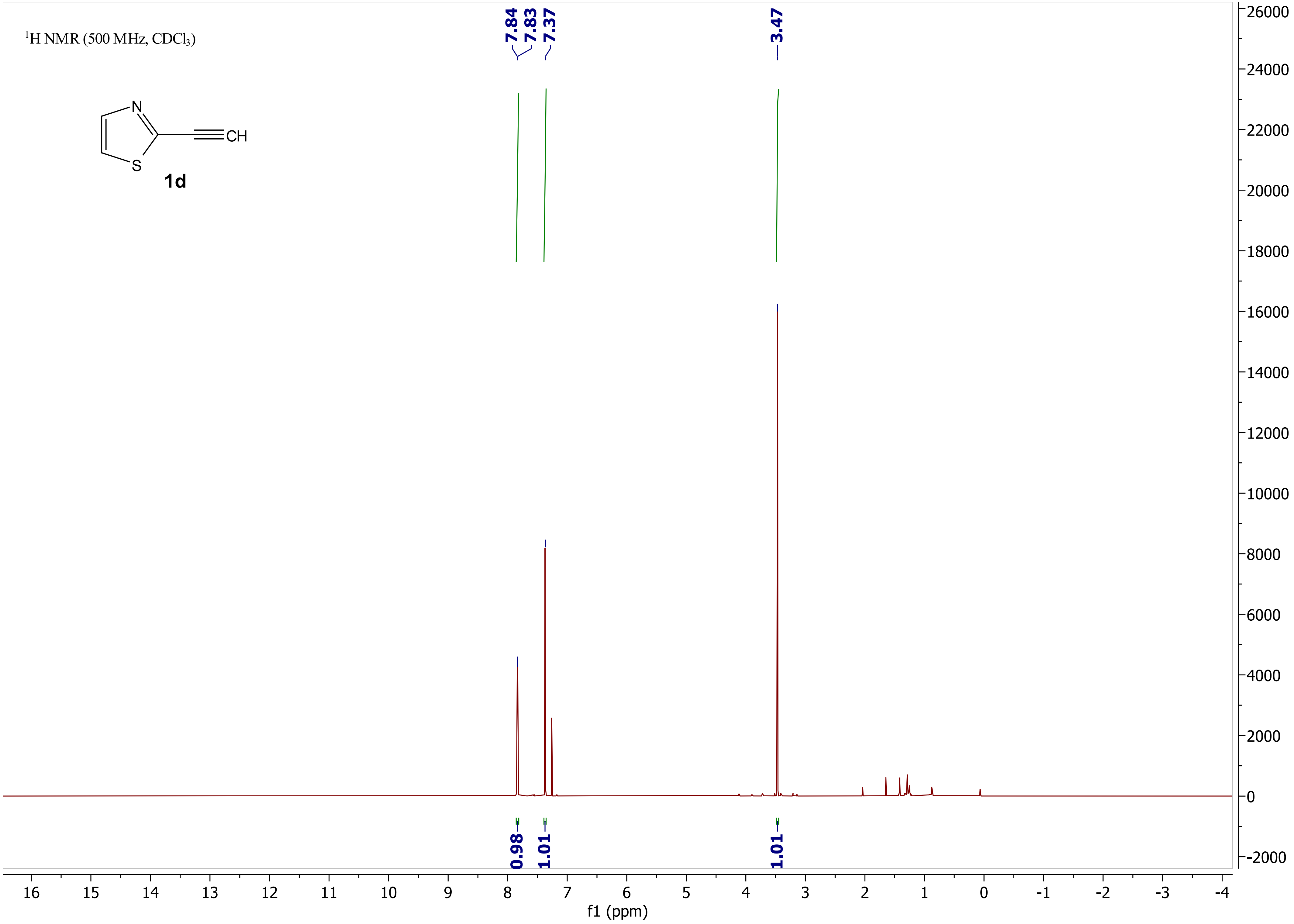

1H NMR (500 MHz, CDCl3)
N
S
CH
1d
7.84
7.83
7.37
3.47
0.98
1.01
1.01
f1 (ppm)

^{13}C NMR (126 MHz, $CDCl_3$)

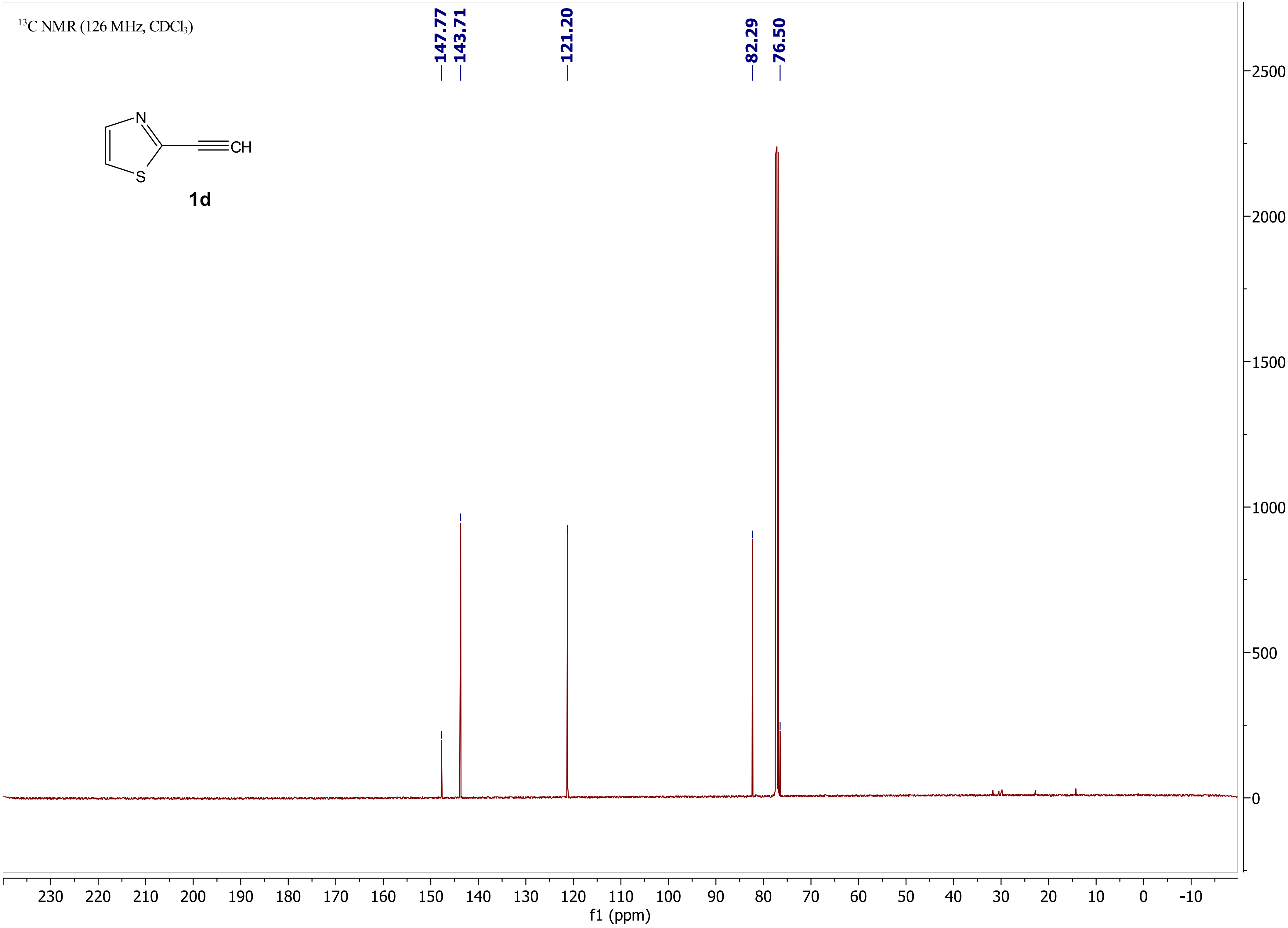

1d

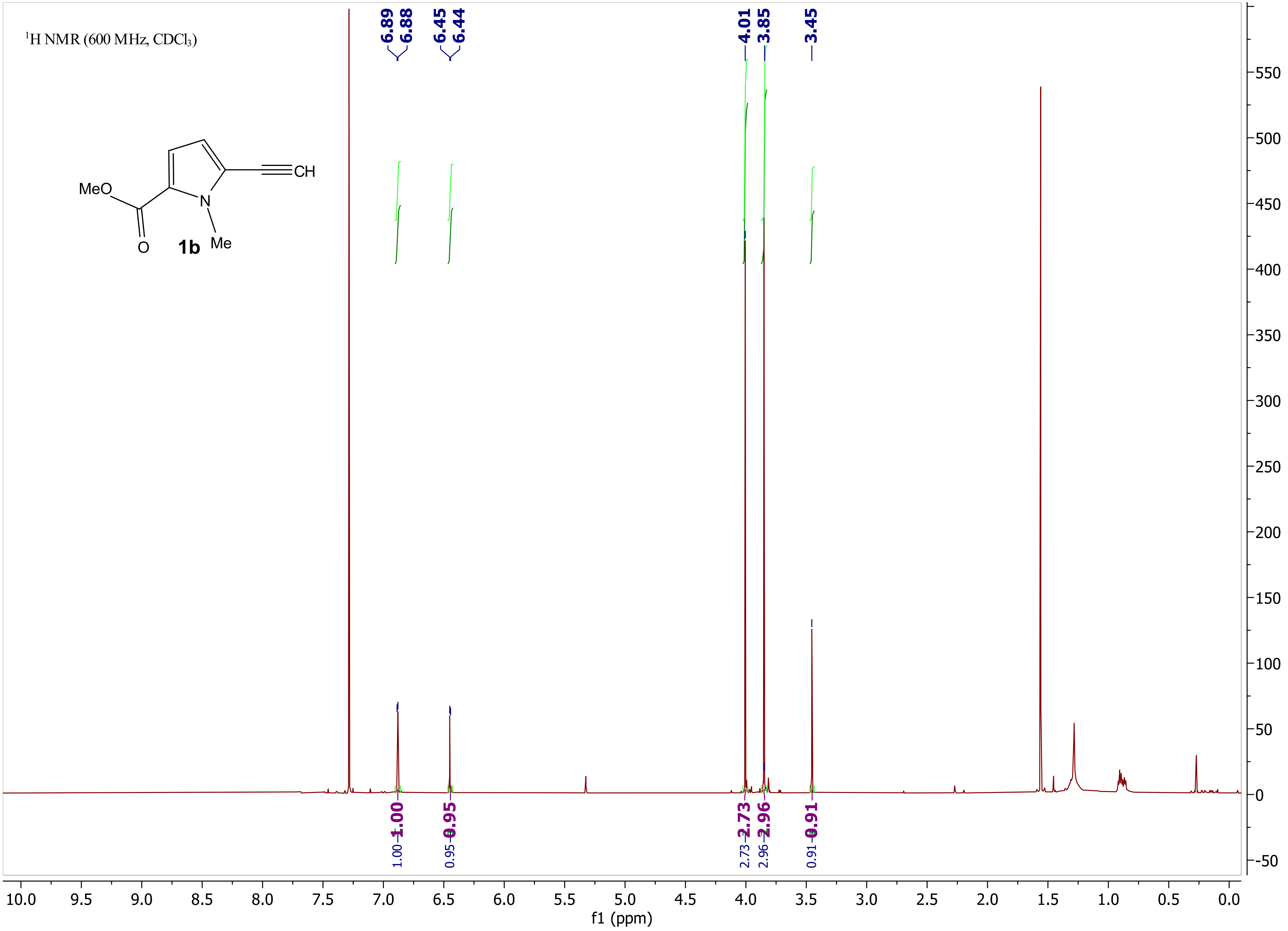

1H NMR (600 MHz, CDCl3)
MeO
N
CH
O
1b
Me
6.89
6.88
6.45
6.44
4.01
3.85
3.45
1.00
0.95
2.73
2.96
0.91
f1 (ppm)

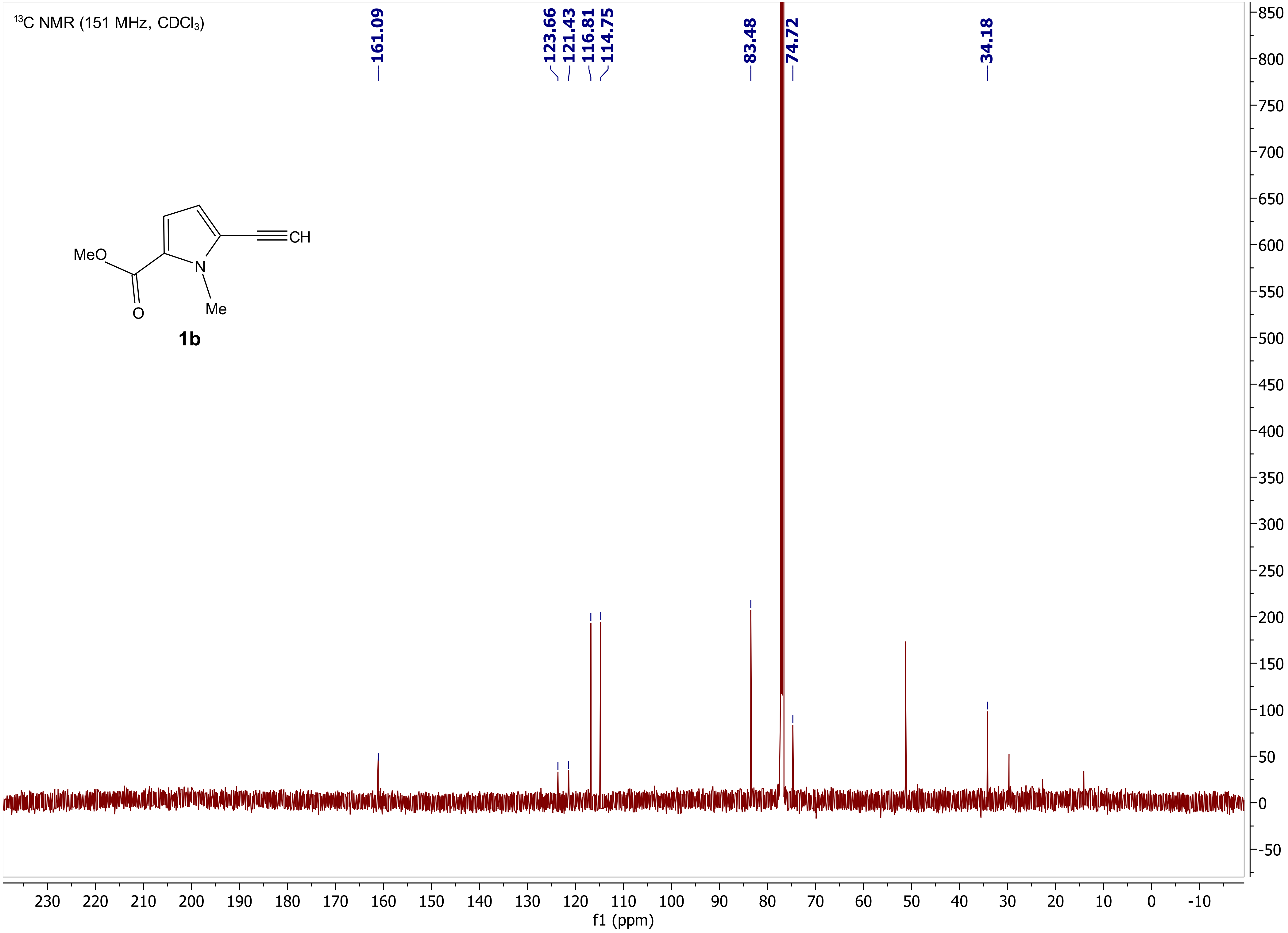

^{13}C NMR (151 MHz, CDCl$_3$)
161.09
123.66
121.43
116.81
114.75
83.48
74.72
34.18
MeO
CH
N
O
Me
1b
f1 (ppm)

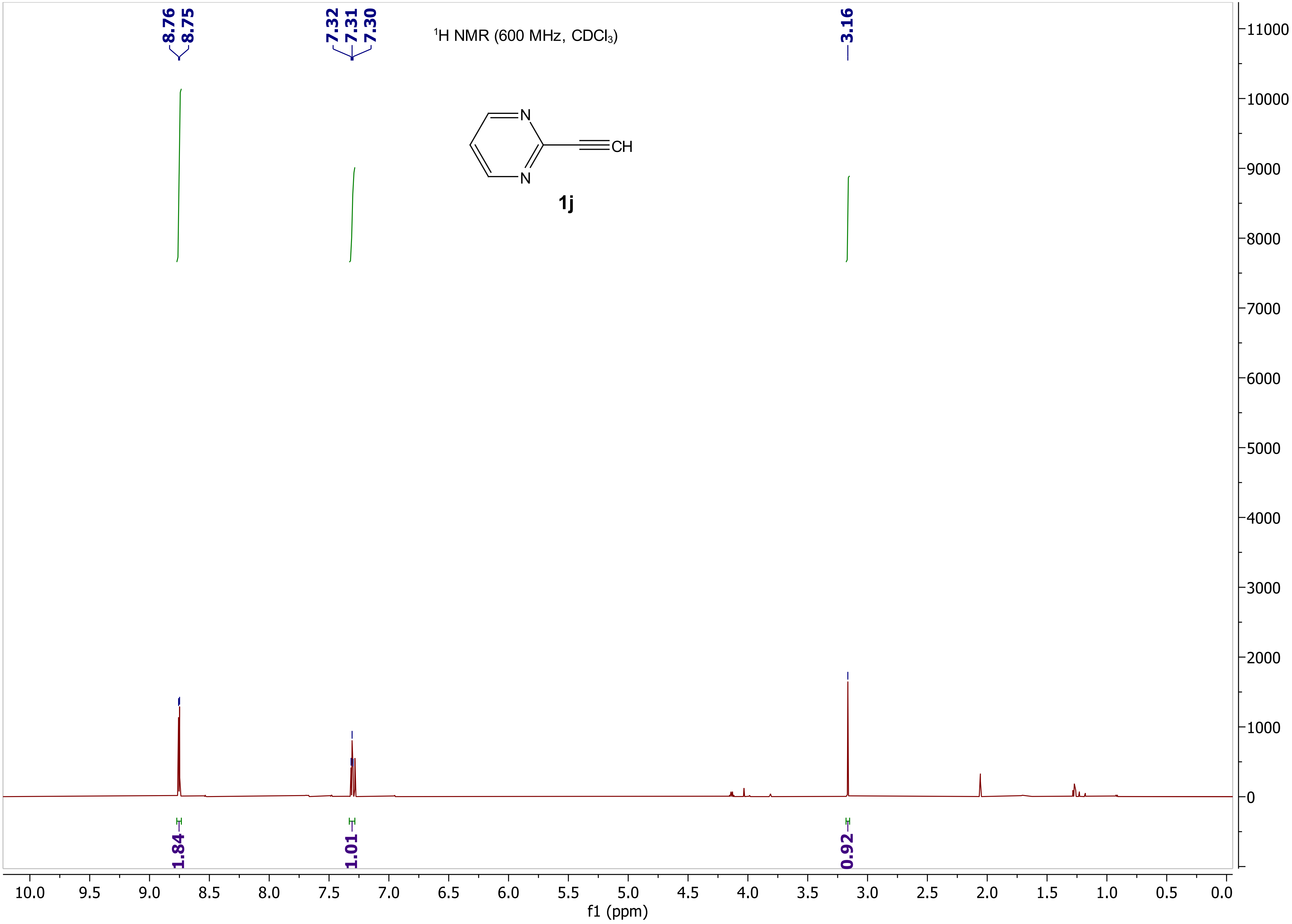

8.76
8.75
7.32
7.31
7.30
3.16
1H NMR (600 MHz, CDCl3)
N
N
CH
1j
1.84
1.01
0.92
f1 (ppm)

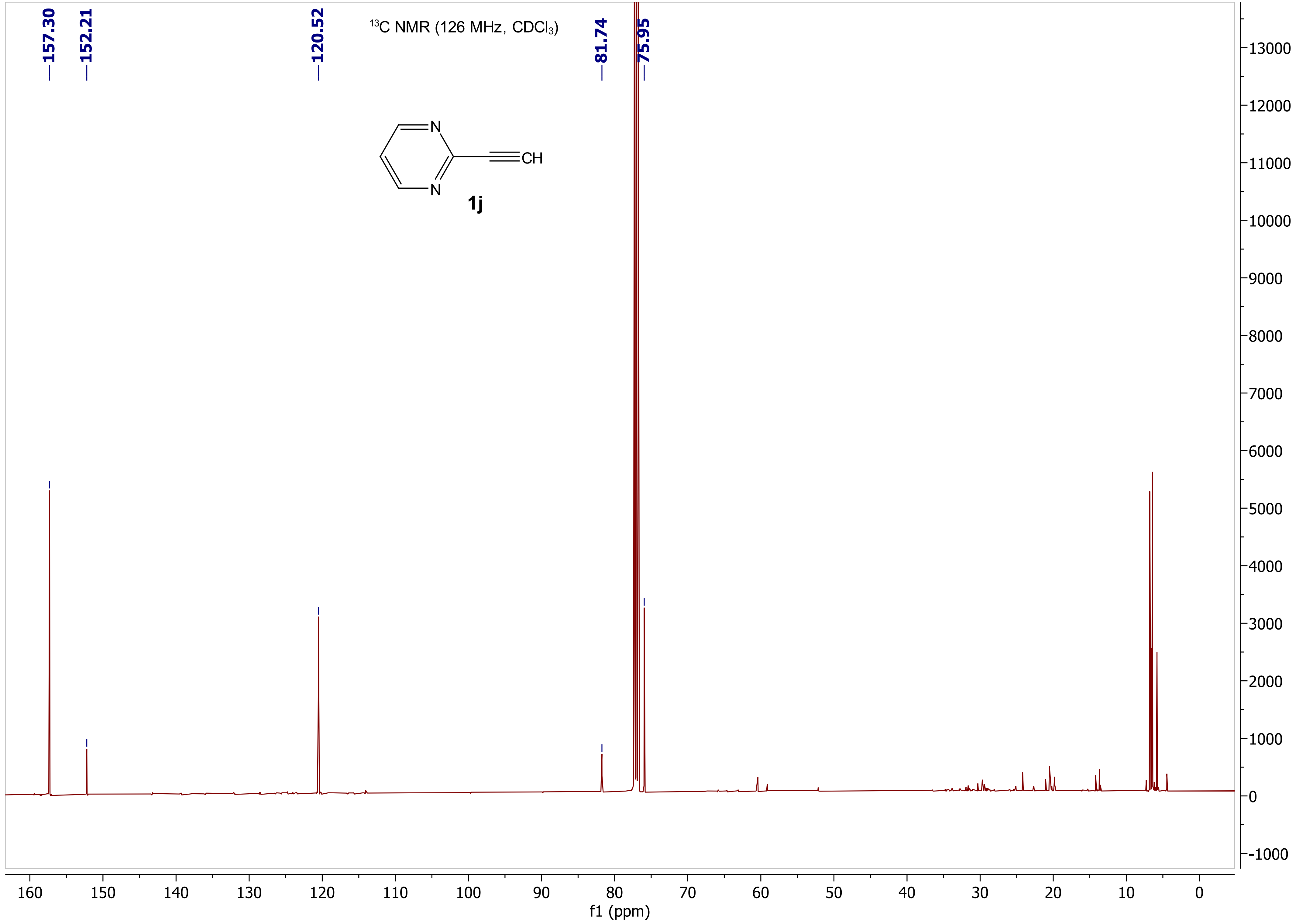

157.30
152.21
120.52
81.74
75.95
^{13}C NMR (126 MHz, CDCl$_3$)
N
N
CH
1j
f1 (ppm)

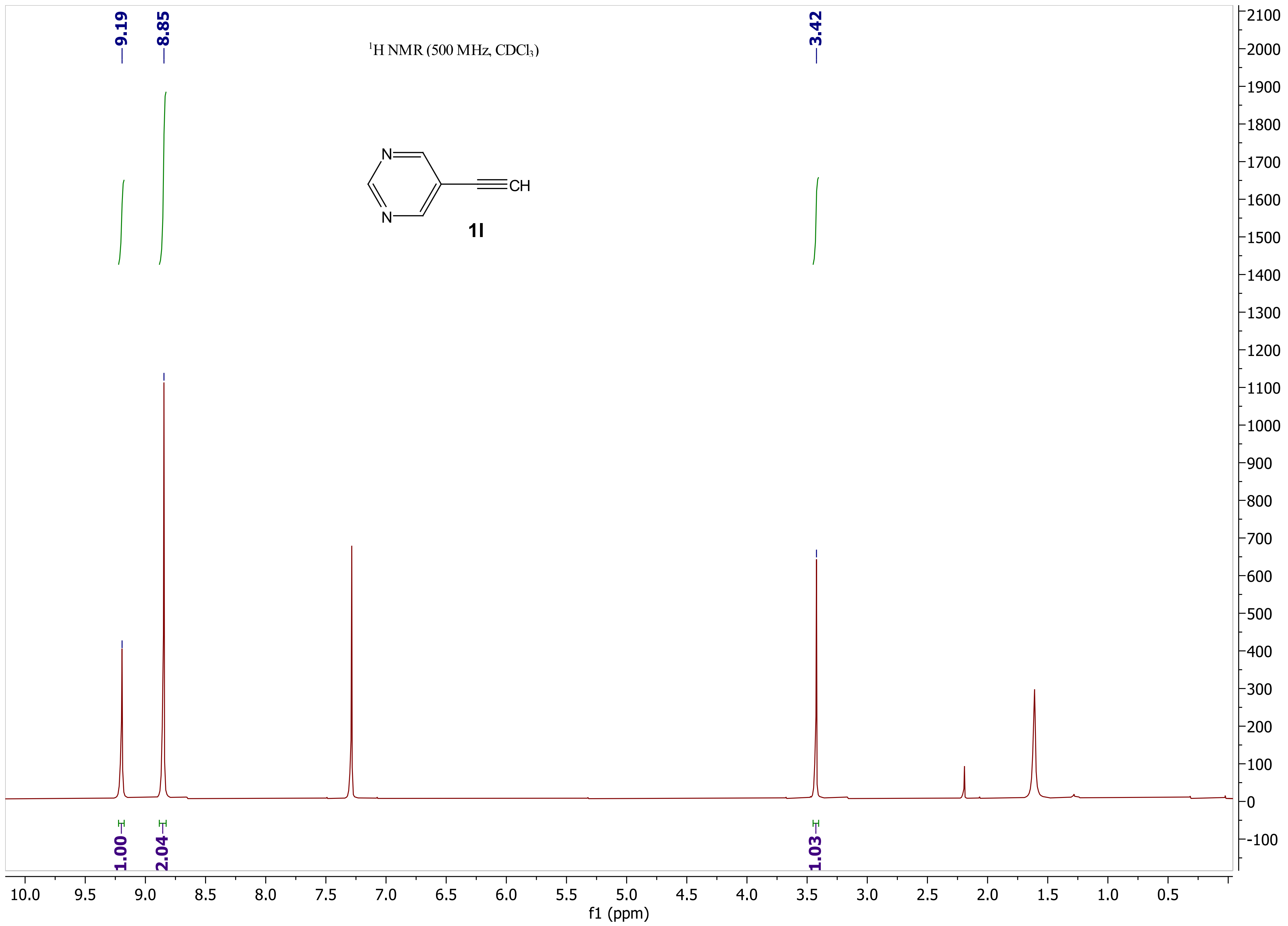

9.19
8.85
3.42
1H NMR (500 MHz, CDCl3)
N
N
CH
1l
1.00
2.04
1.03
f1 (ppm)

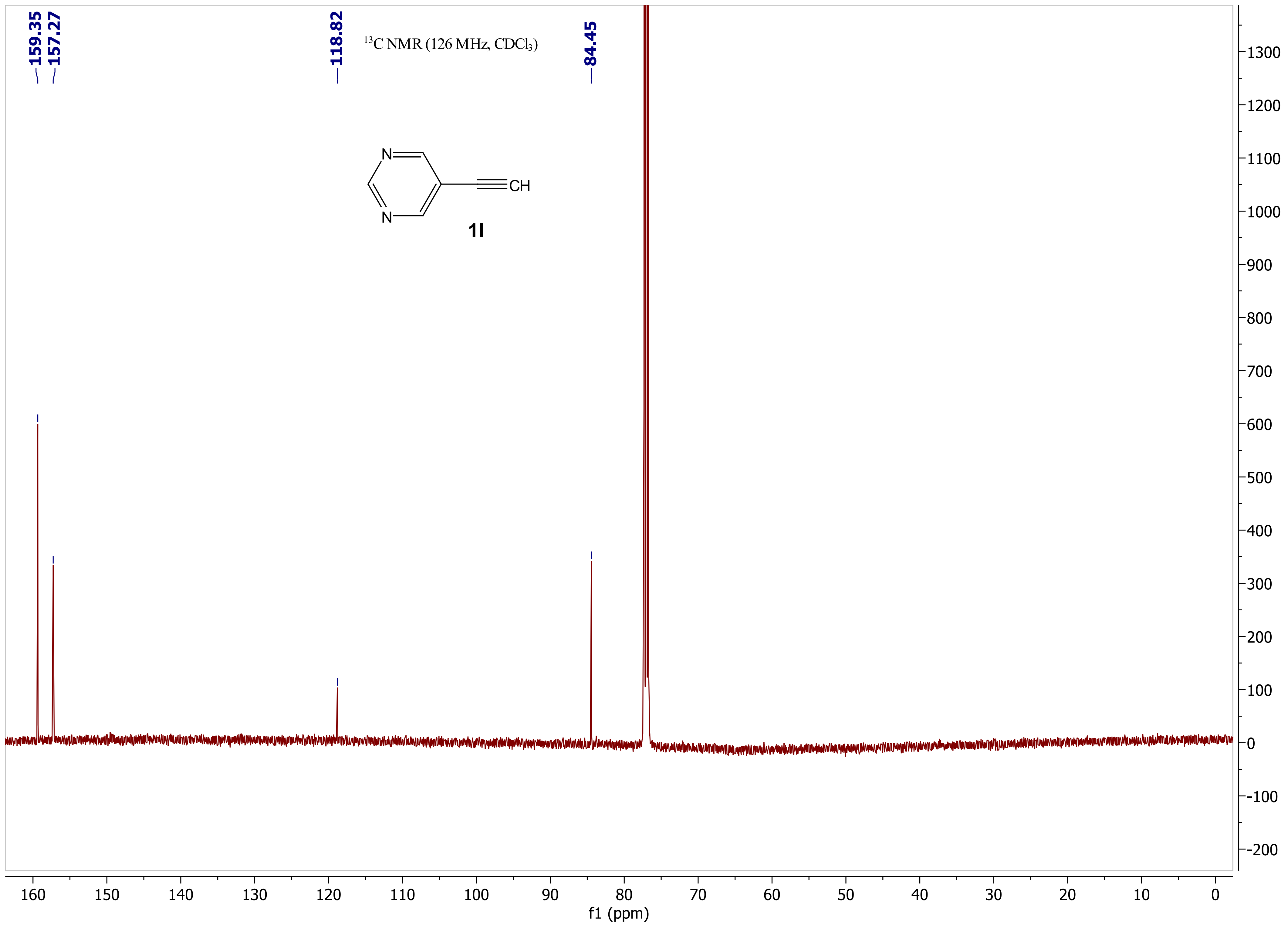
159.35
157.27
118.82
84.45
13C NMR (126 MHz, CDCl3)
N
N
CH
1l
f1 (ppm)

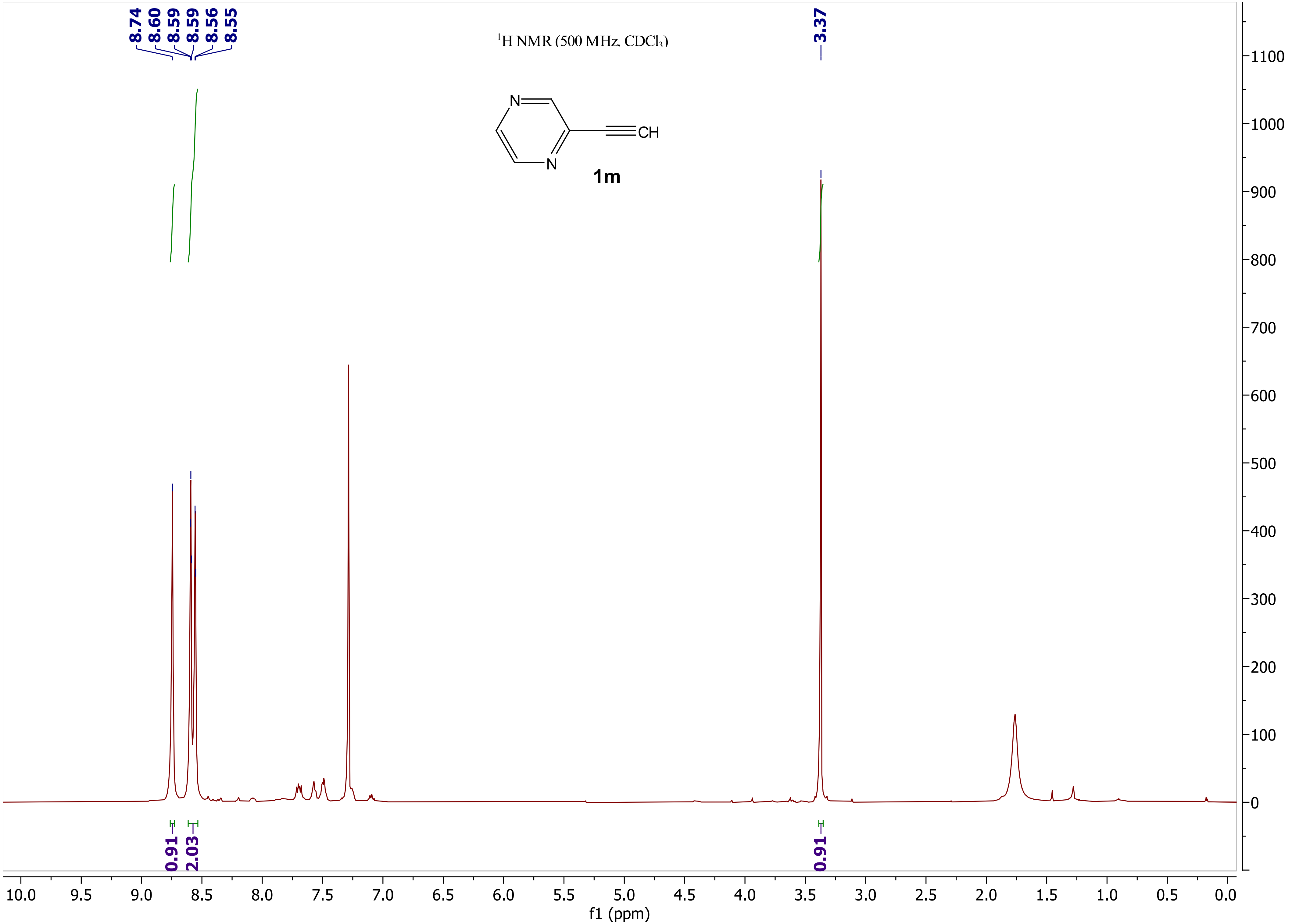

8.74
8.60
8.59
8.59
8.56
8.55
3.37
¹H NMR (500 MHz, CDCl₃)
N
N
CH
1m
0.91
2.03
0.91
f1 (ppm)

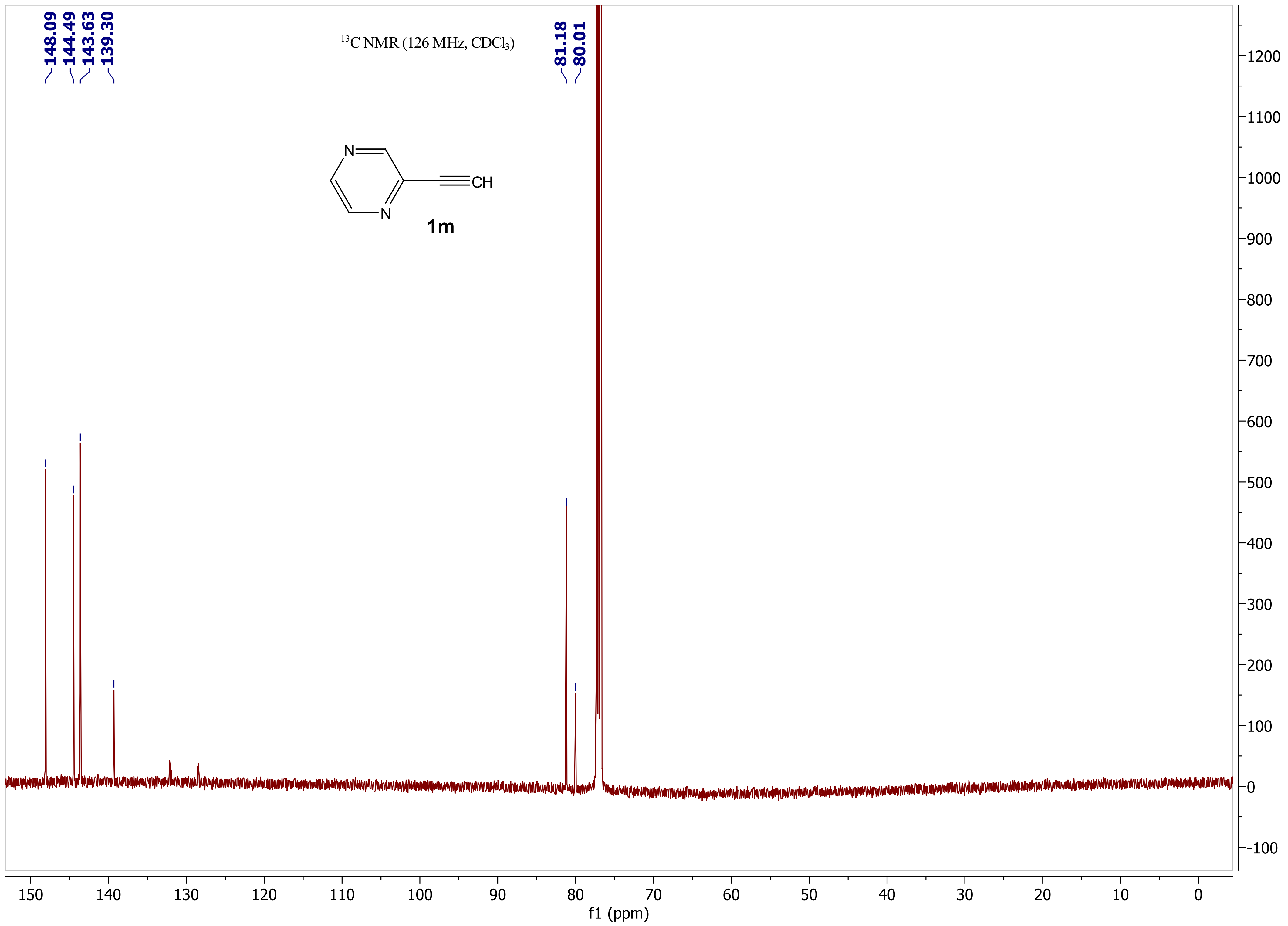
148.09
144.49
143.63
139.30
13C NMR (126 MHz, CDCl3)
81.18
80.01
N
N
CH
1m
1200
1100
1000
900
800
700
600
500
400
300
200
100
0
-100
150
140
130
120
110
100
90
80
70
60
50
40
30
20
10
0
f1 (ppm)

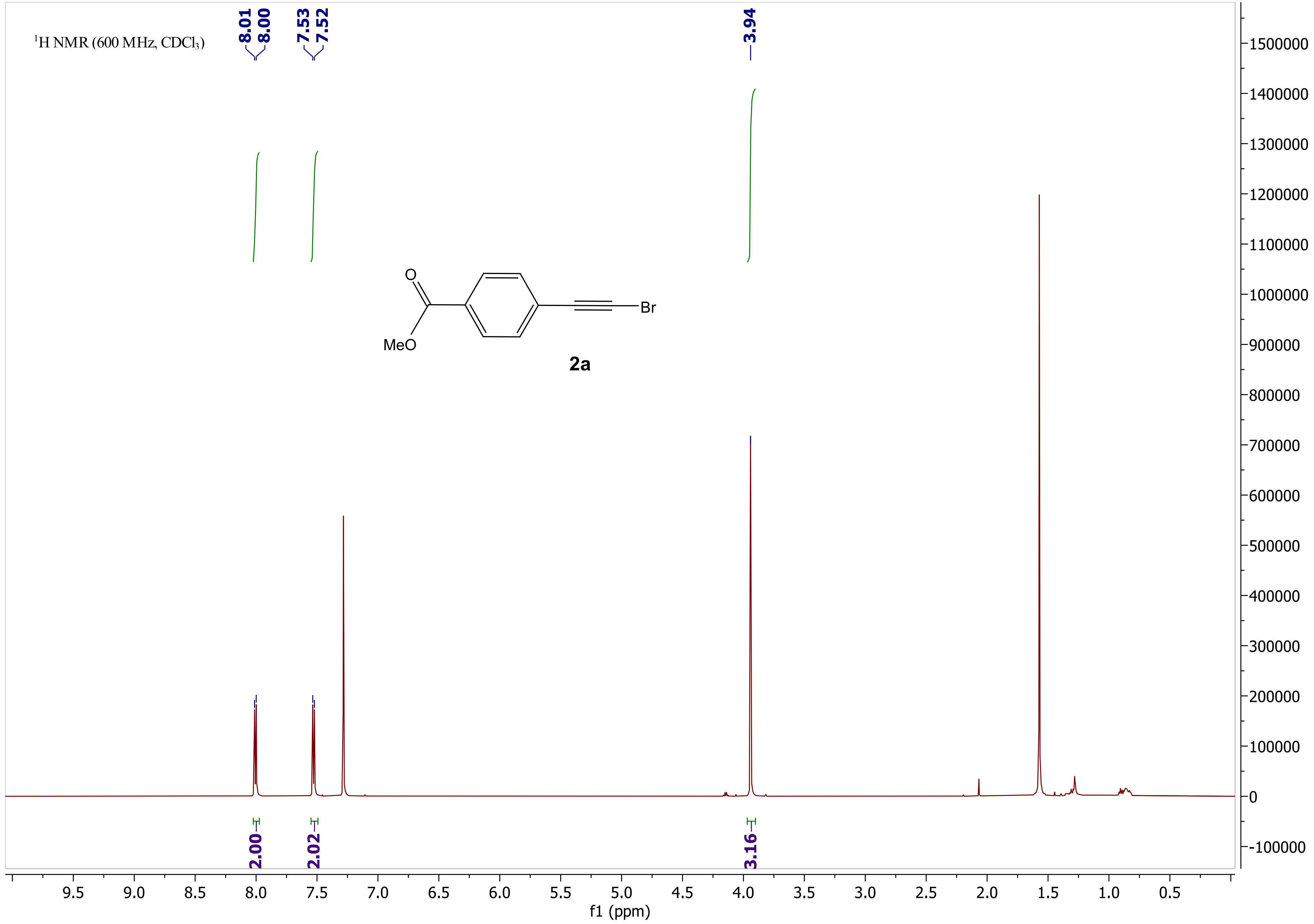

1H NMR (600 MHz, CDCl3)
8.01
8.00
7.53
7.52
3.94
MeO
O
Br
2a
2.00
2.02
3.16
f1 (ppm)

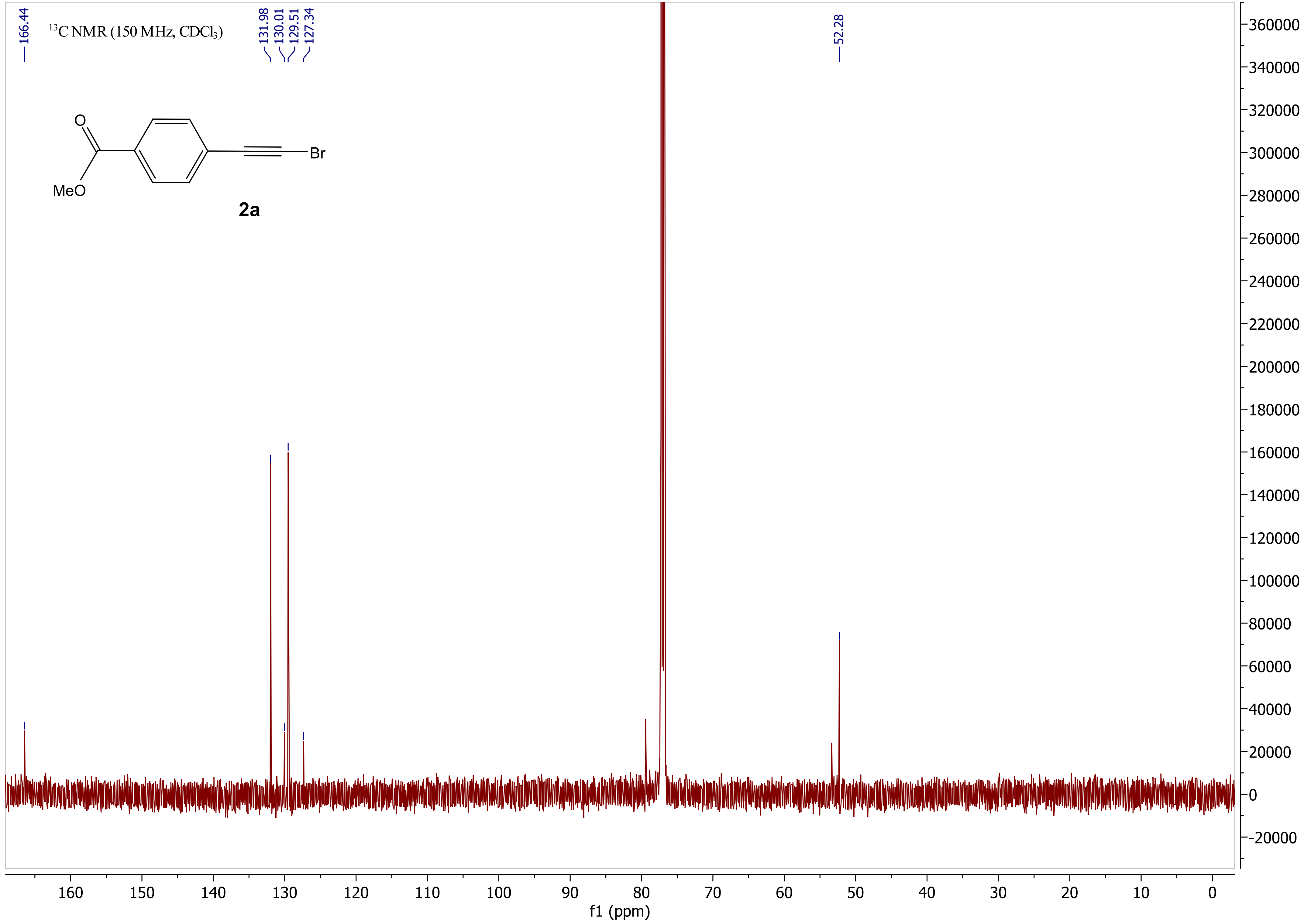

13C NMR (150 MHz, CDCl3)
166.44
131.98
130.01
129.51
127.34
52.28
O
MeO
Br
2a
f1 (ppm)

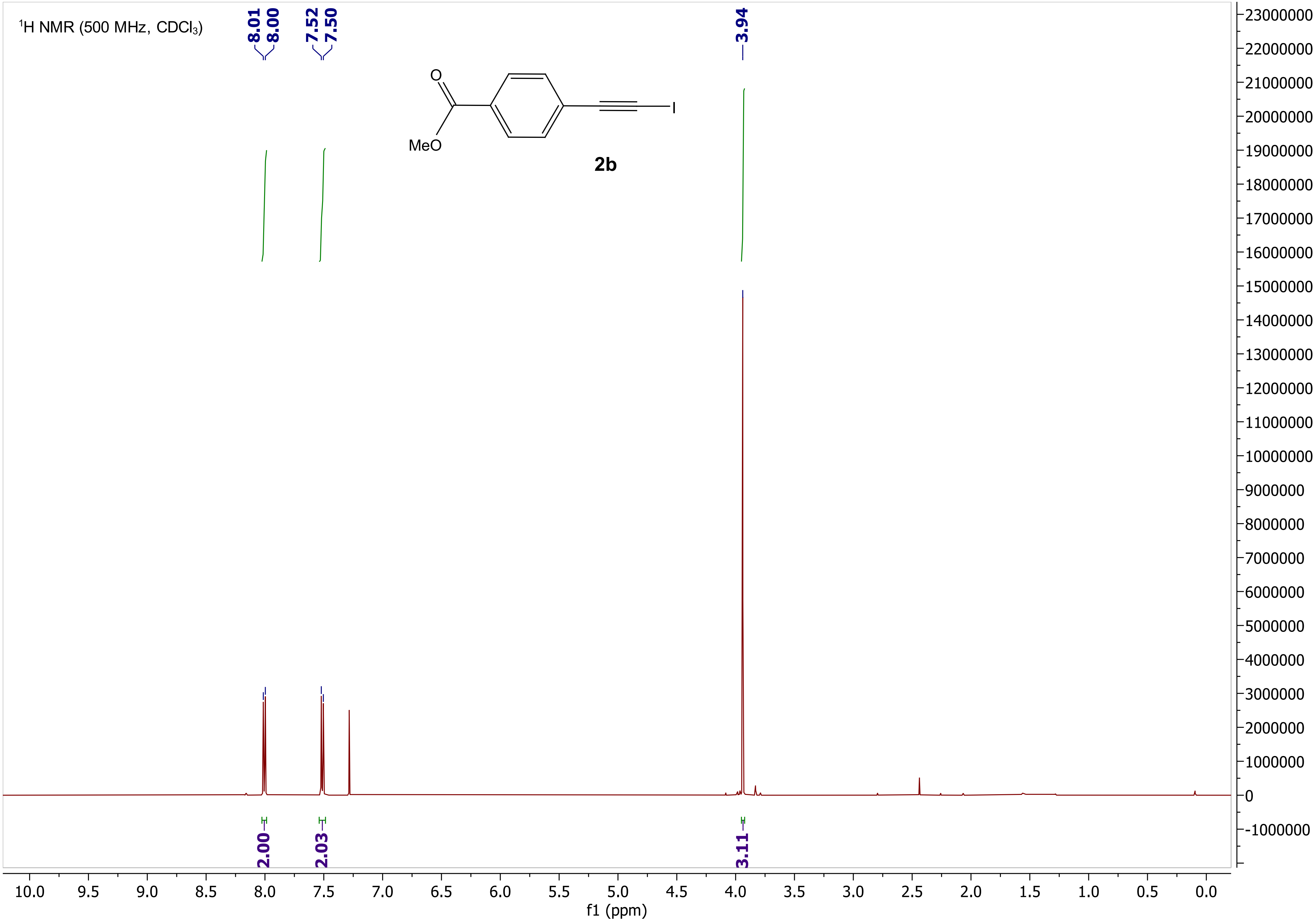

1H NMR (500 MHz, CDCl3)
8.01
8.00
7.52
7.50
3.94
O
MeO
I
2b
2.00
2.03
3.11
f1 (ppm)

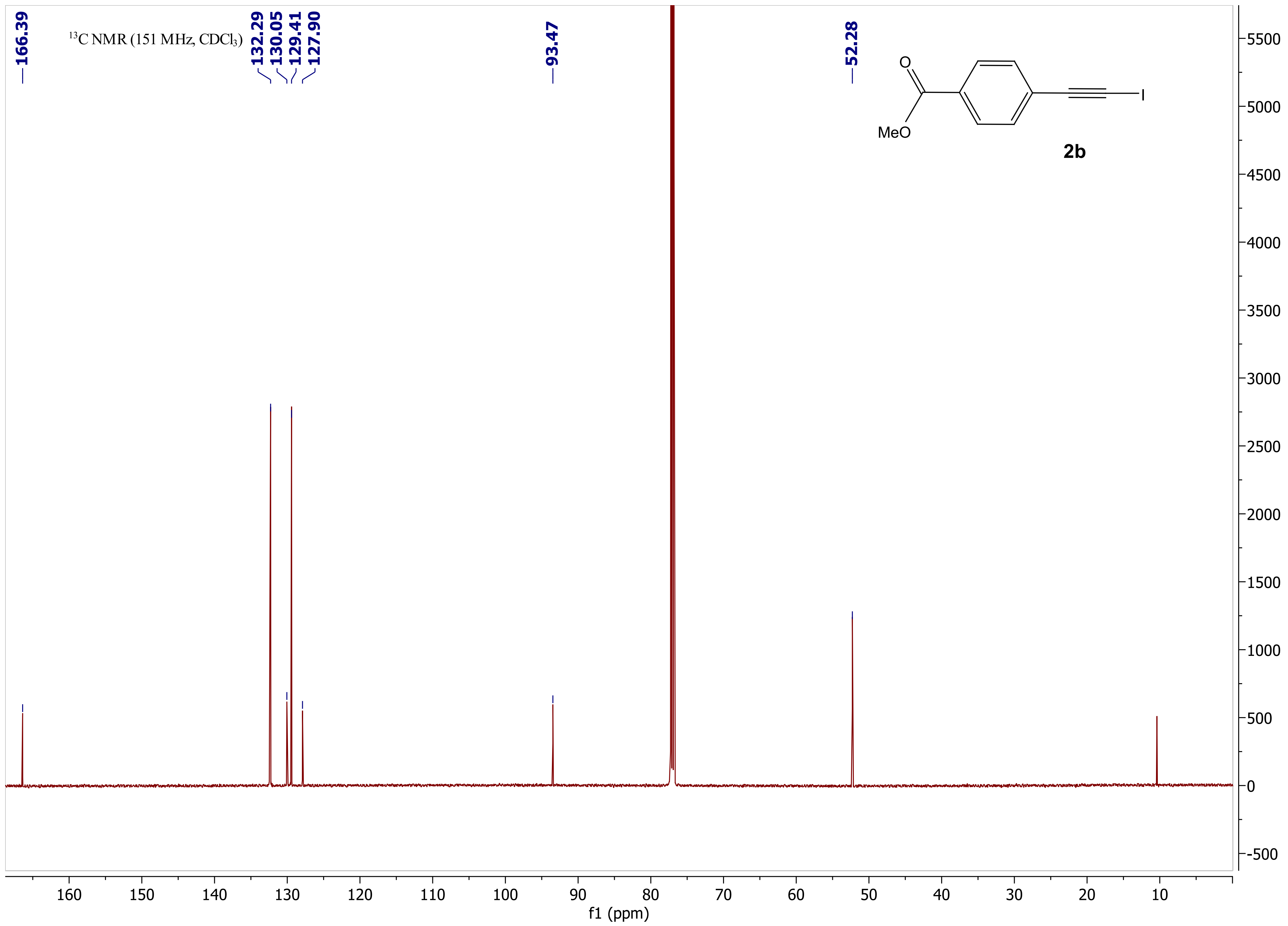

^{13}C NMR (151 MHz, CDCl$_3$)
166.39
132.29
130.05
129.41
127.90
93.47
52.28
O
MeO
I
2b
f1 (ppm)

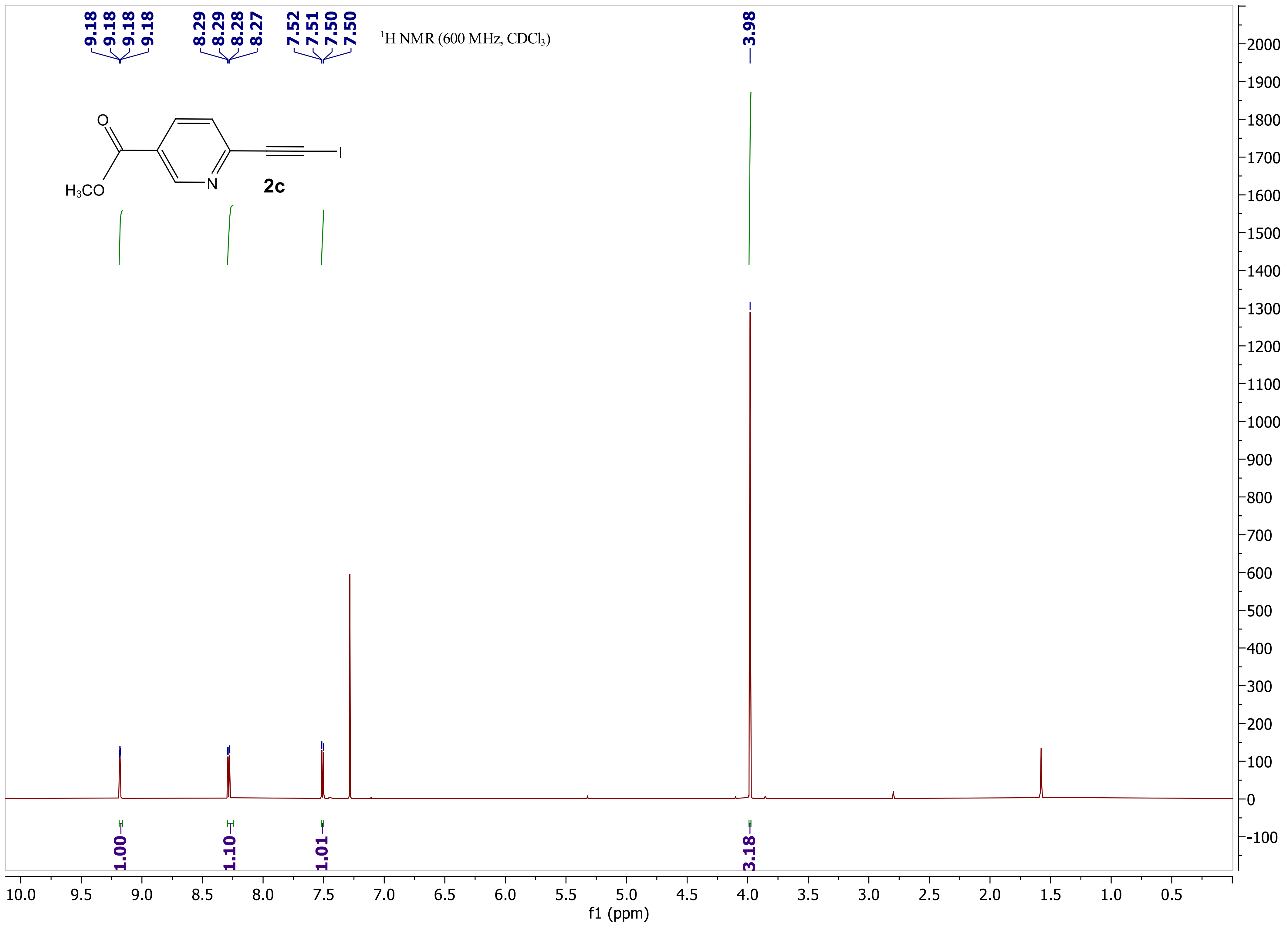

^{1}H NMR (600 MHz, $CDCl_3$)
9.18 9.18 9.18 9.18
8.29 8.29 8.28 8.27
7.52 7.51 7.50 7.50
3.98
O
H_3CO
N
I
2c
1.00
1.10
1.01
3.18
f1 (ppm)

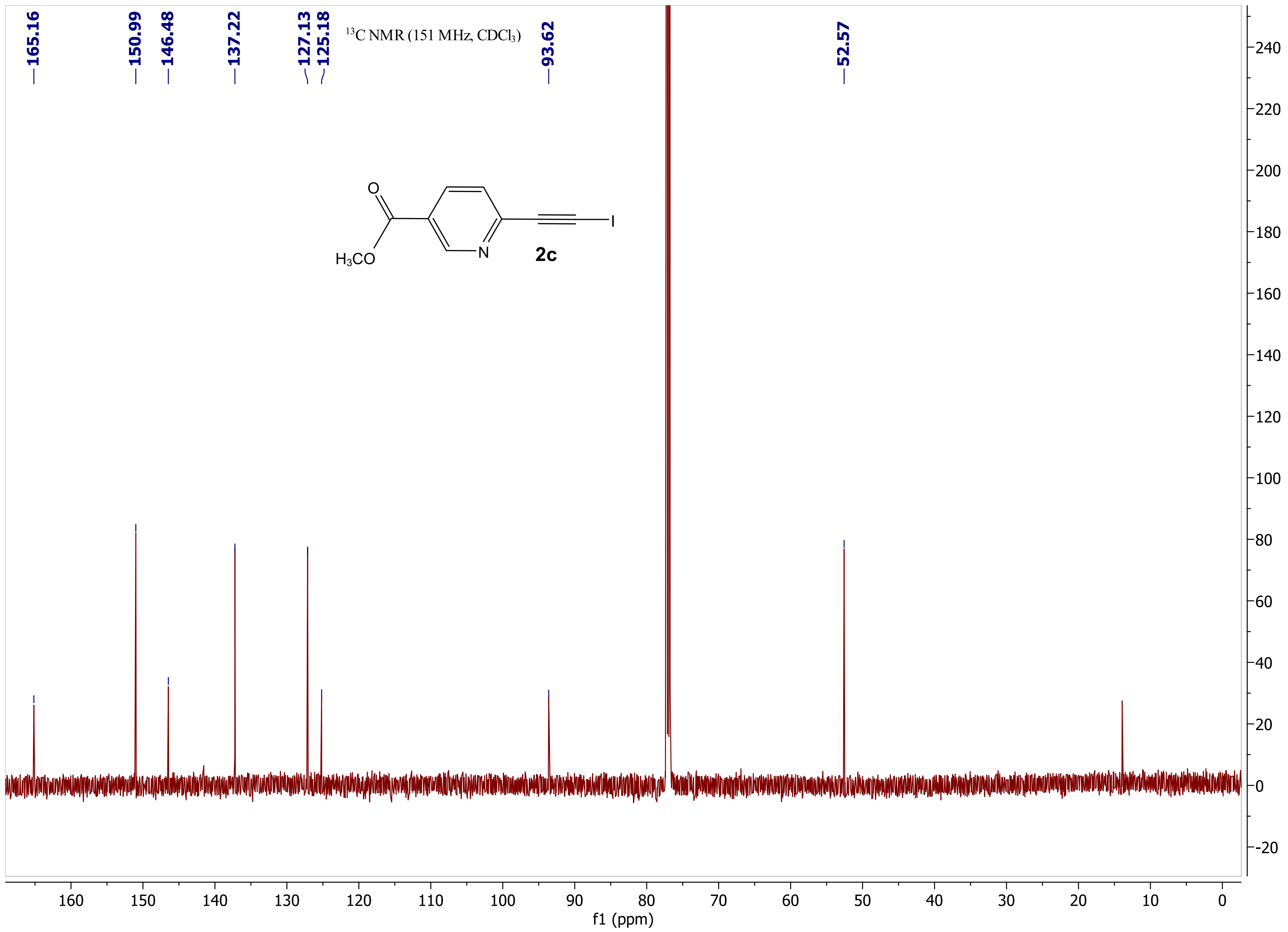

165.16
150.99
146.48
137.22
127.13
125.18
93.62
52.57
^{13}C NMR (151 MHz, CDCl$_3$)
O
H$_3$CO
N
I
2c
f1 (ppm)

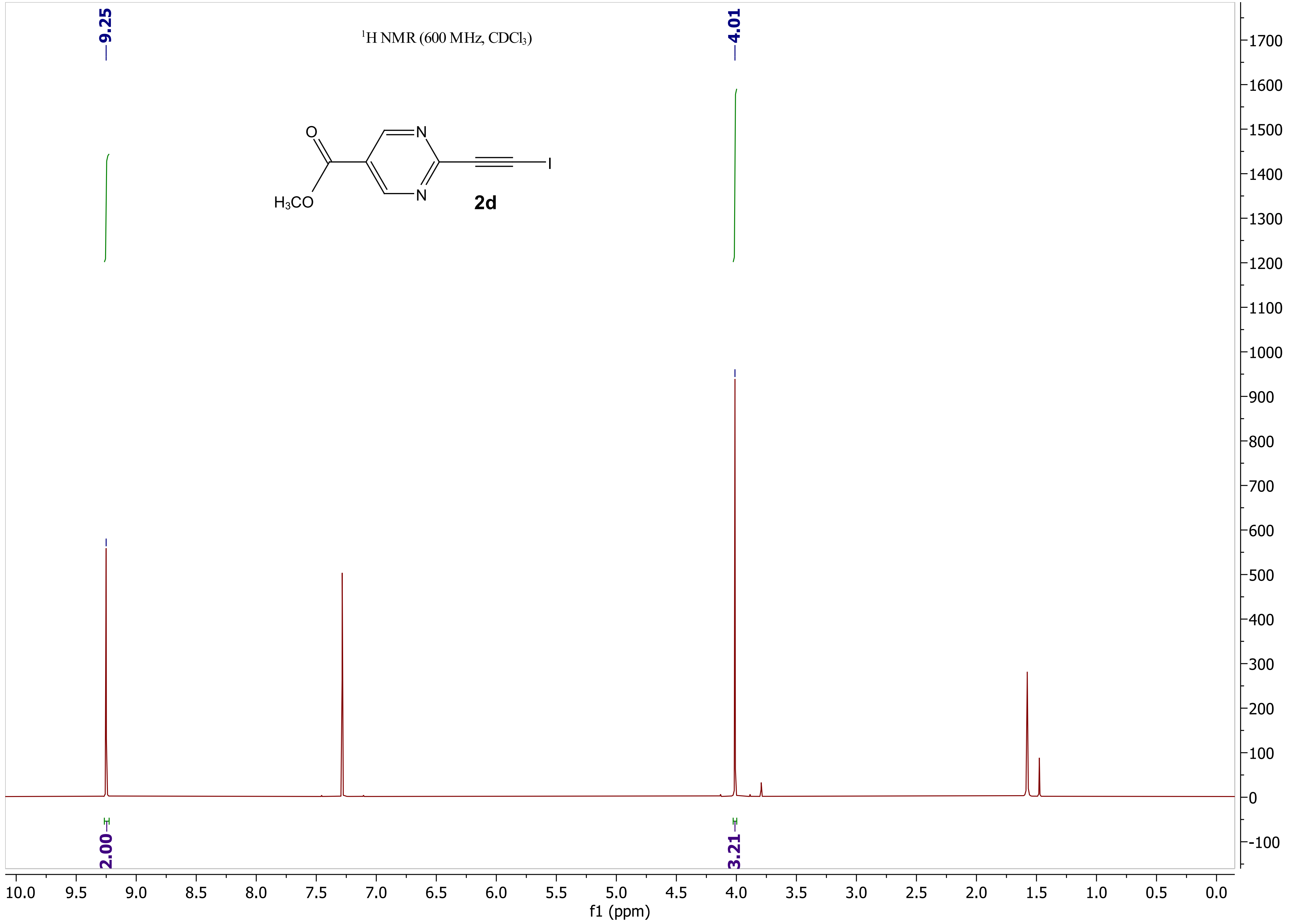

^{1}H NMR (600 MHz, $CDCl_3$)
2d
H_3CO
O
N
N
I
9.25
4.01
2.00
3.21
f1 (ppm)

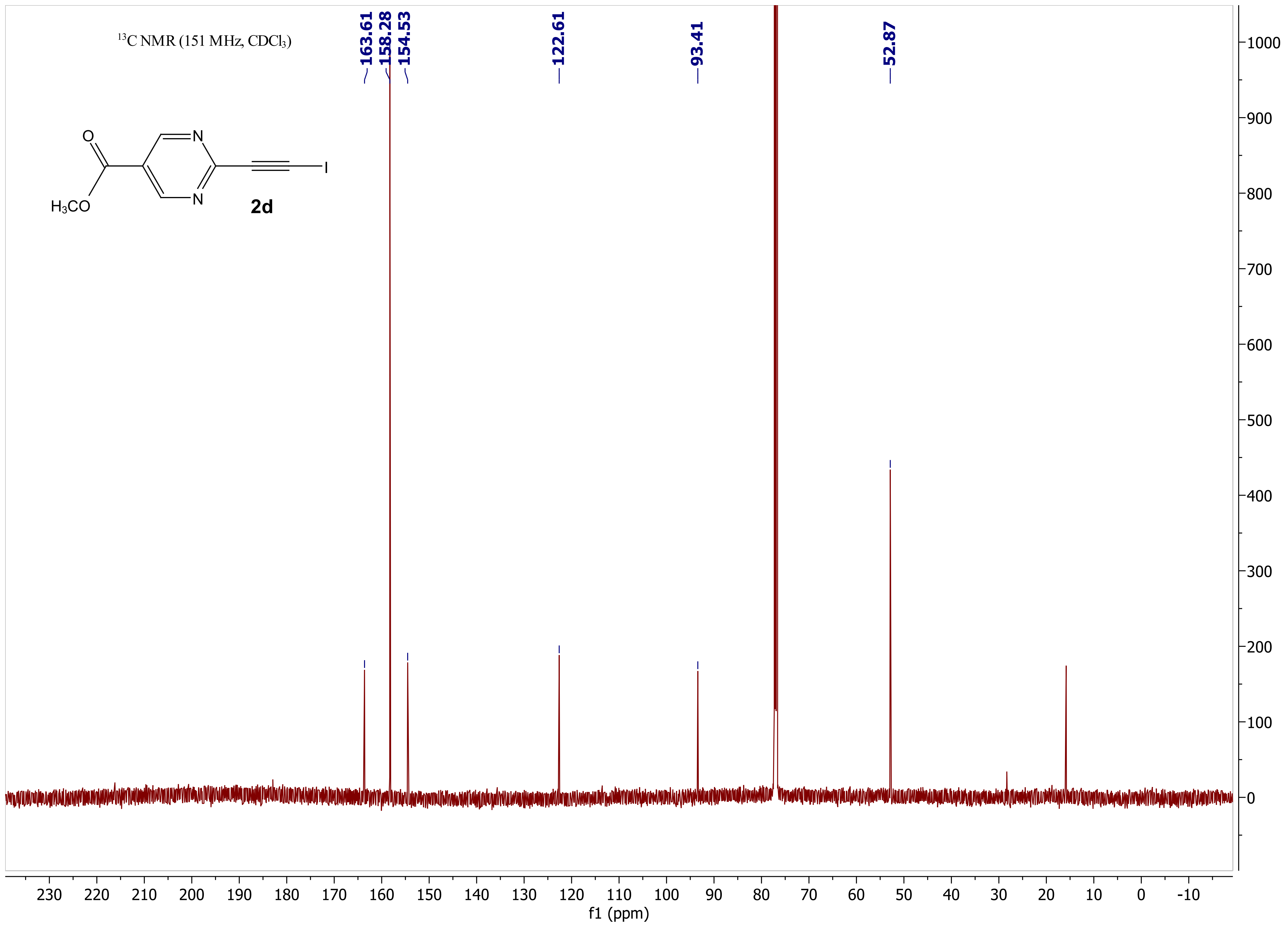

^{13}C NMR (151 MHz, CDCl$_3$)
163.61
158.28
154.53
122.61
93.41
52.87
O
N
N
I
H$_3$CO
2d
1000
900
800
700
600
500
400
300
200
100
0
230 220 210 200 190 180 170 160 150 140 130 120 110 100 90 80 70 60 50 40 30 20 10 0 -10
f1 (ppm)

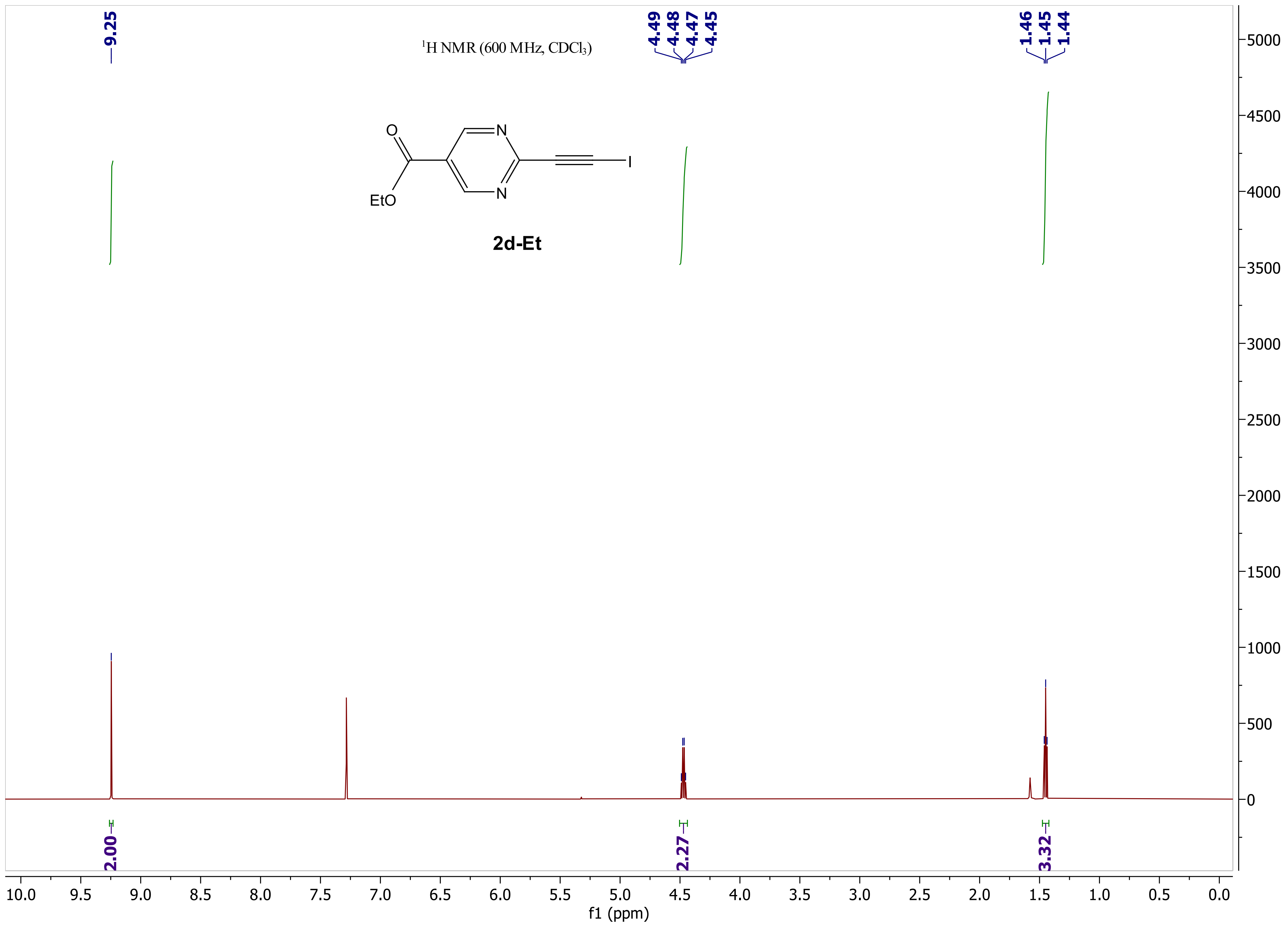

9.25
4.49
4.48
4.47
4.45
1.46
1.45
1.44
^{1}H NMR (600 MHz, CDCl$_3$)
O
N
I
N
EtO
2d-Et
5000
4500
4000
3500
3000
2500
2000
1500
1000
500
0
2.00
2.27
3.32
10.0
9.5
9.0
8.5
8.0
7.5
7.0
6.5
6.0
5.5
5.0
4.5
4.0
3.5
3.0
2.5
2.0
1.5
1.0
0.5
0.0
f1 (ppm)

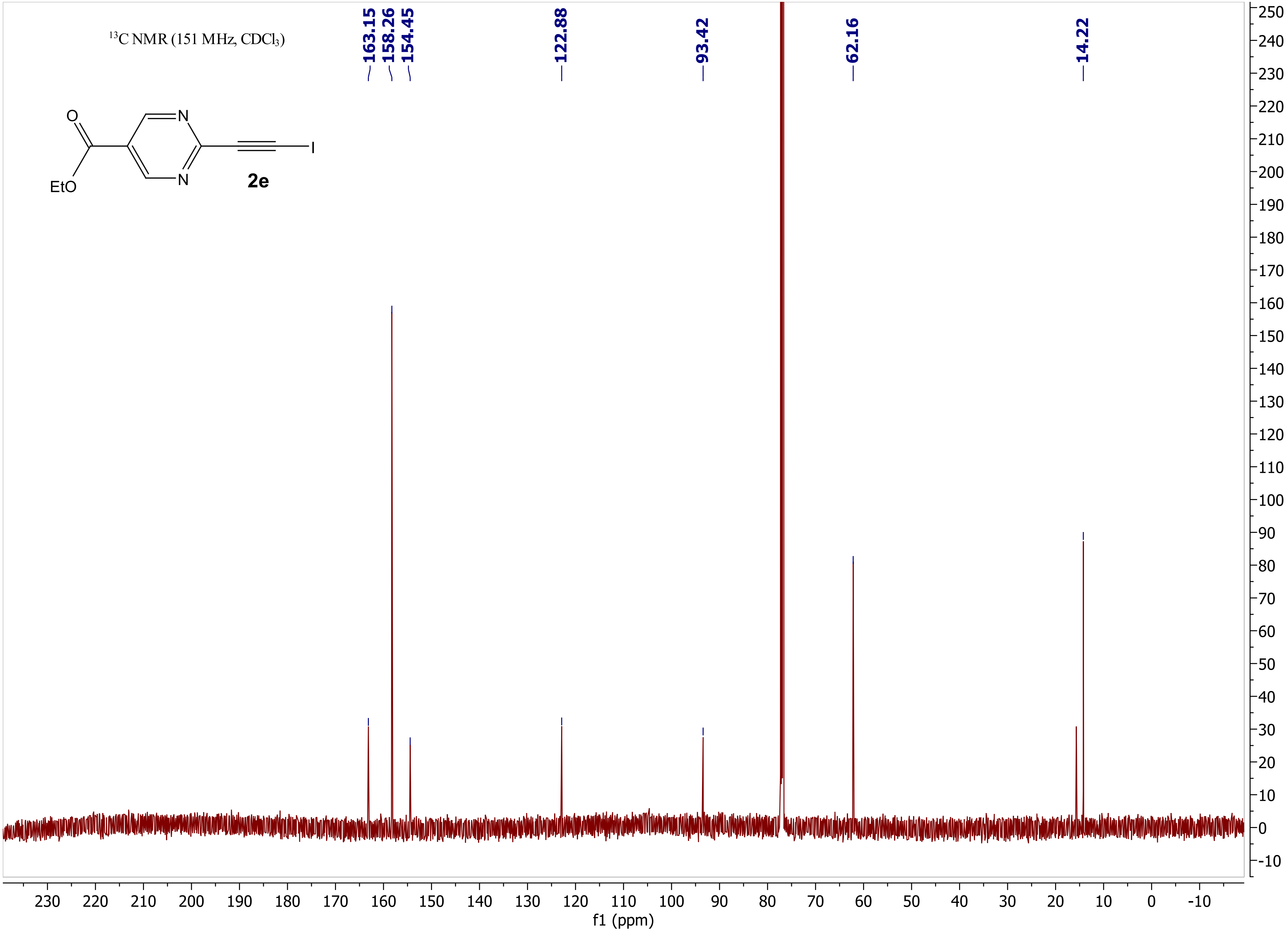

13C NMR (151 MHz, CDCl3)
163.15
158.26
154.45
122.88
93.42
62.16
14.22
O
N
N
I
EtO
2e
f1 (ppm)

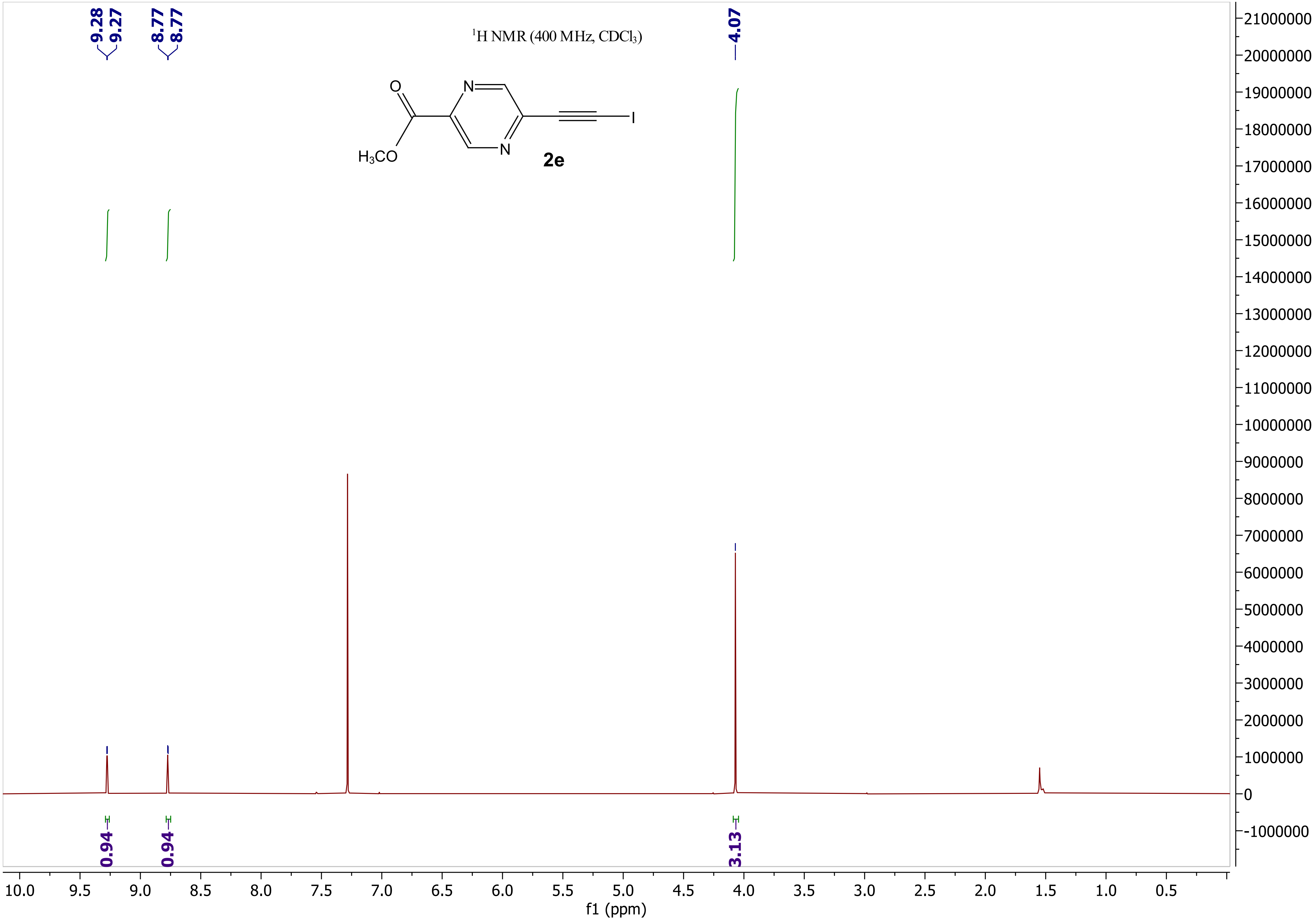

^1H NMR (400 MHz, $CDCl_3$)
9.28
9.27
8.77
8.77
4.07
O
N
N
I
H_3CO
2e
0.94
0.94
3.13
f1 (ppm)

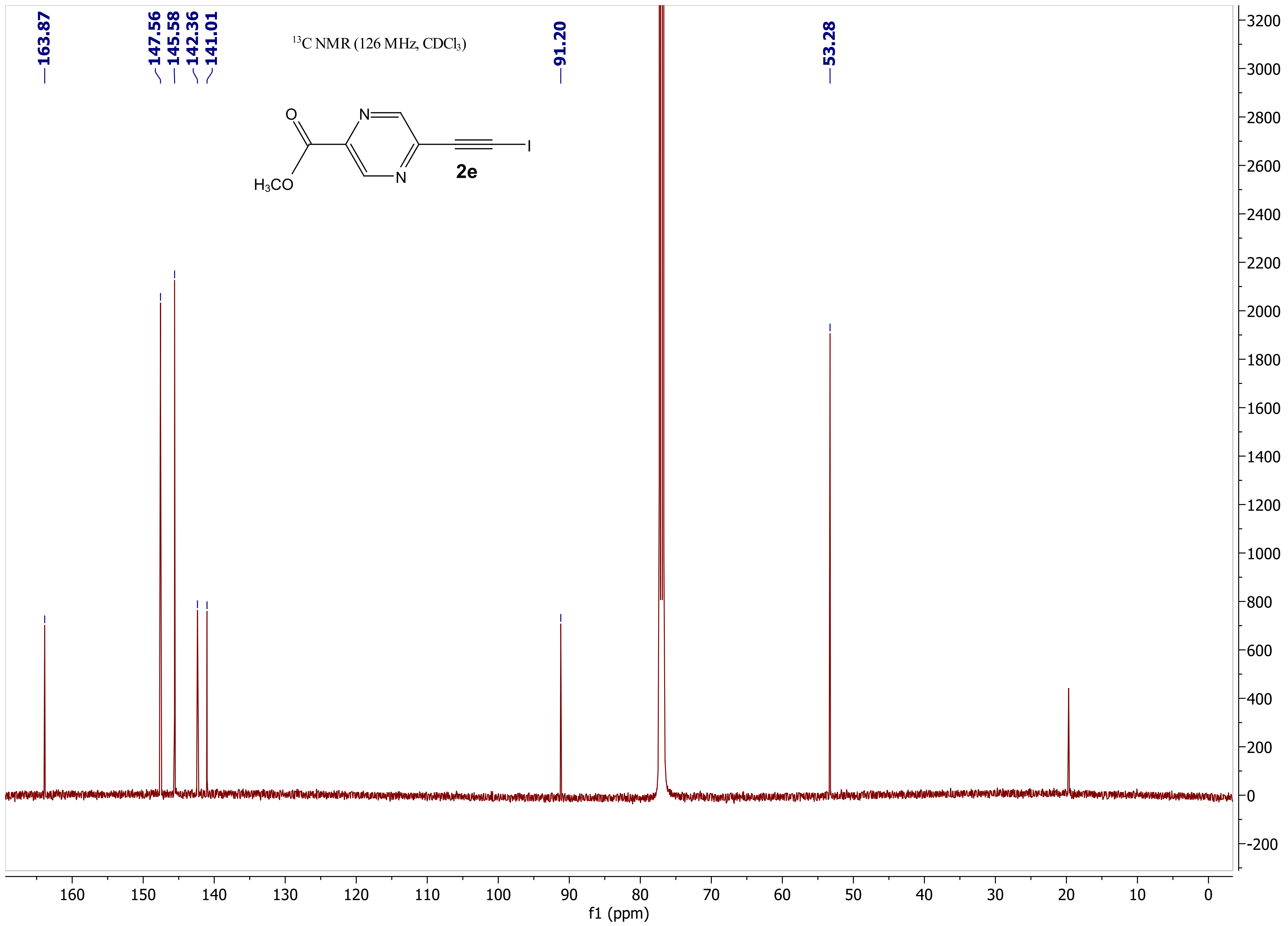

163.87
147.56
145.58
142.36
141.01
13C NMR (126 MHz, CDCl3)
91.20
53.28
O
N
N
H3CO
I
2e
f1 (ppm)

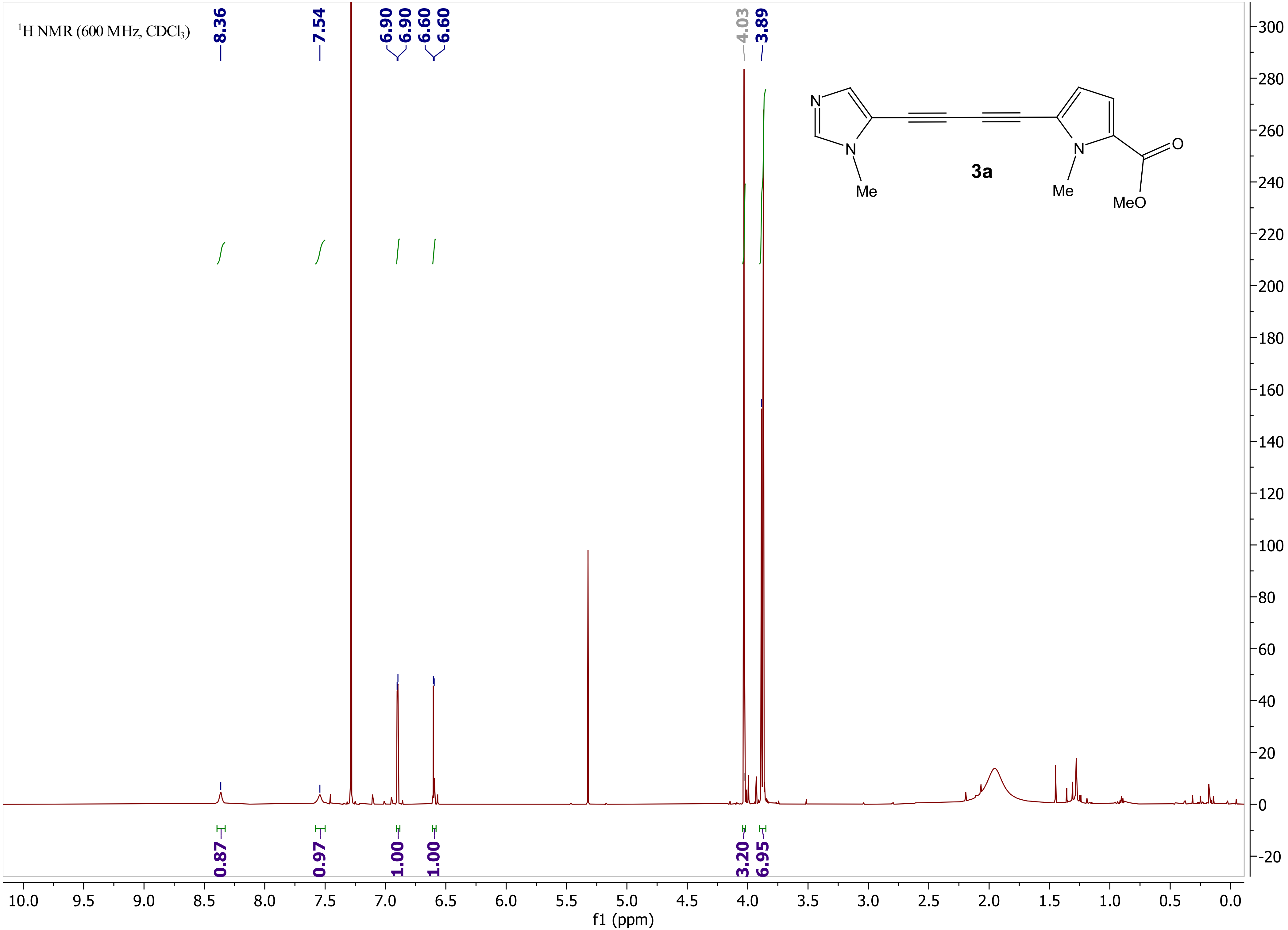

^{1}H NMR (600 MHz, CDCl$_3$)
8.36
7.54
6.90
6.90
6.60
6.60
4.03
3.89
3a
N
N
Me
N
Me
O
MeO
0.87
0.97
1.00
1.00
3.20
6.95
f1 (ppm)

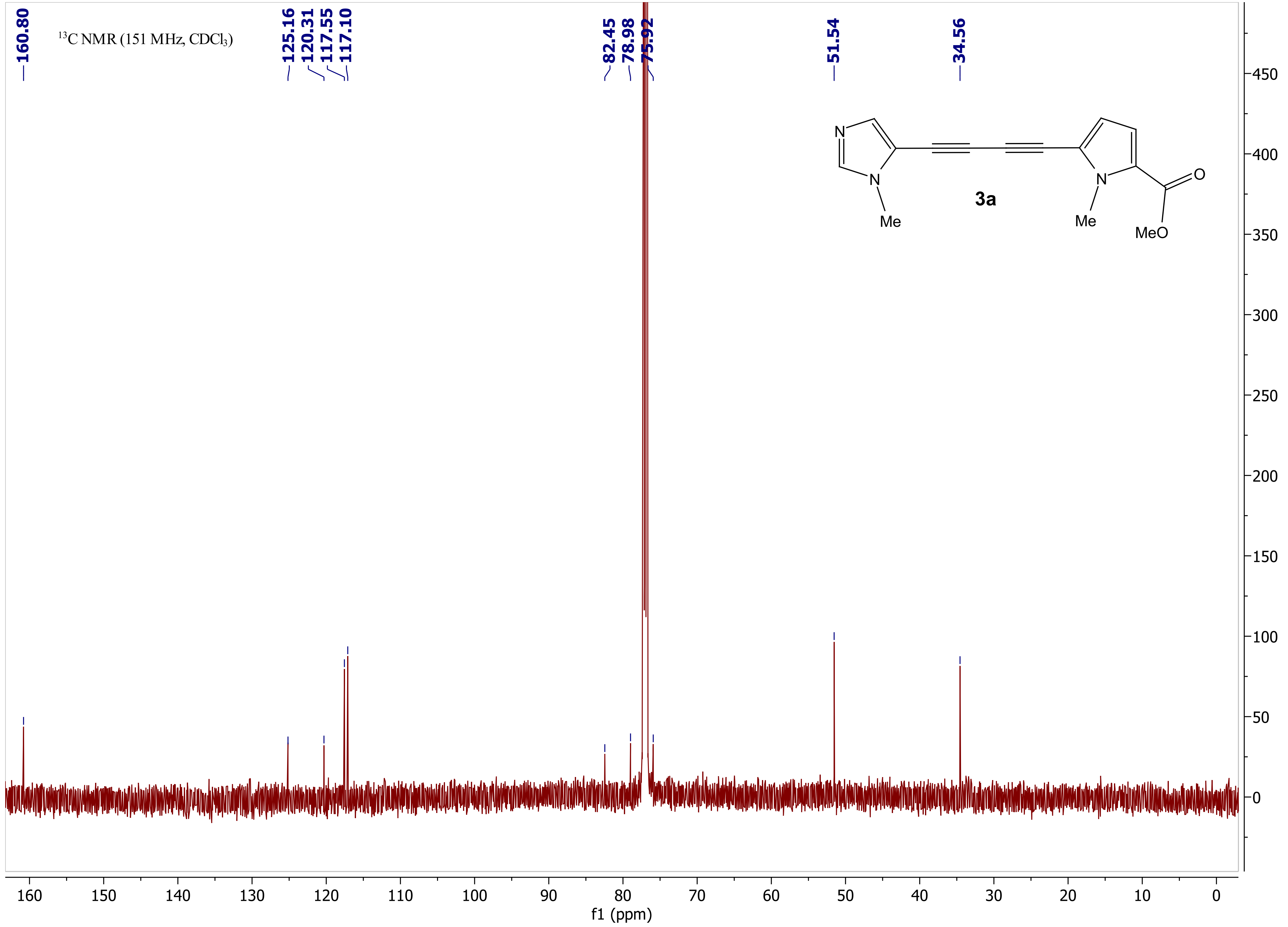
^{13}C NMR (151 MHz, CDCl$_3$)
160.80
125.16
120.31
117.55
117.10
82.45
78.98
75.92
51.54
34.56
N
N
Me
N
Me
O
MeO
3a
f1 (ppm)
160
150
140
130
120
110
100
90
80
70
60
50
40
30
20
10
0
450
400
350
300
250
200
150
100
50
0

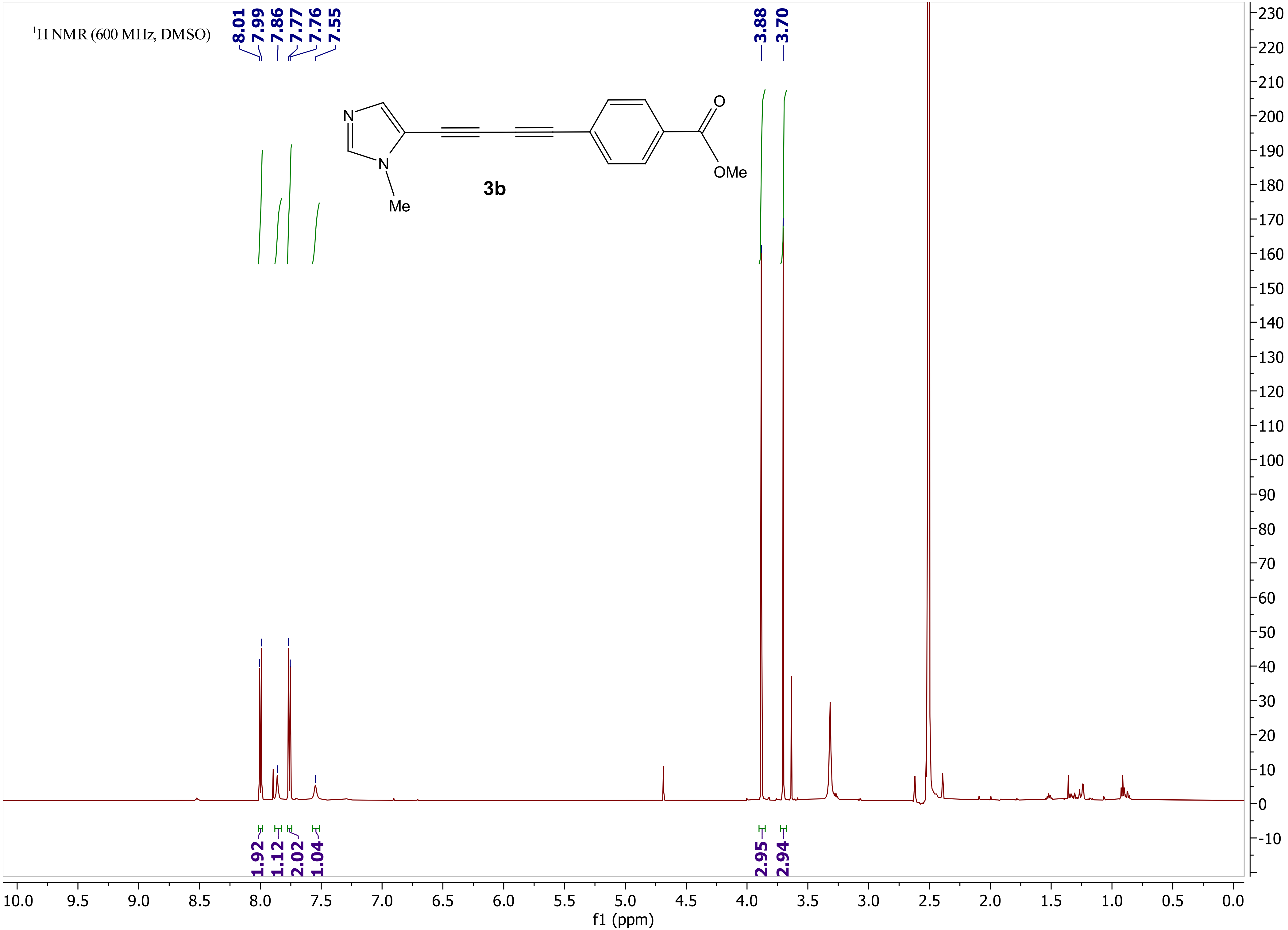

1H NMR (600 MHz, DMSO)
8.01
7.99
7.86
7.77
7.76
7.55
3.88
3.70
N
N
Me
O
OMe
3b
1.92
1.12
2.02
1.04
2.95
2.94
f1 (ppm)

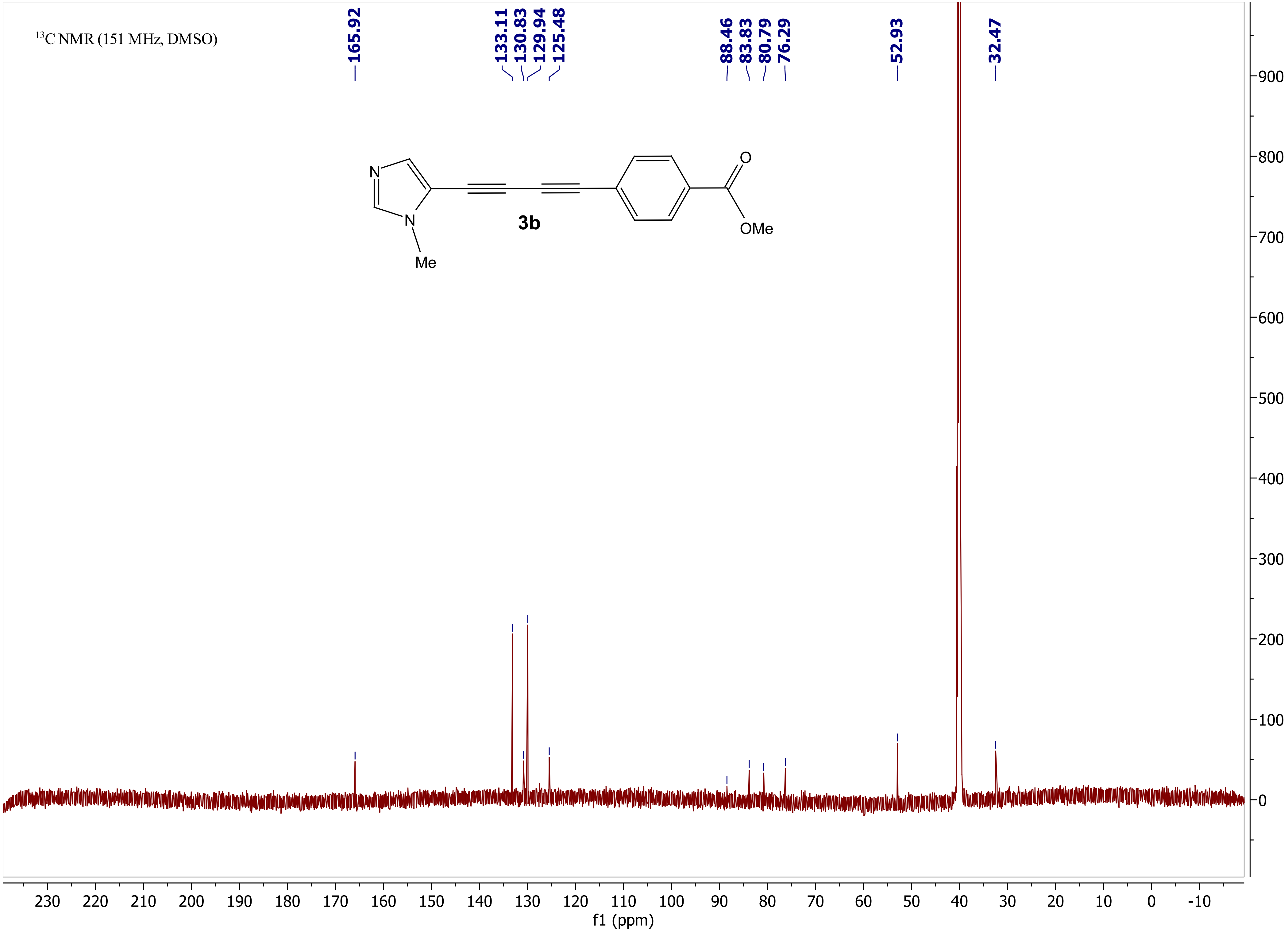

^{13}C NMR (151 MHz, DMSO)
165.92
133.11
130.83
129.94
125.48
88.46
83.83
80.79
76.29
52.93
32.47
N
N
Me
3b
O
OMe
f1 (ppm)

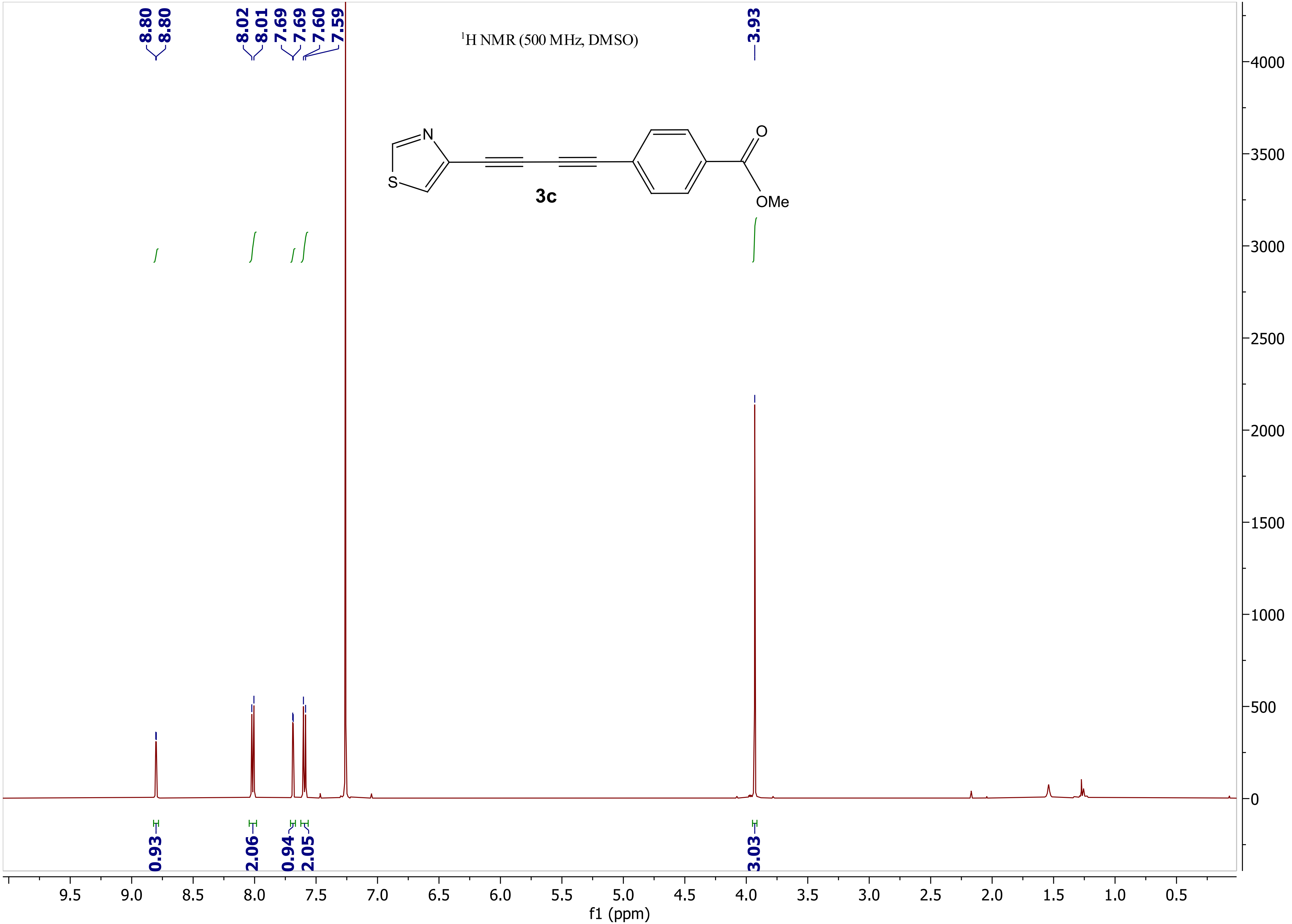

8.80
8.80
8.02
8.01
7.69
7.69
7.60
7.59
3.93
1H NMR (500 MHz, DMSO)
N
S
O
OMe
3c
0.93
2.06
0.94
2.05
3.03
f1 (ppm)

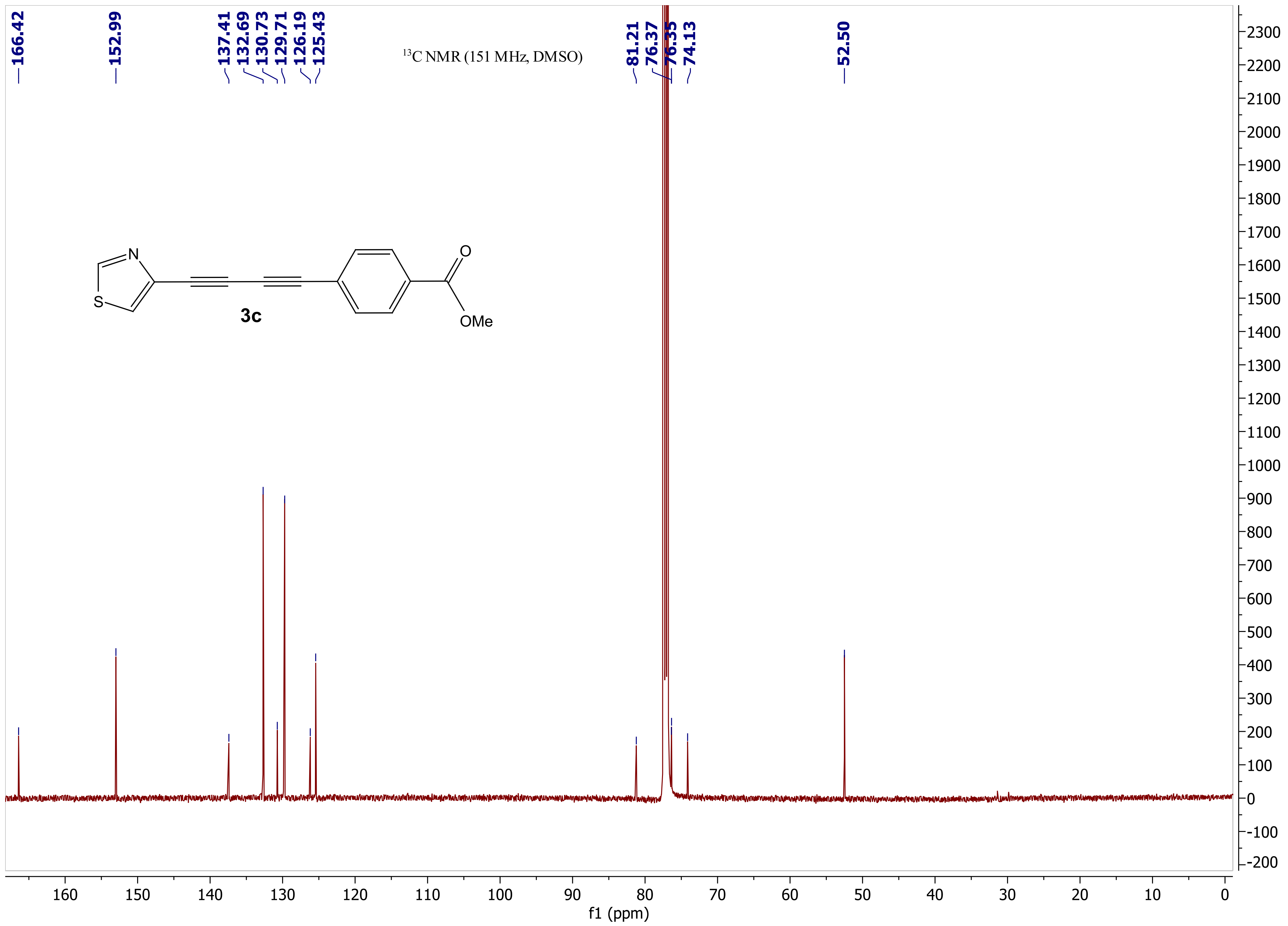

166.42
152.99
137.41
132.69
130.73
129.71
126.19
125.43
^{13}C NMR (151 MHz, DMSO)
81.21
76.37
76.35
74.13
52.50
N
S
3c
O
OMe
f1 (ppm)

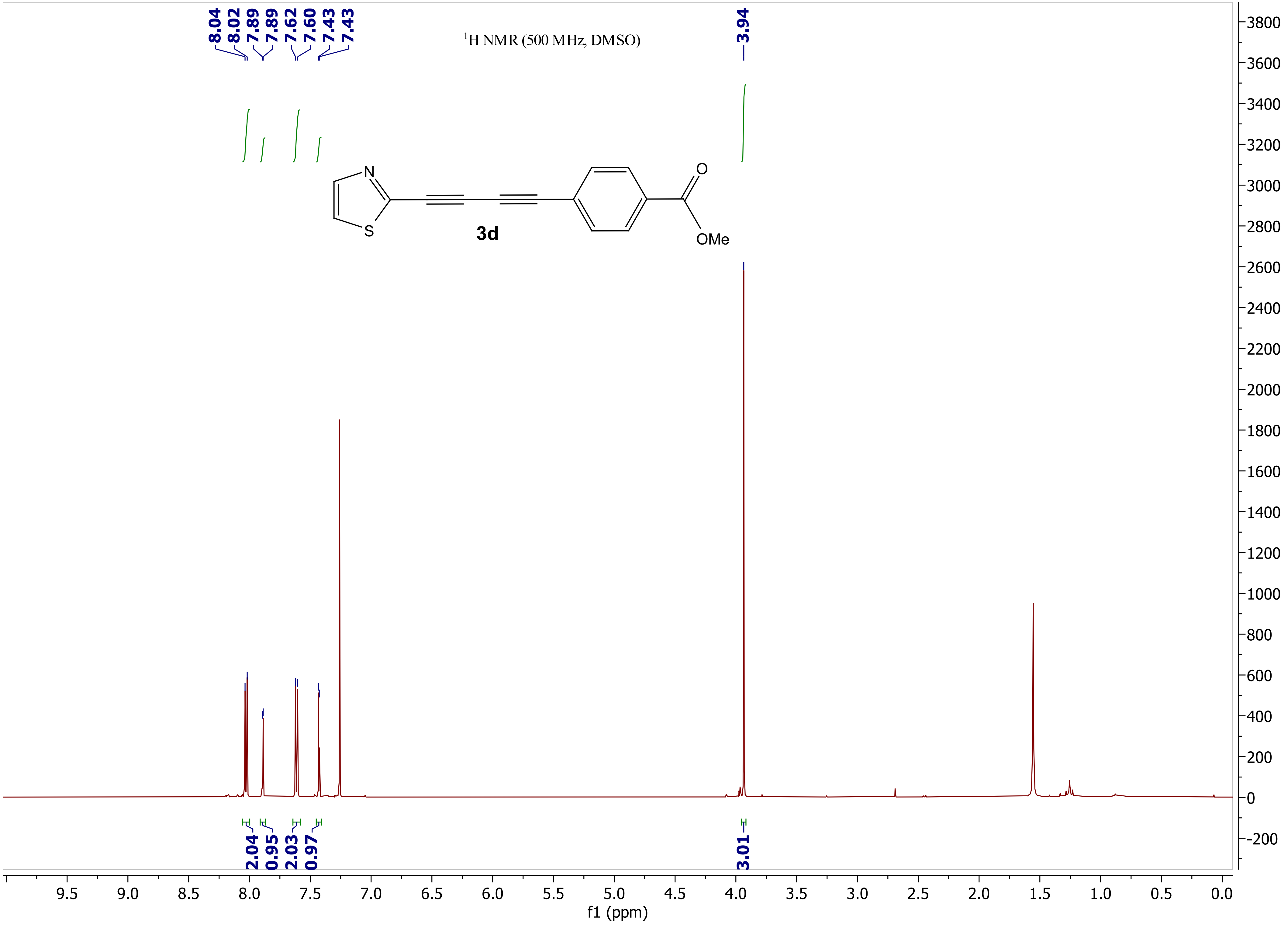

8.04
8.02
7.89
7.89
7.62
7.60
7.43
7.43
3.94
¹H NMR (500 MHz, DMSO)
N
S
O
OMe
3d
2.04
0.95
2.03
0.97
3.01
f1 (ppm)

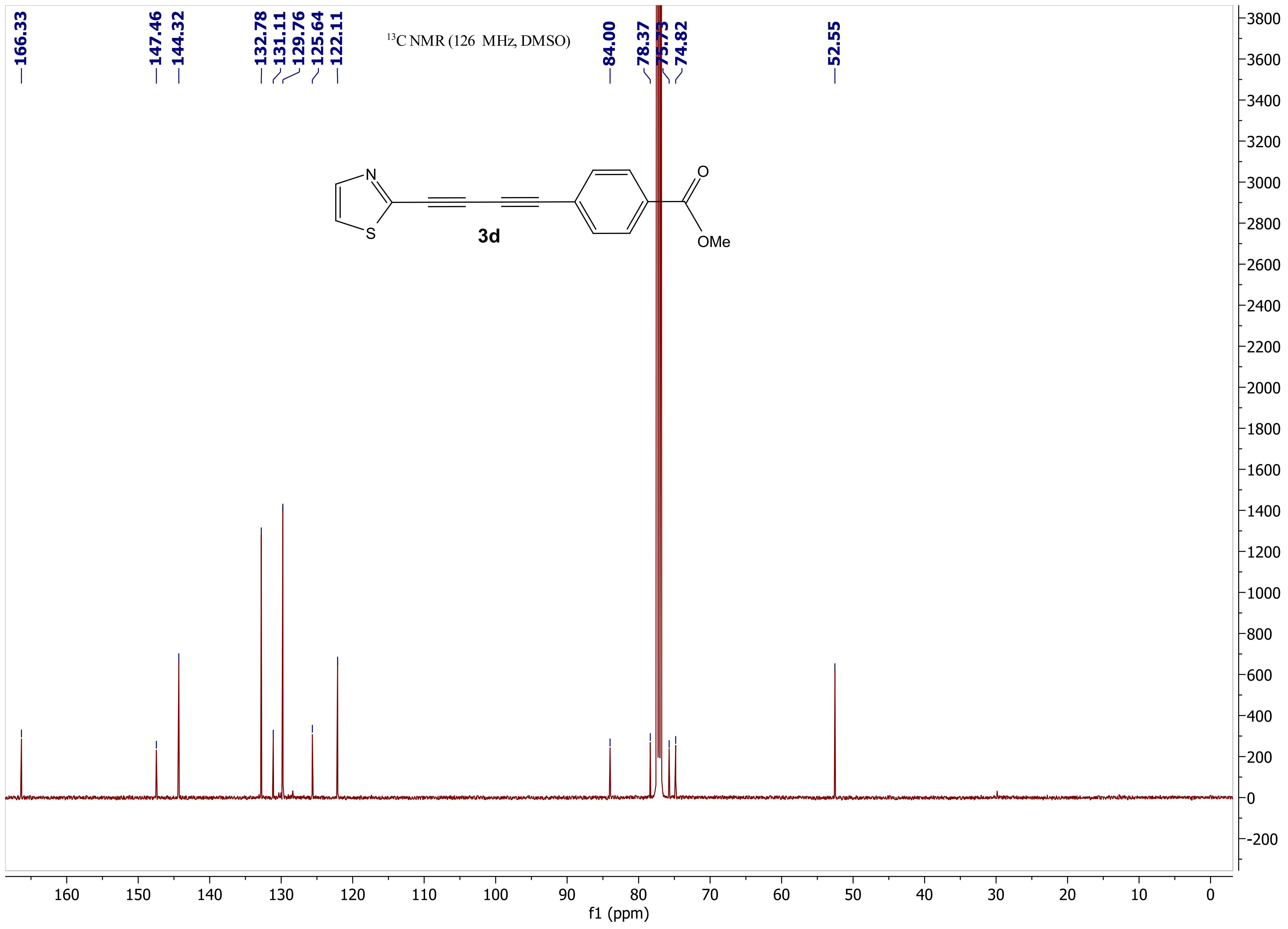
166.33
147.46
144.32
132.78
131.11
129.76
125.64
122.11
13C NMR (126 MHz, DMSO)
84.00
78.37
75.73
74.82
52.55
N
S
O
OMe
3d
f1 (ppm)

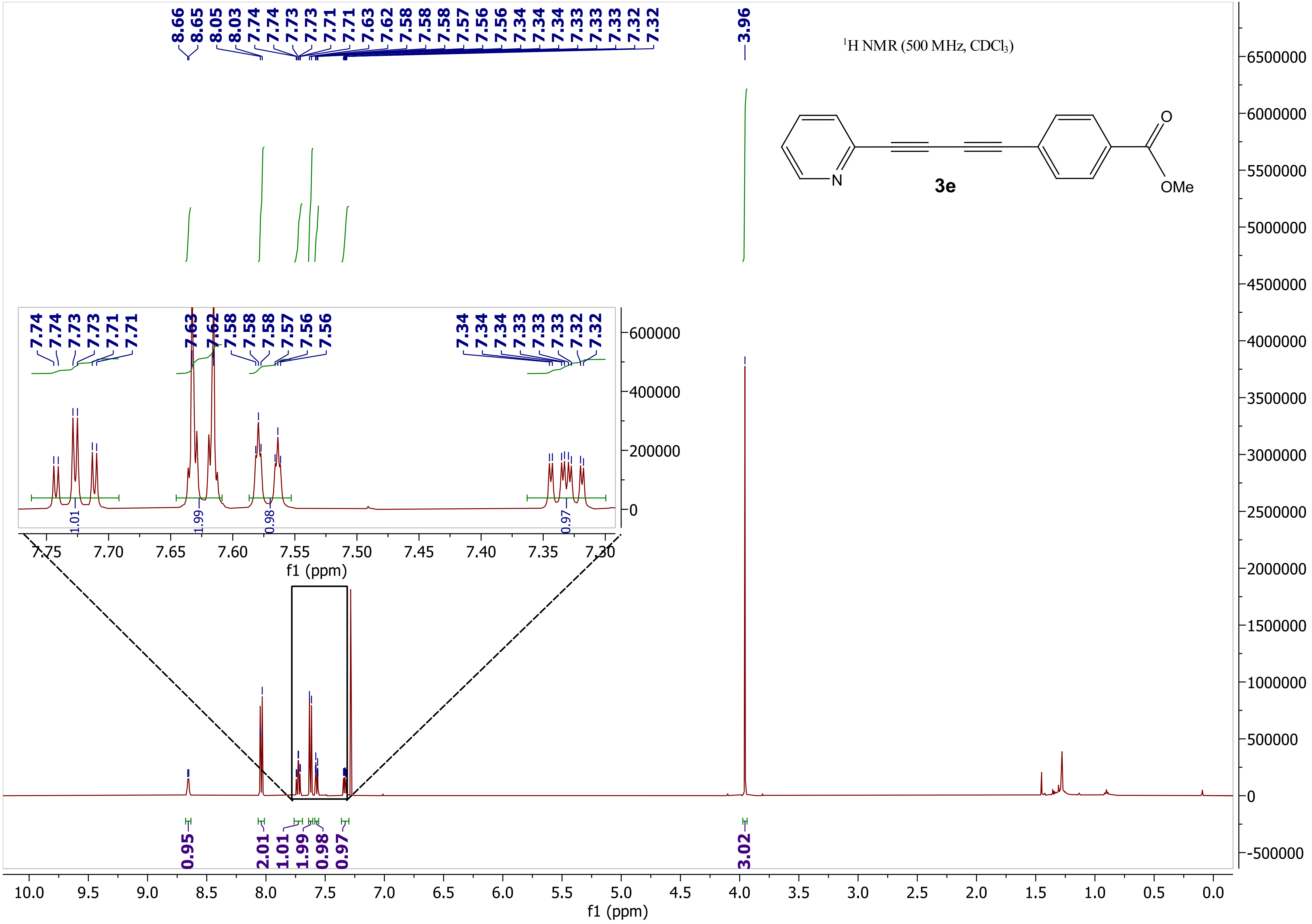

8.66 8.65 8.05 8.03 7.74 7.74 7.73 7.73 7.71 7.71 7.63 7.62 7.58 7.58 7.58 7.57 7.56 7.56 7.34 7.34 7.34 7.33 7.33 7.33 7.32 7.32
3.96
^{1}H NMR (500 MHz, CDCl3)
3e
N
O
OMe
0.95
2.01
1.01
1.99
0.98
0.97
3.02
f1 (ppm)

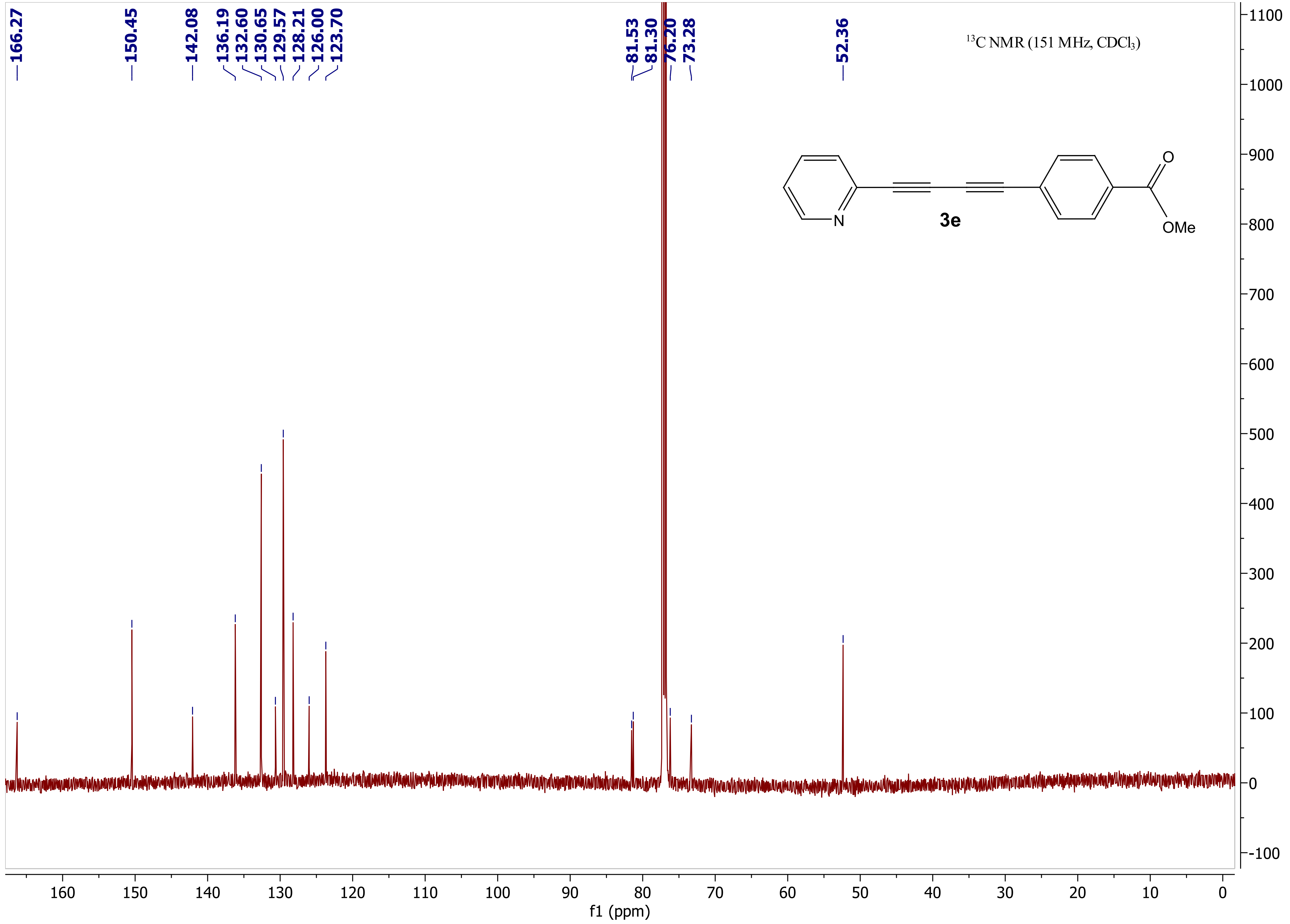

166.27
150.45
142.08
136.19
132.60
130.65
129.57
128.21
126.00
123.70
81.53
81.30
76.20
73.28
52.36
^{13}C NMR (151 MHz, $CDCl_3$)
O
N
3e
OMe
f1 (ppm)

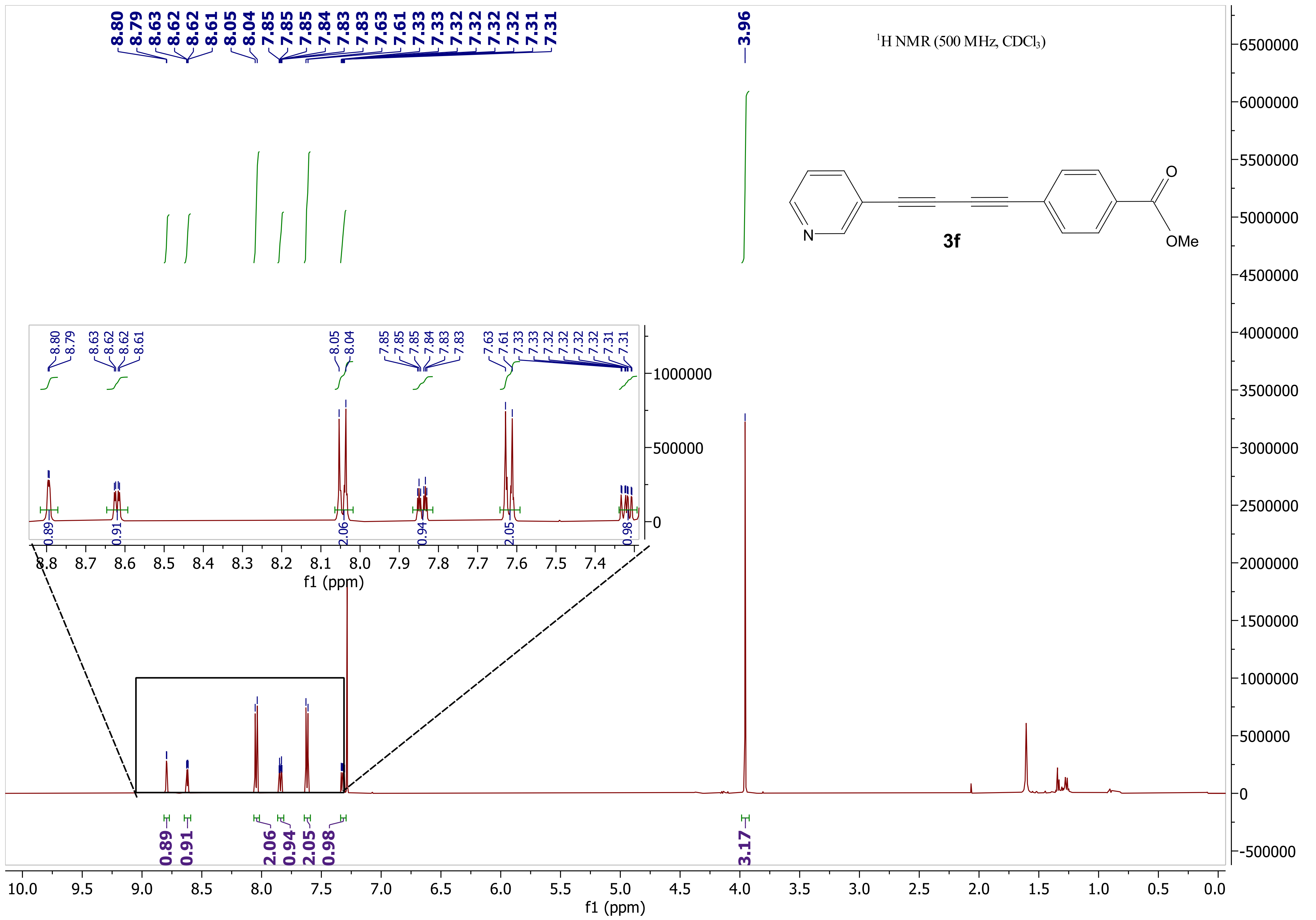

^{1}H NMR (500 MHz, $CDCl_3$)
N
O
OMe
3f
8.80 8.79 8.63 8.62 8.62 8.61 8.05 8.04 7.85 7.85 7.85 7.84 7.83 7.83 7.63 7.61 7.33 7.33 7.32 7.32 7.32 7.32 7.31 7.31
3.96
0.89 0.91 2.06 0.94 2.05 0.98 3.17
f1 (ppm)

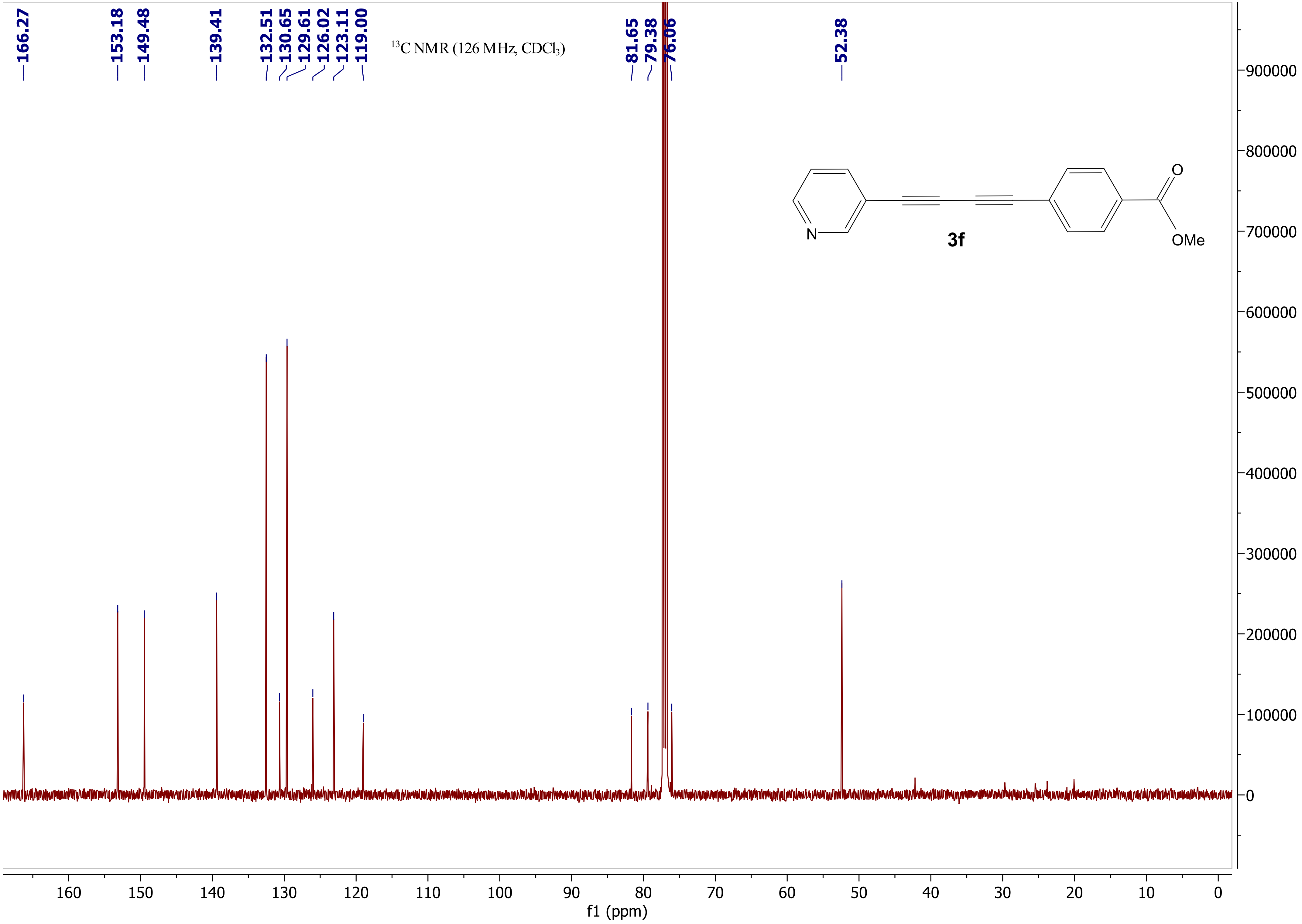

166.27
153.18
149.48
139.41
132.51
130.65
129.61
126.02
123.11
119.00
^{13}C NMR (126 MHz, CDCl$_3$)
81.65
79.38
76.06
52.38
N
O
OMe
3f
900000
800000
700000
600000
500000
400000
300000
200000
100000
0
160
150
140
130
120
110
100
90
80
70
60
50
40
30
20
10
0
f1 (ppm)

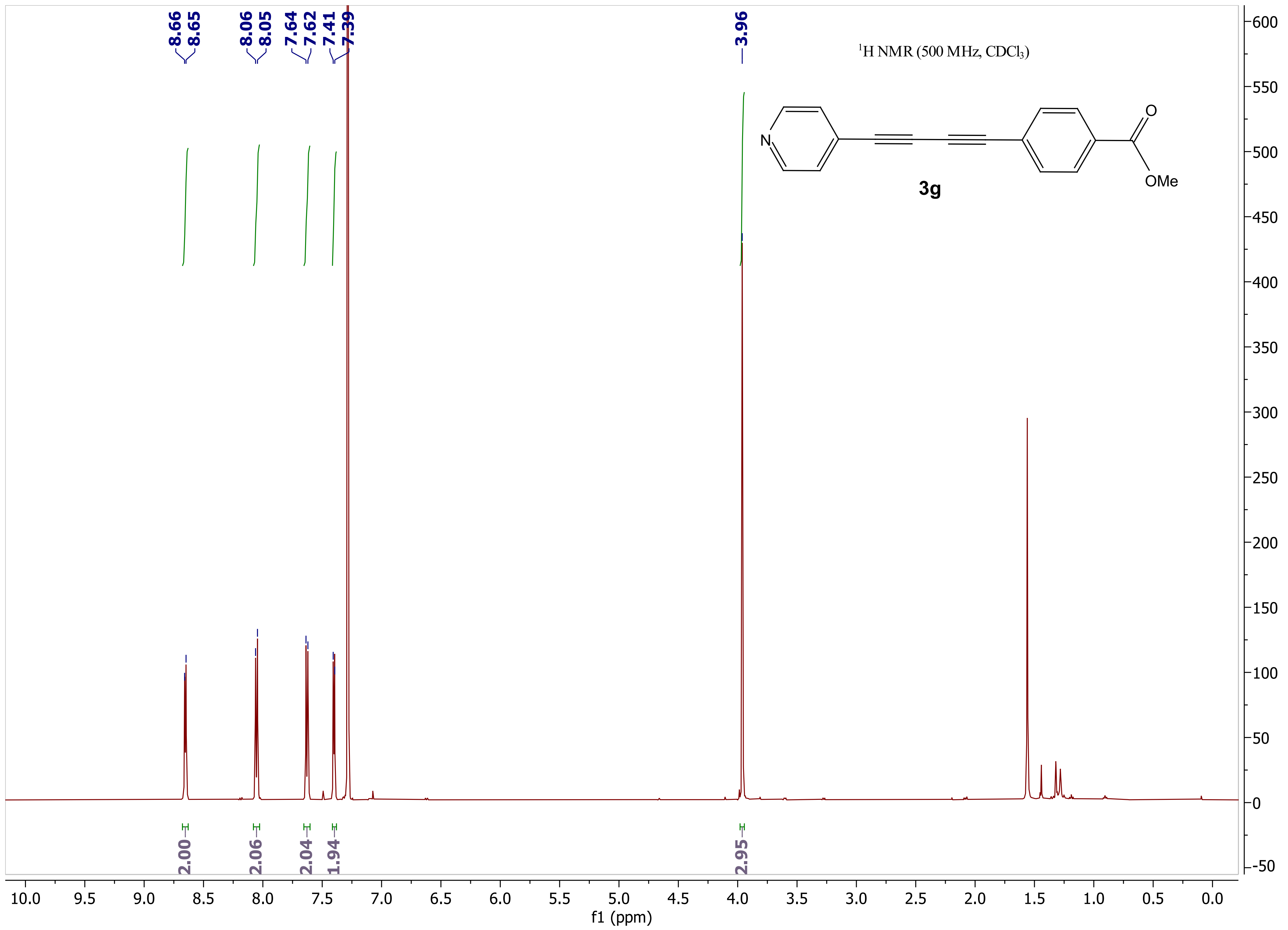

8.66
8.65
8.06
8.05
7.64
7.62
7.41
7.39
3.96
¹H NMR (500 MHz, CDCl₃)
N
O
OMe
3g
2.00
2.06
2.04
1.94
2.95
f1 (ppm)

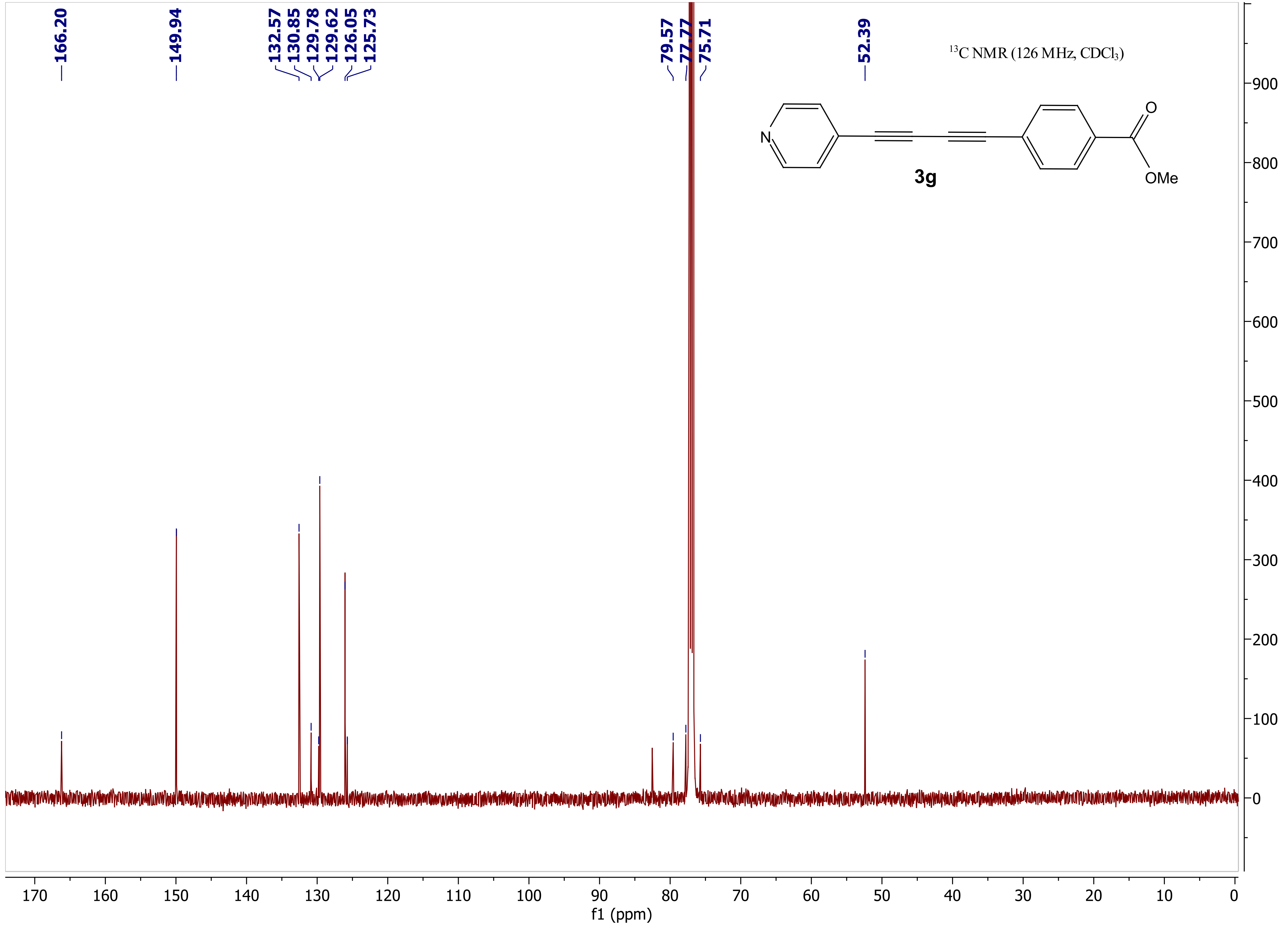

166.20
149.94
132.57
130.85
129.78
129.62
126.05
125.73
79.57
77.77
75.71
52.39
^{13}C NMR (126 MHz, $CDCl_3$)
N
O
OMe
3g
f1 (ppm)

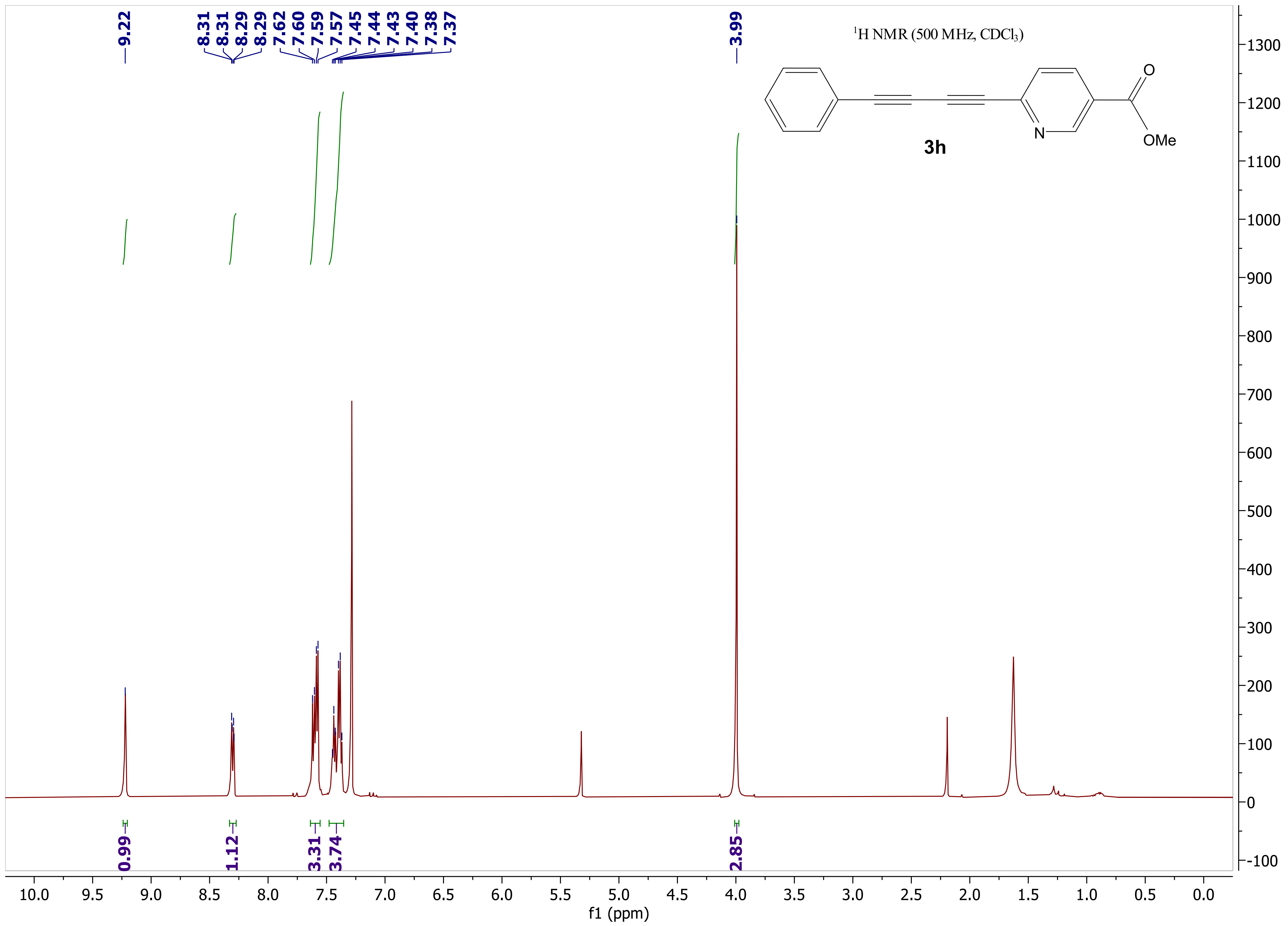

¹H NMR (500 MHz, CDCl₃)
3h
N
O
OMe
9.22
8.31
8.31
8.29
8.29
7.62
7.60
7.59
7.57
7.45
7.44
7.43
7.40
7.38
7.37
3.99
0.99
1.12
3.31
3.74
2.85
f1 (ppm)

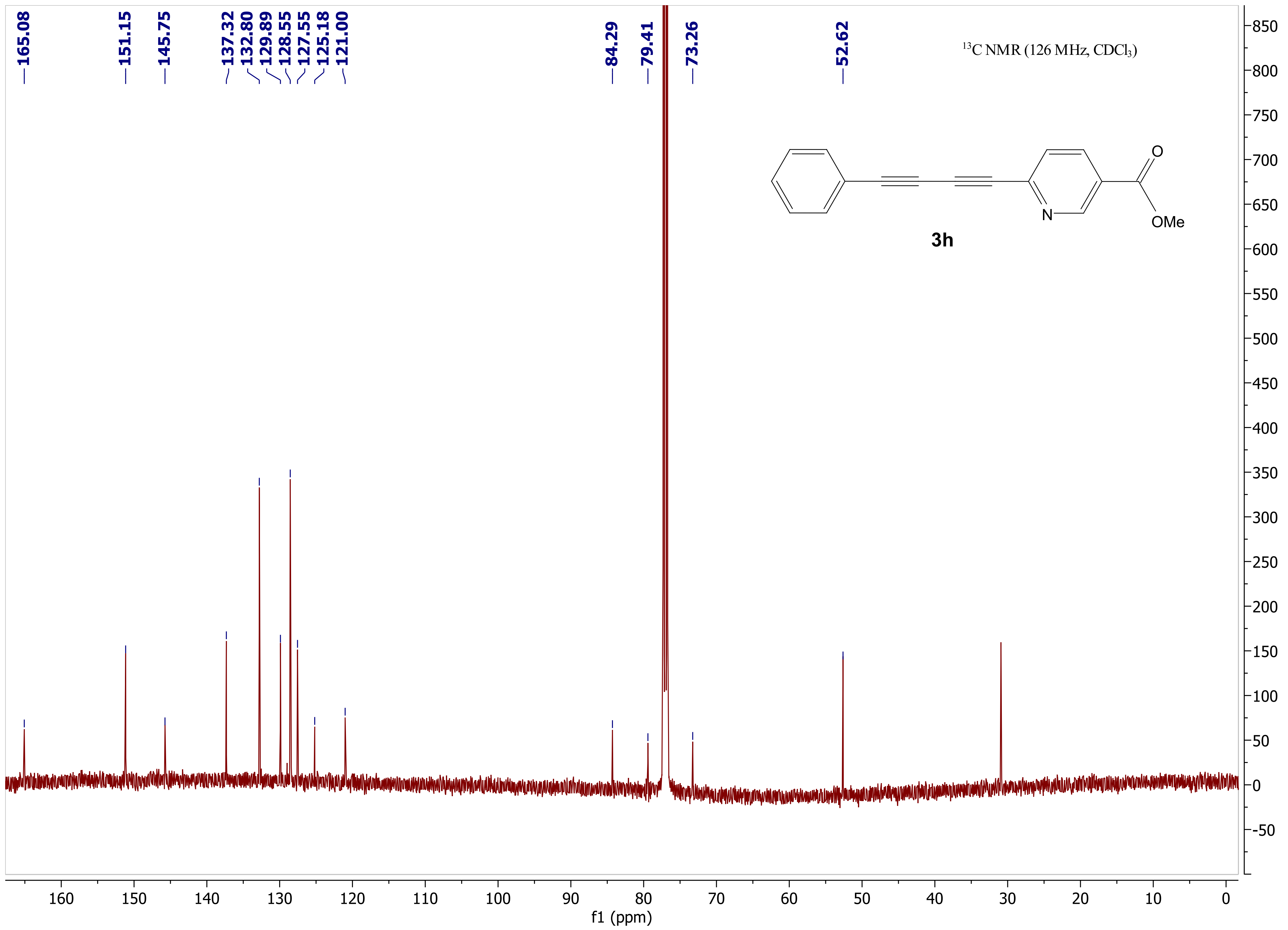
165.08
151.15
145.75
137.32
132.80
129.89
128.55
127.55
125.18
121.00
84.29
79.41
73.26
52.62
^{13}C NMR (126 MHz, CDCl$_3$)
O
N
OMe
3h
f1 (ppm)

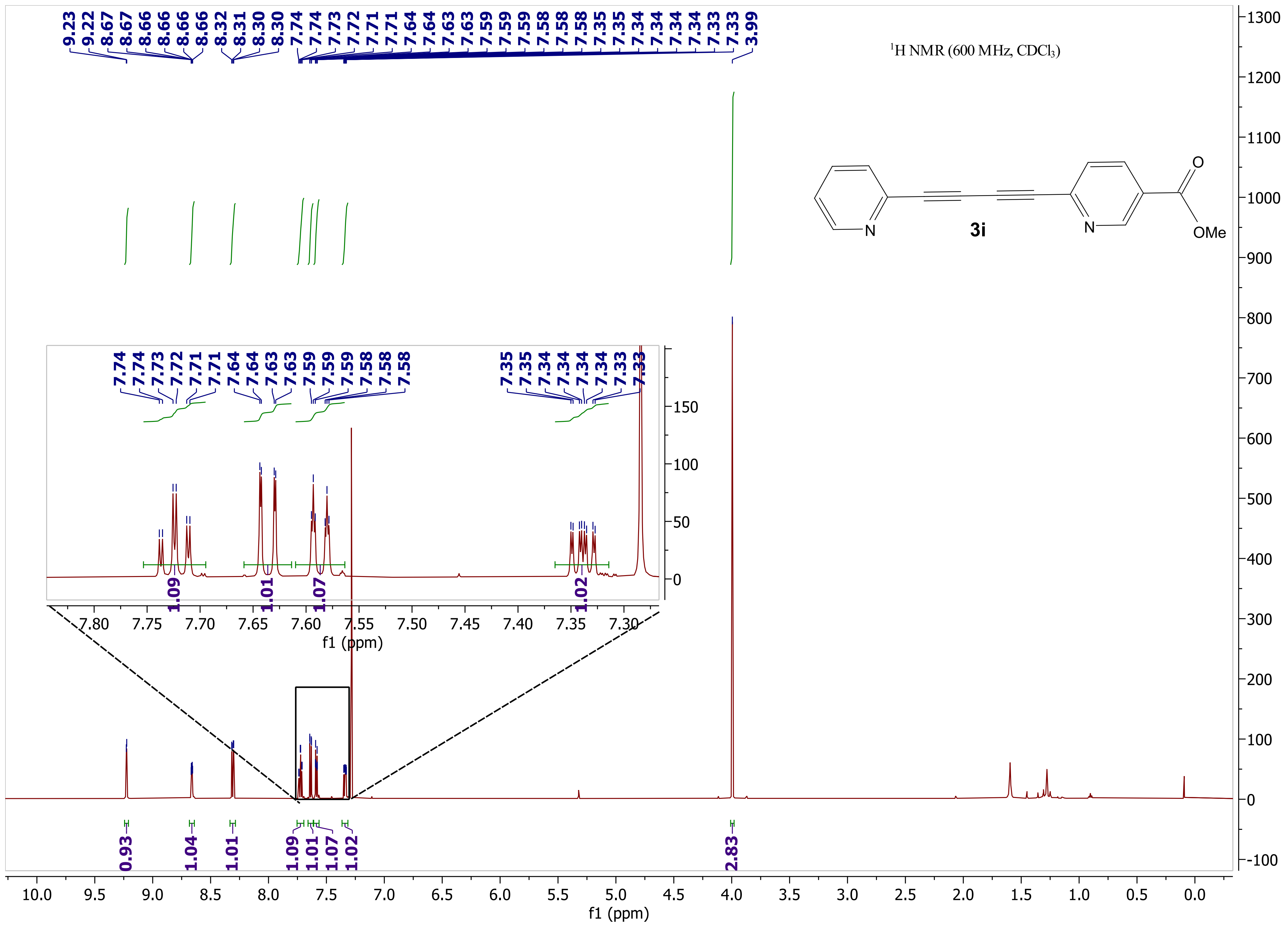

^{1}H NMR (600 MHz, CDCl$_3$)
3i
N
N
O
OMe
9.23 9.22 8.67 8.67 8.66 8.66 8.66 8.66 8.32 8.31 8.30 8.30 7.74 7.74 7.73 7.72 7.71 7.71 7.64 7.64 7.63 7.63 7.59 7.59 7.59 7.58 7.58 7.58 7.35 7.35 7.34 7.34 7.34 7.34 7.33 7.33 3.99
0.93 1.04 1.01 1.09 1.01 1.07 1.02 2.83
f1 (ppm)

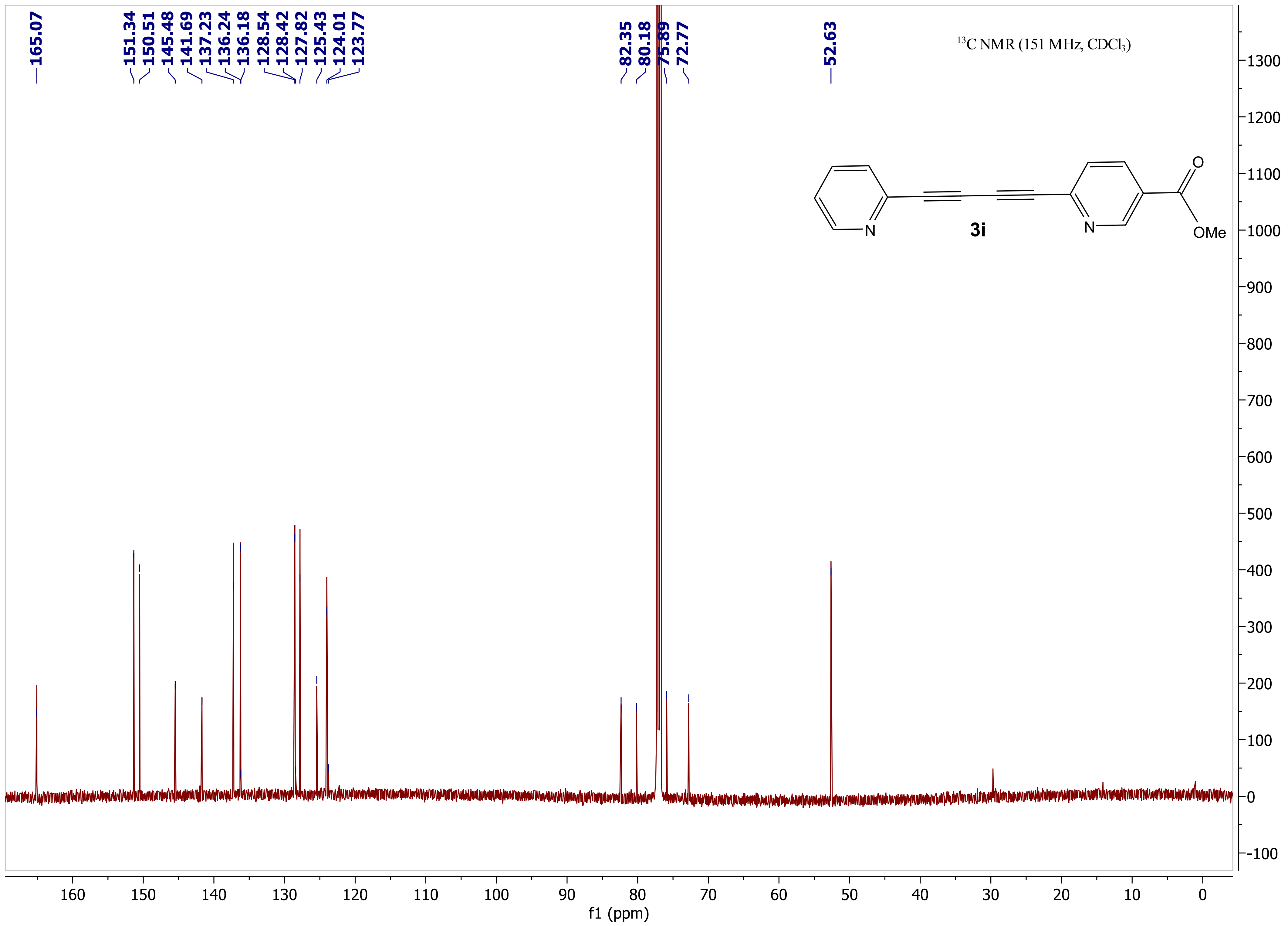

165.07
151.34
150.51
145.48
141.69
137.23
136.24
136.18
128.54
128.42
127.82
125.43
124.01
123.77
82.35
80.18
75.89
72.77
52.63
^{13}C NMR (151 MHz, CDCl$_3$)
N
N
O
OMe
3i
f1 (ppm)

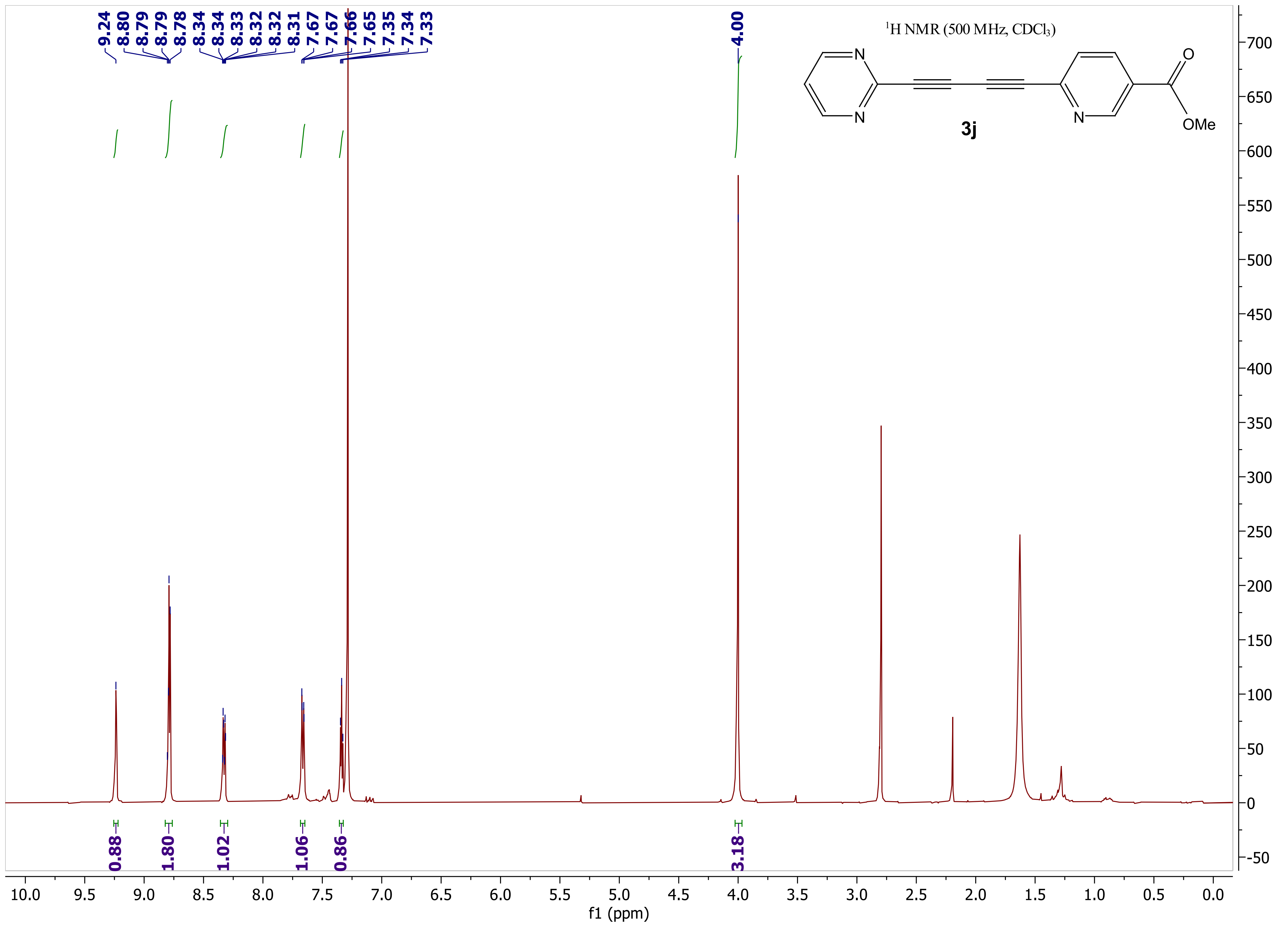

9.24
8.80
8.79
8.79
8.78
8.34
8.34
8.33
8.32
8.32
8.31
7.67
7.67
7.66
7.65
7.35
7.34
7.33
4.00
¹H NMR (500 MHz, CDCl₃)
N
N
N
O
OMe
3j
0.88
1.80
1.02
1.06
0.86
3.18
f1 (ppm)

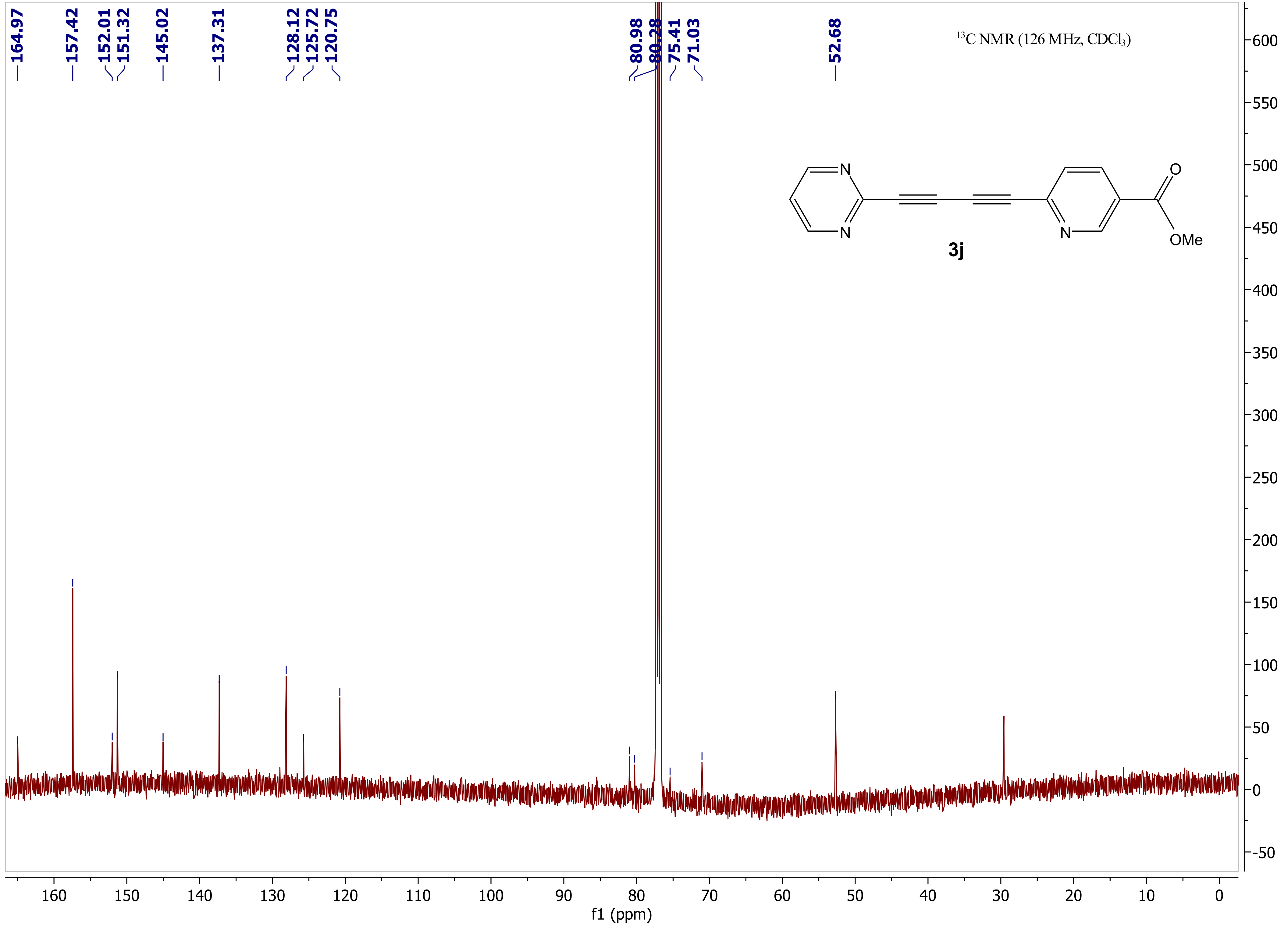

164.97
157.42
152.01
151.32
145.02
137.31
128.12
125.72
120.75
80.98
80.28
75.41
71.03
52.68
^{13}C NMR (126 MHz, $CDCl_3$)
N
N
N
O
OMe
3j
f1 (ppm)

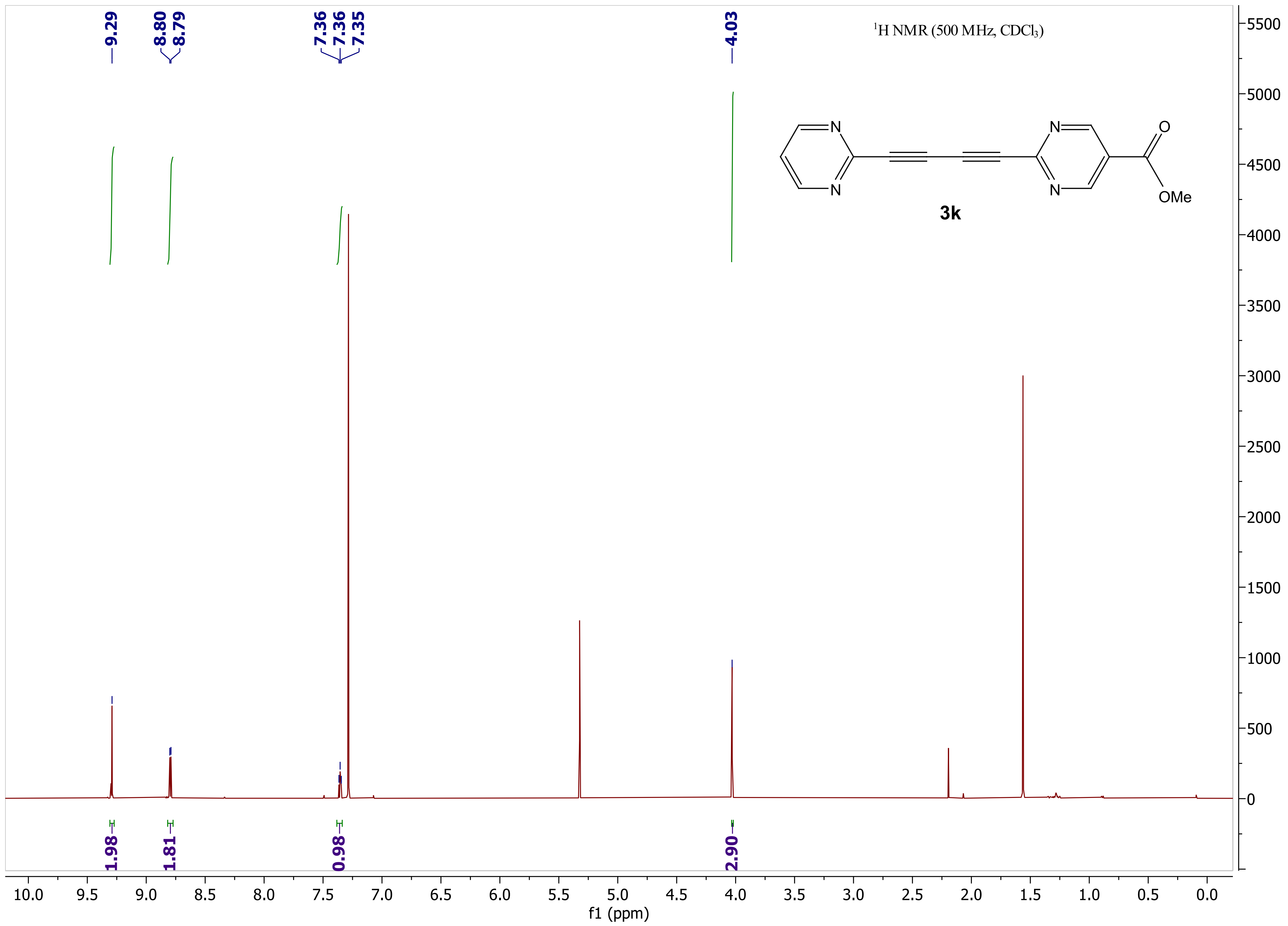

1H NMR (500 MHz, CDCl3)
9.29
8.80
8.79
7.36
7.36
7.35
4.03
N
N
N
N
O
OMe
3k
1.98
1.81
0.98
2.90
f1 (ppm)

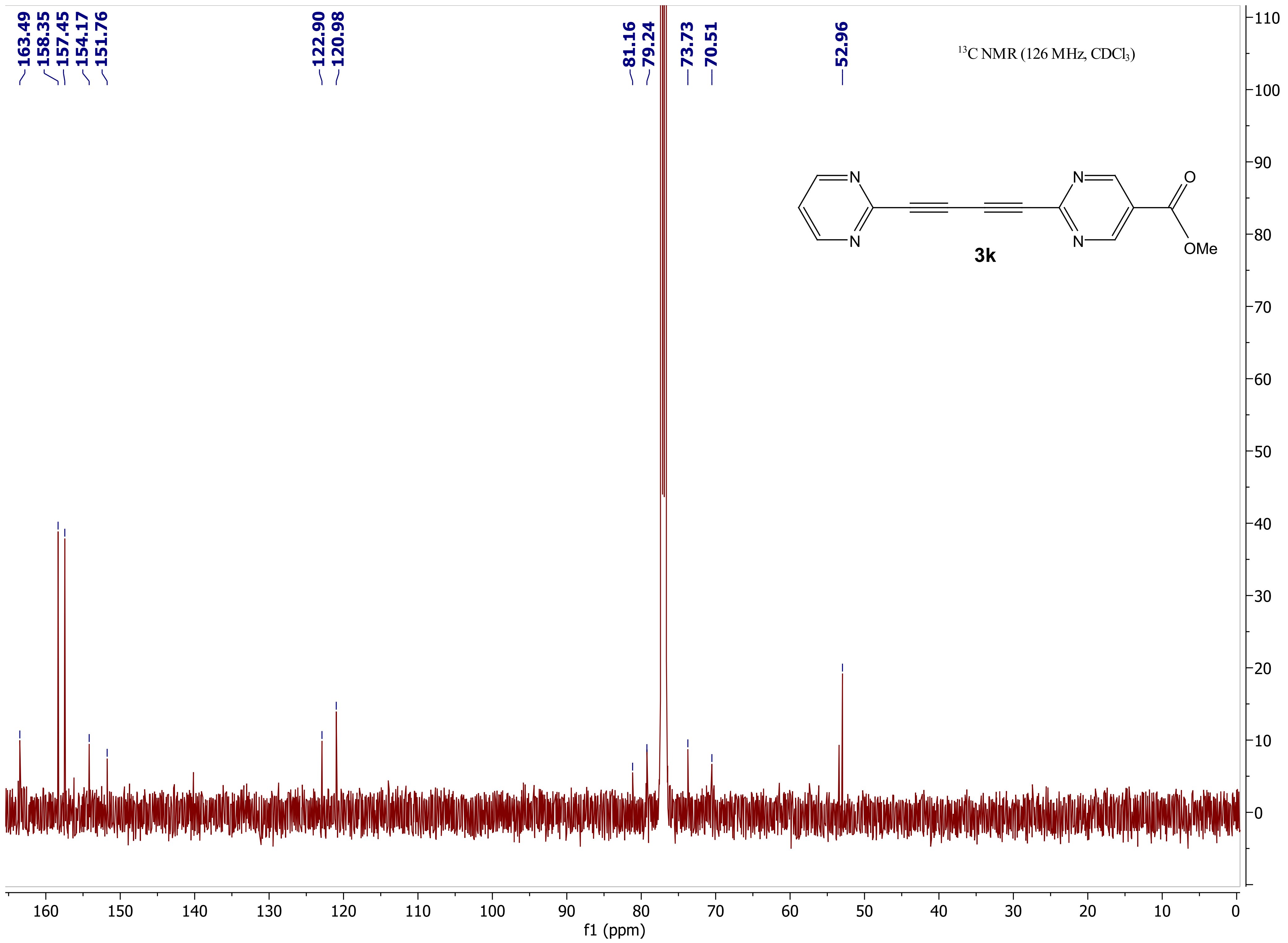
163.49
158.35
157.45
154.17
151.76
122.90
120.98
81.16
79.24
73.73
70.51
52.96
13C NMR (126 MHz, CDCl3)
N
N
N
N
O
OMe
3k
f1 (ppm)
160
150
140
130
120
110
100
90
80
70
60
50
40
30
20
10
0
110
100
90
80
70
60
50
40
30
20
10
0

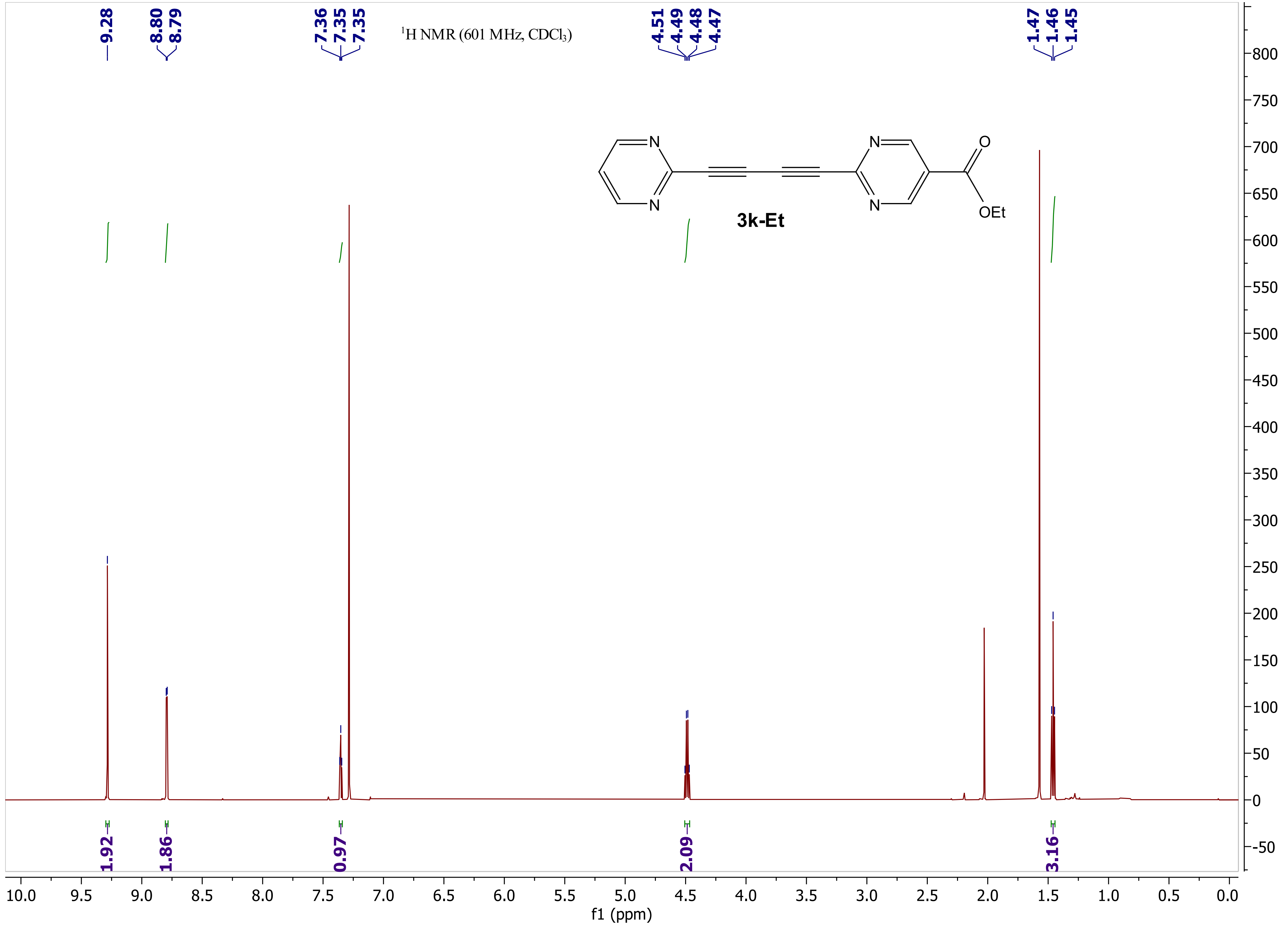

9.28
8.80
8.79
7.36
7.35
7.35
¹H NMR (601 MHz, CDCl₃)
4.51
4.49
4.48
4.47
1.47
1.46
1.45
3k-Et
OEt
1.92
1.86
0.97
2.09
3.16
f1 (ppm)

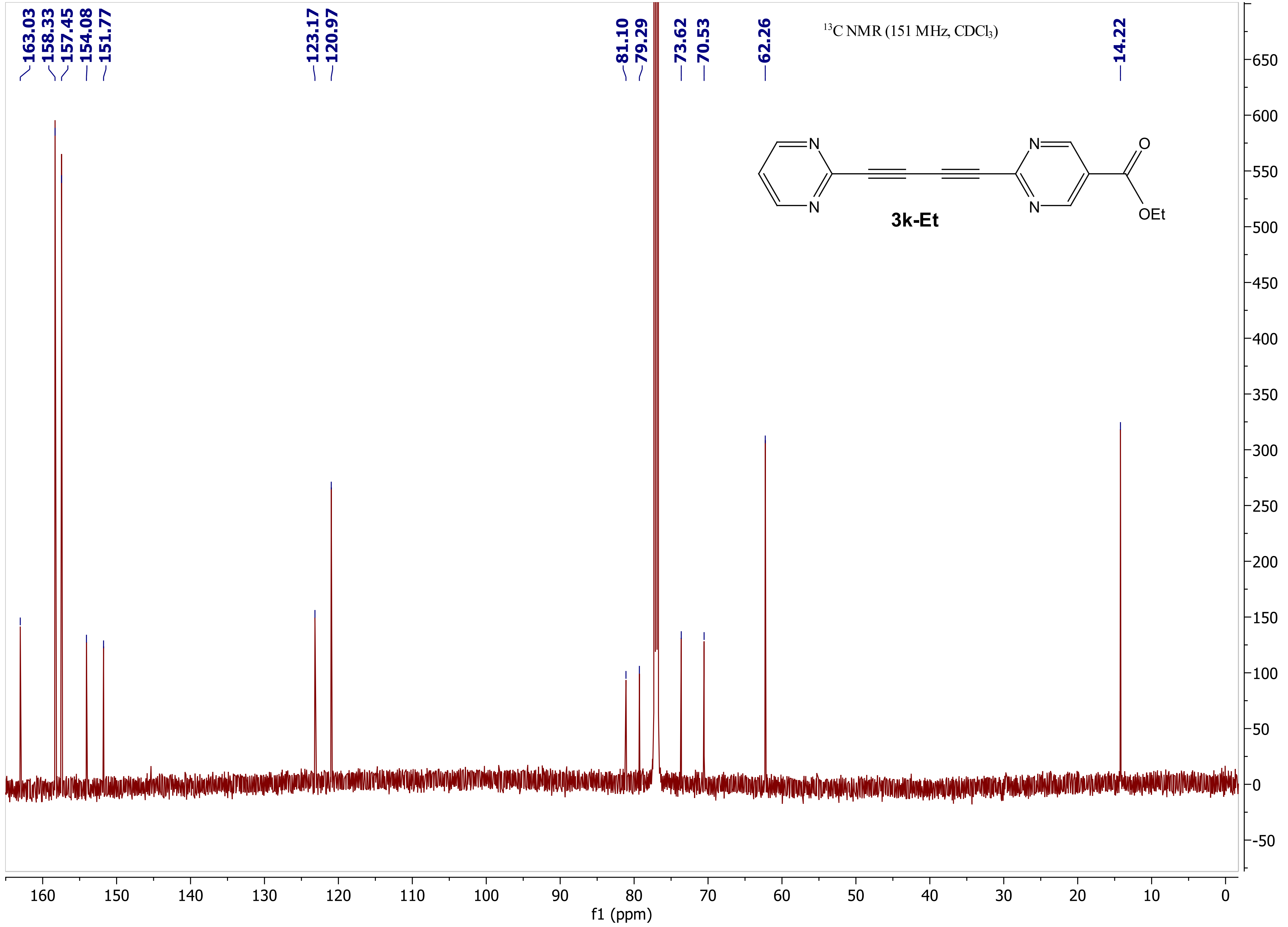

163.03
158.33
157.45
154.08
151.77
123.17
120.97
81.10
79.29
73.62
70.53
62.26
14.22
^{13}C NMR (151 MHz, $CDCl_3$)
N
N
N
N
O
OEt
3k-Et
f1 (ppm)

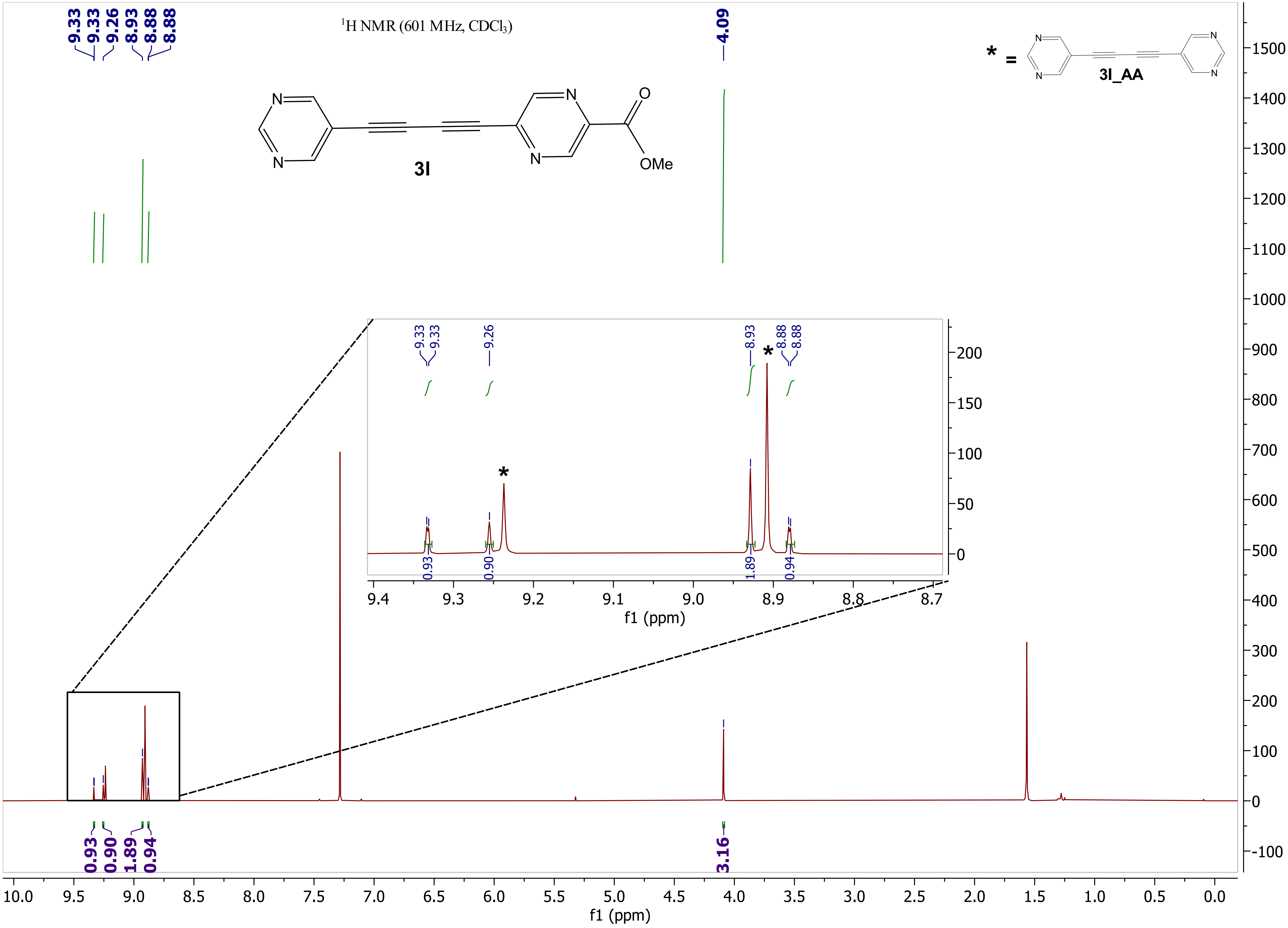

¹H NMR (601 MHz, CDCl₃)
3l
* = 3l_AA
9.33
9.33
9.26
8.93
8.88
8.88
4.09
0.93
0.90
1.89
0.94
3.16
f1 (ppm)

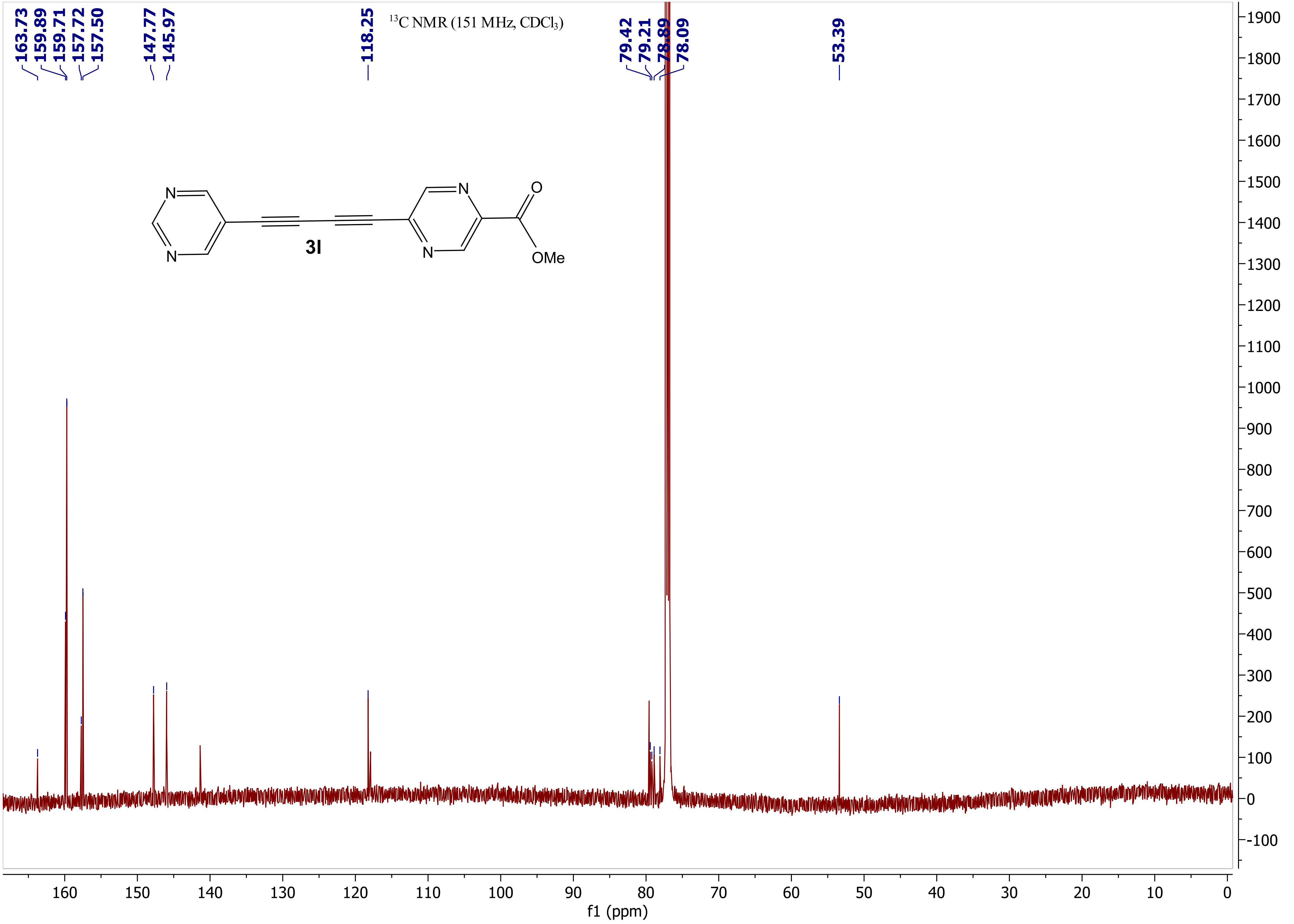

163.73
159.89
159.71
157.72
157.50
147.77
145.97
118.25
^{13}C NMR (151 MHz, $CDCl_3$)
79.42
79.21
78.89
78.09
53.39
3l
OMe
f1 (ppm)

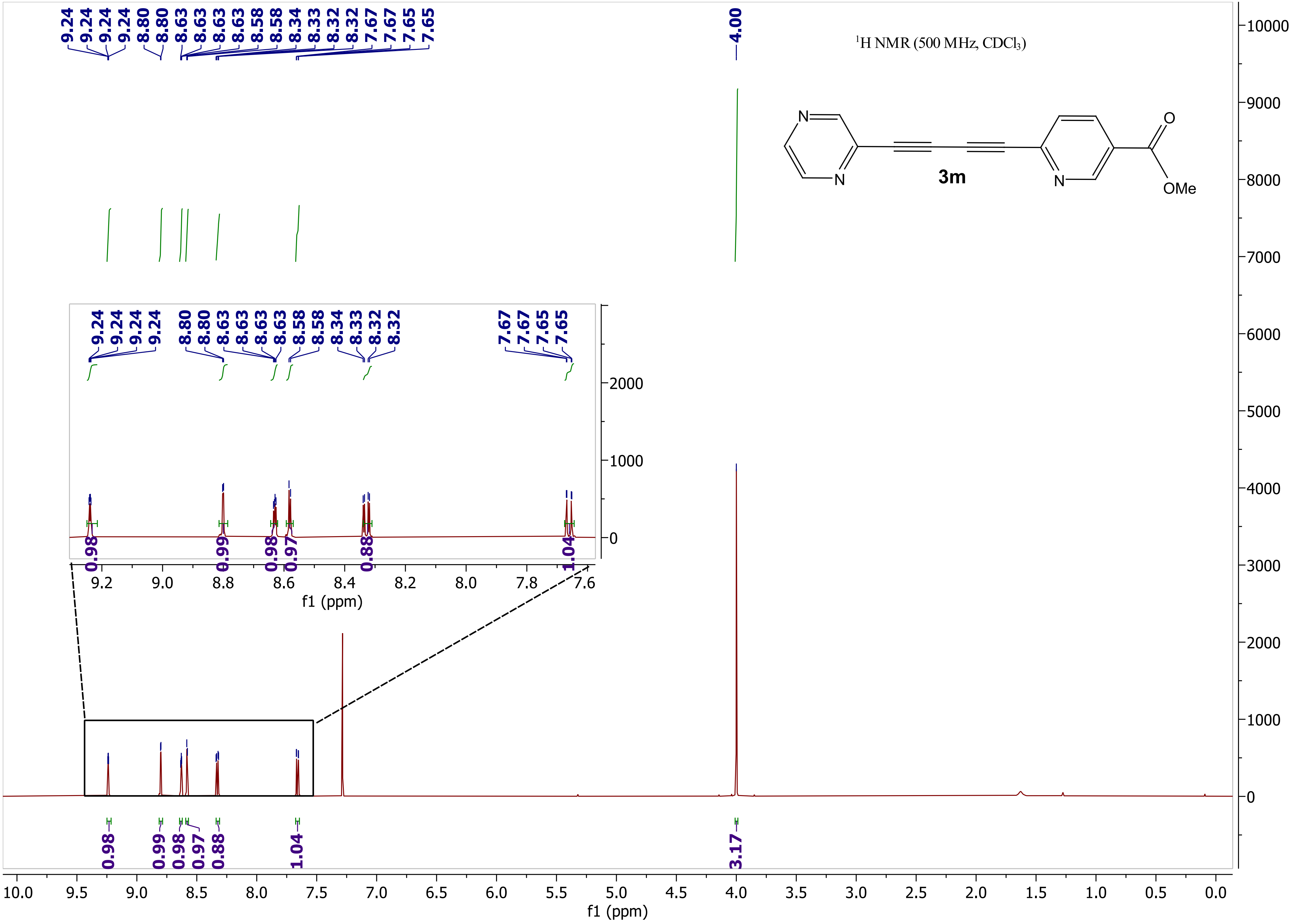

^{1}H NMR (500 MHz, CDCl3)
3m
OMe
f1 (ppm)

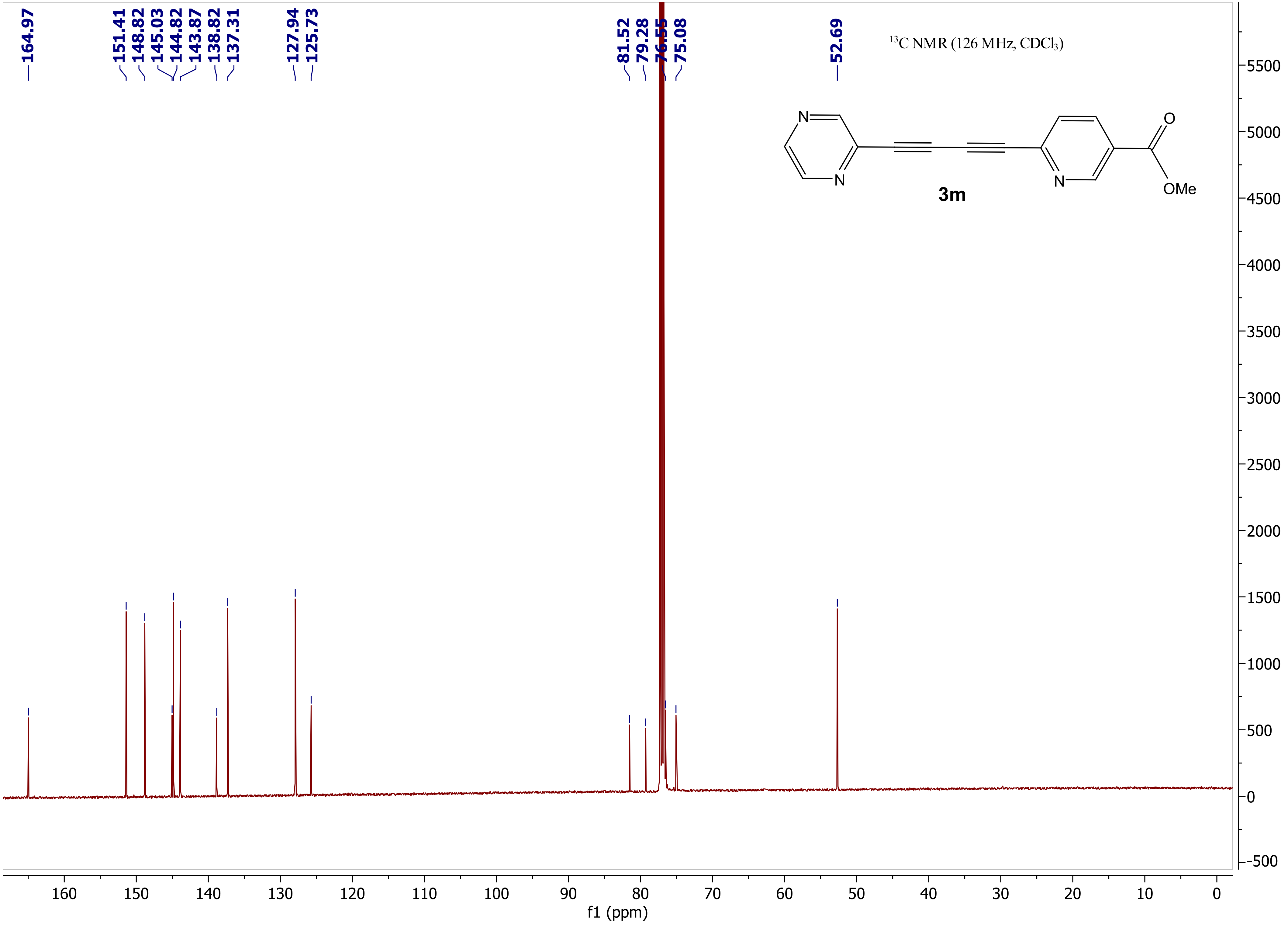

164.97
151.41
148.82
145.03
144.82
143.87
138.82
137.31
127.94
125.73
81.52
79.28
76.55
75.08
52.69
^{13}C NMR (126 MHz, CDCl$_3$)
N
N
N
O
OMe
3m
5500
5000
4500
4000
3500
3000
2500
2000
1500
1000
500
0
-500
160
150
140
130
120
110
100
90
80
70
60
50
40
30
20
10
0
f1 (ppm)

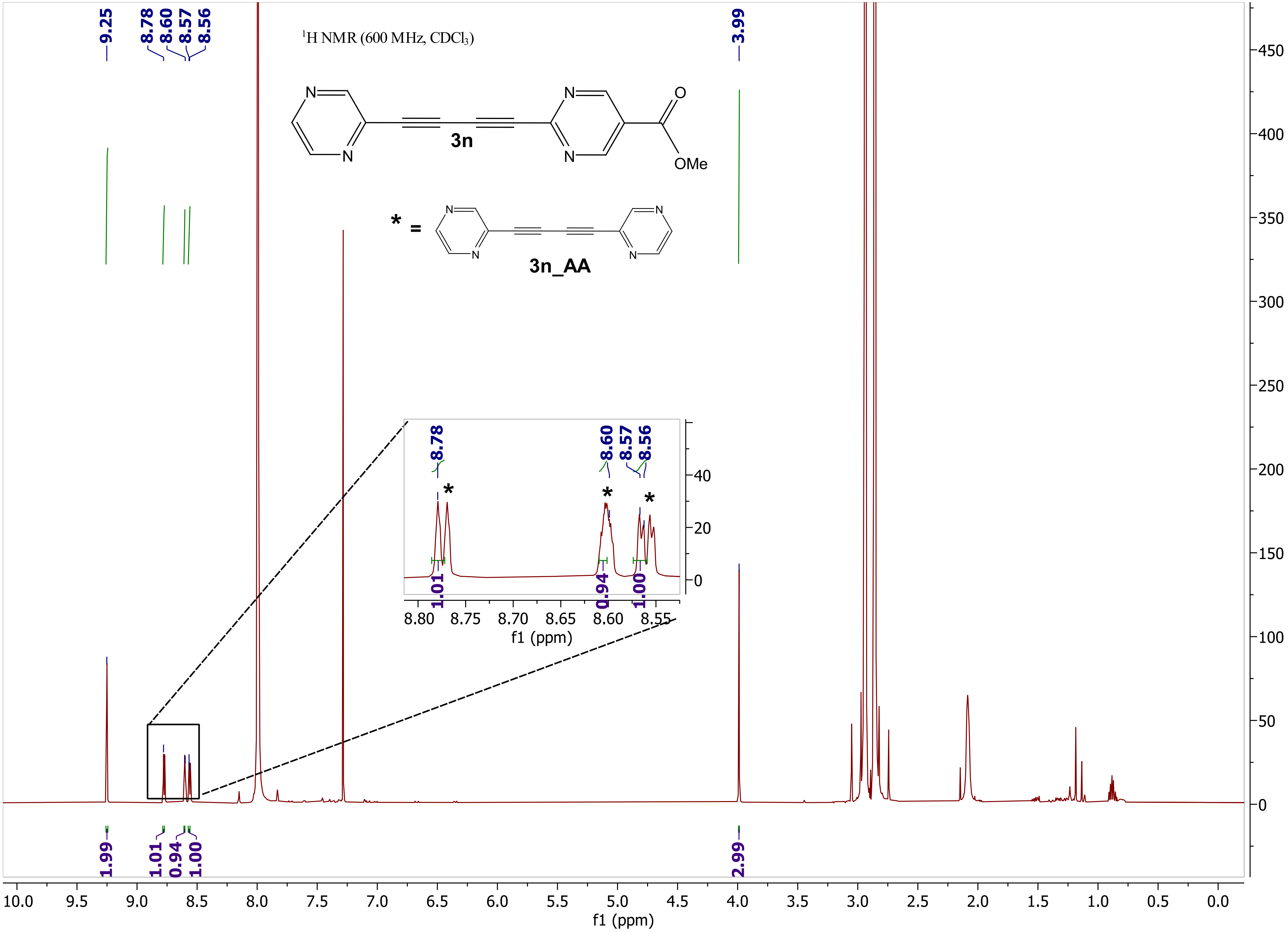

^{1}H NMR (600 MHz, CDCl3)
3n
* =
3n_AA
9.25
8.78
8.60
8.57
8.56
3.99
1.99
1.01
0.94
1.00
2.99
f1 (ppm)

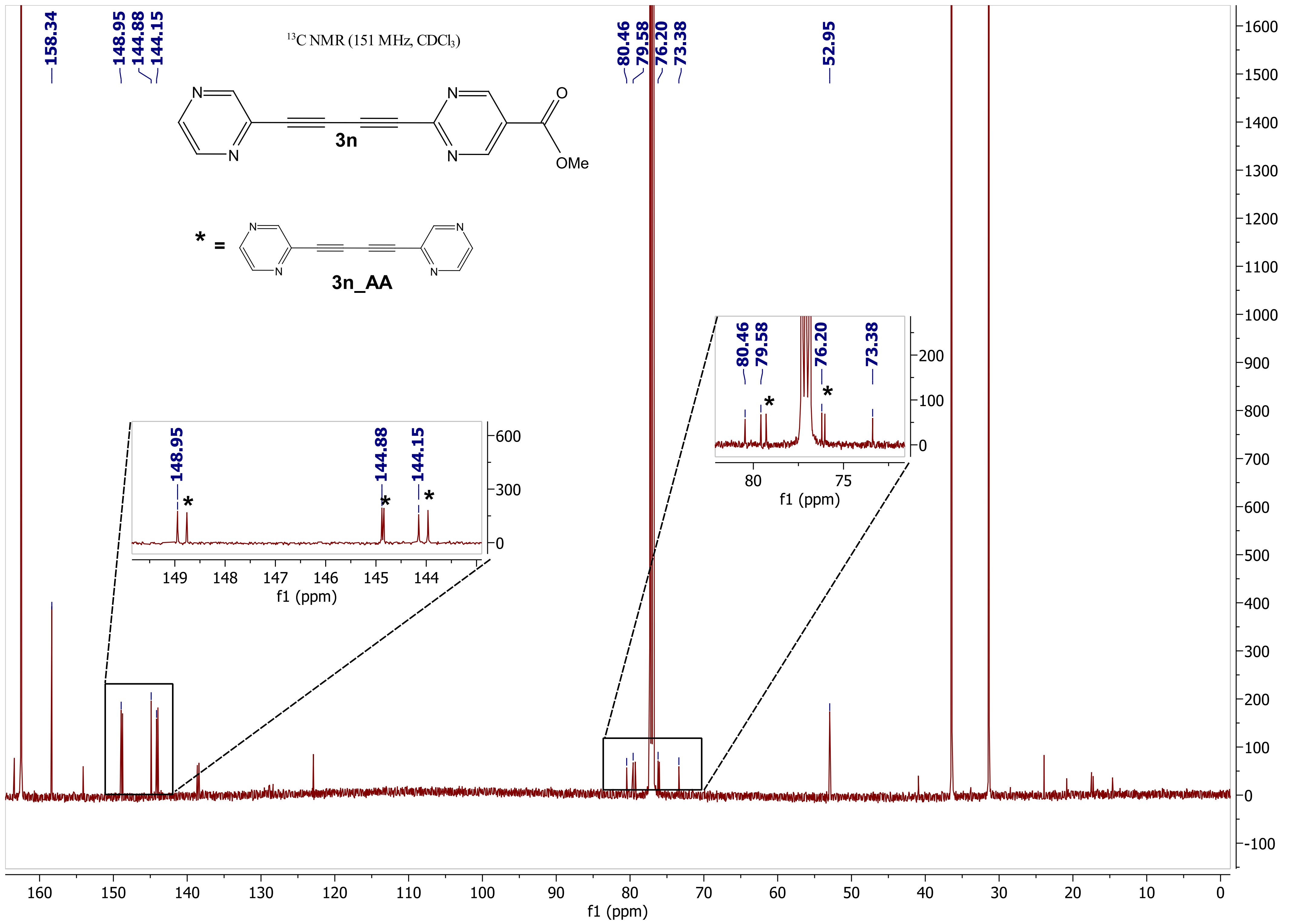

^{13}C NMR (151 MHz, $CDCl_3$)
3n
OMe
* =
3n_AA
158.34
148.95
144.88
144.15
80.46
79.58
76.20
73.38
52.95
f1 (ppm)

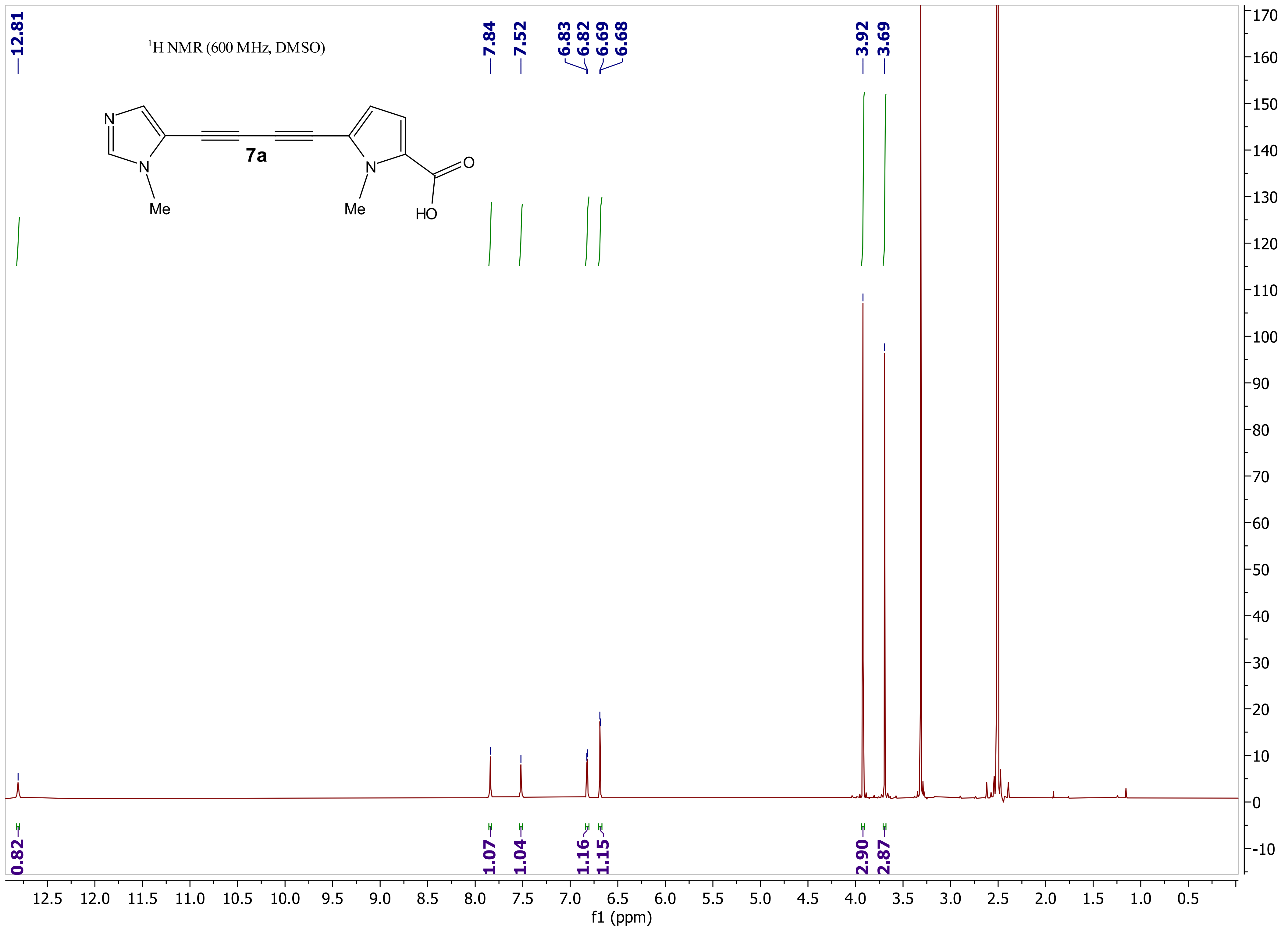

1H NMR (600 MHz, DMSO)
7a
N
N
Me
N
Me
O
HO
12.81
7.84
7.52
6.83
6.82
6.69
6.68
3.92
3.69
0.82
1.07
1.04
1.16
1.15
2.90
2.87
f1 (ppm)

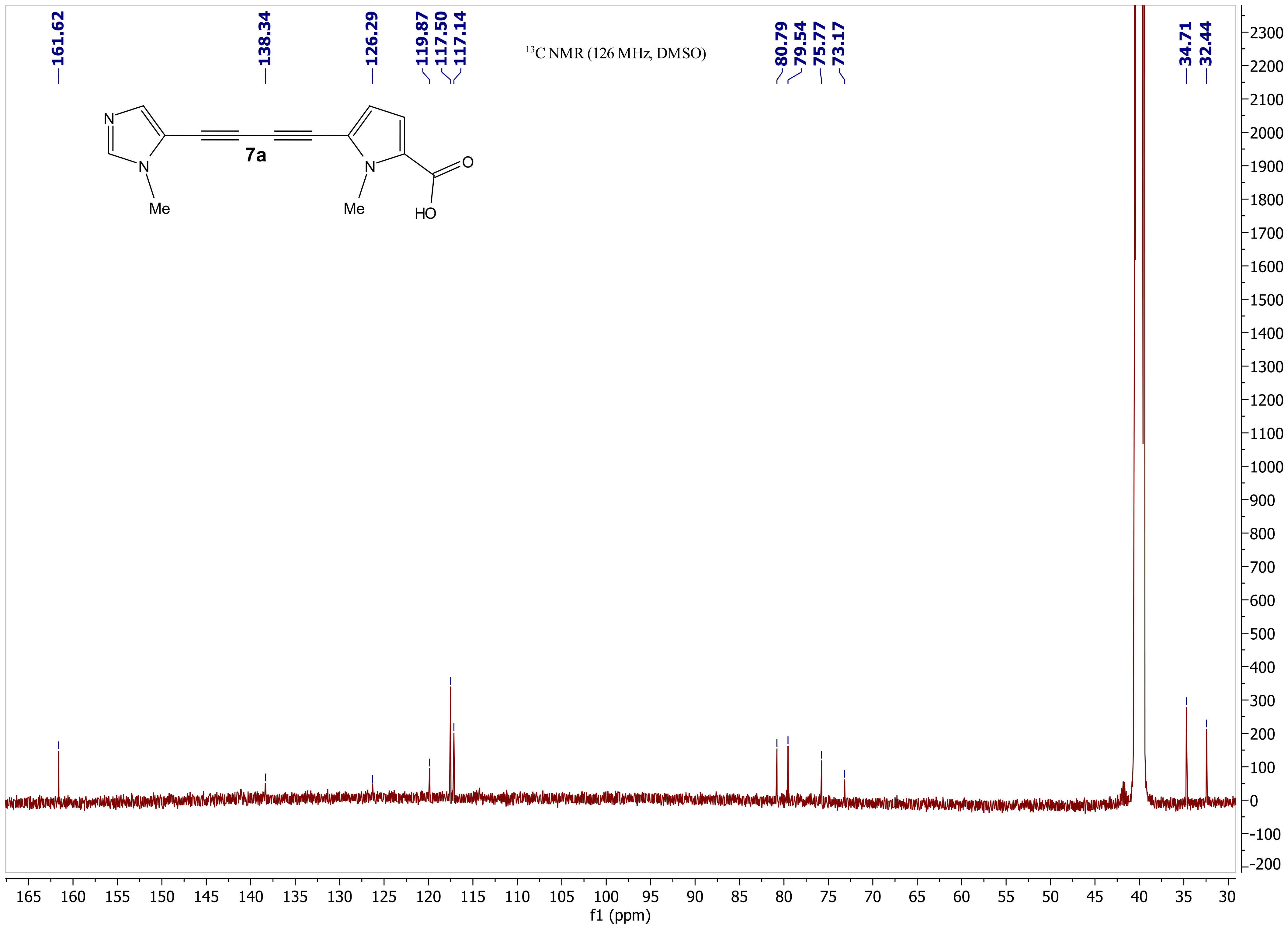
161.62
138.34
126.29
119.87
117.50
117.14
13C NMR (126 MHz, DMSO)
80.79
79.54
75.77
73.17
34.71
32.44
7a
N
N
Me
N
Me
O
HO
f1 (ppm)

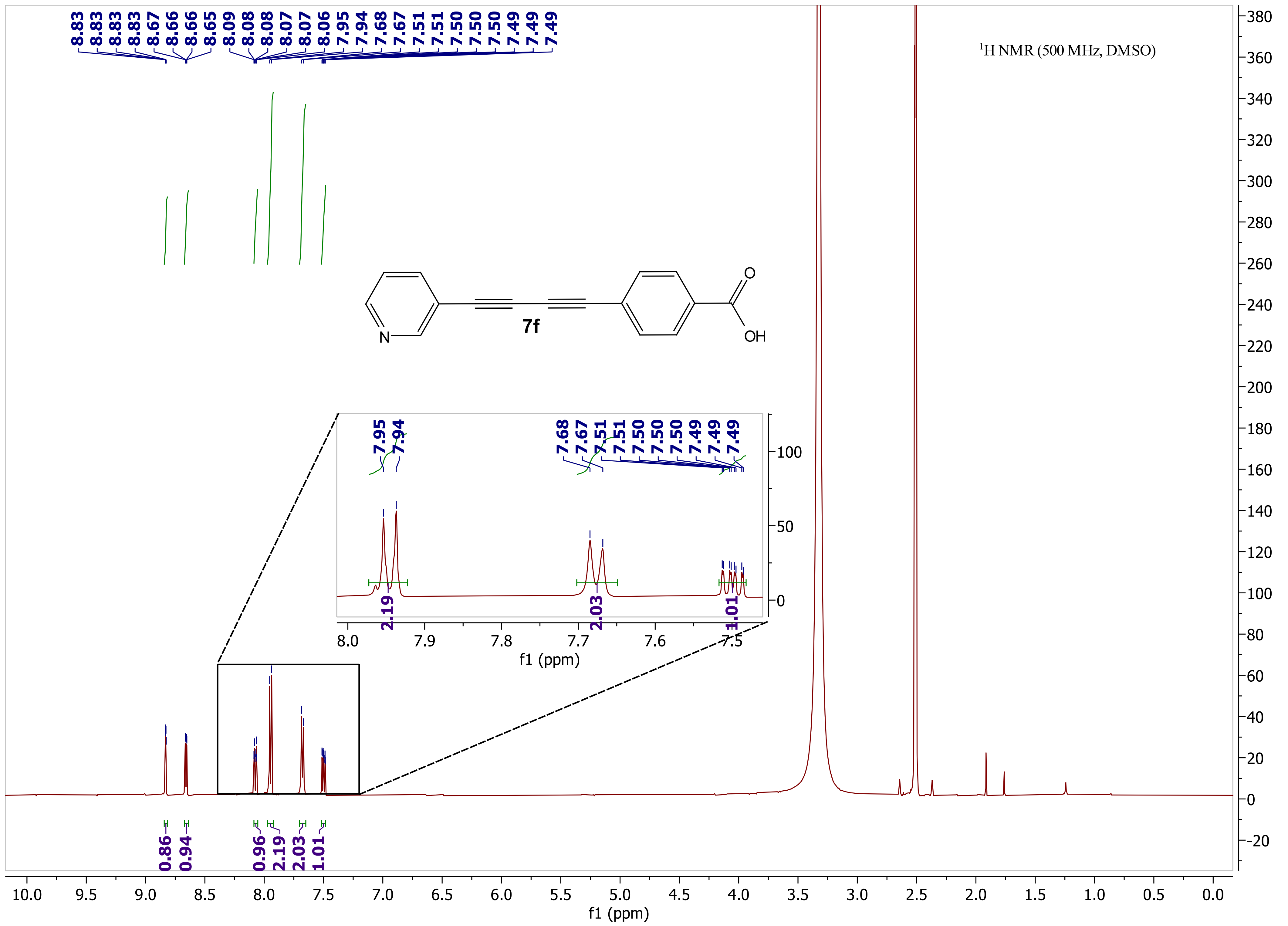

¹H NMR (500 MHz, DMSO)
7f
O
OH
N
8.83
8.83
8.83
8.83
8.67
8.66
8.66
8.65
8.09
8.08
8.08
8.07
8.07
8.06
7.95
7.94
7.68
7.67
7.51
7.51
7.50
7.50
7.50
7.49
7.49
7.49
0.86
0.94
0.96
2.19
2.03
1.01
f1 (ppm)

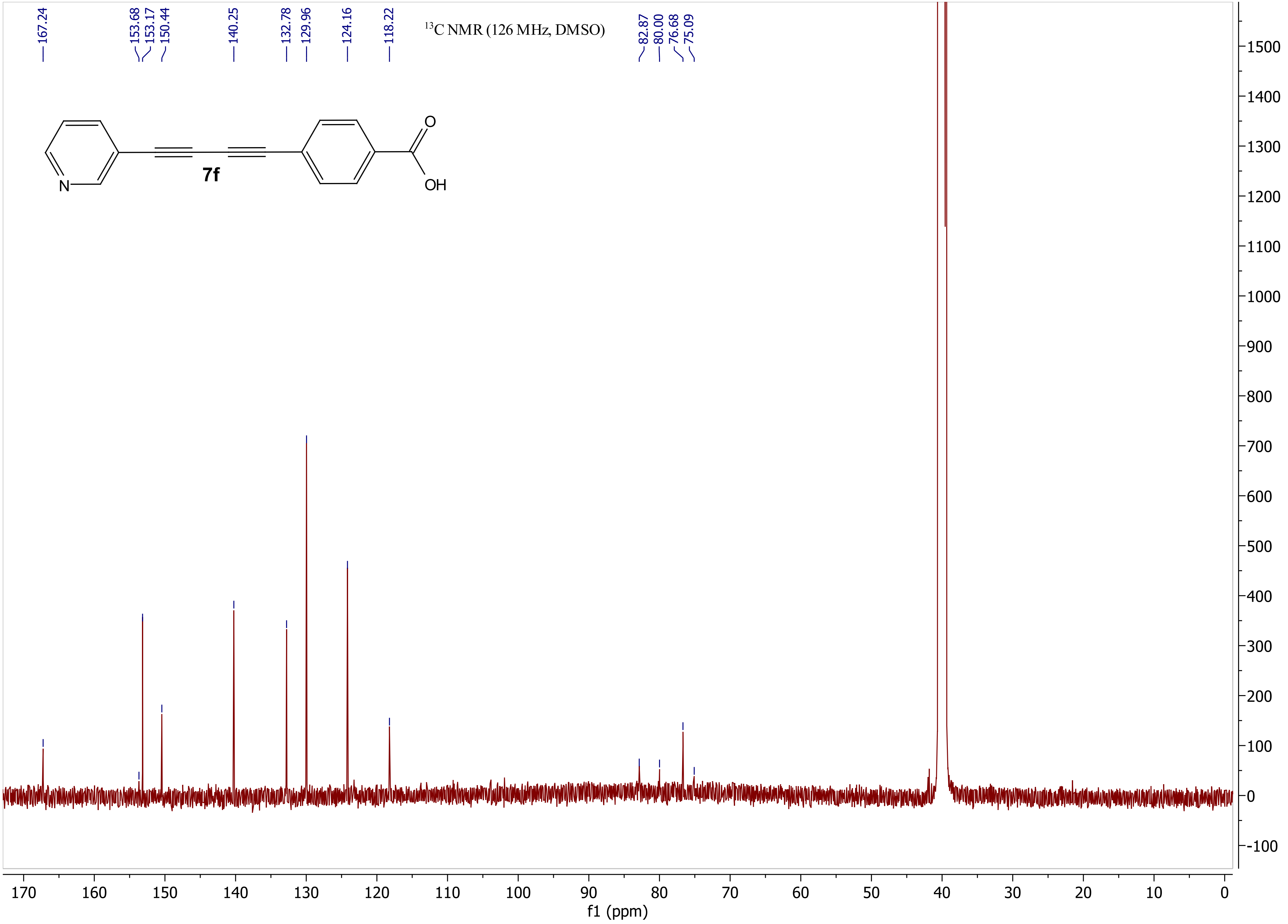

167.24
153.68
153.17
150.44
140.25
132.78
129.96
124.16
118.22
^{13}C NMR (126 MHz, DMSO)
82.87
80.00
76.68
75.09
7f
N
O
OH
f1 (ppm)

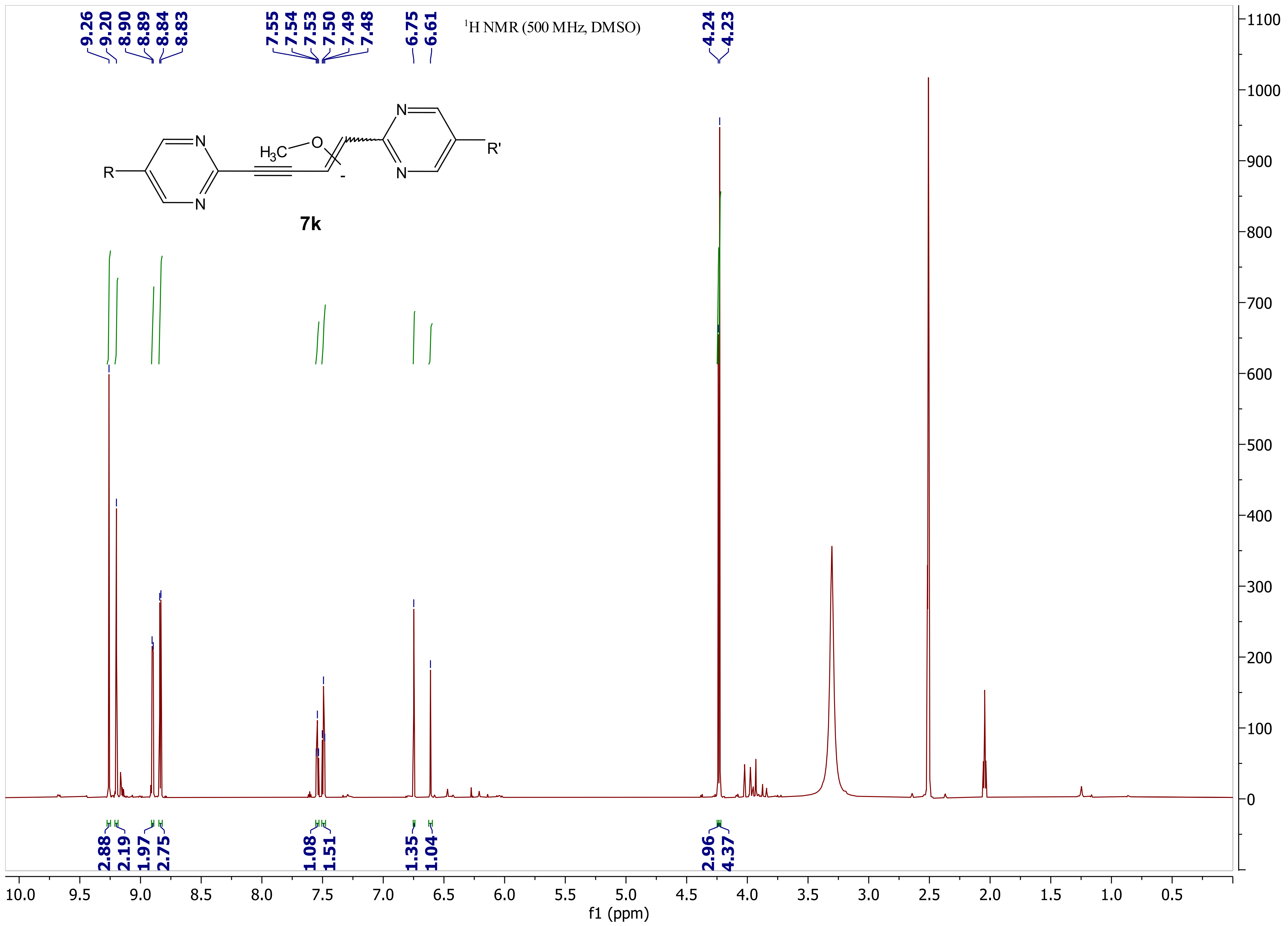
9.26
9.20
8.90
8.89
8.84
8.83
7.55
7.54
7.53
7.50
7.49
7.48
6.75
6.61
¹H NMR (500 MHz, DMSO)
4.24
4.23
N
N
N
N
R
R'
H3C
O
7k
2.88
2.19
1.97
2.75
1.08
1.51
1.35
1.04
2.96
4.37
f1 (ppm)

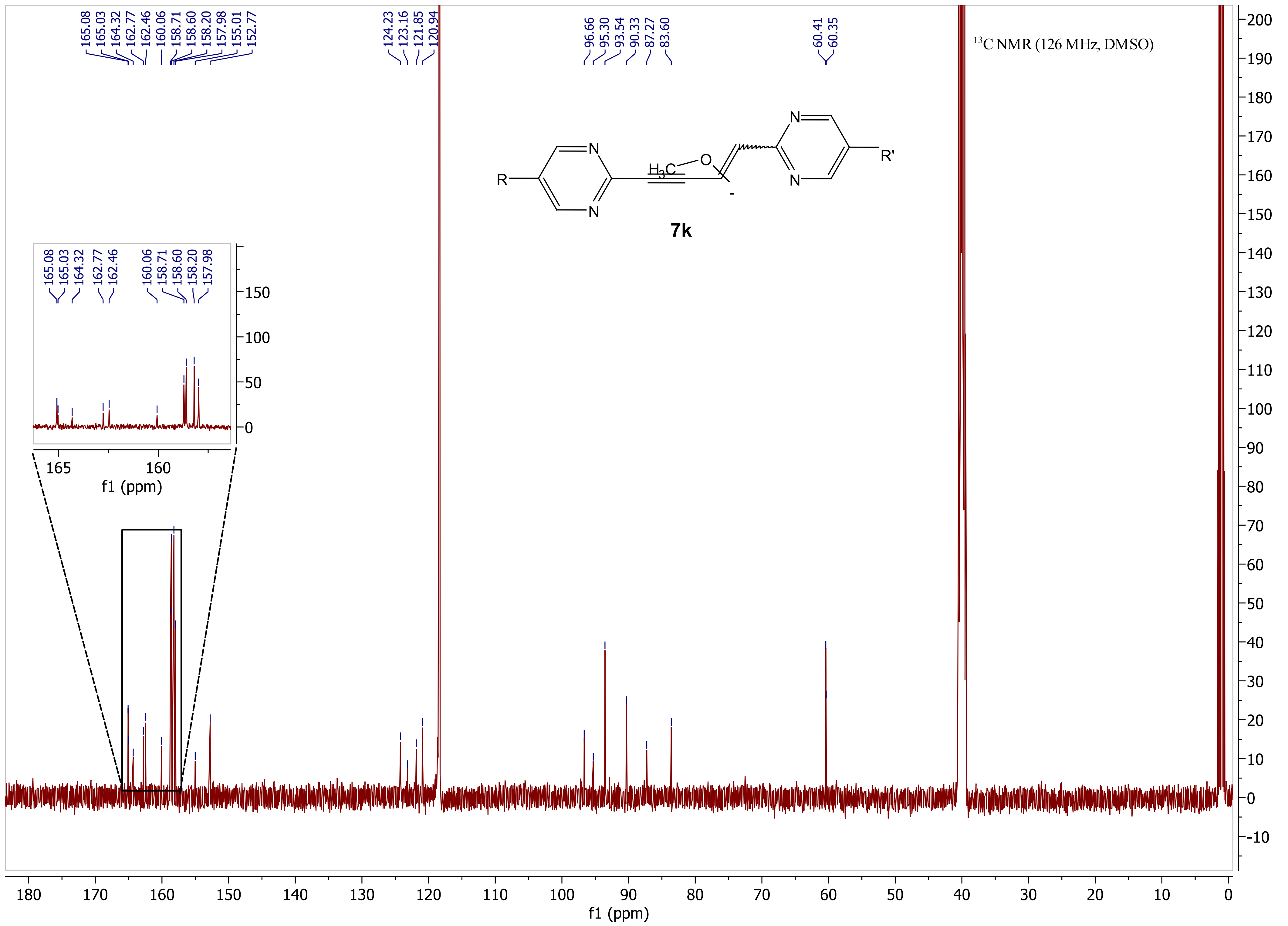

165.08
165.03
164.32
162.77
162.46
160.06
158.71
158.60
158.20
157.98
155.01
152.77
124.23
123.16
121.85
120.94
96.66
95.30
93.54
90.33
87.27
83.60
60.41
60.35
^{13}C NMR (126 MHz, DMSO)
R
N
N
H_3C
O
N
N
R'
7k
f1 (ppm)

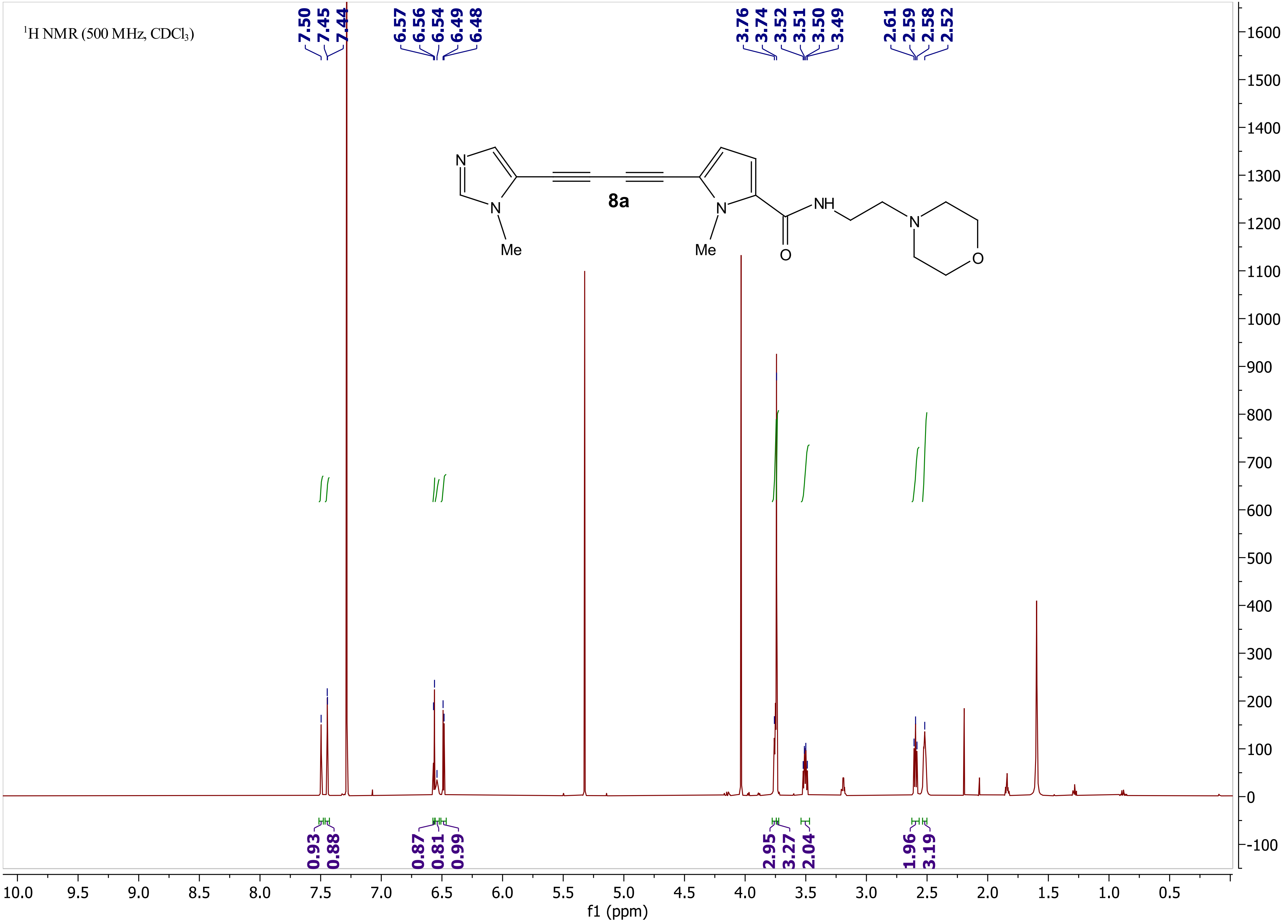

1H NMR (500 MHz, CDCl3)
7.50
7.45
7.44
6.57
6.56
6.54
6.49
6.48
3.76
3.74
3.52
3.51
3.50
3.49
2.61
2.59
2.58
2.52
N
N
Me
8a
N
Me
O
NH
N
O
0.93
0.88
0.87
0.81
0.99
2.95
3.27
2.04
1.96
3.19
f1 (ppm)

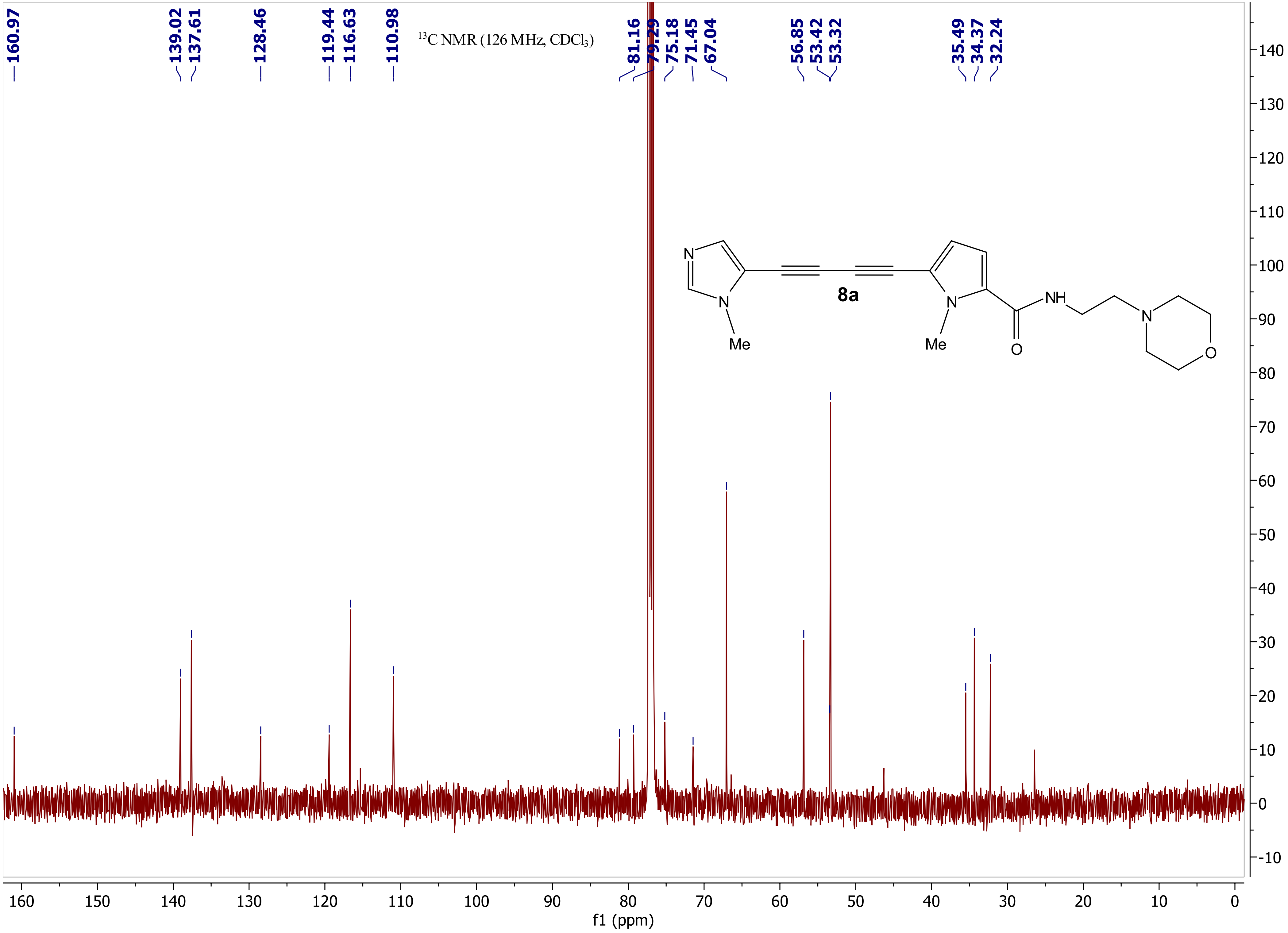

13C NMR (126 MHz, CDCl3)
160.97
139.02
137.61
128.46
119.44
116.63
110.98
81.16
79.29
75.18
71.45
67.04
56.85
53.42
53.32
35.49
34.37
32.24
N
N
Me
8a
N
Me
O
NH
N
O
f1 (ppm)
160 150 140 130 120 110 100 90 80 70 60 50 40 30 20 10 0
140 130 120 110 100 90 80 70 60 50 40 30 20 10 0 -10

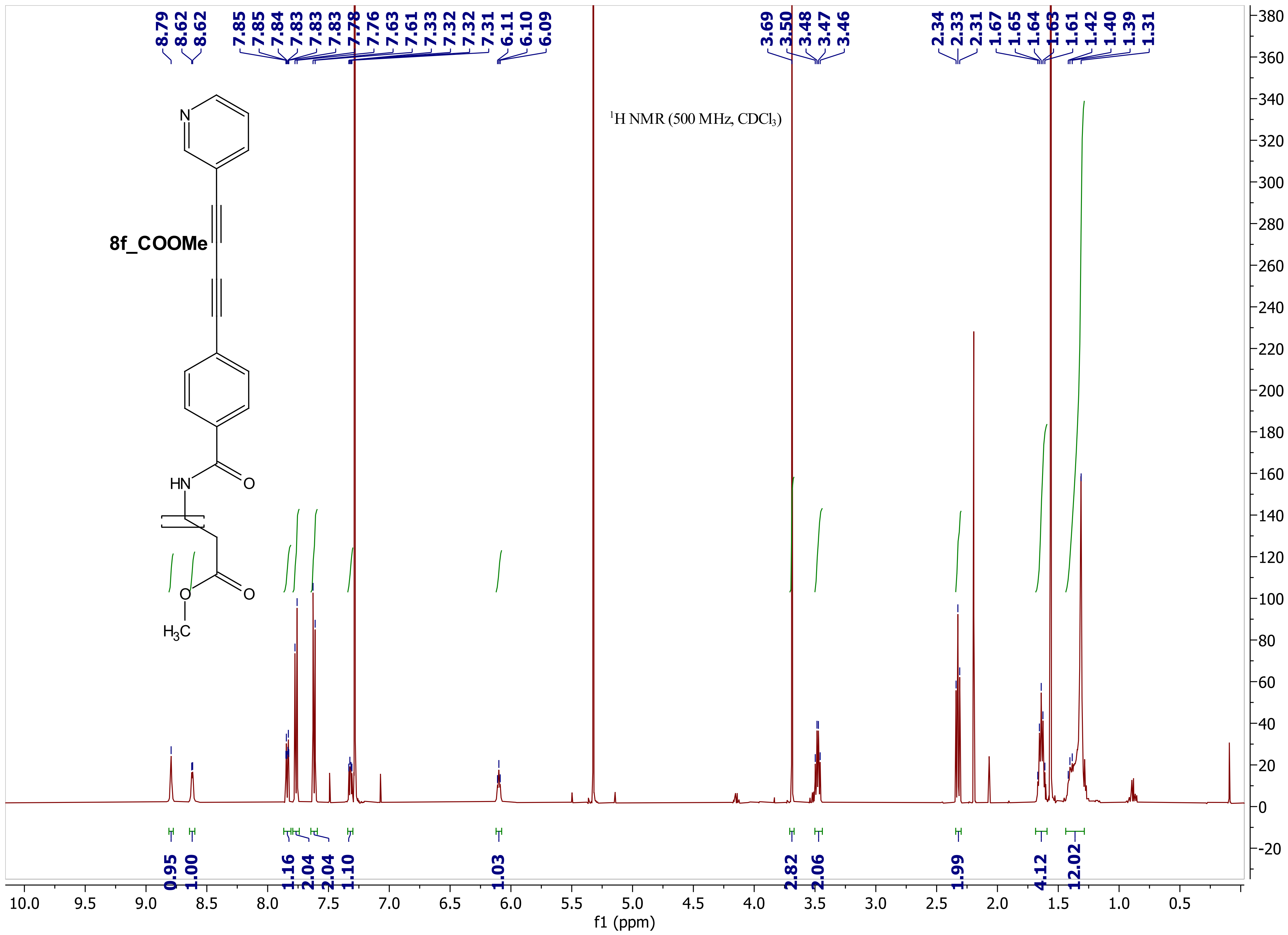
8.79
8.62
8.62
7.85
7.85
7.84
7.83
7.83
7.83
7.78
7.76
7.63
7.61
7.33
7.32
7.32
7.31
6.11
6.10
6.09
3.69
3.50
3.48
3.47
3.46
2.34
2.33
2.31
1.67
1.65
1.64
1.63
1.61
1.42
1.40
1.39
1.31
^{1}H NMR (500 MHz, $CDCl_3$)
8f_COOMe
N
HN
O
O
O
H_3C
0.95
1.00
1.16
2.04
2.04
1.10
1.03
2.82
2.06
1.99
4.12
12.02
f1 (ppm)

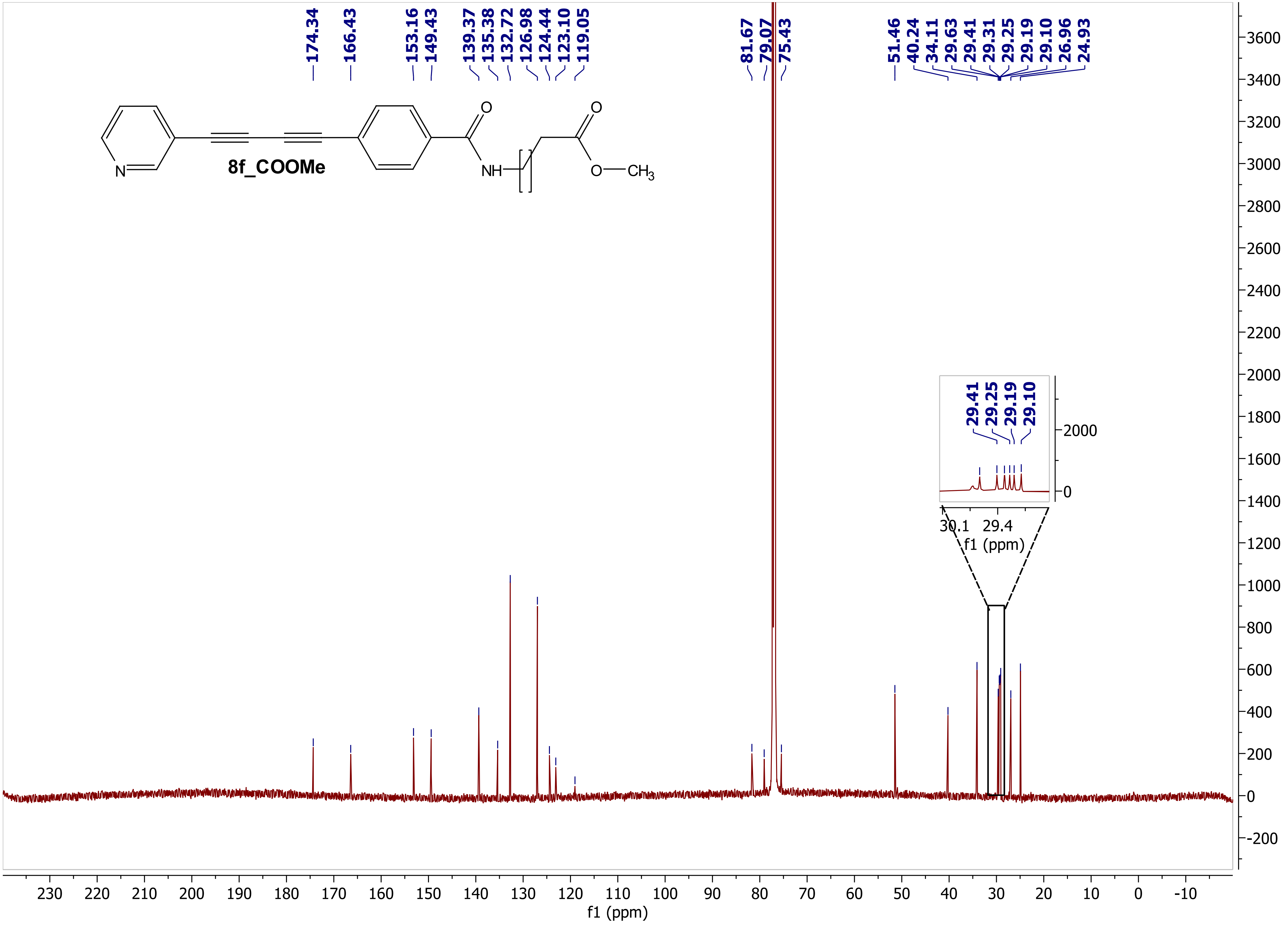
174.34
166.43
153.16
149.43
139.37
135.38
132.72
126.98
124.44
123.10
119.05
81.67
79.07
75.43
51.46
40.24
34.11
29.63
29.41
29.31
29.25
29.19
29.10
26.96
24.93
8f_COOMe
N
NH
O
O
O
CH_3
29.41
29.25
29.19
29.10
30.1 29.4
f1 (ppm)
f1 (ppm)

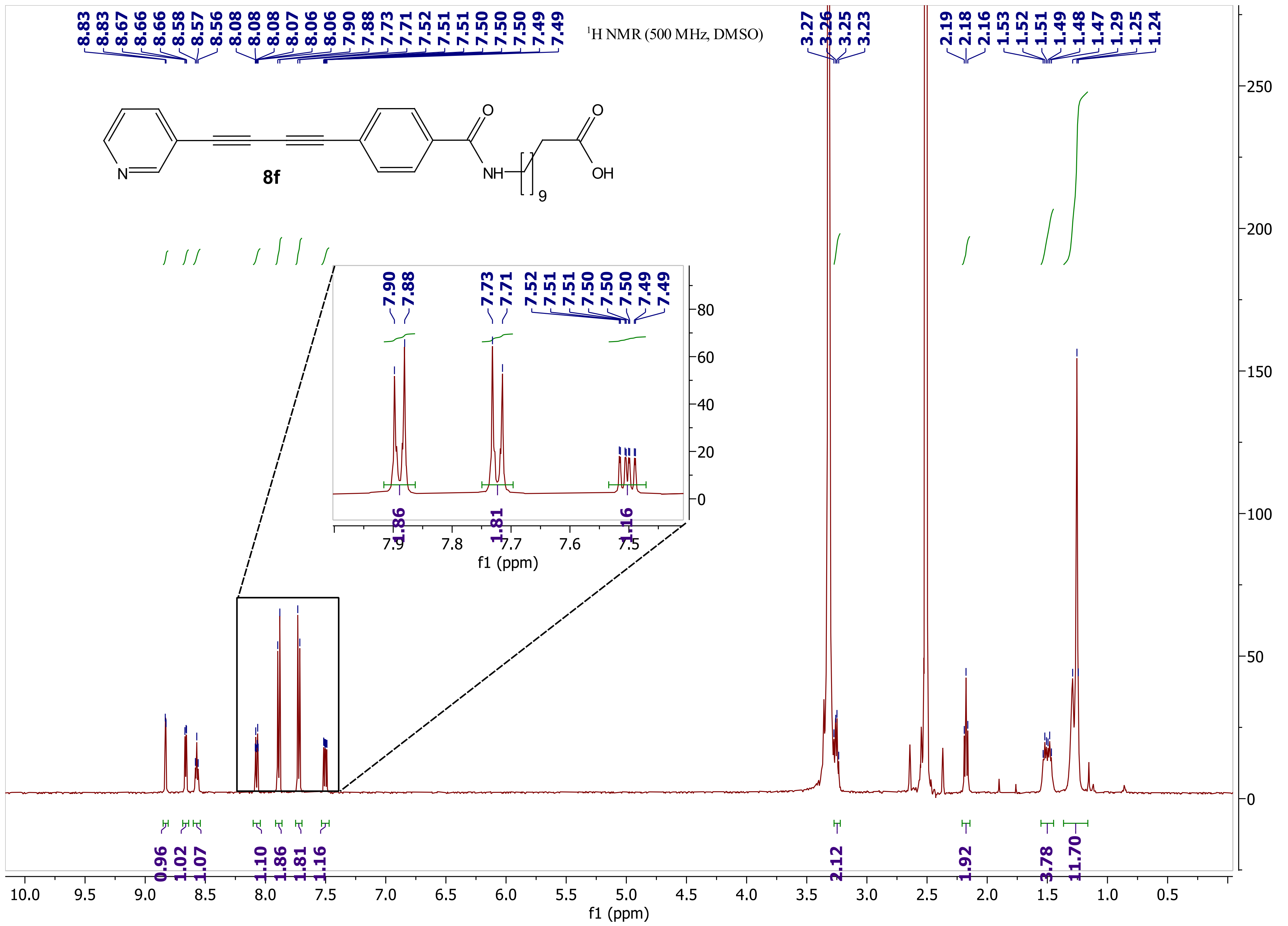

8.83 8.83 8.67 8.66 8.66 8.58 8.57 8.56 8.08 8.08 8.08 8.07 8.06 8.06 7.90 7.88 7.73 7.71 7.52 7.51 7.51 7.50 7.50 7.50 7.49 7.49
1H NMR (500 MHz, DMSO)
3.27 3.26 3.25 3.23
2.19 2.18 2.16 1.53 1.52 1.51 1.49 1.48 1.47 1.29 1.25 1.24
O
O
N
8f
NH
9
OH
7.90 7.88 7.73 7.71 7.52 7.51 7.51 7.50 7.50 7.50 7.49 7.49
1.86 1.81 1.16
f1 (ppm)
0.96 1.02 1.07 1.10 1.86 1.81 1.16 2.12 1.92 3.78 11.70
10.0 9.5 9.0 8.5 8.0 7.5 7.0 6.5 6.0 5.5 5.0 4.5 4.0 3.5 3.0 2.5 2.0 1.5 1.0 0.5
f1 (ppm)

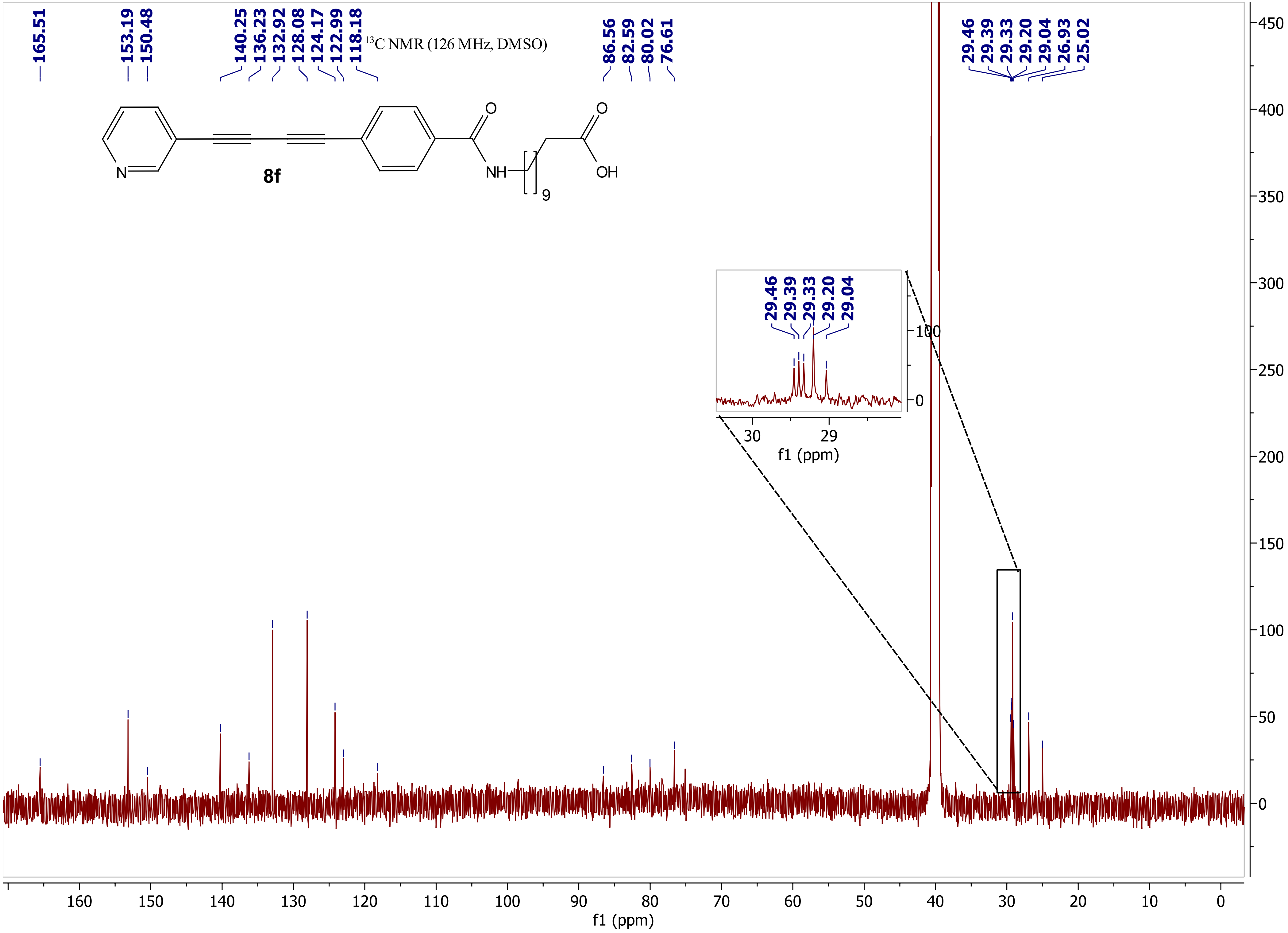

165.51
153.19
150.48
140.25
136.23
132.92
128.08
124.17
122.99
118.18
¹³C NMR (126 MHz, DMSO)
86.56
82.59
80.02
76.61
29.46
29.39
29.33
29.20
29.04
26.93
25.02
8f
NH
OH
O
N
9
f1 (ppm)
